# Unsupervised machine learning identifies distinct ALS molecular subtypes in post-mortem motor cortex and blood expression data

**DOI:** 10.1186/s40478-023-01686-8

**Published:** 2023-12-21

**Authors:** Heather Marriott, Renata Kabiljo, Guy P Hunt, Ahmad Al Khleifat, Ashley Jones, Claire Troakes, Project MinE ALS Sequencing Consortium, TargetALS Sequencing Consortium, Abigail L Pfaff, John P Quinn, Sulev Koks, Richard J Dobson, Patrick Schwab, Ammar Al-Chalabi, Alfredo Iacoangeli

**Affiliations:** 1 Department of Basic and Clinical Neuroscience, Maurice Wohl Clinical Neuroscience Institute, Institute of Psychiatry, Psychology and Neuroscience, King?s College London, London, SE5 9NU UK; 2 Department of Biostatistics and Health Informatics, Institute of Psychiatry, Psychology and Neuroscience, King’s College London, London, UK; 3 Perron Institute for Neurological and Translational Science, Nedlands, WA 6009 Australia; 4 Centre for Molecular Medicine and Innovative Therapeutics, Murdoch University, Murdoch, WA 6150 Australia; 5 MRC London Neurodegenerative Diseases Brain Bank, Institute of Psychiatry, Psychology and Neuroscience, King’s College London, London, UK; 6 Department of Pharmacology and Therapeutics, Institute of Systems, Molecular and Integrative Biology, University of Liverpool, Liverpool, L69 3BX UK; 7 NIHR Maudsley Biomedical Research Centre (BRC), South London and Maudsley NHS Foundation Trust and King’s College London, London, UK; 8 Institute of Health Informatics, University College London, London, UK; 9 NIHR Biomedical Research Centre, University College London Hospitals NHS Foundation Trust, London, UK; 10 GlaxoSmithKline, Artificial Intelligence and Machine Learning, Durham, NC USA; 11 King’s College Hospital, London, SE5 9RS UK

**Keywords:** Amyotrophic lateral sclerosis, Unsupervised and supervised machine learning, Precision medicine, Transcriptomics, Patient stratification, Biomarkers

## Abstract

Amyotrophic lateral sclerosis (ALS) displays considerable clinical and genetic heterogeneity. Machine learning approaches have previously been utilised for patient stratification in ALS as they can disentangle complex disease landscapes. However, lack of independent validation in different populations and tissue samples have greatly limited their use in clinical and research settings. We overcame these issues by performing hierarchical clustering on the 5000 most variably expressed autosomal genes from motor cortex expression data of people with sporadic ALS from the KCL BrainBank (N = 112). Three molecular phenotypes linked to ALS pathogenesis were identified: synaptic and neuropeptide signalling, oxidative stress and apoptosis, and neuroinflammation. Cluster validation was achieved by applying linear discriminant analysis models to cases from TargetALS US motor cortex (N = 93), as well as Italian (N = 15) and Dutch (N = 397) blood expression datasets, for which there was a high assignment probability (80–90%) for each molecular subtype. The ALS and motor cortex specificity of the expression signatures were tested by mapping KCL BrainBank controls (N = 59), and occipital cortex (N = 45) and cerebellum (N = 123) samples from TargetALS to each cluster, before constructing case-control and motor cortex-region logistic regression classifiers. We found that the signatures were not only able to distinguish people with ALS from controls (AUC 0.88 ± 0.10), but also reflect the motor cortex-based disease process, as there was perfect discrimination between motor cortex and the other brain regions. Cell types known to be involved in the biological processes of each molecular phenotype were found in higher proportions, reinforcing their biological interpretation. Phenotype analysis revealed distinct cluster-related outcomes in both motor cortex datasets, relating to disease onset and progression-related measures. Our results support the hypothesis that different mechanisms underpin ALS pathogenesis in subgroups of patients and demonstrate potential for the development of personalised treatment approaches. Our method is available for the scientific and clinical community at https://alsgeclustering.er.kcl.ac.uk.

**Supplementary Information:**

The online version contains supplementary material available at 10.1186/s40478-023-01686-8.

## Introduction

Amyotrophic lateral sclerosis (ALS) is a fatal neurodegenerative disease which displays considerable genetic heterogeneity. In approximately 90% of people with ALS, the disease is labelled as sporadic, without an apparent family history of the disease, with the remainder classed as familial [1]. Mutations in approximately 40 genes are known to be linked with ALS and can explain the majority of familial cases and approximately 20% of sporadic cases (SALS) [2]. However, a further 130 genes have been proposed to contribute to its risk or act as disease modifiers [3, 4]. ALS is also phenotypically variable, with differences in age and site of onset (spinal-innervated muscles vs. bulbar), the balance of upper and lower motor neuron involvement, rate of disease progression, and the presence of cognitive or non-motor symptoms [5]. Furthermore, a multitude of molecular processes have been implicated in its pathogenesis, in part due to the vast number of causative and modifier genes associated with ALS that code for diverse cellular functions [6]. It is therefore plausible that there is no universal approach to the treatment of people with ALS, especially given that many therapeutic strategies target specific molecular pathways. For example, the protective action of Riluzole on motor neurons is proposed to be the result of a reduction in glutamate-mediated excitotoxicity [7].

Machine learning approaches can be used to help us to understand the genetic and molecular complexity and heterogeneity of ALS, for example, by finding patterns in biological and clinical data that distinguish some groups of patients from the others. These subgroups can aid in identifying the best candidates for therapeutics which target specific biological processes. Machine learning methods have previously been applied to brain expression data to stratify people with SALS into molecular subgroups [8–11], and has led to valuable insight into the genomic heterogeneity of ALS. However, some of these studies integrated samples from different brain regions to generate clusters and characterise their molecular architectures [9, 11, 12]. This design might present limitations in reflecting motor neuron-related ALS pathogenesis. Other studies adopted a case-control framework [8–10], which could lead to reduced power given the potential decoupling between mechanisms underlying risk and phenotype variability [13–15]. Furthermore, previous work has not been validated in independent datasets or in different populations and did not investigate whether molecular subtypes identified in post-mortem brains are reflected in other tissues available pre-mortem. Such factors have greatly limited the applicability and impact of these results. We therefore aimed to identify and validate molecular and phenotypic patterns across multiple independent datasets, tissue types and populations, to generate gene expression derived molecular subtypes of ALS that can be utilised for stratification in the design and interpretation of future research and clinical studies.

## Methods

### Study cohorts

We obtained raw post-mortem motor cortex bulk RNA sequencing data in FASTQ format from two datasets. The first, which was used to generate the clusters, consisted of 112 people from the UK with SALS from King’s College London and the MRC London Neurodegenerative Diseases Brain Bank (KCL BrainBank) [16]. We additionally obtained matching whole-genome sequencing (WGS), methylation data and clinical data for the KCL BrainBank samples from Project MinE to perform subgroup clinical and omics-based phenotype analysis [17]. For validation of KCL BrainBank-derived cluster expression signatures, 168 US motor cortex samples from 93 people with SALS of North European ancestry, present in the Target ALS Human Post-mortem Tissue Core (TargetALS) were used. For further validation of KCL BrainBank-derived clusters, we also processed two peripheral blood mononuclear cell (PBMC) datasets; bulk RNA sequencing data in FASTQ format of 15 Italian people with SALS (Zucca) [18], and hg18-aligned log2 transformed and quantile normalised microarray gene probe intensities of 397 Dutch people with ALS (van Rheenen) [19]. To determine if the clusters could discriminate between ALS cases and controls, we also used RNA sequencing data in FASTQ format from 59 healthy controls in the KCL BrainBank. Finally, we obtained raw transcript counts for two additional TargetALS case datasets to determine if the expression signatures reflected a motor cortex-specific disease process, which included 45 samples from the occipital cortex, and 128 samples from 123 individuals from the cerebellum. Sequencing specific methods are described in more detail in the Supplementary Methods. The basic demographics of each of the datasets used in this study are detailed in Supplementary Table 1. 

### Bulk RNA sequencing data Processing

Paired FASTQ files from KCL BrainBank, TargetALS motor cortex and Zucca datasets were interleaved using BBMap reformat v38.18.0 under default options before adapters were right-clipped and both sides of each read were quality-trimmed with BBMap bbduk v38.18.0 [20]. The interleaved FASTQ files were aligned to hg38 using STAR v2.7.10a under default settings [21]. Raw transcript counts for each gene were then quantified using HTSeq [22] on a sample-wise basis before merging into dataset-specific matrices. For the TargetALS occipital cortex and cerebellum datasets, transcripts were quantified with Salmon [23] before being converted into gene-specific expression counts with tximport [24]. For all datasets, raw counts were normalised using the *estimateSizeFactors* function of DESeq2, before lowly expressed genes and non-autosomal chromosomes were removed. Expression values were then standardized using the variance stabilising transformation *(vsd)* function in DESeq2 [25].

### Hierarchical clustering of KCL samples

Our hierarchical clustering was based on a protocol that was previously used to identify cortical molecular phenotypes of ALS [11]. Briefly, the 5000 most variably expressed genes, selected based on the highest median absolute deviation values, were extracted from the KCL BrainBank gene expression matrix. Unsupervised hierarchical clustering was then performed with the non-smooth negative factorisation (nsNMF) algorithm, using helper functions outlined in the SAKE package [26]. The optimal number of clusters was identified by running nsNMF with 100 runs and 1000 iterations for different values of k (two to ten). Cluster estimation results are available in Supplementary Fig. 1. We then ran the nsNMF algorithm with k = three, 100 runs and 1000 iterations, with the resulting consensus matrix showing a clear separation of samples (Supplementary Fig. 2). Informative gene and sample assignment for each of the three clusters was then extracted. The list of informative genes for each cluster was then used to characterise their molecular phenotypes by performing gene enrichment analysis using the GProfiler2 R package [27]. Genes from the whole KCL expression matrix were used as a custom gene background. The default g:SCS algorithm was used to assess significant enrichment for several process and pathway categories in the following databases: Gene Ontology (Biological Process (GO:BP), Molecular Function (GO:MF) and Cellular Component (GO:CC)), Kyoto Encyclopaedia of Genes and Genomes (KEGG), Reactome, CORUM, TRANSFAC, and miRTarBase. Additionally, MetaCore^™^ (available at https://portal.genego.com) was used to construct cluster-specific gene pathway networks using the *’analyze network’* algorithm under default options, with the network that displayed the highest significance selected as the one that most defines the cluster.

### Validation of KCL BrainBank-derived clusters

To determine if the informative genes which defined each cluster could be used to successfully stratify samples in other ALS datasets, we applied linear discriminant analysis (LDA) models to the TargetALS, Zucca, and van Rheenen ALS datasets, using the MASS R package [28]. Each dataset-specific model was trained using the intersection of dataset-specific and informative cluster genes, which yielded 470, 381, and 535 genes for TargetALS, Zucca and van Rheenen, respectively. The linear discriminants were derived from the KCL BrainBank gene and sample cluster assignments. The same approach was carried out for KCL BrainBank controls and the occipital cortex and cerebellum of people with ALS from TargetALS, with 787, 651 and 622 genes shared between each respective dataset and KCL BrainBank cases. Classification probability was evaluated based on the average dataset-specific posterior probabilities of cluster assignment. Cluster stability was then assessed using bootstrapping, implemented in the resample function of the scikit-learn package. Resampling with replacement was performed with 1000 iterations. For each iteration, the median and 95% confidence intervals for accuracy and silhouette of the cluster assignment was collected, before being averaged to form the final estimate.

As linear discriminant analysis is constrained to assign every sample to one class, we performed additional analyses to confirm that controls and post-mortem expression data from different brain regions assigned to each molecular phenotype can be distinguished from motor-cortex case samples. To determine the specificity of the cluster one signature for ALS in the KCL BrainBank dataset, we performed case-control differential expression analysis of the 131 genes which constituted its signature using DESeq2 [25], applying the same standardisation and normalisation procedure that was used to pre-process the expression data for hierarchical clustering. Differentially expressed genes were identified via the independent hypothesis weighting multiple testing approach using Benjamini-Hochberg adjustment, with p-value < 0.050 denoting significance. For both the KCL BrainBank cluster 1 case-control dataset and the TargetALS motor-occipital and motor-cerebellum case datasets for all clusters, we built logistic regression classifiers with ten-fold cross validation using the scikit-learn and imblearn Python libraries [29, 30] to ascertain the discriminative ability of each cluster-specific gene signature. Three scenarios were employed: (1) using all of the cluster-specific genes present in each dataset, (2) removing multicollinear features using the SelectNonCollinear function of the collinearity package with a correlation threshold of 0.4 and ANOVA F-value as the scoring parameter [31], and (3) extracting the uncorrelated features present in all folds to subset the cluster-specific signatures before retraining the model. For all scenarios, the data was firstly normalised by removing the mean and scaling to unit variance using StandardScaler before oversampling was performed to address potential class imbalance using the synthetic-minority oversampling technique (SMOTE) function of *imblearn*. For each scenario, the best hyperparameters were selected using GridSearchCV with ROC_AUC as the scoring parameter, before the model was evaluated using the average ROC_AUC, precision, recall, and F1-score over all folds. Both hyperparameter tuning and cross-validation was performed using StratifiedKFold with ten splits and shuffling of the samples within each cluster.

We also performed two additional analyses to determine the robustness of our discovery and validation methods. The first analysis involved performing hierarchical clustering on the top 5000 variably expressed genes in TargetALS motor cortex samples to obtain informative-gene based cluster assignments in the same way as described for KCL BrainBank. By doing this, TargetALS was the discovery dataset, whilst KCL BrainBank served as the replication dataset. We then analysed the overlap between the original assignments and new assignments to gather the natural grouping of samples. To support the discriminative performance of the KCL BrainBank classifier, we constructed ten additional logistic regression classifier models with 10-fold cross validation using the cases and controls assigned to cluster 1. Each classifier was supplied with 131 randomly sampled genes from the transformed expression matrix. The resulting performance metrics over all ten classifiers were averaged to form the final estimate before the performance was compared to the cluster 1 expression signature specific classifier.

### Cell type deconvolution analysis of motor cortex case datasets

To assess whether the molecular phenotypes we identified in bulk RNAseq data could also be reflective of cell composition, we used the MuSiC R package (v1.0.0; [32]) to derive cell proportions in the KCL BrainBank and TargetALS case datasets for the following cell types: astrocytes, endothelial cells, microglia, neurons, and oligodendrocytes. We performed deconvolution with the raw RNAseq counts. The single-cell RNAseq reference dataset which was used to derive expression information for each cell type consisted of 8 adults and 4 embryonic samples (16–18 weeks gestational age) from the temporal lobe [33], which was downloaded via the scRNAseq R package (v2.14.0; [34]). Differences in composition between clusters in each dataset were assessed using one-way ANCOVA corrected for sex assigned at birth and age of death, with post-hoc Tukey’s test used to determine subcluster-specific trends. The normality of each variable for each dataset was assessed using the Shapiro-Wilk test, with any variables that were non-normally distributed (p-value < 0.050) being log-transformed before analysis.

### Subgroup phenotype analysis

To reveal and compare the phenotypic architecture of each cluster, we extracted several clinical and omics variables from each case-specific dataset. We performed the chi-square test of independence to assess if there were differences in the proportion of *C9orf72*-positive, limb-onset, bulbar-onset, and combined limb and bulbar onset cases between each of the clusters in the KCL BrainBank and TargetALS datasets, the limb: bulbar ratio in the van Rheenen datasets, and the male:female ratio in all four case-only datasets. A p-value < 0.05 denotes significance. Due to variations in the phenotypic information collected and accessibility of other omics data, we could not extract some phenotypic variables for all datasets. A breakdown of the collected phenotypic variables for each motor cortex and blood ALS dataset is available in Supplementary Table 2. Transcriptional age acceleration was calculated by using RNAAgeCalc to obtain tissue-specific transcriptional age estimates for each dataset [35], before being subtracted from the chronological age (age at death for KCL BrainBank and TargetALS, age at last blood draw for Zucca and van Rheenen). Telomere length and mitochondrial DNA copy number were obtained by applying TelSeq v0.0.2 [36] and fastMitoCalc v1.2 [37] to the whole-genome sequencing BAM files, respectively. Biological age was estimated from the methylation beta-value matrix using CorticalClock [38], before acceleration was calculated by subtracting each value from the age at death. Differences between clusters were assessed using one-way ANCOVA corrected for sex assigned at birth, with post-hoc Tukey’s test used to determine subcluster-specific trends. The normality of each variable for each dataset was assessed using the Shapiro-Wilk test, with any variables that were non-normally distributed (p-value < 0.050) being log-transformed before analysis. Additionally, we applied a Cox proportional-hazards model to assess differences in age of onset among clusters by combining samples from both KCL BrainBank and TargetALS datasets, with which p-value < 0.050 denotes significance.

### Code availability

The implementation of our class assignment model based on the KCL BrainBank data, can be used to assign class membership to new expression samples (both microarray and RNAseq) and is publicly available at https://alsgeclustering.er.kcl.ac.uk. The code for the analyses performed in this study is available at https://github.com/KHP-Informatics/HierarchicalClusteringALS/.

## Results

**Fig. 1:**
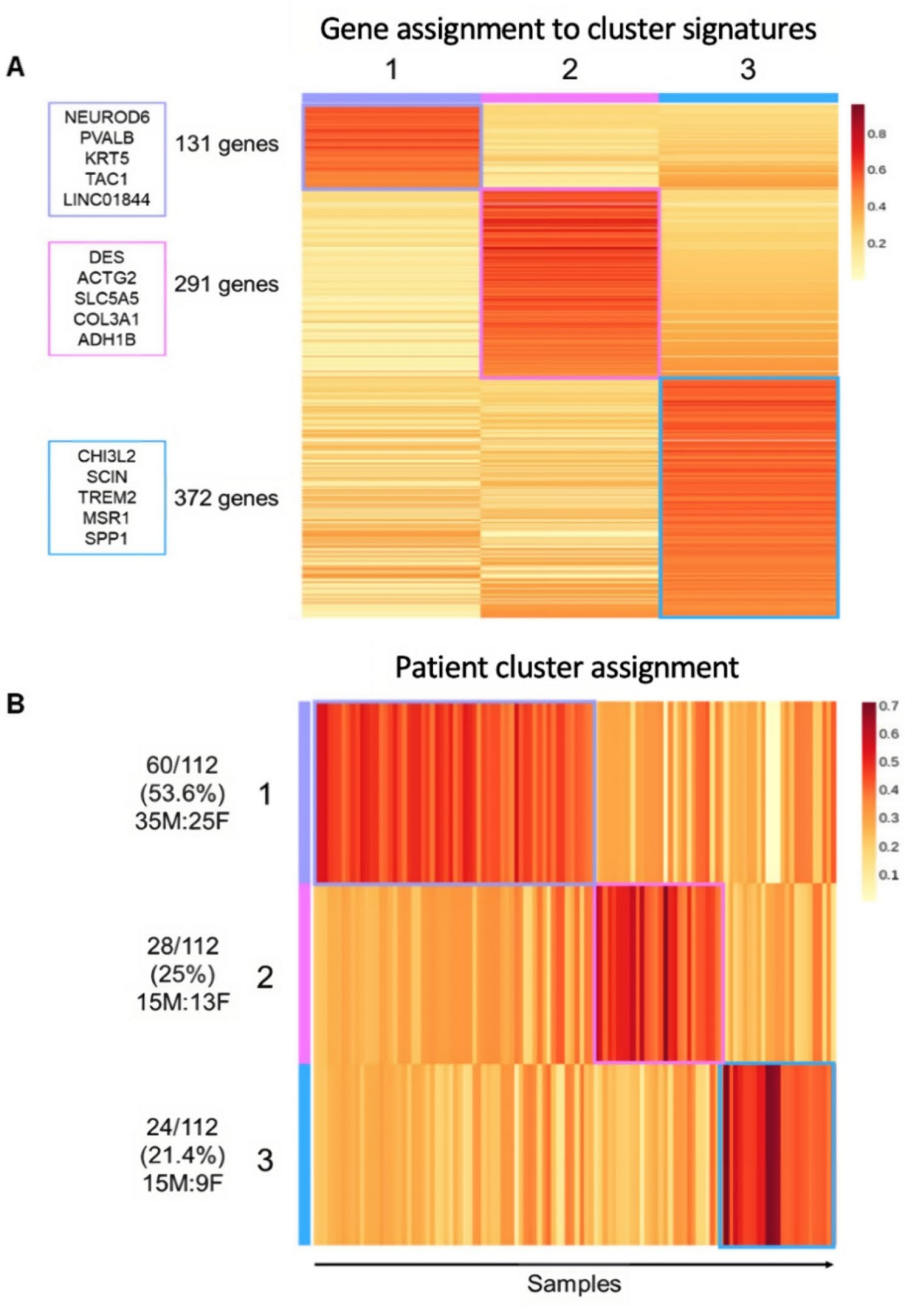
Informative gene and sample assignment for KCL BrainBank-generated clusters. (**A**) Number of the 794 informative genes uniquely assigned to each cluster, with the top five contributing genes (defined by posterior probability) listed at the side. (**B**) Distribution of cluster assignment of people with SALS alongside the male:female ratio, based on sex assigned at birth. The coloured scale refers to the posterior probability value

The nsNMF algorithm identified 794 of the 5000 most variably expressed genes as being the most informative for defining the clusters. Each informative gene was uniquely assigned to one cluster, yielding three distinct clusters, each with a unique gene expression profile. There were 131, 291, and 372 genes which defined clusters one, two, and three, respectively (Fig. 1A). The full list of genes which comprise each cluster are available in Supplementary Table 3. The larger proportion of the people with ALS (60; 53.6%) were assigned to cluster one, followed by cluster two (28; 25%) and cluster three (24; 21.4%), without substantial differences in male:female ratio in each cluster, based on sex assigned at birth (Fig. 1B) or the proportion of males and females assigned to the clusters (X^2^ = 0.43, p-value = 0.81). Almost all *C9orf72* positive cases (7; 87.5%) were assigned to cluster one (Table 1), with no significant difference in the proportion of these cases between clusters (X^2^ = 4.24, p-value = 0.12).

Four known ALS-associated genes (*HSPB1, CAV1, CX3CR1, RNASE2*) were among the informative genes selected for the cluster signatures, with all four demonstrating significant differences in their average expression values between clusters when performing one-way ANCOVA corrected for sex assigned at birth, age at death and post-mortem delay (Supplementary Fig. 3). When performing post-hoc analysis to assess which clusters show differential expression, only *CX3CR1*, which was assigned to cluster three, was significantly upregulated compared to cluster one (Tukey p-value = 1.2E-05) and cluster two (Tukey p-value = 7.2E-05) without difference in expression between clusters one and two (Supplementary Fig. 3C). The other gene’s cluster assignments did not have a complete influence on their expression in cases assigned to the gene’s cluster, although for the cluster three informative gene RNASE2, there was a trend for higher expression in cases assigned to that cluster compared to the others (Supplementary Fig. 3D). The full statistical results are available in Supplementary Fig. 3E.

**Table 1:** Demographics and omics-based/clinical phenotypes for the samples assigned to each cluster for each dataset

	KCL BrainBank (motor cortex)			TargetALS (motor cortex)			Zucca (blood)			van Rheenen (blood)		
	1	2	3	1	2	3	1	2	3	1	2	3
Number of Samples (%)	60 (53.57)	28 (25.00)	24 (21.43)	97 (57.7)	28 (16.6)	43 (25.6)	13 (86.70)	1 (6.65)	1 (6.65)	335 (84.4)	33 (8.31)	29 (7.31)
Number of Samples with a posterior probability ≥ 80% (%)	NA	NA	NA	88 (90.7)	22 (78.6)	31 (72.1)	9 (69.2)	1 (100)	0 (0)	275 (82.1)	31 (93.9)	11 (37.9)
N Males: N Females (Ratio)	35:25 (1.4)	15:13 (1.15)	15:9 (1.67)	60:37 (1.62)	18:10 (1.80)	21:22 (0.95)	6:7 (0.86)	0:1 (0)	1:0 (0)	205:130 (1.58)	18:15 (1.20)	16:13 (1.23)
C9 positive individuals (N)	7	1	0	11	0	4	NA	NA	NA	NA	NA	NA
Age at Symptom Onset in Years (mean ± SD)	58.8 ± 11.6	65.7 ± 12.3	61.7 ± 15.7	59.6 ± 11.1	64.9 ± 9.52	60.3 ± 11.5	63.6 ± 8.6	67.0 ± 0.0	65.0 ± 0.0	62.7 ± 11.9	57.9 ± 12.0	60.9 ± 12.3
Age at Blood Draw in Years (mean ± SD)	NA	NA	NA	NA	NA	NA	66.1 ± 9.8	69.0 ± 0.0	68.0 ± 0.0	NA	NA	NA
Age At Death in Years (mean ± SD)	62.5 ± 11.4	70.2 ± 11.4	64.2 ± 15.6	63.2 ± 10.2	69.5 ± 9.0	64.5 ± 8.9	NA	NA	NA	NA	NA	NA
Limb Onset (N)	36	10	17	65	22	17	NA	NA	NA	215	21	15
Bulbar Onset (N)	15	7	5	14	5	21	NA	NA	NA	120	12	14
Limb + Bulbar Onset (N)	1	1	0	7	0	1	NA	NA	NA	NA	NA	NA
Diagnostic Delay in Years (mean ± SD)	0.0015 ± 0.0013	0.00047 ± 0.00085	0.001 ± 0.0012	0.025 ± 0.32	0.073 ± 0.59	0.12 ± 0.35	NA	NA	NA	NA	NA	NA
Disease Duration in Years (median (IQR))	3.16 (1.96)	2.30 (1.81)	2.38 (1.75)	3.00 (2.13)	4.00 (3.48)	2.00 (2.00)	NA	NA	NA	2.41 (2.02)	2.47 (1.14)	2.37 (1.53)
Post-mortem Delay in Hours (mean ± SD)	26.1 ± 12.10	26.0 ± 10.70	25.9 ± 13.90	9.9 ± 6.10	10.0 ± 7.45	12.0 ± 8.26	NA	NA	NA	NA	NA	NA
Mitochondrial DNA Copy Number (mean ± SD)	465 ± 22.0	457 ± 22.4	459 ± 17.3	NA	NA	NA	NA	NA	NA	NA	NA	NA
Telomere Length in Kilobytes (mean ± SD)	4.04 ± 0.46	3.98 ± 0.56	3.77 ± 0.42	NA	NA	NA	NA	NA	NA	NA	NA	NA
Transcriptional Age Acceleration in Years (mean ± SD)	6.16 ± 9.24	0.45 ± 10.90	5.59 ± 10.80	10.50 ± 8.63	4.19 ± 8.08	8.54 ± 8.44	-23.50 ± 9.90	-28.60 ± 0.00	-26.93 ± 0.00	-41.21 ± 11.66	-36.68 ± 11.84	-38.62 ± 11.69
Biological Age Acceleration in Years (mean ± SD)	5.99 ± 2.92	4.06 ± 4.65	7.93 ± 4.67	NA	NA	NA	NA	NA	NA	NA	NA	NA

NA represents values that could not be collected due to omics and clinical data availability, SD = standard deviation, N = number of samples or individuals, IQR = interquartile range

### Each cluster represents a molecularly distinct phenotype that is linked to ALS pathogenesis

**Fig. 2:**
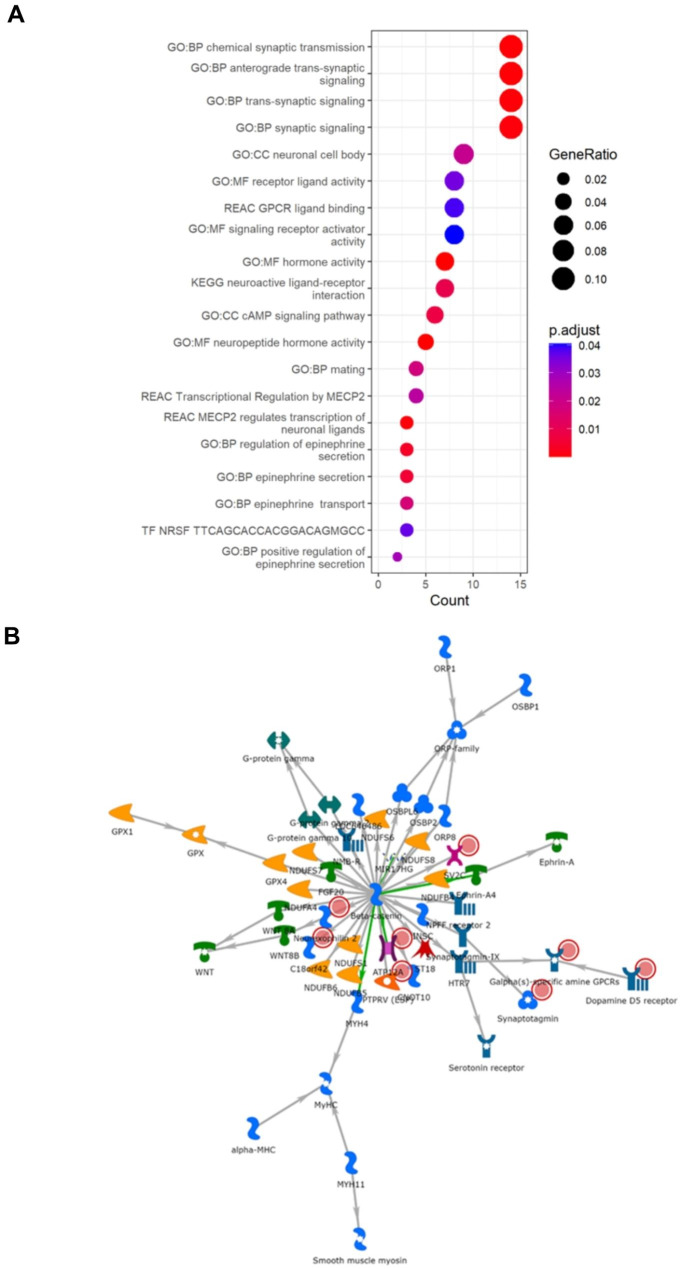
Results of gene enrichment and network analyses for Cluster 1. (**A**) GProfiler2 reveals enrichment for various synaptic and neuropeptide signalling related processes. (**B**) The most significant sub-cluster reveals a mitochondrial-specific and neuronal signalling network. Red circles present in each network represent informative genes identified in each cluster. The descriptions of what each symbol represents is available in Supplementary Fig. 4

Characterising the molecular architectures of each cluster by using gene enrichment and gene network analyses, we found that each cluster represents a distinct molecular phenotype. Cluster one was significantly enriched for various neuronal and synaptic signalling-related processes such as neuropeptide activity, cAMP signalling, and neuroactive ligand transcription, binding, and receptor interaction (Fig. 2A, Supplementary Table 4). Network analysis revealed that a mitochondria specific signalling network is also present (Fig. 2B, p-value = 1.05E-20). Led by *NXPH2*, *ATP12A*, *PTPRV*, *SV2C* and *C18orf42*, this network is enriched for mitochondrial ATP synthesis coupled electron transport and the aerobic electron transport chain.

**Fig. 3:**
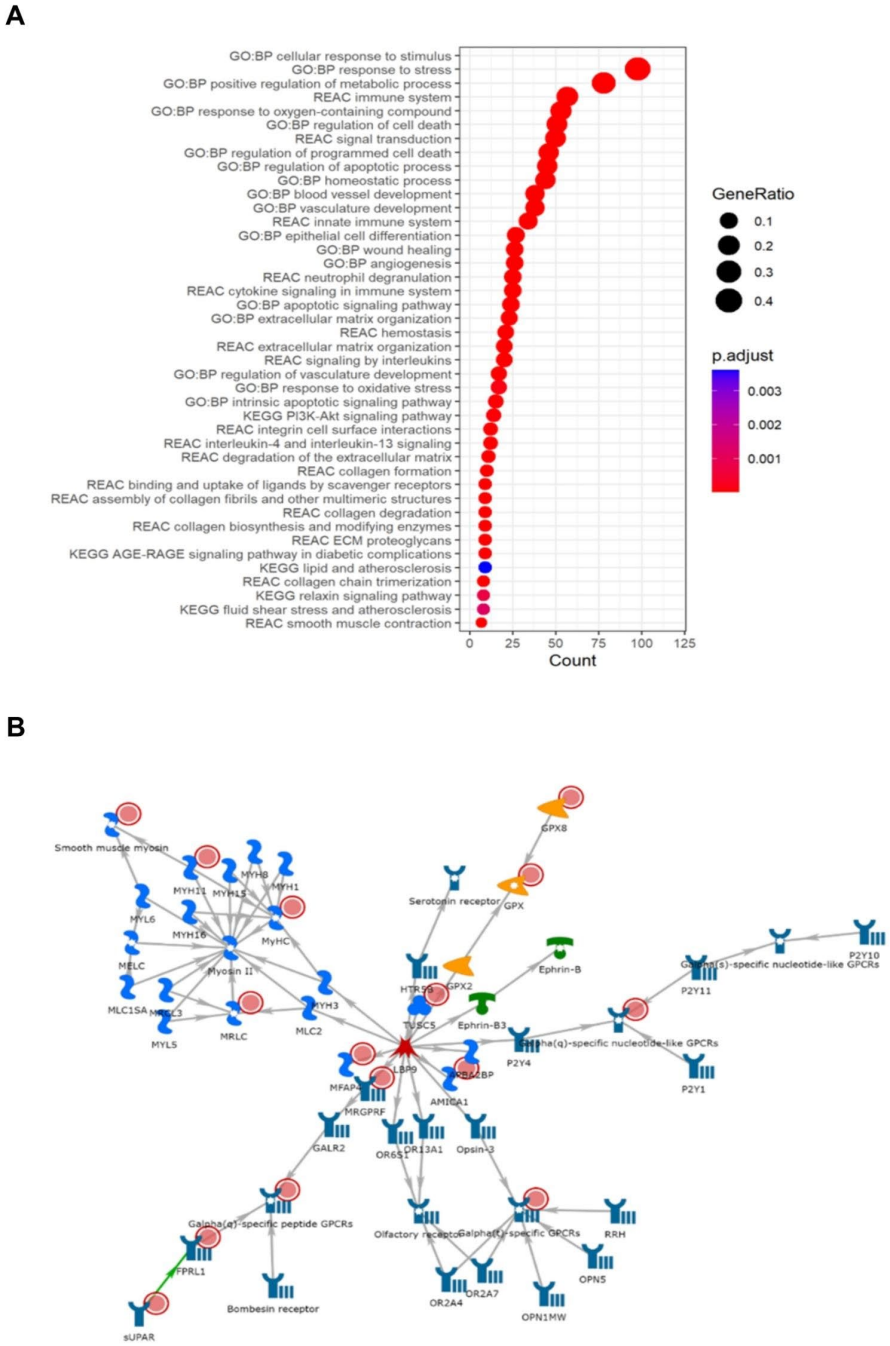
Results of gene enrichment and network analyses for Cluster 2. (**A**) GProfiler2 reveals enrichment for oxidative stress, apoptosis, anti-inflammatory and muscle system-related processes. (**B**) The most significant sub-cluster strengthens the support for muscle contraction processes defining the core of the cluster. Red circles present in each network represent informative genes identified in each cluster. The descriptions of what each symbol represents is available in Supplementary Fig. 4

**Fig. 4:**
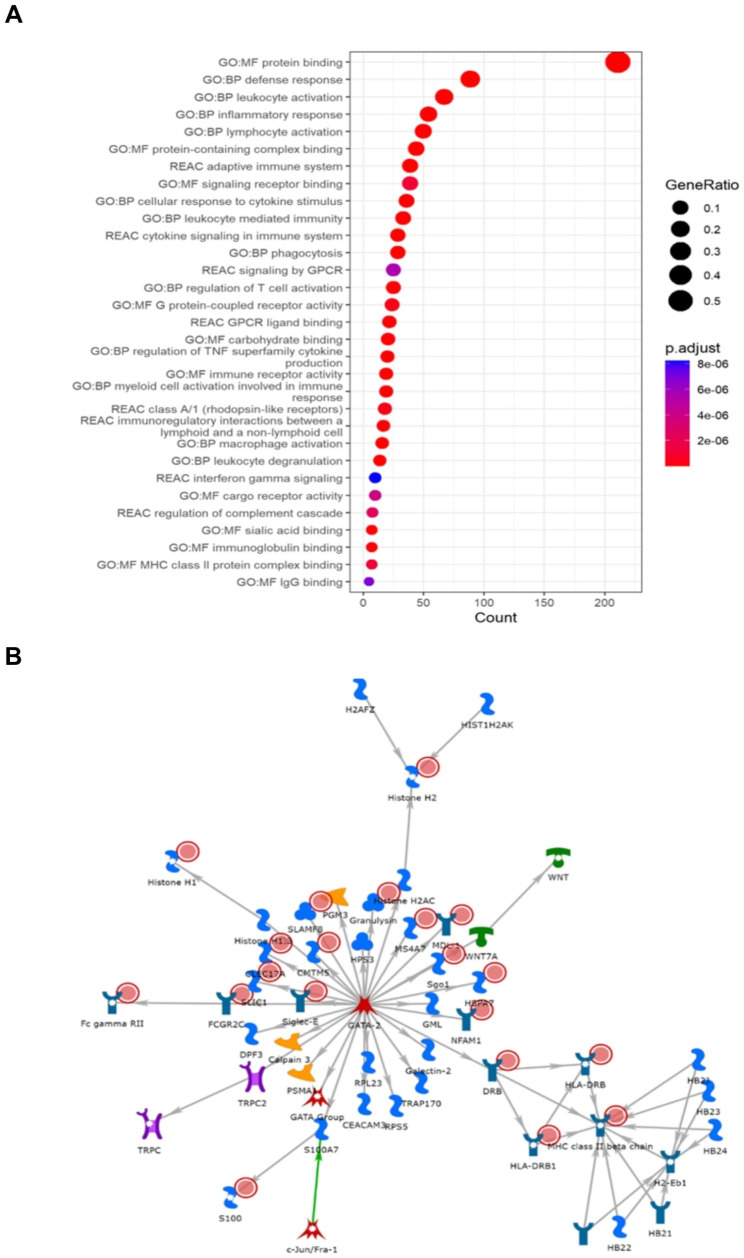
Results of gene enrichment and network analyses for Cluster 3. (**A**) GProfiler2 reveals enrichment for pro-inflammatory processes. (**B**) The most significant sub-cluster reinforces the link to inflammation with the identification of an MHC Class 2 specific network. Red circles present in each network represent informative genes identified in each cluster. The descriptions of what each symbol represents is available in Supplementary Fig. 4

Cluster two was strongly linked with oxidative stress, apoptotic signalling, and vasculature related processes including angiogenesis, blood vessel development, epithelial cell differentiation and atherosclerosis (Fig. 3A). Moreover, muscle-system and extracellular-matrix (ECM) specific enrichments (e.g., collagen synthesis and degradation, smooth muscle contraction, ECM proteoglycans and degradation), and anti-inflammatory pathways (interleukin-4 and interleukin-13 signalling, neutrophil degranulation) from Reactome were also associated with this cluster (Fig. 3A). The muscle contraction theme was strengthened with GO:CC enrichments for banded collagen fibril, supramolecular fiber, myofibril, Z disc, I band, sarcomere, and the actin cytoskeleton (Supplementary Table 5). Cluster two was also enriched for the ALS-gene related NOS3-CAV1 CORUM complex (p-value = 0.018). Furthermore, the cluster two network (Fig. 3B, p-value = 1.09E-17), which was driven by *MFAP4*, *FPRL1*, *TUSC5*, *MRGPRF*, and *PLAUR*, was associated with muscle contraction and actin-myosin filament sliding as well as phospholipase C-activating G protein coupled signalling. Cluster three represents an inflammatory phenotype, with biological process enrichment strongly associated with immune response in GO:BP and KEGG (Supplementary Table 6), as well as links with adaptive immunity, complement cascade, and interferon gamma signalling in Reactome and immunoglobulin activity and major histocompatibility complex (MHC) class II in GO:MF (Fig. 4A). Furthermore, C1q and TLR1-TLR2 CORUM complexes and viral diseases present in KEGG, such as Epstein-Barr disease, herpes simplex virus 1, and influenza A were among the most significant enrichments. Nine microRNAs were also significantly enriched in cluster two (including hsa-miR-335-5p, hsa-miR-146a-5p, hsa-mIR-124-3p, hsa-miR-29a-3p, and hsa-miR-204-5p), with hsa-miR-335-5p also being enriched in cluster three (Supplementary Tables 5 and 6). The cluster three network (Fig. 4B, p-value = 1.47E-26), defined by *GNLY*, *HSPA7*, *SLAMF8*, *CLEC17A*, and *Sgo1*, is MHC-class II specific and enriched for antigen processing, peptide antigen assembly, and presentation of peptides and polysaccharide antigens. Furthermore, the centre of the network, *GATA-2*, was the most significantly enriched TRANSFAC element in cluster three (*GATAD2A*, p-value = 9.56E-17, Supplementary Table 6).

### The molecular phenotypes are robust and validated in Independent brain and blood datasets

**Fig. 5:**
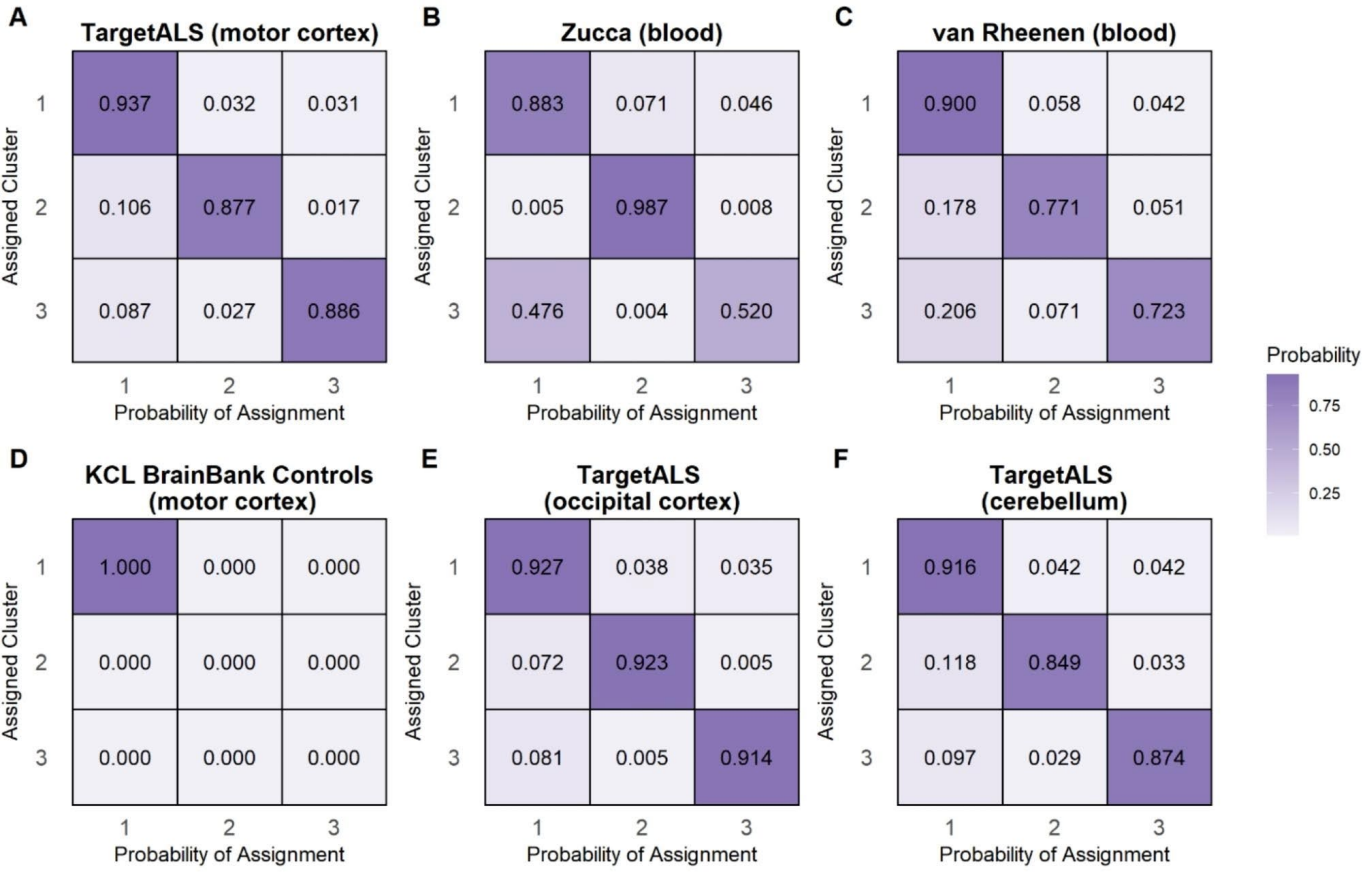
Posterior probabilities of cluster assignment for the six independent expression datasets using linear discriminant analysis trained on the shared informative genes between each dataset and KCL BrainBank. The x-axis represents the average predicted posterior probability of being assigned to one of the three clusters, with the diagonals of the y-axis representing the average posterior probability of being assigned to the correct cluster

To validate the KCL BrainBank derived clusters, we performed linear discriminant-driven cluster assignments of the TargetALS, Zucca and van Rheenen samples, using the intersection between the genes expressed in each one of them and the 794 genes that were used to define the clusters in the KCL BrainBank. Samples from each dataset were assigned to one of the three clusters with high certainty (between 80 and 90%) based on average posterior probability (diagonal cells in Fig. 5A, B and C). A breakdown of the sample to cluster composition for all case datasets is available in Table 1. For the Zucca dataset, the posterior probability of belonging to cluster three is marginally higher than cluster two as only one sample was assigned to it.

To determine whether the molecular phenotypes also withheld validity in control datasets, we applied the same approach to healthy controls from the KCL BrainBank as well as TargetALS case datasets of the occipital cortex and cerebellum (demographics available in Supplementary Table 1). We found that all KCL BrainBank controls were assigned to cluster one (Fig. 5D), whereas for the TargetALS datasets, cluster accuracy was not degraded as there were similar average probability estimates for cluster assignment as in the TargetALS motor cortex dataset (diagonal cells in Fig. 5E and F). A visual inspection of the sample assignments based on the calculated linear discriminants available in Supplementary Fig. 5 (case datasets) and Supplementary Fig. 6 (control datasets). The posterior probability of assignment to each of the three clusters for each sample in the case datasets is available in Supplementary Table 7. Bootstrapping to assess cluster assignment stability for each of the six datasets revealed that all TargetALS datasets and the Zucca dataset had a 100% median assignment accuracy (Table 2), confirming that these cluster assignments are robust. The van Rheenen dataset and KCL BrainBank controls had a variable assignment accuracy, therefore their cluster stability was deemed to be relatively unstable.

We also performed a reverse validation of the TargetALS motor cortex case dataset by performing hierarchical clustering in the replication dataset (TargetALS) as initially performed on KCL BrainBank (the discovery dataset). The purpose of this was to assess whether unsupervised clustering in both datasets leads to similar clustering assignments. Three clusters were defined by a total of 238 informative genes, with 47, 42 and 79 samples assigned to three clusters when TargetALS was utilised for the hierarchical clustering. We found that high proportions of samples assigned to these clusters (81.4%, 67% and 71.4% respectively) were the same as when the KCL BrainBank was utilised for clustering.

**Table 2:** Bootstrapping results for the linear discriminant analysis-derived sample assignments for the six independent datasets. Bootstrapping was performed with 1000 iterations, with average accuracy of correct class assignment used as the evaluation metric. Average accuracy and silhouette values are reported as median and 95% confidence intervals

Dataset	Number of Genes	Median Accuracy (95% CI)	Median Silhouette (95% CI)
TargetALS	470	1.000 (1.000–1.000)	0.137 (0.109–0.168)
Zucca	381	1.000 (1.000–1.000)	0.127 (0.0560–0.234)
van Rheenen	535	0.738 (0.693, 0.778)	0.0185 (-0.0145-0.0512)
BrainBank Controls	787	0.661 (0.543–0.780)	0.220 (0.167–0.281)
TargetALS (occipital cortex)	651	1.000 (1.000–1.000)	0.199 (0.132–0.283)
TargetALS (cerebellum)	622	1.000 (1.000–1.000)	0.174 (0.139–0.217)

### The molecular phenotypes are not present in controls and represent specific features of motor cortex gene expression

**Fig. 6:**
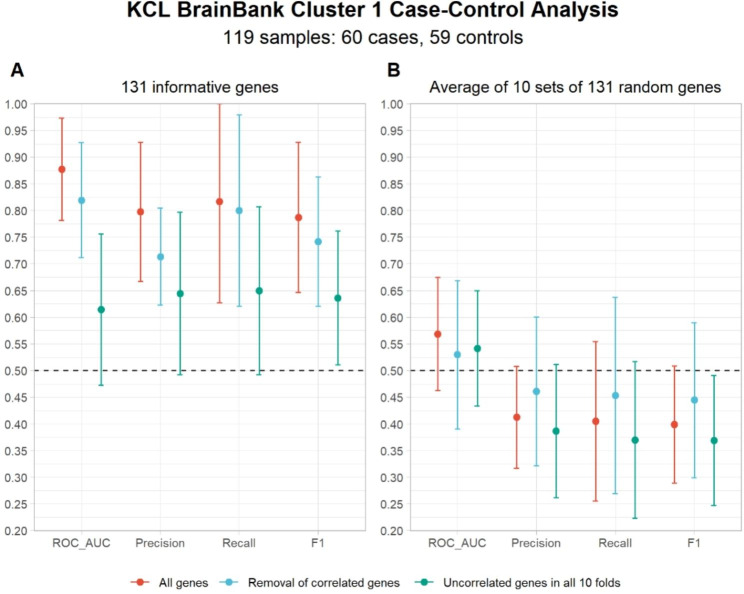
Average performance metrics over all 10 cross-validation folds under three scenarios for KCL BrainBank Cluster 1 case-control analysis. The performance of the classifier supplied with all 131 informative genes in cluster 1 (**A**) was compared to the average performance when supplying ten classifiers with a random set of 131 genes from the full expression matrix (**B**). The x-axis represents each metric used to assess the discriminative performance of each model, with the average score represented on the y-axis. Each point represents the mean and standard deviation. The dotted line at 0.5 represents the performance value you would expect by chance

As all KCL BrainBank controls were assigned to cluster one, and the model is constrained to assign each sample to at least one class, we sought to see if there were differences in the expression of informative genes between cases and controls belonging to cluster one. We found that 87 genes (66.4%) were differentially expressed in cases (Supplementary Fig. 7, Supplementary Table 8), which supports that this gene-specific expression profile is altered in ALS. We then constructed a logistic regression classification model with ten-fold cross-validation to determine if this expression profile is altered in such a way that it can accurately discriminate between ALS and control status (Fig. 6A). We supplied the model with three different cluster one gene signature scenarios (one: all 131 genes in the signature, two: removing multicollinear genes from each fold, three: genes common to all folds after removing multicollinearity). We found that training the models under scenarios one and two achieved excellent discriminative ability (one: ROC AUC 0.88 ± 0.10, two: ROC AUC 0.82 ± 0.11), thus supporting the ALS-specificity of the clusters and expression profiles. Notably, scenario one also achieved the highest performance based on all metrics (precision = 0.80 ± 0.13, recall = 0.82 ± 0.19, and F1 = 0.79 ± 0.14). Conversely, the discriminative power under scenario three was poor (ROC AUC 0.61 ± 0.14). The specificity of the cluster one expression signature for ALS was further supported with the finding that the average performance over ten rounds of supplying the classifier with 131 randomly sampled genes for ROC AUC and the other metrics are in line with and below what would be expected by chance, regardless of scenario (Fig. 6B).

We then assessed whether these molecular phenotypes are truly representative of a motor-cortex based disease process by adopting the same approach as with KCL BrainBank but for distinguishing between samples from the motor cortex and other brain regions of cases from TargetALS. For each cluster and scenario, motor cortex-occipital cortex and motor cortex-cerebellum classifiers were constructed. We found that each molecular phenotype did indeed reflect features of motor cortex gene expression as there was perfect discrimination between motor cortex and the other brain regions when supplying all of the cluster-specific informative genes to the model. The overall performance metrics for all case-control, motor cortex-occipital cortex and motor cortex-cerebellum classifiers are available in Supplementary Table 9.

### Cell composition analysis of the ALS motor cortex reinforces the biological interpretation of each molecular subtype

**Fig. 7:**
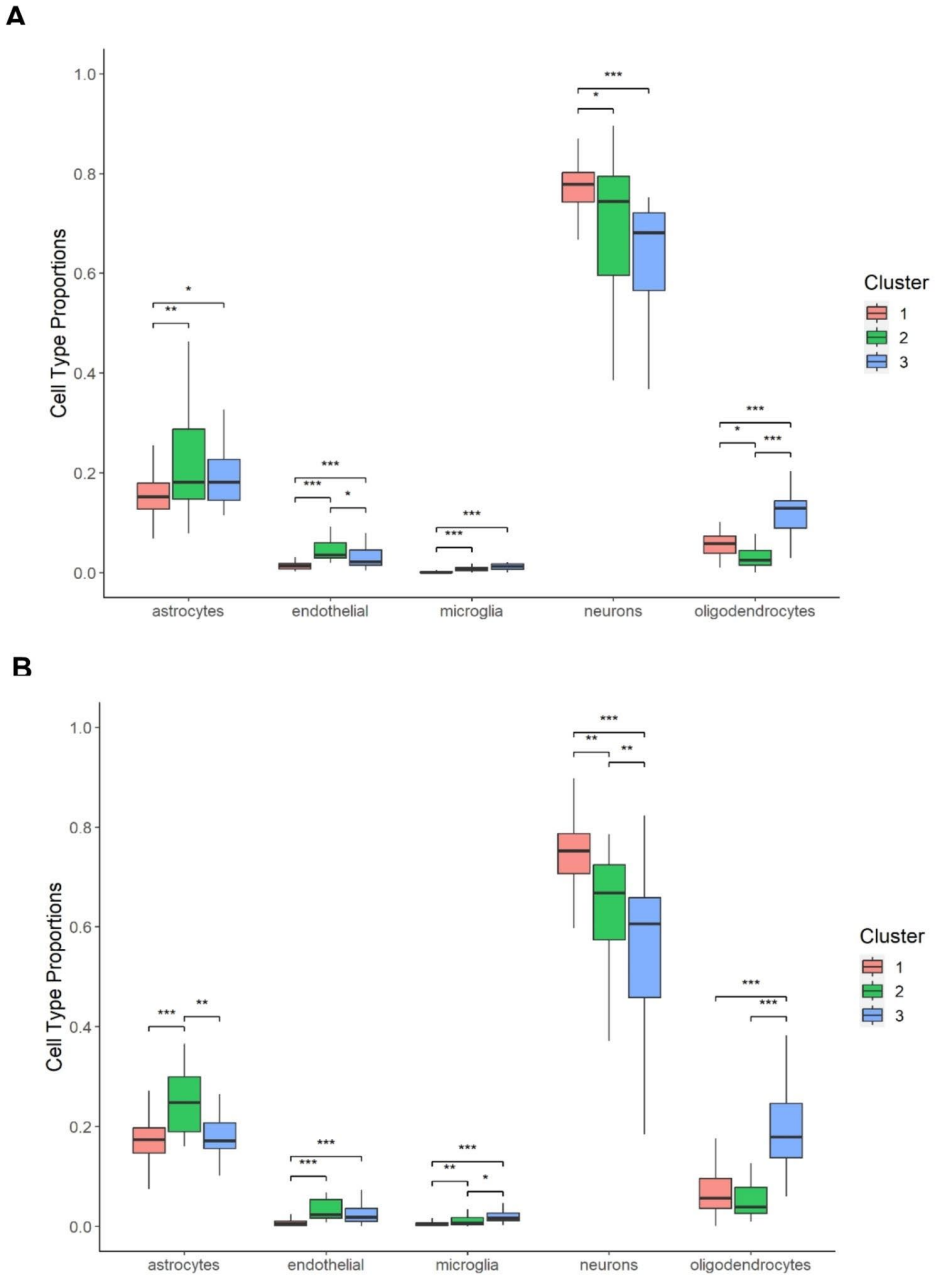
Results of the cell-type deconvolution analysis for (**A**) KCL BrainBank cases and (**B**) TargetALS cases. Results were corrected for age of death and sex assigned at birth. Asterisks refer to one-way ANCOVA post-hoc Tukey p-values: * < 0.05, ** < 0.01, *** < 0.001

When performing cell deconvolution analysis for the KCL BrainBank and TargetALS case datasets, we found that samples that were assigned to each cluster had distinctive cell-type profiles, which were reflective of the predominant biological processes of each molecular phenotype. These profiles were almost identical in both datasets (Fig. 7), with significant overall differences in the proportion of all five cell types. Samples residing in cluster one had a significantly higher proportion of neurons compared to clusters two and three. A higher proportion of astrocytes and endothelial cells were present in samples assigned to cluster two than in cluster one, whilst samples residing in cluster three displaying higher proportions of microglia than cluster one, and oligodendrocytes than clusters one and two. The full statistical results are available in Supplementary Tables 10 and 11.

### Clusters present different clinical outcomes and omics measures

**Fig. 8:**
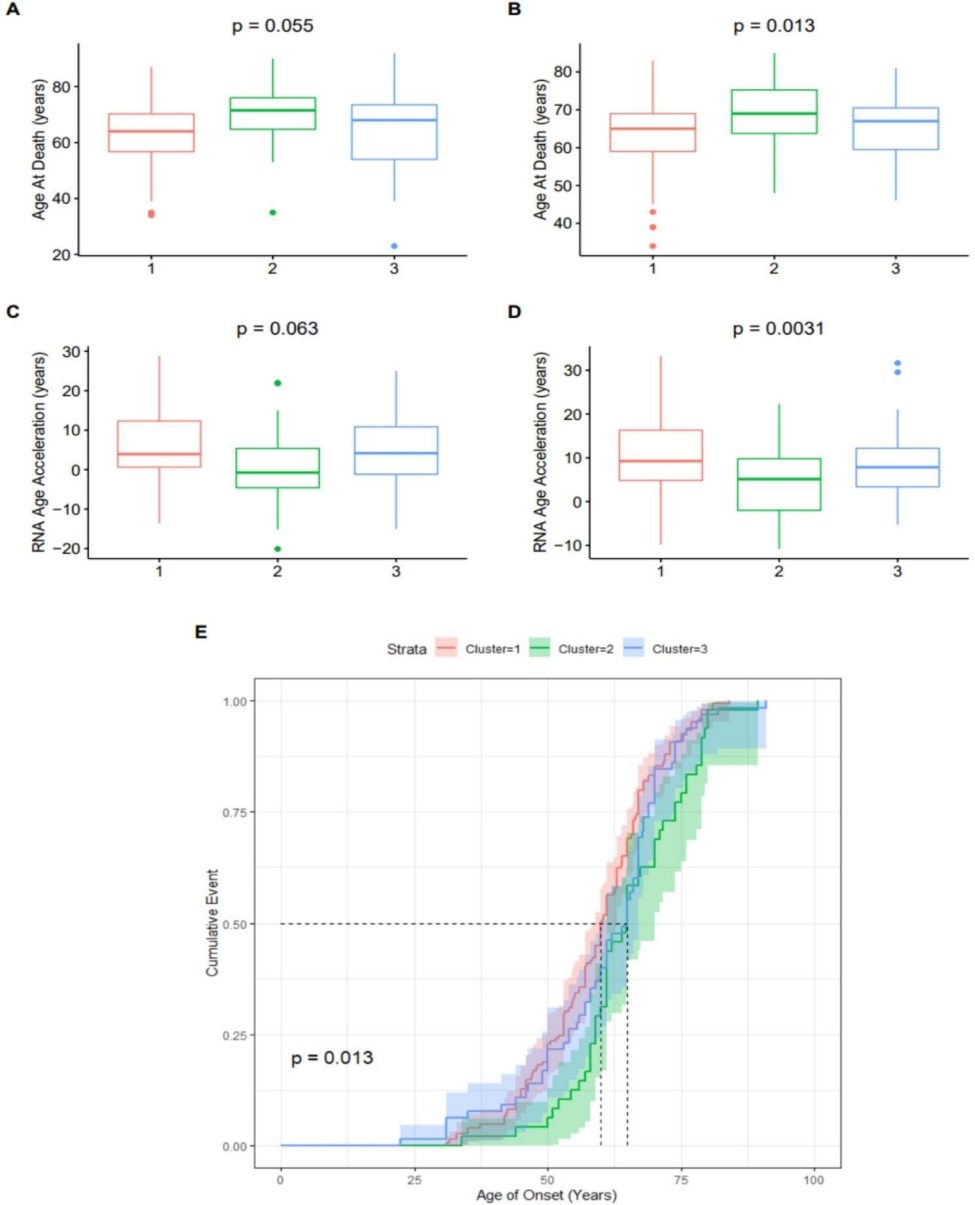
Subgroup phenotype analysis between samples residing in each cluster for KCL BrainBank and TargetALS. Variables visualised here include the age of death for (**A**) KCL BrainBank and (**B**) TargetALS, and transcriptional age acceleration for (**C**) KCL BrainBank and (**D**) TargetALS. P-values are from performing one-way ANCOVA, corrected for sex. (**E**) Cox proportional hazards model for the age of onset of samples from both BrainBank and TargetALS datasets, showing that samples from Cluster 1 have a significantly lower age of onset than Clusters 2 and 3

In both KCL BrainBank and TargetALS, we observed that cluster two demonstrated differences in several phenotypic and omics measures (full results available in Table 3). For instance, cluster two compared to cluster one had a higher age of death (Fig. 8A and B) and smaller transcriptional age acceleration (Fig. 8 C and D). This trend continues when looking at variables present in one of the two datasets, with a 3.87 year slower biological age acceleration being observed in cluster two compared to cluster three in KCL BrainBank (p = 0.020), and a longer but albeit non-significant increase in disease duration in TargetALS samples assigned to cluster two. We also found trends for higher mitochondrial DNA copy number in cluster one, and shorter telomere length in cluster 3 in KCL BrainBank samples (Table 3). When assessing differences in age of onset based on samples combined from KCL BrainBank and TargetALS, we found that samples residing in cluster one had a lower age of onset compared to clusters two and three (Fig. 8E; p = 0.013). For the Zucca and van Rheenen datasets, there was no significant alteration in age of onset and transcriptional age acceleration between clusters.

When assessing potential differences in the site of onset between clusters, we found that in KCL BrainBank, there was a borderline significant difference in the proportion of people with limb-onset SALS assigned to the clusters (X^2^ = 6.05, p-value = 0.05). Bulbar-onset and combined limb and bulbar onset SALS were not overrepresented in any of the clusters (bulbar: X^2^ = 0.18, p-value = 0.91; limb and bulbar: X^2^ = 0.95, p-value = 0.62). As found with the KCL BrainBank dataset, the proportion of limb-onset TargetALS cases differed significantly between clusters (X^2^ = 13.49, p-value = 1.2E-03). The distribution of bulbar-onset cases also varied significantly (X^2^ = 20.10, p-value = 4.3E-05). There was no difference in the proportion of *C9orf72*-positive (X^2^ = 3.45, p-value = 0.18) or combined limb and bulbar onset cases (X^2^ = 3.25, p-value = 0.20). In the van Rheenen dataset, there was no association between limb: bulbar ratio and cluster assignment (X^2^ = 1.78, p-value = 0.41). Across the three case-only datasets, there was no difference in the male:female ratio (TargetALS: X^2^ = 2.48, p-value = 0.29; Zucca: X^2^ = 2.02, p-value = 0.36; van Rheenen: X^2^ = 0.88, p-value = 0.64).

**Table 3:** Statistical results of clinical and omics-based phenotype analysis. Variables that demonstrated non-normality via Shapiro Wilk were log transformed before running one-way ANCOVA (corrected for sex) and post-hoc Tukey’s to assess cluster-specific trends

KCL BrainBank (motor cortex)			
*Phenotype*	*Normality (Shapiro-Wilk W, p-value)*	*One-Way ANCOVA (F-statistic, p-value)*	*Post-Hoc Analysis (Tukey p-value)*
Age at Onset	0.983, 0.22	2.160, 0.121	1 vs. 2; 0.107, 1 vs. 3; 0.622, 2 vs. 3; 0.569
Age at Death	0.976, **0.042**	2.979, 0.055	1 vs. 2; 0.051, 1 vs. 3; 0.988, 2 vs. 3; 0.168
Disease Duration (years)	0.943; **3.5E-04**	4.211; **0.018**	1 vs. 2; **0.036**, 1 vs. 3; 0.092, 2 vs. 3; 0.890
Post-mortem Delay	0.951, **4.4E-04**	0.178, 0.837	1 vs. 2; 0.997, 1 vs. 3; 0.851, 2 vs. 3; 0.855
mtDNA Coverage	0.944, **3.2E-04**	1.886, 0.157	1 vs. 2; 0.988, 1 vs. 3; 0.145, 2 vs. 3; 0.294
mtDNA Copy Number	0.966; **9.9E-03**	1.643, 0.199	1 vs. 2; 0.231, 1 vs. 3; 0.458, 2 vs. 3; 0.945
Telomere Length	0.972, **0.028**	2.451, 0.092	1 vs. 2; 0.810, 1 vs. 3; 0.074, 2 vs. 3; 0.350
Biological Age Acceleration	0.971, **0.025**	3.858, **0.025**	1 vs. 2; 0.110, 1 vs. 3; 0.414, 2 vs. 3; **0.020**
RNA Age Acceleration	0.981, 0.142	2.847, 0.063	1 vs. 2; 0.055, 1 vs. 3; 0.973, 2 vs. 3; 0.203
**TargetALS (motor cortex)**			
***Phenotype***	***Normality (Shapiro-Wilk W, p-value)***	***One-Way ANCOVA (F-statistic, p-value)***	***Post-Hoc Analysis (Tukey p-value)***
Age at Onset	0.977, **7.1E-03**	2.463, 0.088	1 vs. 2; 0.075, 1 vs. 3; 0.968, 2 vs. 3; 0.194
Age at Death	0.984, 0.053	4.456, **0.013**	1 vs. 2; **0.009**, 1 vs. 3; 0.765, 2 vs. 3; 0.089
Diagnostic Delay	0.776, **2.9E-14**	0.926, 0.398	1 vs. 2; 0.840, 1 vs. 3; 0.373, 2 vs. 3; 0.867
Disease Duration (years)	0.705, **2.2E-16**	2.403, 0.094	1 vs. 2; 0.114, 1 vs. 3; 0.944, 2 vs. 3; 0.110
Post-mortem Delay	0.883, **6.8E-10**	1.176, 0.311	1 vs. 2; 0.892, 1 vs. 3; 0.405, 2 vs. 3; 0.349
RNA Age Acceleration	0.989, 0.292	6.004, **3.1E-03**	1 vs. 2; **0.002**, 1 vs. 3; 0.420, 2 vs. 3; 0.092
**Zucca (blood)**			
***Phenotype***	***Normality (Shapiro-Wilk W, p-value)***	***One-Way ANCOVA (F-statistic, p-value)***	***Post-Hoc Analysis (Tukey p-value)***
Age at Onset	0.926, 0.242	0.078, 0.926	1 vs. 2; 0.926, 1 vs. 3; 0.987, 2 vs. 3; 0.986
RNA Age Acceleration	0.990, 0.999	0.178, 0.839	1 vs. 2; 0.868, 1 vs. 3; 0.936, 2 vs. 3; 0.992
**van Rheenen (blood)**			
***Phenotype***	***Normality (Shapiro-Wilk W, p-value)***	***One-Way ANCOVA (F-statistic, p-value)***	***Post-Hoc Analysis (Tukey p-value)***
Age at Onset	0.975, **2.0E-06**	2.282, 0.103	1 vs. 2; 0.100, 1 vs. 3; 0.738, 2 vs. 3; 0.634
Disease Duration (years)	0.815; **< 2.2E-16**	0.00950, 0.991	1 vs. 2; 0.990, 1 vs. 3; 1.000, 2 vs. 3; 0.992
RNA Age Acceleration	0.973, **9.1E-07**	2.788, 0.063	1 vs. 2; 0.082, 1 vs. 3; 0.479, 2 vs. 3; 0.787

P-values < 0.05 are in bold

## Discussion

In this study, we used KCL BrainBank motor cortex gene expression data and machine learning to identify expression signatures which constitute three biologically homogeneous subgroups of SALS: synaptic and neuropeptide signalling (cluster one), oxidative stress and apoptosis (cluster two), and neuroinflammation (cluster three). These molecular phenotypes reflect three previously hypothesised key mechanisms of ALS pathogenesis, which have been recently identified using a deep learning-based approach using expression data from human iPSC-derived *C9orf72*, *TARDBP*, *SOD1* and *FUS* mutant motor neurons [39]. The biological interpretation of each cluster is further reinforced by the fact that in KCL BrainBank and TargetALS case datasets, significantly higher proportions of neurons, endothelial cells, and microglia contribute to clusters one, two and three, respectively.

Genes which constitute the three main subgroups of cortical inhibitory GABAergic interneurons (*PVALB, SST, VIP*) were identified in cluster one [40] This is interesting given that alterations in their excitability patterns cause global hyperexcitability of corticospinal neurons [41], which has long been hypothesised as a trigger for the spread of ALS pathology [42, 43]. There were also several informative genes related to body mass index, metabolism, and energy homeostasis (*LINC01844*, *ADCYAP1*, *CRH, CRHBP, CARTPT, VGF)*. These processes are linked with worse survival and progression outcomes in ALS [44–47].

Several oxidative stress, apoptosis and muscle system related enrichments defined cluster two, as well as anti-inflammatory signalling processes. In fact, the signature of this cluster contained several neuroprotective microglial secretory markers (*IL4R, TGFB1I1, TGFBI, CD163*) [48], as well as the *MMP9* metalloproteinase gene, whose knockdown slows disease progression in ALS mutant models [49–51]. With microglia contributing minimally to this cluster, based on cell deconvolution analysis of KCL BrainBank cases, and better clinical and omics-based age outcomes defining the cluster’s phenotypic profile in this dataset, we can postulate that a reversal of pro-inflammatory processes may be occurring in this SALS subpopulation. This is further supported by evidence that knockout of the ALS risk gene *CAV1* [52] in endothelial cells, whose proportion in samples assigned to this cluster was significantly higher, can reduce innate immune system signalling via activation of endothelial nitric oxide synthase (*NOS3*) [53]; a complex of which was observed in our enrichment analysis. Moreover, this cluster was enriched for several potential microRNA biomarkers. The most encouraging in terms of its impact on the molecular phenotype are miR-335-5p and miR-29b-3, as they are downregulated in ALS patients [54]. Additionally, their downregulation in model systems induces reactive oxygen species-mediated excitotoxicity [55], and intrinsic apoptosis mediated motor neuron loss [56]; key processes which defined this cluster.

In cluster three, there was a clear involvement of the major histocompatibility complex class II and the HLA complex (*HLA-DRA, HLA-DMB, HLA-DOA, HLA-DPA1, HLA-DRB1, HLA-DRB5, HLA-DRB6*), M1 or activated microglia (*CD14, CD86, TREM2, TYROBP, TMEM119, TMEM125)* [48], and pro-inflammatory metalloproteinases (*MMP14*), as well as many immune related genes which were identified in other motor cortex and spinal cord SALS expression studies [8, 57, 58]. The tentative ALS-related modifier gene *CX3CR1* [59]), which is thought to protect against proinflammatory processes and microglial-induced neuronal cell loss [60], was also present in this cluster. Several well studied serum and CSF biomarkers of ALS progression were also present, such as *SPP1* [61], the human chitinases *CHI3L1* and *CHI3L2* [62, 63], and complement C3 [64], in addition to prognostic and predictive CSF biomarkers such as *TREM2*, *LILRA2* and *ITGB2* [65].

We also demonstrated that these molecular phenotypes can define distinct subgroups of people with SALS across independent motor cortex (TargetALS) and blood (Zucca et al.; van Rheenen et al.) datasets of European ancestry, by applying separate linear discriminant models trained on the KCL BrainBank case-derived sample assignments and gene intersections. The average probability of being assigned to the cluster that the samples from each dataset were allocated to was very high (between 0.8 and 0.9). Because this model is constrained to assign samples to one class, in order to test the ALS and motor cortex specificity of the clusters, the same approach was carried out in three additional control datasets (KCL BrainBank controls, TargetALS occipital cortex, TargetALS cerebellum). All KCL BrainBank controls were assigned to cluster one, whereas there were similar average probability estimates for the TargetALS datasets from other regions compared to the motor cortex. With the exception of KCL BrainBank controls, the cluster stability estimates were robust, which supports the validity of the cluster assignments. Furthermore, when performing reverse validation by applying hierarchical clustering to the TargetALS motor cortex dataset and comparing the grouping of samples to the linear discriminant analysis derived assignments, we found a 67–81% overlap in sample assignment which demonstrates that the cluster assignments, regardless of gene composition, is consistent. To determine if the expression signatures could distinguish between cases and controls in KCL BrainBank and between motor cortex, cerebellum and occipital cortex, we constructed a logistic regression classifier and found that the signatures had excellent discriminative power, which indicates that this molecular phenotypes are linked to ALS and the motor cortex and shows the diagnostic potential of the expression signatures.

In regard to the sample assignment to different clusters, specifically cluster one, the proportion of samples varied based on tissue type (approximately 60% for the motor cortex versus 85% for the blood datasets). A potential explanation for this is that as the motor cortex represents the end stage of disease, perhaps other biological processes explained by the remaining molecular phenotypes more strongly influence the progression of disease in samples assigned to those clusters. This may not be as apparent in the blood datasets given that the samples were collected at different stages of the disease. This is also plausible as all the KCL BrainBank controls, which are not affected by ALS, are assigned to cluster one.

We also discovered that there were distinct clinical and omics-related outcomes that distinguished each cluster in both motor cortex case datasets. Cluster two was associated with a higher age of death and longer disease duration, accompanied by a decrease in transcriptional age acceleration. There are several plausible explanations as to why this trend was observed; the first is that more people assigned to this cluster may have had a history of Riluzole usage than other clusters, as it modulates apoptosis, autophagy and other excitotoxicity-related processes which are prevalent biological processes in this cluster [66, 67]. Another possibility is that genomic variants present in inflammatory genes assigned to this cluster may diminish their effects. This theory is supported by the example of *IL18RAP*, which is an M1 secretory marker present in this cluster [48], of whom 3’UTR variants were recently found to protect against ALS, by impeding microglial-dependent motor neuron degeneration [68]. In KCL BrainBank cases assigned to cluster two, there was also a significant decrease in biological age acceleration. Whether this phenomenon is also apparent in TargetALS, and the blood datasets could not be analysed because biological age acceleration could only be measured in KCL BrainBank as there was matching epigenetic information available. However, this warrants further investigation in additional datasets as evidence links increased serum levels of the chronic inflammation marker suPAR, encoded by the cluster two gene *PLAUR*, with higher biological age acceleration in the normal population [69]. Therefore, suPAR could be a modulator of prognostic outcomes in SALS patients associated with this molecular phenotype. Telomere length was shorter in cluster three in KCL BrainBank, which despite being non-significant, is also an important trend to investigate as although mounting evidence supports the association between longer telomere length and worsened severity of ALS [70, 71], there is also an established link between chronic inflammatory states and telomere shortening in aging and disease [72–74]. Finally, we found that samples in cluster one had a lower age of onset in a combined analysis of KCL BrainBank and TargetALS, which makes sense given that this cluster is linked to neuronal dysfunction and therefore motor neuron degeneration. The proportion of people with limb-onset ALS assigned to the clusters also differed significantly in both motor cortex datasets. Despite the association between distinct age of onset with each cluster in the motor cortex datasets, this was not replicated in the blood datasets, therefore further examination is needed to establish if clear phenotype differences exist across clusters.

There are several limitations of this study which will require further investigation in the context of our findings. First, only samples belonging to the KCL BrainBank dataset had matching multi-omics data, which meant that cluster-specific effects on omics variables could not be assessed in the other datasets. Likewise, both blood datasets had limited clinical information, which did not allow us to validate all possible clinical phenotype associations. Furthermore, the van Rheenen blood dataset did not replicate the association between age of death and age at onset with class membership. Some potential explanations are that microarray technology was used to obtain the transcriptomic profiles in this dataset, translating in a lower number of genes available that were part of the subtype signatures and lower class assignment accuracy. Indeed, clusters two and three represented approximately 25% of ALS patients each in the brain datasets, while only ~ 8% each in the van Rheenen blood dataset. Moreover, the Dutch population might present a more distinct structure compared to other European countries [75]. Finally, we did not integrate genomic variants into our analysis to further enhance our molecular classification, like recent studies that built upon their previous clustering analyses [9, 76] as this would have resulted in underpowered analyses given our sample sizes, or correlated our clustering findings with neuropathological findings and co-existing pathologies in the motor cortex datasets, as this data was not available to us when the study took place. Future work should attempt to integrate these additional modalities to further enhance the disease relevance of the identified molecular phenotypes. As we did not perform a comparative analysis of the cluster assignment of people with familial ALS or other neurodegenerative diseases i.e. FTD, Parkinson’s disease, we cannot be absolutely sure that the molecular phenotypes identified in this study are truly ALS-specific. Although, we can say that they represent sporadic ALS subtypes as the clusters were validated in three independent ALS datasets which did not contain samples from people with familial ALS. They also represent motor-cortex specific aspects of the disease process as the expression signatures of each molecular phenotype can distinguish samples from the motor cortex from other brain regions for TargetALS cases.

In conclusion, we have demonstrated that people with ALS can be successfully stratified into molecularly and phenotypically distinct subgroups using gene expression data. Our results support the hypothesis that different mechanisms underly distinct forms of ALS pathogenesis and can be identified in patients via specific expression signatures. These molecular phenotypes discovered in a UK cohort, were validated in independent motor cortex and blood datasets and could be used to distinguish patients from controls, showing potential to be used for clinical trial stratification and the development of biomarkers for personalised treatments and diagnostics. Our analysis also revealed several known candidate gene biomarkers which could be exploited to stratify people with SALS in future studies. We have developed a publicly available web app (https://alsgeclustering.er.kcl.ac.uk) to allow the broader scientific and clinical community to use our model for the stratification of pre- and post-mortem samples in their studies.

### Electronic supplementary material

Below is the link to the electronic supplementary material.

Supplementary methods, figures and tables

## Data Availability

The RNAseq (Zucca et al.) and microarray (van Rheenen et al.) blood expression data is publicly available and accessible via the Gene Expression Omnibus website (see Methods: Accession Numbers for details). The KCL BrainBank datasets are available upon reasonable request from the corresponding author. The TargetALS dataset is available upon approval by the TargetALS Postmortem Tissue Core.
