# Supplementary material for: Unsupervised machine learning identifies distinct ALS molecular subtypes in post-mortem motor cortex and blood expression data
Source: Acta Neuropathol Commun. 2023 Dec 21;11:208. doi: 10.1186/s40478-023-01686-8 (PMC10734072; doi:10.1186/s40478-023-01686-8)
Supplement: Supplementary file 1 — Supplementary methods, figures and tables [file 40478_2023_1686_MOESM1_ESM.docx]

**Supplementary materials of Unsupervised machine learning identifies distinct ALS molecular subtypes in post-mortem motor cortex and blood expression data**

Heather Marriott^1,2^, Renata Kabiljo^2^, Guy P Hunt^1,2,4,5^, Ahmad Al Khleifat^1^, Ashley Jones^1^, Claire Troakes^1,3^, Project MinE ALS Sequencing Consortium, TargetALS Sequencing Consortium, Abigail L Pfaff^4,5^, John P Quinn^6^, Sulev Koks^4,5^, Richard J Dobson^2,7,8,9^, Patrick Schwab^10^, Ammar Al-Chalabi^1,11^ and Alfredo Iacoangeli^1,2,7,$^

**Supplementary Information**

**Table of Contents**

Sequencing Details………………………………………………………………………………………….. 2

RNA-Sequencing: MRC London Neurodegenerative Diseases Brain Bank (KCL) ……………... 2

RNA-Sequencing: Target ALS Postmortem Tissue Core ………………………………………... 2

RNA-Sequencing: Peripheral Blood Mononuclear Cells (PBMC) Datasets ……………………... 2

Whole Genome Sequencing and Methylation Microarray Data ………………………………….. 2

Supplementary Figure 1 …………………………………………………………...………………………... 3

Supplementary Figure 2 …………………………………………………………………………………….. 3

Supplementary Figure 3 …………………………………………………………………………………….. 4

Supplementary Figure 4 …………………………………………………………………………………….. 5

Supplementary Figure 5 …………………………………………………………………………………….. 6

Supplementary Figure 6 …………………………………………………………………………………….. 7

Supplementary Figure 7 …………………………………………………………………………………….. 8

Supplementary Table 1 ……………………………………………………………………………………... 9

Supplementary Table 2 ……………………………………………………………………………………... 10

Supplementary Table 3 ……………………………………………………………………………………... 11

Supplementary Table 4 ……………………………………………………………………………………... 29

Supplementary Table 5 ……………………………………………………………………………………... 30

Supplementary Table 6 ……………………………………………………………………………………... 92

Supplementary Table 7 ……………………………………………………………………………………... 146

Supplementary Table 8 ……………………………………………………………………………………... 159

Supplementary Table 9 ……………………………………………………………………………………... 163

Supplementary Table 10 ……………………………………………………………………………………... 164

Supplementary Table 11 ……………………………………………………………………………………... 165

References …………………………………………………………………………………………………... 166

**RNA-Sequencing: MRC London Neurodegenerative Diseases Brain Bank (KCL)**

The 100mg frozen tissue blocks were divided; one for RNA purification and the other for DNA. For each sample, a 30mg tissue block for RNA was homogenised using a Qiagen PowerLyzer 24 Homogenizer. Total RNA was purified from the homogenate using the standard protocol of the RNeasy Lipid Tissue Mini Kit (Qiagen), with on-column DNAse digestion. RNA integrity was estimated using Agilent Bioanalyzer 2100’s RNA 6000 Nano assays. RNA quantification was performed using a NanoDrop. Library preparation was performed using the standard Illumina TruSeq Stranded Total RNA Sample Preparation Guide with Ribo-Zero Human/Mouse/Rat (October 2013 Rev E.). Fragmentation steps were tailored to degrees of degraded RNA samples using Agilent Bioanalyzer 2100’s Nano assay results from the previous section. Libraries were validated using an Agilent Bioanalyzer 2100 to assess fragment size distribution. Library concentrations were estimated using a Qubit RNA High Sensitivity Assay Kit. Nanomolar (nM) concentrations were estimated using nM = ng/ul X (1500/Average bp). Libraries were sequenced using Illumina HiSeq 4000 flow cells with 150bp paired-end reads with a target depth of 30 million clusters (60 million reads per sample).

**RNA-Sequencing: Target ALS Postmortem Tissue Core**

Information for the library preparation and RNA extraction of the TargetALS dataset is available at <http://www.targetals.org/wp-content/uploads/2020/11/README-1.zip>.

**RNA-Sequencing: Peripheral Blood Mononuclear Cells (PBMC) Datasets**

Information for the library preparation and RNA extraction of the Zucca dataset is available at,^1^ and the microarray sequencing and normalisation protocol for the van Rheenen dataset is available at.^2^

**Whole Genome Sequencing and Methylation Microarray Data**

For the KCL dataset, we also included matching whole genome sequencing and methylation microarray data collected under Datafreeze Two of the Project MinE ALS sequencing consortium.^3^ DNA was isolated from venous blood using standard methods. The DNA concentrations were set at 100 ng/uL as measured by a fluorimeter with the PicoGreen® dsDNA (Thermo Scientific, Waltham, MA) quantitation assay. DNA integrity was assessed using gel electrophoresis. The whole genome sequencing protocol is as follows: all samples were sequenced using Illumina’s FastTrack services (Illumina, San Diego, CA) on the Illumina HiSeq 2000 platform. Sequencing was 100 bp paired-end performed using polymerase chain reaction (PCR)-free library preparations and yielded ∼40x coverage across each sample. Binary sequence alignment/map formats (BAM) were generated for each individual. DNA methylation was analysed using Illumina Infinium EPIC array following the standard Infinium HD array methylation protocol (Illumina).


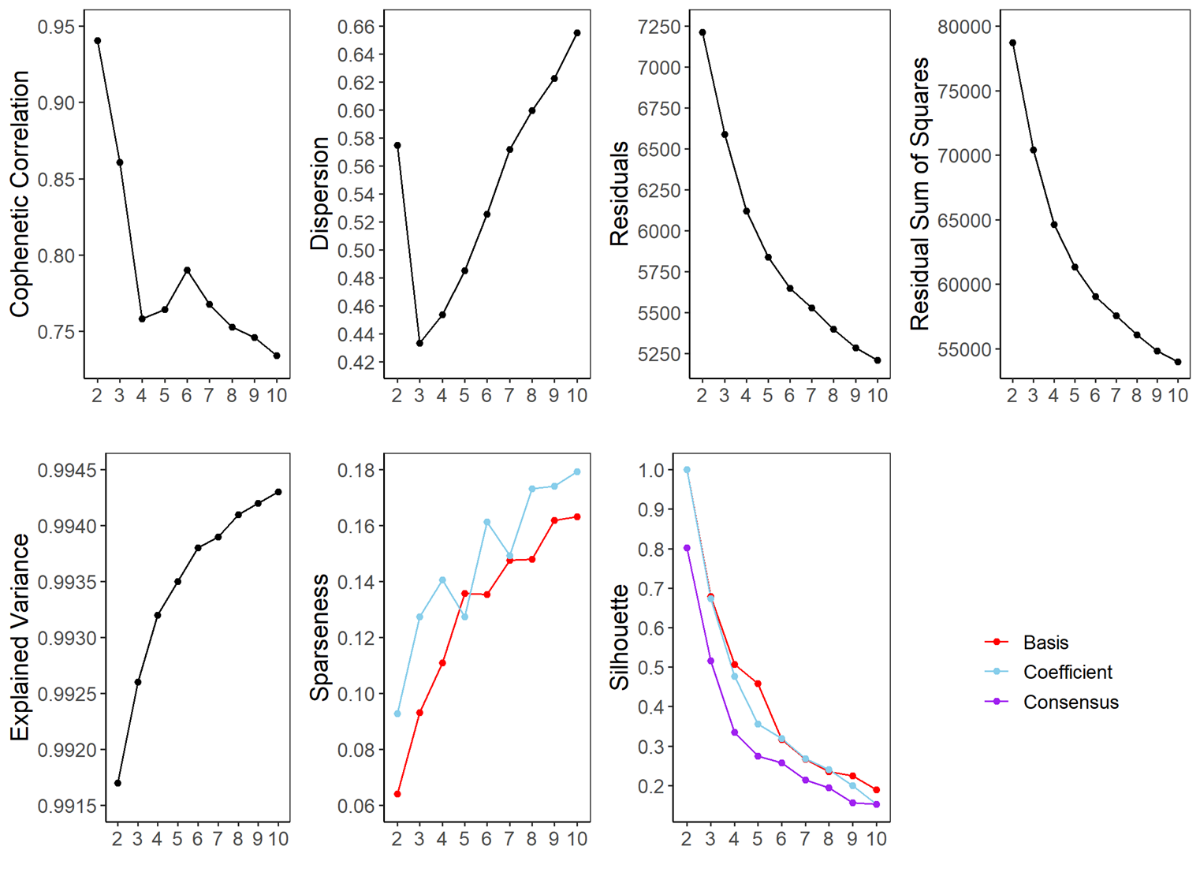
**Supplementary Figures**

**Supplementary Figure 1. Cluster estimation metrics produced when running nsNMF with k = 2-10, 100 runs and 1000 iterations.**

**
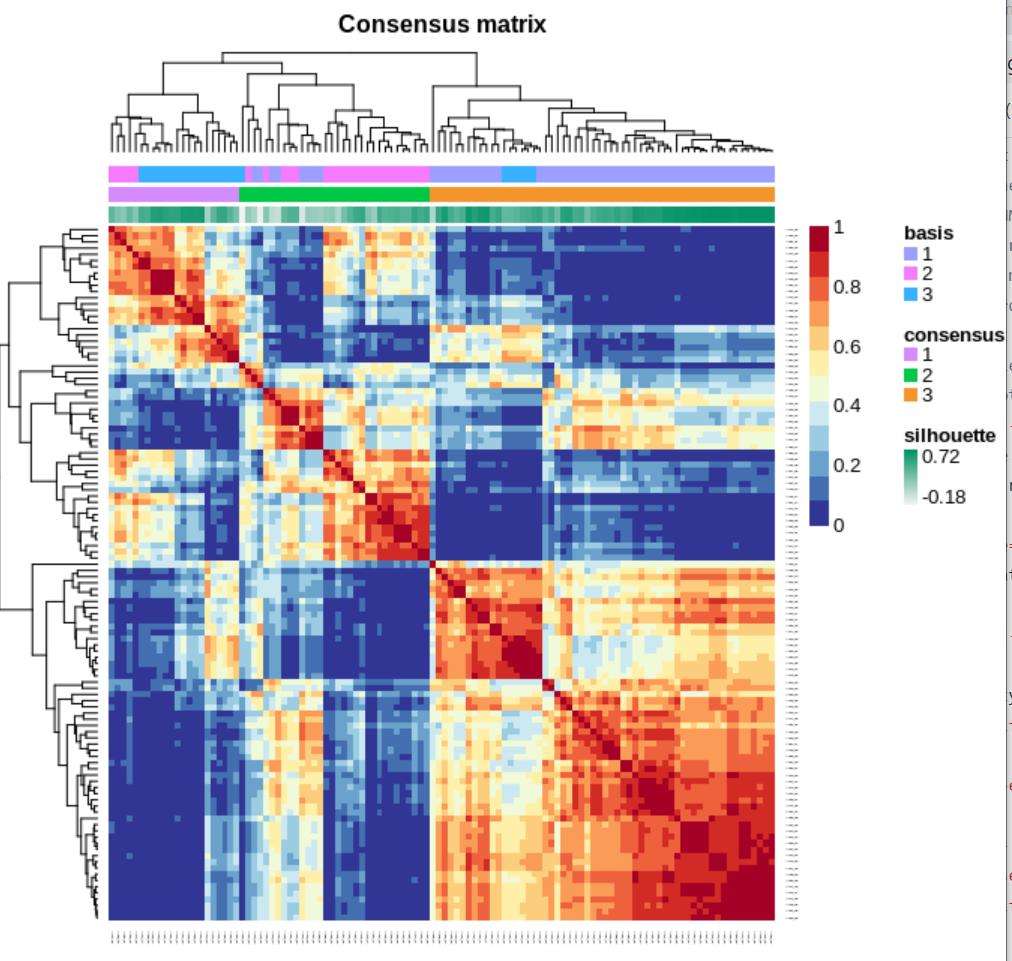
**

**Supplementary Figure 2. Cluster construction with the KCL BrainBank sample matrix, sorted by consensus, for the KCL BrainBank dataset.** The nsNMF hierarchical clustering algorithm was ran with k = 3, 100 runs and 1000 iterations.


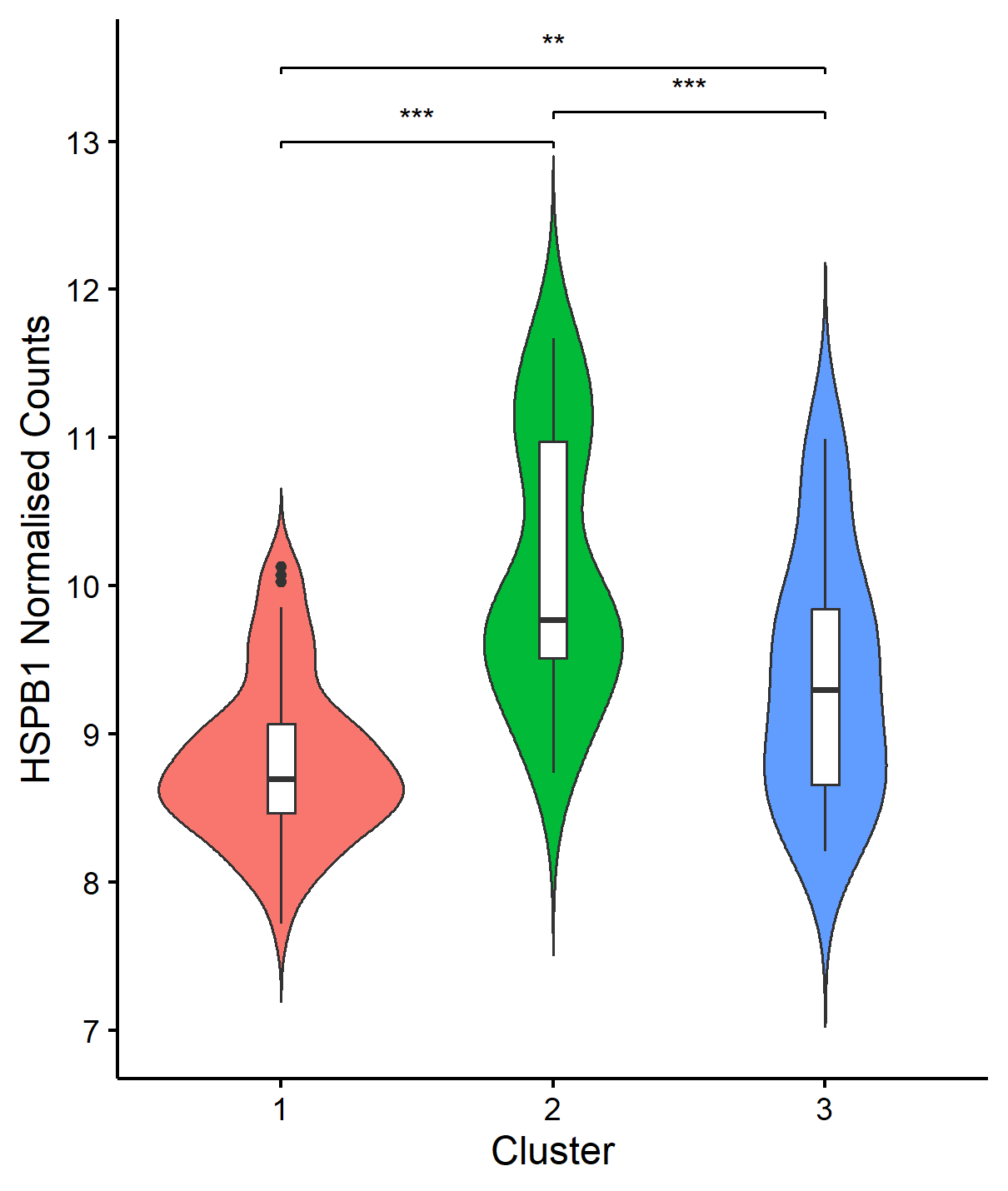

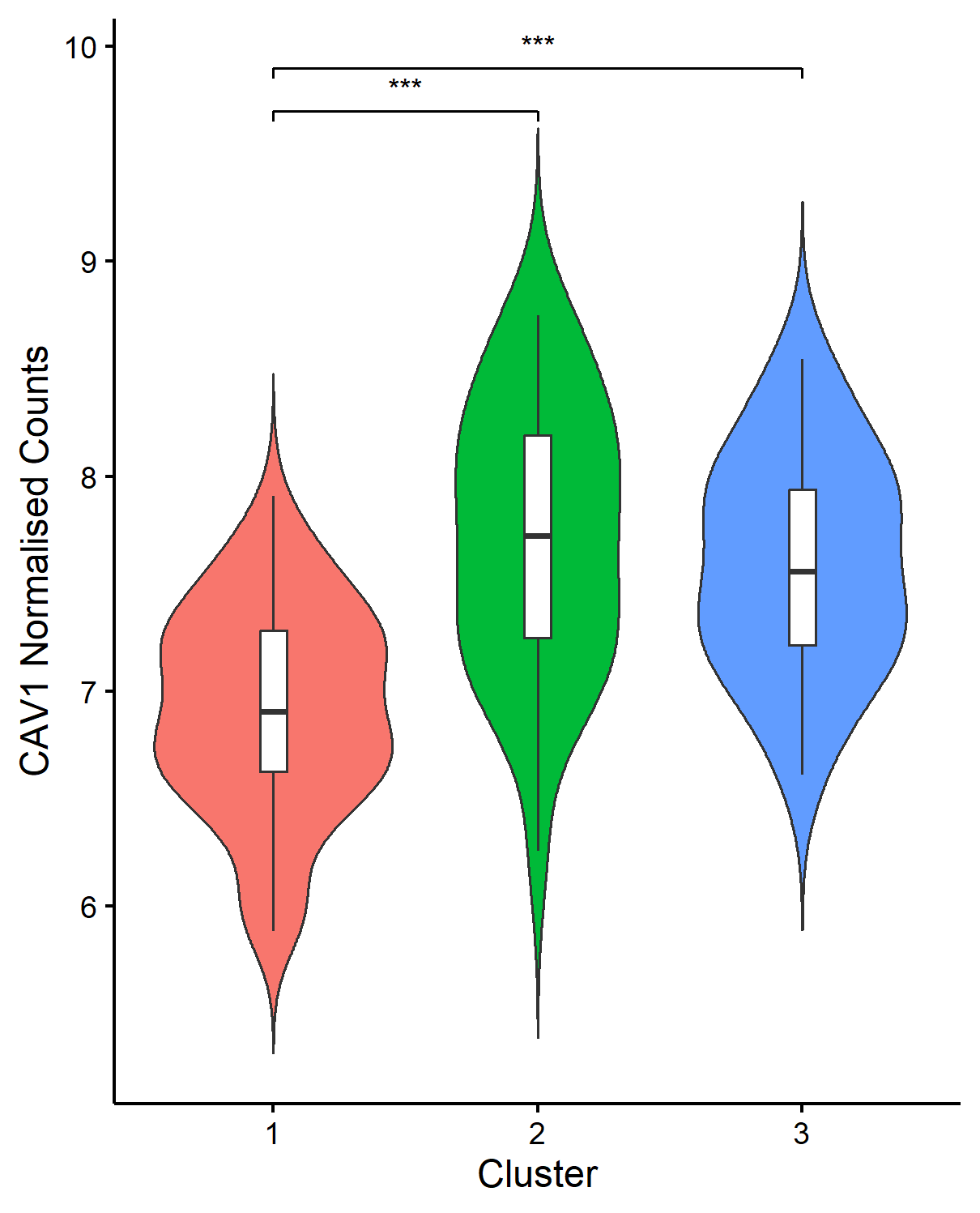

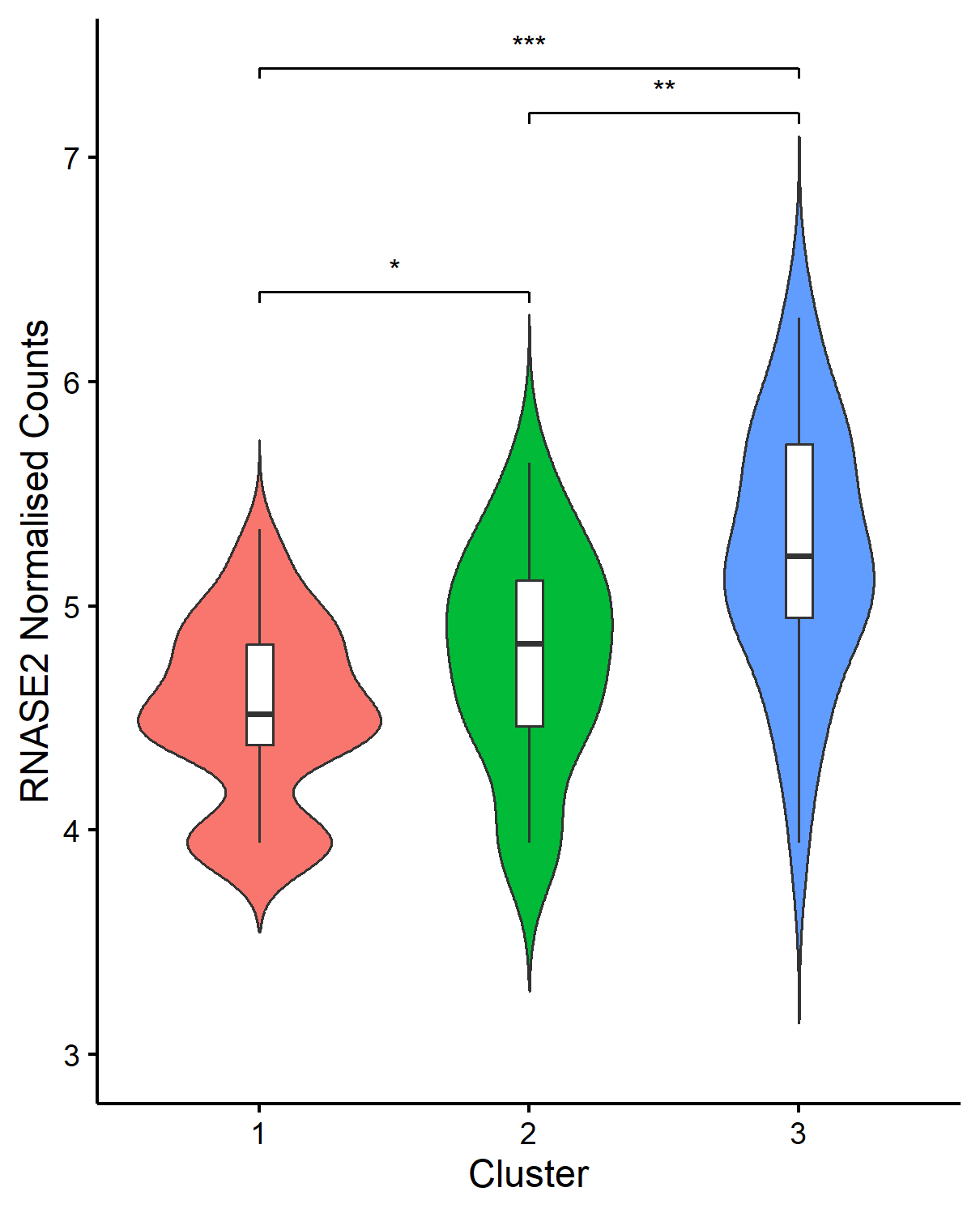

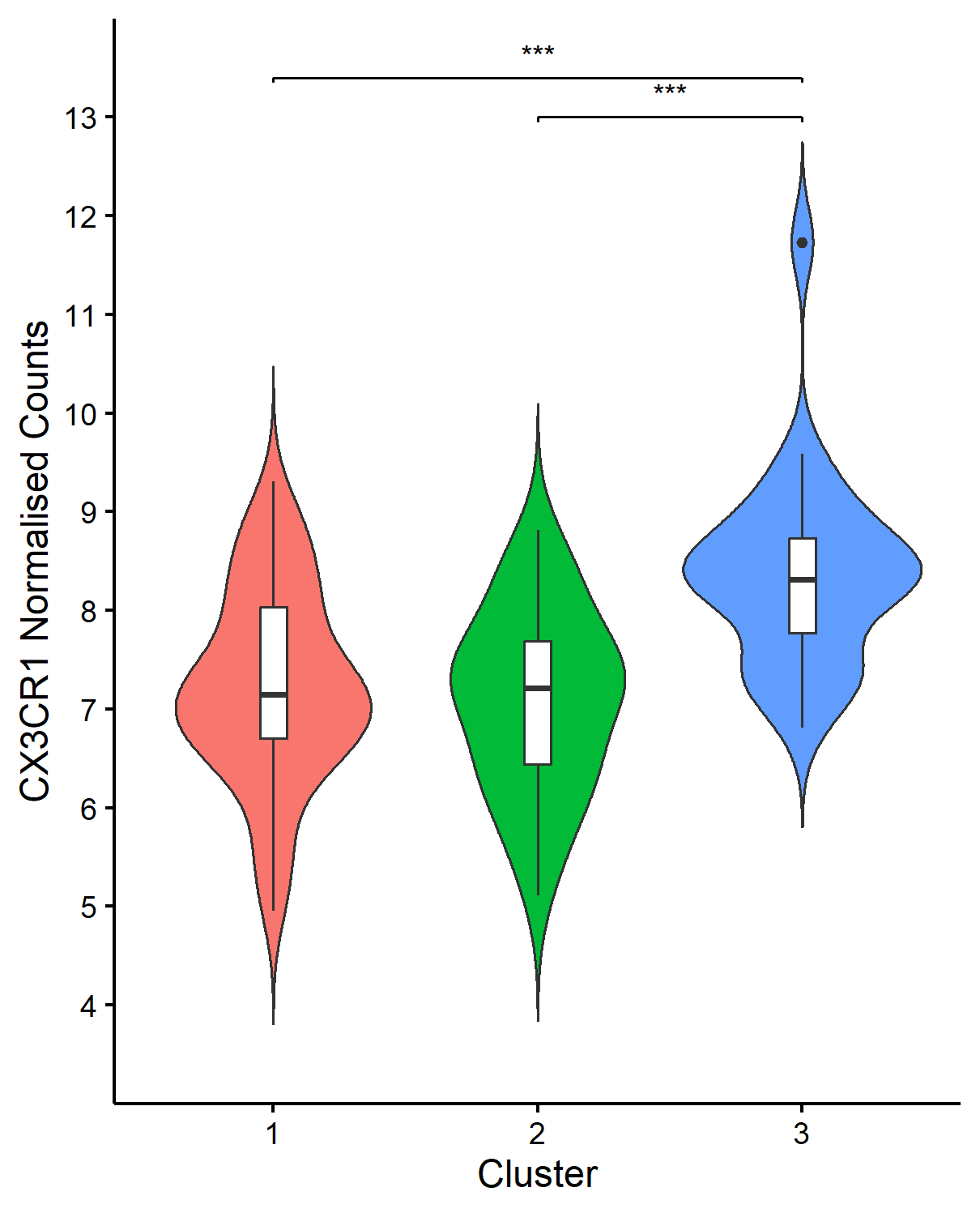


**A**

**B**

**C**

**D**

**E**

**Supplementary Figure 3. Comparison of the expression of the four identified ALS-related genes.** Genes are as follows: A) HSPB1, B) CAV1, C) CX3CR1, and D) RNASE2, in each cluster. Asterisks represent significance levels of * <0.05, ** < 0.01, *** < 0.0001 with post-hoc Tukey’s test after running one-way ANCOVA corrected for sex, age at death and post-mortem delay. E) Summary of the ANCOVA results assessing the differences in gene expression levels in ALS-linked genes between clusters. Bold p-values denote significance (< 0.05).


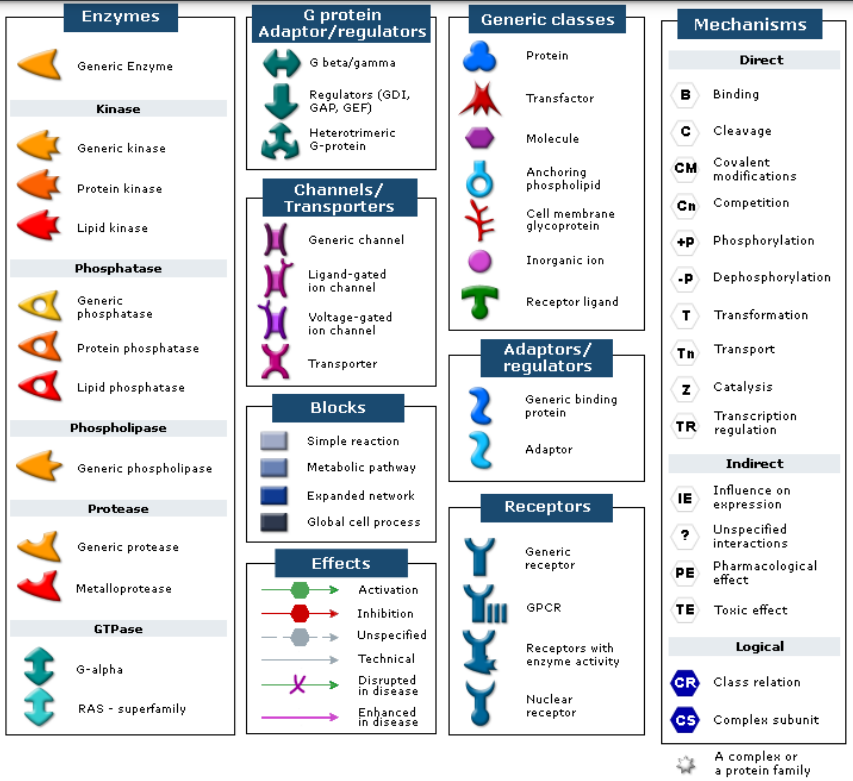


**Supplementary Figure 4. A key of all of the symbols present in the sub-cluster networks.**

**
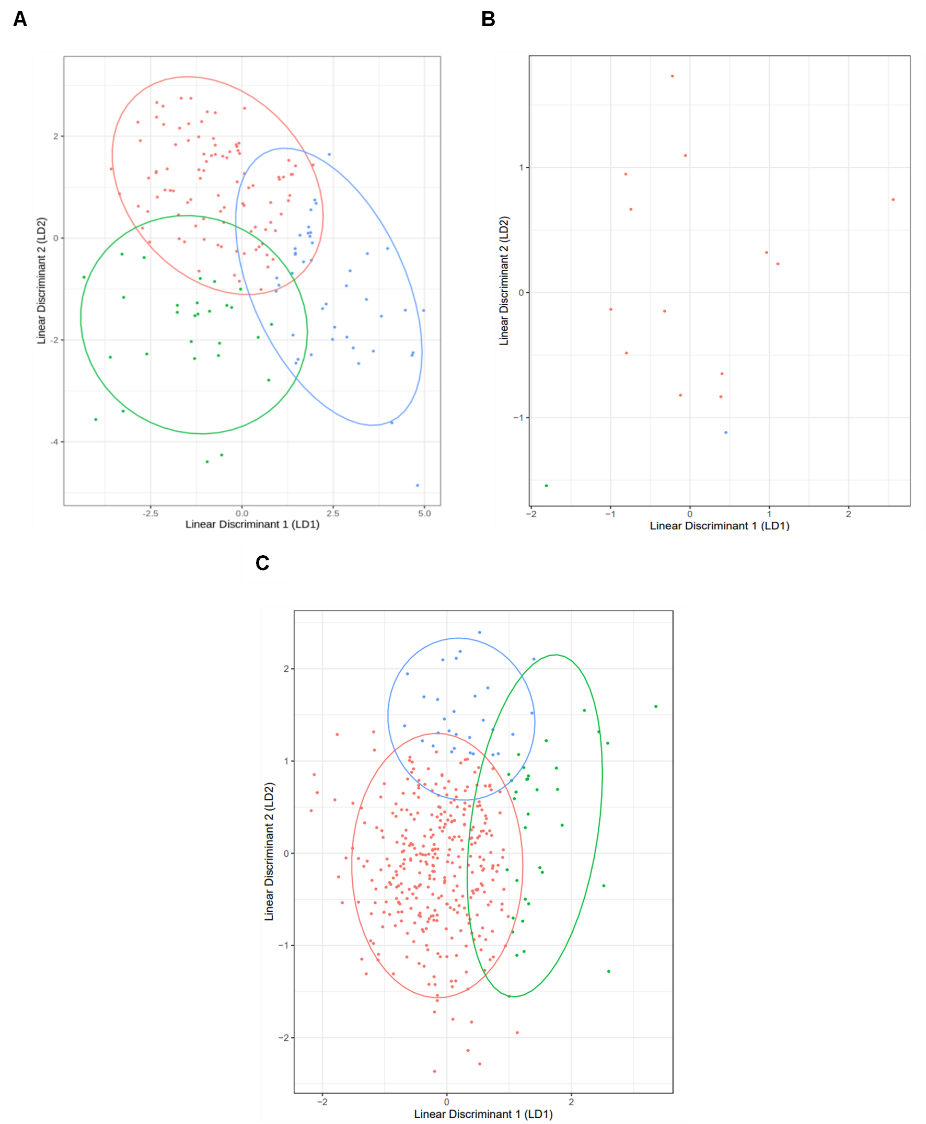
**

**Supplementary Figure 5. Sample assignment of A) TargetALS, B) Zucca and C) van Rheenen case datasets to the KCL BrainBank-defined clusters.** Linear discriminant analysis models were trained on the KCL sample assignments and informative genes shared with each dataset.


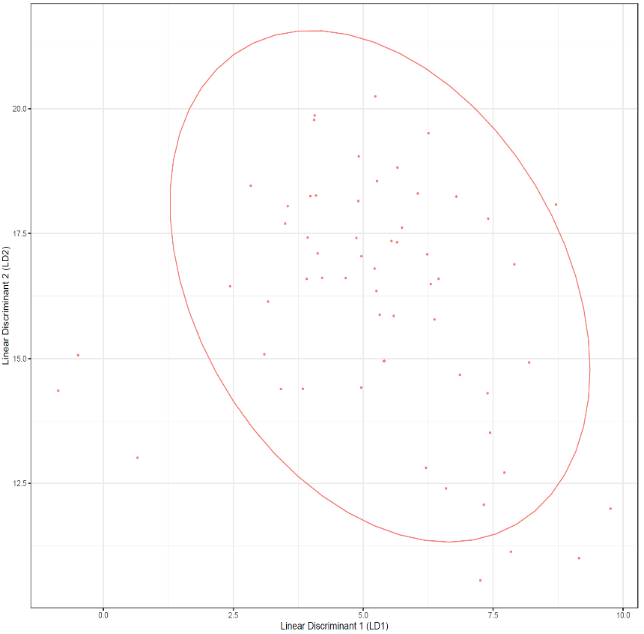


**A**

**B**

**C**


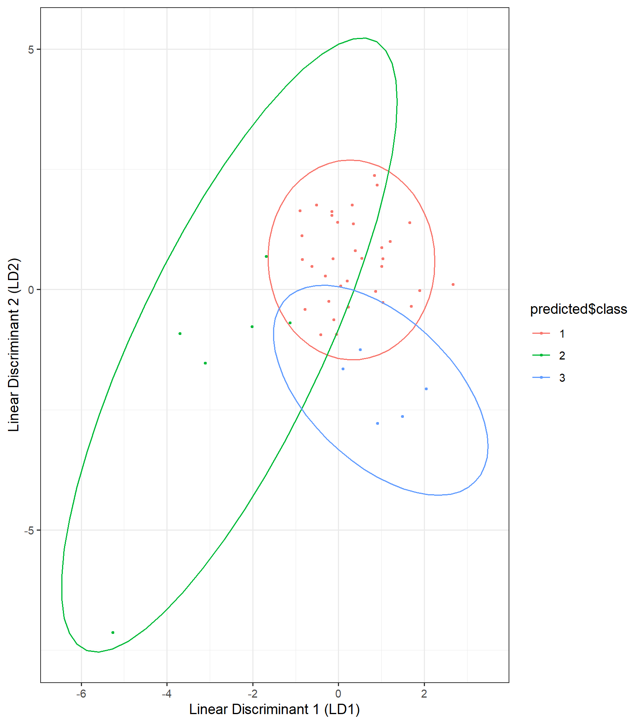

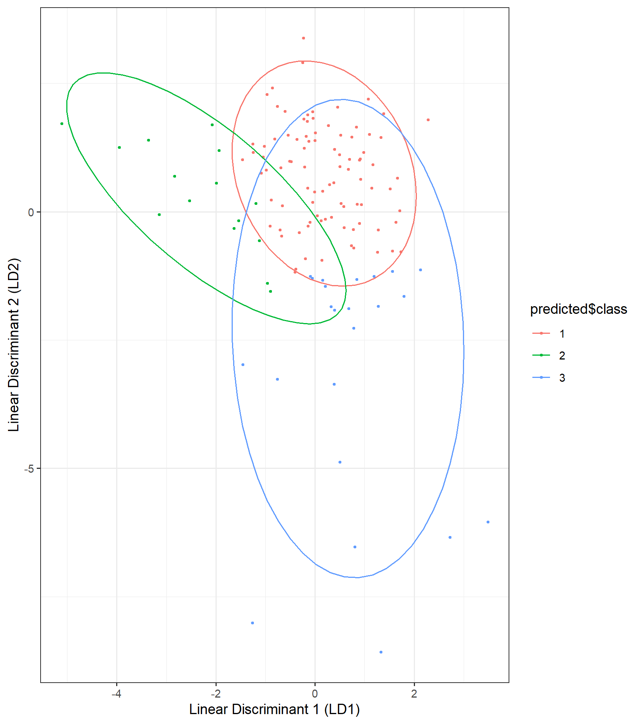


**Supplementary Figure 6. Sample assignment of A) KCL BrainBank controls, B) TargetALS occipital cortex and C) TargetALS cerebellum control datasets to the KCL BrainBank-defined clusters.** Linear discriminant analysis models were trained on the KCL sample assignments and informative genes shared with each dataset.


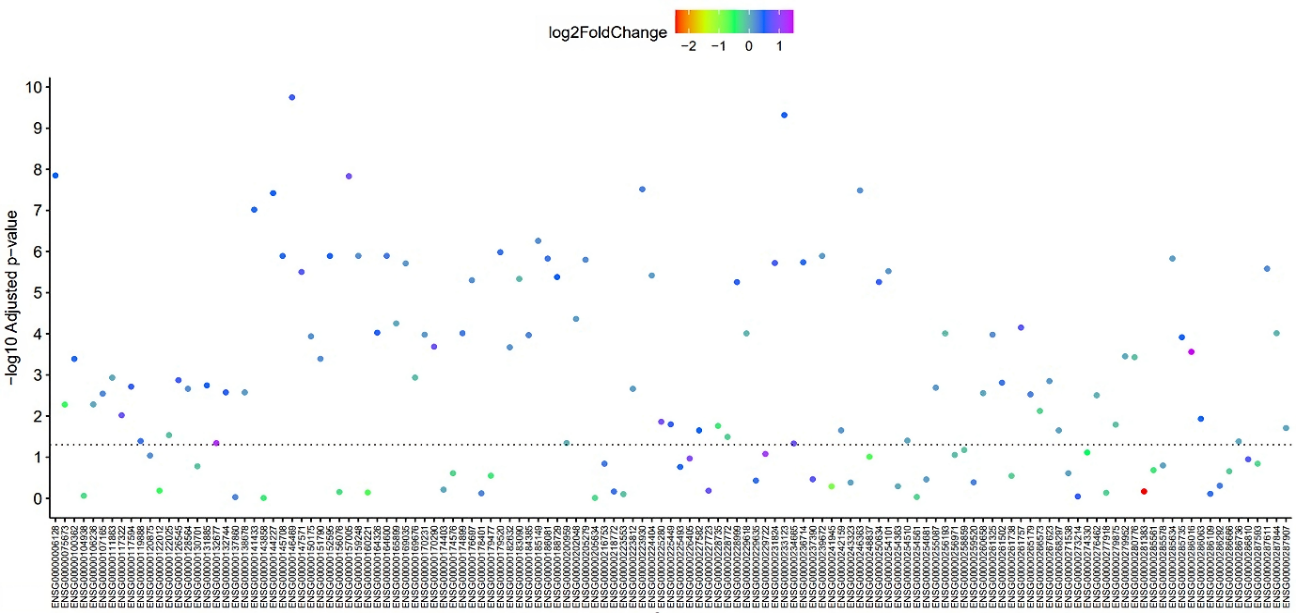


**Supplementary Figure 7. Dot chart demonstrating significant differences in expression of 87 of the 131 cluster one genes between KCL BrainBank cases and controls, coloured by log2 fold change.** An increase in log2 fold change means the gene is upregulated in cases, and vice versa. The dotted line represents the -log10 adjusted p-value that corresponds to a Benjamini-Hochberg p-value of 0.05.

**Supplementary Tables**

|  | **KCL BrainBank (Cases)** | **TargetALS** | **Zucca** | **van Rheenen** | **KCL BrainBank (Controls)** | **TargetALS (occipital cortex)** | **TargetALS (cerebellum)** |
| --- | --- | --- | --- | --- | --- | --- | --- |
| **Number of Individuals** | 112 | 93 | 15 | 397 | 59 | 45 | 123 |
| **Male (%)** | 65 (58·04%) | 55 (59·14%) | 7 (46·67%) | 239 (60·20%) | 26 (44·07%) | 22 (48.89%) | 71 (57.72%) |
| **Age (mean ± SD)** | 68·81 ± 12·71 | 64·21 ± 10·19 | 66·40 ± 9·12 | 62·17 ± 11·95 | 76·19 ± 14·58 | 64.40 ± 10.53 | 63.66 ± 9.34 |
| **PMD (mean ± SD)** | 26·05 ± 12·08 | 10·67 ± 6·24 | NA | NA | 37·56 ± 19·34 | 10.00 ± 7.12 | 10.12 ± 6.90 |

**Supplementary Table 1: Basic demographics of each of the seven datasets used in this study.** PMD: postmortem delay, measured in hours. Age refers to age of death in BrainBank and TargetALS datasets, and age at last blood draw in Zucca and van Rheenen.

|  | **KCL BrainBank** | **TargetALS** | **Zucca** | **van Rheenen** |
| --- | --- | --- | --- | --- |
| **Clinical** |  |  |  |  |
| Age at Symptom Onset | ✓ | ✓ | ✓ | ✓ |
| Age at Death | ✓ | ✓ |  |  |
| Disease Duration | ✓ | ✓ |  |  |
| Diagnostic Delay | ✓ | ✓ |  |  |
| Postmortem Delay | ✓ | ✓ |  |  |
| Survival | ✓ | ✓ |  |  |
| **Omics** |  |  |  |  |
| Telomere Length | ✓ |  |  |  |
| Mitochondrial DNA Copy Number | ✓ |  |  |  |
| Transcriptional Age Acceleration | ✓ | ✓ | ✓ | ✓ |
| Biological Age Acceleration | ✓ |  |  |  |

**Supplementary Table 2: Breakdown of the phenotypic variables available for each of the 4 case datasets used in this study.**

| **Cluster** | **Gene ID** | **Gene Symbol** | **Feature Score** | **Probability** |
| --- | --- | --- | --- | --- |
| 1 | ENSG00000164600 | NEUROD6 | 0·2928 | 0·7002 |
| 1 | ENSG00000100362 | PVALB | 0·2442 | 0·6736 |
| 1 | ENSG00000186081 | KRT5 | 0·2344 | 0·6725 |
| 1 | ENSG00000006128 | TAC1 | 0·2378 | 0·6502 |
| 1 | ENSG00000236714 | LINC01844 | 0·2060 | 0·6443 |
| 1 | ENSG00000141433 | ADCYAP1 | 0·1663 | 0·6258 |
| 1 | ENSG00000128564 | VGF | 0·1616 | 0·6254 |
| 1 | ENSG00000147571 | CRH | 0·1534 | 0·6083 |
| 1 | ENSG00000280776 | LINC01202 | 0·1441 | 0·6068 |
| 1 | ENSG00000205279 | CTXN3 | 0·1418 | 0·6043 |
| 1 | ENSG00000246363 | LINC02458 | 0·1693 | 0·6036 |
| 1 | ENSG00000164326 | CARTPT | 0·2121 | 0·6029 |
| 1 | ENSG00000233123 | LINC01007 | 0·1434 | 0·5992 |
| 1 | ENSG00000157005 | SST | 0·2557 | 0·5984 |
| 1 | ENSG00000223812 | PYDC2-AS1 | 0·1476 | 0·5963 |
| 1 | ENSG00000205634 | LINC00898 | 0·1320 | 0·5941 |
| 1 | ENSG00000224404 | Lnc-LARGE1-1 | 0·1353 | 0·5926 |
| 1 | ENSG00000151790 | TDO2 | 0·1494 | 0·5917 |
| 1 | ENSG00000122012 | SV2C | 0·1272 | 0·5900 |
| 1 | ENSG00000144227 | NXPH2 | 0·1520 | 0·5897 |
| 1 | ENSG00000261757 | miR-1255 | 0·1283 | 0·5827 |
| 1 | ENSG00000254363 | LOC101929719 | 0·1810 | 0·5804 |
| 1 | ENSG00000183090 | FREM3 | 0·1174 | 0·5758 |
| 1 | ENSG00000188729 | OSTN | 0·1506 | 0·5757 |
| 1 | ENSG00000159248 | GJD2 | 0·1175 | 0·5743 |
| 1 | ENSG00000285561 | None | 0·1102 | 0·5732 |
| 1 | ENSG00000261738 | MIR3976HG | 0·1128 | 0·5712 |
| 1 | ENSG00000131885 | KRT17P1 | 0·1079 | 0·5698 |
| 1 | ENSG00000261325 | LINC02192 | 0·1131 | 0·5683 |
| 1 | ENSG00000254561 | None | 0·1068 | 0·5679 |
| 1 | ENSG00000237390 | Lnc-RHBG-1 | 0·1131 | 0·5645 |
| 1 | ENSG00000242159 | ABCF2P1 | 0·1116 | 0·5637 |
| 1 | ENSG00000256193 | LINC00507 | 0·1380 | 0·5636 |
| 1 | ENSG00000130701 | RBBP8NL | 0·1008 | 0·5625 |
| 1 | ENSG00000265179 | Lnc-YES1-8 | 0·1087 | 0·5602 |
| 1 | ENSG00000169035 | KLK7 | 0·0983 | 0·5602 |
| 1 | ENSG00000150175 | FRMPD2B | 0·0995 | 0·5592 |
| 1 | ENSG00000271538 | LINC02427 | 0·1744 | 0·5588 |
| 1 | ENSG00000104938 | CLEC4M | 0·1060 | 0·5573 |
| 1 | ENSG00000228735 | LOC124901635 | 0·0935 | 0·5545 |
| 1 | ENSG00000170290 | SLN | 0·1750 | 0·5544 |
| 1 | ENSG00000281383 | Lnc-KCNE1B-157 | 0·0933 | 0·5532 |
| 1 | ENSG00000143858 | SYT2 | 0·0924 | 0·5522 |
| 1 | ENSG00000228999 | LINC01830 | 0·1127 | 0·5497 |
| 1 | ENSG00000254101 | LINC02055 | 0·0925 | 0·5489 |
| 1 | ENSG00000285735 | LINC02717 | 0·0886 | 0·5486 |
| 1 | ENSG00000276462 | LINC03025 | 0·0907 | 0·5477 |
| 1 | ENSG00000286736 | None | 0·0923 | 0·5462 |
| 1 | ENSG00000075673 | ATP12A | 0·0913 | 0·5442 |
| 1 | ENSG00000174403 | MIR1-1HG-AS1 | 0·0865 | 0·5438 |
| 1 | ENSG00000287844 | None | 0·0860 | 0·5438 |
| 1 | ENSG00000286063 | None | 0·0889 | 0·5432 |
| 1 | ENSG00000111863 | ADTRP | 0·1065 | 0·5421 |
| 1 | ENSG00000176697 | BDNF | 0·0847 | 0·5415 |
| 1 | ENSG00000287611 | Lnc-HRH4-2 | 0·0847 | 0·5403 |
| 1 | ENSG00000279875 | None | 0·0948 | 0·5391 |
| 1 | ENSG00000223930 | Lnc-ZMAT3-3 | 0·0928 | 0·5381 |
| 1 | ENSG00000160221 | GATD3A | 0·0864 | 0·5375 |
| 1 | ENSG00000225493 | LINC01107 | 0·0903 | 0·5375 |
| 1 | ENSG00000279952 | None | 0·0826 | 0·5373 |
| 1 | ENSG00000185149 | NPY2R | 0·1257 | 0·5354 |
| 1 | ENSG00000248837 | LOC105374524 | 0·0860 | 0·5341 |
| 1 | ENSG00000202048 | SNORD114-20 | 0·0838 | 0·5338 |
| 1 | ENSG00000250634 | LINC01182 | 0·0784 | 0·5329 |
| 1 | ENSG00000169676 | DRD5 | 0·1015 | 0·5324 |
| 1 | ENSG00000268297 | CLEC4GP1 | 0·0753 | 0·5315 |
| 1 | ENSG00000120875 | DUSP4 | 0·0753 | 0·5310 |
| 1 | ENSG00000122025 | FLT3 | 0·0920 | 0·5308 |
| 1 | ENSG00000231824 | AKAIN1 | 0·0803 | 0·5303 |
| 1 | ENSG00000145708 | CRHBP | 0·1696 | 0·5296 |
| 1 | ENSG00000182632 | CCNYL2 | 0·0952 | 0·5295 |
| 1 | ENSG00000174576 | NPAS4 | 0·0765 | 0·5284 |
| 1 | ENSG00000241945 | PWP2 | 0·0735 | 0·5259 |
| 1 | ENSG00000285578 | Lnc-DUSP22-2 | 0·0837 | 0·5259 |
| 1 | ENSG00000165899 | OTOGL | 0·0717 | 0·5256 |
| 1 | ENSG00000267623 | Cyclin Y-Like Pseudogene | 0·0717 | 0·5248 |
| 1 | ENSG00000152595 | MEPE | 0·1049 | 0·5242 |
| 1 | ENSG00000138678 | GPAT3 | 0·0725 | 0·5235 |
| 1 | ENSG00000239672 | NME1 | 0·0979 | 0·5225 |
| 1 | ENSG00000179477 | ALOX12B | 0·0699 | 0·5218 |
| 1 | ENSG00000254510 | Lnc-NPAS4-1 | 0·0688 | 0·5217 |
| 1 | ENSG00000286292 | None | 0·1351 | 0·5215 |
| 1 | ENSG00000274330 | ADAM20 Pseudogene | 0·0686 | 0·5209 |
| 1 | ENSG00000106236 | NPTX2 | 0·0749 | 0·5204 |
| 1 | ENSG00000285634 | LOC105376121 | 0·0825 | 0·5184 |
| 1 | ENSG00000170231 | FABP6 | 0·1070 | 0·5184 |
| 1 | ENSG00000146469 | VIP | 0·0681 | 0·5180 |
| 1 | ENSG00000117594 | HSD11B1 | 0·2147 | 0·5167 |
| 1 | ENSG00000254681 | PKD1P5 | 0·1017 | 0·5151 |
| 1 | ENSG00000258859 | LINC02296 | 0·0909 | 0·5148 |
| 1 | ENSG00000260658 | Lnc-CDH8-10 | 0·1106 | 0·5143 |
| 1 | ENSG00000286016 | None | 0·0947 | 0·5108 |
| 1 | ENSG00000229618 | Lnc-ARL4A-54 | 0·0836 | 0·5104 |
| 1 | ENSG00000132677 | RHBG | 0·0677 | 0·5103 |
| 1 | ENSG00000287593 | Antisense to ADTRP | 0·0751 | 0·5091 |
| 1 | ENSG00000229722 | Lnc-SGK1-3 | 0·1479 | 0·5090 |
| 1 | ENSG00000225280 | LOC105372558 | 0·0998 | 0·5087 |
| 1 | ENSG00000227582 | ADGRF5P1 | 0·1094 | 0·5078 |
| 1 | ENSG00000119888 | EPCAM | 0·0706 | 0·5069 |
| 1 | ENSG00000156076 | WIF1 | 0·0761 | 0·5035 |
| 1 | ENSG00000174899 | SLC66A1L | 0·0722 | 0·5015 |
| 1 | ENSG00000229635 | Pseudogene Similar to Part of FBXW1B | 0·0740 | 0·5011 |
| 1 | ENSG00000226405 | HSD17B12 Pseudogene | 0·1263 | 0·5006 |
| 1 | ENSG00000286109 | Lnc-KCNK1-1 | 0·0681 | 0·5001 |
| 1 | ENSG00000287907 | None | 0·0696 | 0·4981 |
| 1 | ENSG00000228772 | Lnc-PRL-3 | 0·0822 | 0·4979 |
| 1 | ENSG00000255087 | LOC101929473 | 0·0713 | 0·4973 |
| 1 | ENSG00000107165 | TYRP1 | 0·1184 | 0·4971 |
| 1 | ENSG00000126545 | CSN1S1 | 0·0804 | 0·4955 |
| 1 | ENSG00000184385 | UMODL1-AS1 | 0·0734 | 0·4942 |
| 1 | ENSG00000261502 | Lnc-CDH8-10 | 0·1012 | 0·4918 |
| 1 | ENSG00000225449 | RAB6C-AS1 | 0·1405 | 0·4911 |
| 1 | ENSG00000137860 | SLC28A2 | 0·1007 | 0·4906 |
| 1 | ENSG00000259520 | SLC28A2-AS1 | 0·1289 | 0·4866 |
| 1 | ENSG00000266573 | Lnc-HRH4-7 | 0·0936 | 0·4845 |
| 1 | ENSG00000132744 | ACY3 | 0·0824 | 0·4835 |
| 1 | ENSG00000234665 | LERFS | 0·0886 | 0·4825 |
| 1 | ENSG00000227723 | Lnc-MYB-5 | 0·1588 | 0·4812 |
| 1 | ENSG00000273214 | Lnc-POLR2F-1 | 0·1015 | 0·4796 |
| 1 | ENSG00000218772 | FAM8A6P | 0·1215 | 0·4786 |
| 1 | ENSG00000286666 | None | 0·0834 | 0·4784 |
| 1 | ENSG00000179520 | SLC17A8 | 0·0938 | 0·4775 |
| 1 | ENSG00000277918 | RNVU1-28 | 0·1078 | 0·4766 |
| 1 | ENSG00000216753 | HMGA1P7 | 0·1011 | 0·4670 |
| 1 | ENSG00000117322 | CR2 | 0·0698 | 0·4657 |
| 1 | ENSG00000256971 | LINC00508 | 0·0889 | 0·4610 |
| 1 | ENSG00000286810 | None | 0·0805 | 0·4581 |
| 1 | ENSG00000178401 | DNAJC22 | 0·0983 | 0·4531 |
| 1 | ENSG00000223553 | SMPD4P1 | 0·0675 | 0·4308 |
| 1 | ENSG00000200959 | SNORA74A | 0·0681 | 0·4273 |
| 1 | ENSG00000243323 | PTPRVP | 0·0713 | 0·4221 |
| 2 | ENSG00000175084 | DES | 0·8407 | 0·9591 |
| 2 | ENSG00000163017 | ACTG2 | 0·5157 | 0·8158 |
| 2 | ENSG00000105641 | SLC5A5 | 0·4185 | 0·7857 |
| 2 | ENSG00000168542 | COL3A1 | 0·4631 | 0·7699 |
| 2 | ENSG00000196616 | ADH1B | 0·4705 | 0·7698 |
| 2 | ENSG00000108821 | COL1A1 | 0·4247 | 0·7545 |
| 2 | ENSG00000118271 | TTR | 0·3322 | 0·7462 |
| 2 | ENSG00000096696 | DSP | 0·3321 | 0·7450 |
| 2 | ENSG00000144810 | COL8A1 | 0·3937 | 0·7423 |
| 2 | ENSG00000111341 | MGP | 0·3728 | 0·7233 |
| 2 | ENSG00000133392 | MYH11 | 0·3091 | 0·7219 |
| 2 | ENSG00000149596 | JPH2 | 0·2880 | 0·7045 |
| 2 | ENSG00000101335 | MYL9 | 0·2608 | 0·6917 |
| 2 | ENSG00000112214 | FHL5 | 0·2641 | 0·6903 |
| 2 | ENSG00000130176 | CNN1 | 0·2413 | 0·6892 |
| 2 | ENSG00000198467 | TPM2 | 0·2413 | 0·6876 |
| 2 | ENSG00000187955 | COL14A1 | 0·2812 | 0·6777 |
| 2 | ENSG00000124253 | PCK1 | 0·3288 | 0·6772 |
| 2 | ENSG00000141052 | MYOCD | 0·2321 | 0·6735 |
| 2 | ENSG00000013588 | GPRC5A | 0·3677 | 0·6732 |
| 2 | ENSG00000163817 | SLC6A20 | 0·2255 | 0·6732 |
| 2 | ENSG00000143867 | OSR1 | 0·2179 | 0·6726 |
| 2 | ENSG00000115602 | IL1RL1 | 0·2727 | 0·6724 |
| 2 | ENSG00000054598 | FOXC1 | 0·2789 | 0·6712 |
| 2 | ENSG00000287338 | Lnc-TLR5-1 | 0·3582 | 0·6663 |
| 2 | ENSG00000107796 | ACTA2 | 0·2111 | 0·6644 |
| 2 | ENSG00000115590 | IL1R2 | 0·3469 | 0·6638 |
| 2 | ENSG00000115648 | MLPH | 0·2497 | 0·6630 |
| 2 | ENSG00000112499 | SLC22A2 | 0·2091 | 0·6619 |
| 2 | ENSG00000205038 | PKHD1L1 | 0·2324 | 0·6579 |
| 2 | ENSG00000164692 | COL1A2 | 0·2609 | 0·6567 |
| 2 | ENSG00000162458 | FBLIM1 | 0·2227 | 0·6525 |
| 2 | ENSG00000269113 | TRABD2B | 0·2354 | 0·6522 |
| 2 | ENSG00000112837 | TBX18 | 0·2049 | 0·6503 |
| 2 | ENSG00000152779 | SLC16A12 | 0·1988 | 0·6485 |
| 2 | ENSG00000131471 | AOC3 | 0·2022 | 0·6476 |
| 2 | ENSG00000241644 | INMT | 0·1894 | 0·6471 |
| 2 | ENSG00000186564 | FOXD2 | 0·2019 | 0·6410 |
| 2 | ENSG00000107438 | PDLIM1 | 0·2306 | 0·6385 |
| 2 | ENSG00000161638 | ITGA5 | 0·2232 | 0·6385 |
| 2 | ENSG00000149257 | SERPINH1 | 0·3113 | 0·6370 |
| 2 | ENSG00000177575 | CD163 | 0·4013 | 0·6367 |
| 2 | ENSG00000164707 | SLC13A4 | 0·1768 | 0·6366 |
| 2 | ENSG00000149591 | TAGLN | 0·1862 | 0·6346 |
| 2 | ENSG00000149573 | MPZL2 | 0·2436 | 0·6342 |
| 2 | ENSG00000112936 | C7 | 0·3239 | 0·6312 |
| 2 | ENSG00000237424 | FOXD2-AS1 | 0·1845 | 0·6312 |
| 2 | ENSG00000226390 | KCNQ2-AS1 | 0·1684 | 0·6311 |
| 2 | ENSG00000124107 | SLPI | 0·1963 | 0·6308 |
| 2 | ENSG00000185585 | OLFML2A | 0·1824 | 0·6269 |
| 2 | ENSG00000176692 | FOXC2 | 0·1617 | 0·6250 |
| 2 | ENSG00000152049 | KCNE4 | 0·2716 | 0·6242 |
| 2 | ENSG00000000971 | CFH | 0·2495 | 0·6231 |
| 2 | ENSG00000173597 | SULT1B1 | 0·2169 | 0·6223 |
| 2 | ENSG00000144837 | PLA1A | 0·2179 | 0·6212 |
| 2 | ENSG00000115604 | IL18R1 | 0·1820 | 0·6208 |
| 2 | ENSG00000279821 | None | 0·1868 | 0·6201 |
| 2 | ENSG00000112175 | BMP5 | 0·1541 | 0·6177 |
| 2 | ENSG00000173530 | TNFRSF10D | 0·2506 | 0·6163 |
| 2 | ENSG00000015520 | NPC1L1 | 0·1809 | 0·6157 |
| 2 | ENSG00000142173 | COL6A2 | 0·1894 | 0·6138 |
| 2 | ENSG00000027644 | INSRR | 0·1484 | 0·6127 |
| 2 | ENSG00000102802 | MEDAG | 0·1481 | 0·6126 |
| 2 | ENSG00000099994 | SUSD2 | 0·1962 | 0·6125 |
| 2 | ENSG00000198848 | CES1 | 0·1597 | 0·6098 |
| 2 | ENSG00000198959 | TGM2 | 0·1946 | 0·6095 |
| 2 | ENSG00000143546 | S100A8 | 0·3517 | 0·6077 |
| 2 | ENSG00000184811 | TRARG1 | 0·1404 | 0·6053 |
| 2 | ENSG00000130635 | COL5A1 | 0·1363 | 0·6012 |
| 2 | ENSG00000163736 | PPBP | 0·1520 | 0·6009 |
| 2 | ENSG00000163220 | S100A9 | 0·3857 | 0·6009 |
| 2 | ENSG00000172935 | MRGPRF | 0·1409 | 0·5978 |
| 2 | ENSG00000166523 | CLEC4E | 0·1432 | 0·5978 |
| 2 | ENSG00000118729 | CASQ2 | 0·1389 | 0·5977 |
| 2 | ENSG00000265107 | GJA5 | 0·1532 | 0·5977 |
| 2 | ENSG00000115594 | IL1R1 | 0·2082 | 0·5974 |
| 2 | ENSG00000124212 | PTGIS | 0·1438 | 0·5956 |
| 2 | ENSG00000165474 | GJB2 | 0·1371 | 0·5954 |
| 2 | ENSG00000196154 | S100A4 | 0·2512 | 0·5943 |
| 2 | ENSG00000128274 | A4GALT | 0·2053 | 0·5942 |
| 2 | ENSG00000173110 | HSPA6 | 0·3594 | 0·5917 |
| 2 | ENSG00000164761 | TNFRSF11B | 0·2011 | 0·5914 |
| 2 | ENSG00000163431 | LMOD1 | 0·1365 | 0·5912 |
| 2 | ENSG00000135842 | NIBAN1 | 0·2978 | 0·5905 |
| 2 | ENSG00000167244 | IGF2 | 0·1261 | 0·5904 |
| 2 | ENSG00000106366 | SERPINE1 | 0·1559 | 0·5904 |
| 2 | ENSG00000174348 | PODN | 0·1846 | 0·5897 |
| 2 | ENSG00000206538 | VGLL3 | 0·1305 | 0·5883 |
| 2 | ENSG00000122176 | FMOD | 0·1288 | 0·5881 |
| 2 | ENSG00000088882 | CPXM1 | 0·1336 | 0·5870 |
| 2 | ENSG00000221869 | CEBPD | 0·1630 | 0·5867 |
| 2 | ENSG00000074219 | TEAD2 | 0·1467 | 0·5861 |
| 2 | ENSG00000153404 | PLEKHG4B | 0·1228 | 0·5853 |
| 2 | ENSG00000120708 | TGFBI | 0·2404 | 0·5845 |
| 2 | ENSG00000047617 | ANO2 | 0·1433 | 0·5842 |
| 2 | ENSG00000142973 | CYP4B1 | 0·1186 | 0·5828 |
| 2 | ENSG00000260337 | Lnc-FAM174B-1 | 0·1209 | 0·5816 |
| 2 | ENSG00000125733 | TRIP10 | 0·1981 | 0·5798 |
| 2 | ENSG00000244734 | HBB | 0·1650 | 0·5781 |
| 2 | ENSG00000138722 | MMRN1 | 0·1785 | 0·5778 |
| 2 | ENSG00000110852 | CLEC2B | 0·2295 | 0·5774 |
| 2 | ENSG00000166482 | MFAP4 | 0·1255 | 0·5773 |
| 2 | ENSG00000101187 | SLCO4A1 | 0·1270 | 0·5771 |
| 2 | ENSG00000106211 | HSPB1 | 0·1555 | 0·5770 |
| 2 | ENSG00000137834 | SMAD6 | 0·1622 | 0·5762 |
| 2 | ENSG00000073792 | IGF2BP2 | 0·1389 | 0·5754 |
| 2 | ENSG00000164294 | GPX8 | 0·1488 | 0·5753 |
| 2 | ENSG00000142798 | HSPG2 | 0·1443 | 0·5747 |
| 2 | ENSG00000137507 | LRRC32 | 0·1669 | 0·5745 |
| 2 | ENSG00000140682 | TGFB1I1 | 0·1366 | 0·5739 |
| 2 | ENSG00000187498 | COL4A1 | 0·1411 | 0·5727 |
| 2 | ENSG00000188536 | HBA2 | 0·1400 | 0·5720 |
| 2 | ENSG00000279142 | None | 0·1117 | 0·5718 |
| 2 | ENSG00000125810 | CD93 | 0·3224 | 0·5718 |
| 2 | ENSG00000184058 | TBX1 | 0·1091 | 0·5717 |
| 2 | ENSG00000281732 | LOC284933 | 0·1097 | 0·5715 |
| 2 | ENSG00000287652 | Antisense to DGCR8 and CDC45 | 0·1736 | 0·5709 |
| 2 | ENSG00000173641 | HSPB7 | 0·1194 | 0·5700 |
| 2 | ENSG00000159403 | C1R | 0·2205 | 0·5690 |
| 2 | ENSG00000206172 | HBA1 | 0·1478 | 0·5680 |
| 2 | ENSG00000159212 | CLIC6 | 0·1114 | 0·5659 |
| 2 | ENSG00000100985 | MMP9 | 0·1078 | 0·5650 |
| 2 | ENSG00000250863 | Lnc-KCTD8-1 | 0·2379 | 0·5643 |
| 2 | ENSG00000169429 | CXCL8 | 0·1011 | 0·5636 |
| 2 | ENSG00000177464 | GPR4 | 0·1500 | 0·5636 |
| 2 | ENSG00000162998 | FRZB | 0·1011 | 0·5635 |
| 2 | ENSG00000160183 | TMPRSS3 | 0·1281 | 0·5617 |
| 2 | ENSG00000109758 | HGFAC | 0·0997 | 0·5615 |
| 2 | ENSG00000233117 | LINC00702 | 0·1065 | 0·5614 |
| 2 | ENSG00000183508 | TENT5C | 0·1446 | 0·5602 |
| 2 | ENSG00000258647 | LINC00930 | 0·0996 | 0·5592 |
| 2 | ENSG00000173421 | IHO1 | 0·1189 | 0·5576 |
| 2 | ENSG00000111057 | KRT18 | 0·1250 | 0·5570 |
| 2 | ENSG00000060138 | YBX3 | 0·1784 | 0·5568 |
| 2 | ENSG00000128917 | DLL4 | 0·1135 | 0·5567 |
| 2 | ENSG00000124762 | CDKN1A | 0·1752 | 0·5554 |
| 2 | ENSG00000239677 | PDZRN3-AS1 | 0·0937 | 0·5545 |
| 2 | ENSG00000243620 | Lnc-ZIC4-3 | 0·1434 | 0·5534 |
| 2 | ENSG00000249307 | LINC01088 | 0·1275 | 0·5529 |
| 2 | ENSG00000115607 | IL18RAP | 0·1272 | 0·5526 |
| 2 | ENSG00000261170 | LOC107984827 | 0·1218 | 0·5520 |
| 2 | ENSG00000138061 | CYP1B1 | 0·1214 | 0·5517 |
| 2 | ENSG00000106624 | AEBP1 | 0·1317 | 0·5514 |
| 2 | ENSG00000117595 | IRF6 | 0·0949 | 0·5510 |
| 2 | ENSG00000187479 | C11orf96 | 0·0954 | 0·5504 |
| 2 | ENSG00000179023 | KLHDC7A | 0·0897 | 0·5499 |
| 2 | ENSG00000168427 | KLHL30 | 0·0902 | 0·5498 |
| 2 | ENSG00000250033 | SLC7A11-AS1 | 0·1174 | 0·5488 |
| 2 | ENSG00000183888 | SRARP | 0·0986 | 0·5487 |
| 2 | ENSG00000162383 | SLC1A7 | 0·2594 | 0·5470 |
| 2 | ENSG00000185201 | IFITM2 | 0·2451 | 0·5458 |
| 2 | ENSG00000125430 | HS3ST3B1 | 0·1002 | 0·5457 |
| 2 | ENSG00000182718 | ANXA2 | 0·2208 | 0·5457 |
| 2 | ENSG00000253123 | LOC100507403 | 0·0915 | 0·5454 |
| 2 | ENSG00000094963 | FMO2 | 0·1120 | 0·5448 |
| 2 | ENSG00000229380 | PRKAR1B-AS2 | 0·0871 | 0·5447 |
| 2 | ENSG00000285938 | BCL6-AS1 | 0·1633 | 0·5444 |
| 2 | ENSG00000149452 | SLC22A8 | 0·1339 | 0·5427 |
| 2 | ENSG00000163739 | CXCL1 | 0·0862 | 0·5420 |
| 2 | ENSG00000196954 | CASP4 | 0·2211 | 0·5413 |
| 2 | ENSG00000173068 | BNC2 | 0·2439 | 0·5410 |
| 2 | ENSG00000003436 | TFPI | 0·1344 | 0·5407 |
| 2 | ENSG00000171345 | KRT19 | 0·0834 | 0·5407 |
| 2 | ENSG00000253686 | LINC01484 | 0·1257 | 0·5405 |
| 2 | ENSG00000004939 | SLC4A1 | 0·1226 | 0·5398 |
| 2 | ENSG00000112303 | VNN2 | 0·1302 | 0·5396 |
| 2 | ENSG00000106034 | CPED1 | 0·1185 | 0·5393 |
| 2 | ENSG00000204389 | HSPA1A | 0·1690 | 0·5393 |
| 2 | ENSG00000262152 | GREP1 | 0·0831 | 0·5389 |
| 2 | ENSG00000148604 | RGR | 0·0865 | 0·5387 |
| 2 | ENSG00000187922 | LCN10 | 0·0839 | 0·5387 |
| 2 | ENSG00000126778 | SIX1 | 0·1048 | 0·5384 |
| 2 | ENSG00000174226 | SNX31 | 0·0937 | 0·5383 |
| 2 | ENSG00000164867 | NOS3 | 0·1047 | 0·5379 |
| 2 | ENSG00000187513 | GJA4 | 0·0914 | 0·5377 |
| 2 | ENSG00000229926 | IL9RP1 | 0·0889 | 0·5374 |
| 2 | ENSG00000276317 | Lnc-GATA5-10 | 0·0815 | 0·5371 |
| 2 | ENSG00000173918 | C1QTNF1 | 0·1052 | 0·5369 |
| 2 | ENSG00000256955 | Lnc-MMP17-1 | 0·0837 | 0·5357 |
| 2 | ENSG00000163638 | ADAMTS9 | 0·1670 | 0·5355 |
| 2 | ENSG00000077238 | IL4R | 0·1918 | 0·5351 |
| 2 | ENSG00000100336 | APOL4 | 0·3160 | 0·5349 |
| 2 | ENSG00000047457 | CP | 0·3633 | 0·5349 |
| 2 | ENSG00000162747 | FCGR3B | 0·1056 | 0·5344 |
| 2 | ENSG00000278130 | FAM166A Pseudogene | 0·0809 | 0·5335 |
| 2 | ENSG00000151929 | BAG3 | 0·1593 | 0·5334 |
| 2 | ENSG00000272512 | Lnc-HES4-2 | 0·0765 | 0·5332 |
| 2 | ENSG00000165507 | DEPP1 | 0·1676 | 0·5330 |
| 2 | ENSG00000186150 | UBL4B | 0·0873 | 0·5326 |
| 2 | ENSG00000129654 | FOXJ1 | 0·1860 | 0·5320 |
| 2 | ENSG00000168079 | SCARA5 | 0·0786 | 0·5318 |
| 2 | ENSG00000230122 | ECEL1P3 | 0·0777 | 0·5313 |
| 2 | ENSG00000241158 | ADAMTS9-AS1 | 0·1303 | 0·5305 |
| 2 | ENSG00000122862 | SRGN | 0·1557 | 0·5294 |
| 2 | ENSG00000135245 | HILPDA | 0·1222 | 0·5294 |
| 2 | ENSG00000050327 | ARHGEF5 | 0·0878 | 0·5292 |
| 2 | ENSG00000233588 | CYP51A1P2 | 0·1109 | 0·5285 |
| 2 | ENSG00000232803 | SLCO4A1-AS1 | 0·0783 | 0·5282 |
| 2 | ENSG00000184557 | SOCS3 | 0·3501 | 0·5275 |
| 2 | ENSG00000287656 | Antisense to SLC22A3 | 0·0726 | 0·5271 |
| 2 | ENSG00000170577 | SIX2 | 0·0784 | 0·5270 |
| 2 | ENSG00000137801 | THBS1 | 0·0703 | 0·5243 |
| 2 | ENSG00000185561 | TLCD2 | 0·1310 | 0·5236 |
| 2 | ENSG00000197405 | C5AR1 | 0·2152 | 0·5233 |
| 2 | ENSG00000249669 | CARMN | 0·0902 | 0·5231 |
| 2 | ENSG00000182885 | ADGRG3 | 0·0785 | 0·5222 |
| 2 | ENSG00000163623 | NKX6-1 | 0·0762 | 0·5212 |
| 2 | ENSG00000163464 | CXCR1 | 0·0771 | 0·5210 |
| 2 | ENSG00000267304 | Lnc-TJP3-2 | 0·0679 | 0·5210 |
| 2 | ENSG00000267065 | LINC02080 | 0·0687 | 0·5208 |
| 2 | ENSG00000258676 | Lnc-ST8SIA2-1 | 0·0683 | 0·5202 |
| 2 | ENSG00000130600 | H19 | 0·0842 | 0·5194 |
| 2 | ENSG00000160593 | JAML | 0·0916 | 0·5190 |
| 2 | ENSG00000141574 | SECTM1 | 0·1016 | 0·5185 |
| 2 | ENSG00000251493 | FOXD1 | 0·0706 | 0·5185 |
| 2 | ENSG00000228133 | LOC105371485 | 0·0710 | 0·5178 |
| 2 | ENSG00000074047 | GLI2 | 0·0794 | 0·5177 |
| 2 | ENSG00000262003 | LOC101927727 | 0·0716 | 0·5171 |
| 2 | ENSG00000254362 | Lnc-PPP2R2A-2 | 0·0734 | 0·5165 |
| 2 | ENSG00000198542 | ITGBL1 | 0·0744 | 0·5158 |
| 2 | ENSG00000150048 | CLEC1A | 0·1016 | 0·5149 |
| 2 | ENSG00000197901 | SLC22A6 | 0·1006 | 0·5148 |
| 2 | ENSG00000188511 | MIR3667HG | 0·0821 | 0·5145 |
| 2 | ENSG00000145623 | OSMR | 0·1585 | 0·5141 |
| 2 | ENSG00000163395 | IGFN1 | 0·1503 | 0·5121 |
| 2 | ENSG00000117318 | ID3 | 0·1579 | 0·5119 |
| 2 | ENSG00000270640 | Lnc-BABAM2-2 | 0·0681 | 0·5117 |
| 2 | ENSG00000140839 | CLEC18B | 0·0926 | 0·5105 |
| 2 | ENSG00000168404 | MLKL | 0·1351 | 0·5098 |
| 2 | ENSG00000125144 | MT1G | 0·0702 | 0·5098 |
| 2 | ENSG00000169894 | MUC3A | 0·0727 | 0·5086 |
| 2 | ENSG00000170801 | HTRA3 | 0·0950 | 0·5082 |
| 2 | ENSG00000171049 | FPR2 | 0·0841 | 0·5080 |
| 2 | ENSG00000148926 | ADM | 0·1033 | 0·5077 |
| 2 | ENSG00000000938 | FGR | 0·1132 | 0·5075 |
| 2 | ENSG00000279320 | None | 0·0784 | 0·5070 |
| 2 | ENSG00000213719 | CLIC1 | 0·1690 | 0·5068 |
| 2 | ENSG00000169908 | TM4SF1 | 0·1260 | 0·5067 |
| 2 | ENSG00000108823 | SGCA | 0·0927 | 0·5051 |
| 2 | ENSG00000205592 | MUC19 | 0·0809 | 0·5050 |
| 2 | ENSG00000112299 | VNN1 | 0·1115 | 0·5050 |
| 2 | ENSG00000134531 | EMP1 | 0·1952 | 0·5048 |
| 2 | ENSG00000136732 | GYPC | 0·1909 | 0·5046 |
| 2 | ENSG00000256443 | Lnc-CPSF7-3 | 0·0683 | 0·5023 |
| 2 | ENSG00000162692 | VCAM1 | 0·1084 | 0·5019 |
| 2 | ENSG00000123374 | CDK2 | 0·1392 | 0·4990 |
| 2 | ENSG00000187634 | SAMD11 | 0·1889 | 0·4974 |
| 2 | ENSG00000205364 | MT1M | 0·0792 | 0·4971 |
| 2 | ENSG00000142089 | IFITM3 | 0·1127 | 0·4960 |
| 2 | ENSG00000205358 | MT1H | 0·0677 | 0·4953 |
| 2 | ENSG00000280109 | PLAC4 | 0·0771 | 0·4950 |
| 2 | ENSG00000125731 | SH2D3A | 0·0905 | 0·4942 |
| 2 | ENSG00000235750 | KIAA0040 | 0·1544 | 0·4933 |
| 2 | ENSG00000268388 | FENDRR | 0·0698 | 0·4933 |
| 2 | ENSG00000115165 | CYTIP | 0·1310 | 0·4923 |
| 2 | ENSG00000152977 | ZIC1 | 0·0967 | 0·4917 |
| 2 | ENSG00000159167 | STC1 | 0·2229 | 0·4904 |
| 2 | ENSG00000185885 | IFITM1 | 0·1422 | 0·4897 |
| 2 | ENSG00000100060 | MFNG | 0·1947 | 0·4889 |
| 2 | ENSG00000132357 | CARD6 | 0·1124 | 0·4887 |
| 2 | ENSG00000145779 | TNFAIP8 | 0·1175 | 0·4887 |
| 2 | ENSG00000183615 | FAM167B | 0·1053 | 0·4886 |
| 2 | ENSG00000124549 | BTN2A3P | 0·1080 | 0·4876 |
| 2 | ENSG00000110719 | TCIRG1 | 0·1600 | 0·4860 |
| 2 | ENSG00000276412 | Lnc-CNTNAP3B-13 | 0·0763 | 0·4851 |
| 2 | ENSG00000028137 | TNFRSF1B | 0·2047 | 0·4815 |
| 2 | ENSG00000281327 | LINC01338 | 0·1457 | 0·4808 |
| 2 | ENSG00000185499 | MUC1 | 0·0847 | 0·4793 |
| 2 | ENSG00000186431 | FCAR | 0·0737 | 0·4782 |
| 2 | ENSG00000105559 | PLEKHA4 | 0·1353 | 0·4772 |
| 2 | ENSG00000118503 | TNFAIP3 | 0·0913 | 0·4767 |
| 2 | ENSG00000231246 | LINC02884 | 0·0746 | 0·4754 |
| 2 | ENSG00000160111 | CPAMD8 | 0·1501 | 0·4751 |
| 2 | ENSG00000070729 | CNGB1 | 0·1294 | 0·4748 |
| 2 | ENSG00000011422 | PLAUR | 0·1129 | 0·4747 |
| 2 | ENSG00000236908 | LINC02827 | 0·0676 | 0·4702 |
| 2 | ENSG00000176046 | NUPR1 | 0·1310 | 0·4701 |
| 2 | ENSG00000090339 | ICAM1 | 0·1558 | 0·4689 |
| 2 | ENSG00000105974 | CAV1 | 0·0781 | 0·4680 |
| 2 | ENSG00000100504 | PYGL | 0·0952 | 0·4621 |
| 2 | ENSG00000166592 | RRAD | 0·0733 | 0·4614 |
| 2 | ENSG00000103710 | RASL12 | 0·1499 | 0·4578 |
| 2 | ENSG00000128016 | ZFP36 | 0·1257 | 0·4530 |
| 2 | ENSG00000170345 | FOS | 0·0876 | 0·4526 |
| 2 | ENSG00000167772 | ANGPTL4 | 0·1161 | 0·4514 |
| 2 | ENSG00000142102 | PGGHG | 0·0681 | 0·4497 |
| 2 | ENSG00000175591 | P2RY2 | 0·0895 | 0·4371 |
| 2 | ENSG00000020633 | RUNX3 | 0·0743 | 0·4361 |
| 2 | ENSG00000156463 | SH3RF2 | 0·0891 | 0·4310 |
| 2 | ENSG00000287689 | None | 0·0712 | 0·4284 |
| 2 | ENSG00000154188 | ANGPT1 | 0·0687 | 0·4277 |
| 3 | ENSG00000064886 | CHI3L2 | 0·4444 | 0·7458 |
| 3 | ENSG00000006747 | SCIN | 0·4155 | 0·6644 |
| 3 | ENSG00000095970 | TREM2 | 0·2306 | 0·6507 |
| 3 | ENSG00000038945 | MSR1 | 0·3232 | 0·6468 |
| 3 | ENSG00000118785 | SPP1 | 0·1842 | 0·6451 |
| 3 | ENSG00000224189 | HAGLR | 0·2821 | 0·6409 |
| 3 | ENSG00000012223 | LTF | 0·1864 | 0·6378 |
| 3 | ENSG00000163823 | CCR1 | 0·2533 | 0·6327 |
| 3 | ENSG00000196136 | SERPINA3 | 0·3858 | 0·6323 |
| 3 | ENSG00000173391 | OLR1 | 0·1675 | 0·6306 |
| 3 | ENSG00000026508 | CD44 | 0·3408 | 0·6300 |
| 3 | ENSG00000203747 | FCGR3A | 0·2844 | 0·6260 |
| 3 | ENSG00000150337 | FCGR1A | 0·1995 | 0·6243 |
| 3 | ENSG00000066294 | CD84 | 0·1908 | 0·6191 |
| 3 | ENSG00000168329 | CX3CR1 | 0·1554 | 0·6083 |
| 3 | ENSG00000173369 | C1QB | 0·3041 | 0·6062 |
| 3 | ENSG00000005844 | ITGAL | 0·2456 | 0·6054 |
| 3 | ENSG00000155307 | SAMSN1 | 0·2182 | 0·6042 |
| 3 | ENSG00000108691 | CCL2 | 0·2850 | 0·6042 |
| 3 | ENSG00000240583 | AQP1 | 0·2052 | 0·6041 |
| 3 | ENSG00000166927 | MS4A7 | 0·2270 | 0·6031 |
| 3 | ENSG00000185811 | IKZF1 | 0·2204 | 0·6021 |
| 3 | ENSG00000148773 | MKI67 | 0·3404 | 0·6020 |
| 3 | ENSG00000077420 | APBB1IP | 0·1666 | 0·5975 |
| 3 | ENSG00000158869 | FCER1G | 0·2290 | 0·5966 |
| 3 | ENSG00000204287 | HLA-DRA | 0·1752 | 0·5957 |
| 3 | ENSG00000011600 | TYROBP | 0·1881 | 0·5945 |
| 3 | ENSG00000082074 | FYB1 | 0·2092 | 0·5931 |
| 3 | ENSG00000167613 | LAIR1 | 0·2646 | 0·5931 |
| 3 | ENSG00000105697 | HAMP | 0·1858 | 0·5916 |
| 3 | ENSG00000258342 | LINC00609 | 0·2780 | 0·5912 |
| 3 | ENSG00000239961 | LILRA4 | 0·1370 | 0·5882 |
| 3 | ENSG00000160255 | ITGB2 | 0·2669 | 0·5882 |
| 3 | ENSG00000104972 | LILRB1 | 0·2167 | 0·5874 |
| 3 | ENSG00000125730 | C3 | 0·1568 | 0·5841 |
| 3 | ENSG00000159189 | C1QC | 0·2604 | 0·5840 |
| 3 | ENSG00000162511 | LAPTM5 | 0·1968 | 0·5834 |
| 3 | ENSG00000134061 | CD180 | 0·2625 | 0·5832 |
| 3 | ENSG00000140968 | IRF8 | 0·1710 | 0·5831 |
| 3 | ENSG00000018280 | SLC11A1 | 0·2388 | 0·5819 |
| 3 | ENSG00000271605 | MILR1 | 0·1310 | 0·5815 |
| 3 | ENSG00000204472 | AIF1 | 0·1678 | 0·5814 |
| 3 | ENSG00000129450 | SIGLEC9 | 0·2089 | 0·5802 |
| 3 | ENSG00000239998 | LILRA2 | 0·1916 | 0·5801 |
| 3 | ENSG00000242574 | HLA-DMB | 0·1448 | 0·5797 |
| 3 | ENSG00000184574 | LPAR5 | 0·1204 | 0·5796 |
| 3 | ENSG00000173988 | LRRC63 | 0·1636 | 0·5787 |
| 3 | ENSG00000226496 | LINC00323 | 0·2143 | 0·5778 |
| 3 | ENSG00000257585 | LINC00609 | 0·1563 | 0·5776 |
| 3 | ENSG00000257826 | Lnc-MBIP-3 | 0·2340 | 0·5772 |
| 3 | ENSG00000276980 | Lnc-GPR108-3 | 0·1544 | 0·5759 |
| 3 | ENSG00000132965 | ALOX5AP | 0·2384 | 0·5757 |
| 3 | ENSG00000165025 | SYK | 0·1635 | 0·5747 |
| 3 | ENSG00000121933 | TMIGD3 | 0·1161 | 0·5733 |
| 3 | ENSG00000131747 | TOP2A | 0·2065 | 0·5730 |
| 3 | ENSG00000233670 | PIRT | 0·1656 | 0·5724 |
| 3 | ENSG00000250771 | TLR2 Pseudogene | 0·1736 | 0·5723 |
| 3 | ENSG00000115956 | PLEK | 0·1586 | 0·5708 |
| 3 | ENSG00000100292 | HMOX1 | 0·2271 | 0·5693 |
| 3 | ENSG00000167755 | KLK6 | 0·3529 | 0·5686 |
| 3 | ENSG00000173372 | C1QA | 0·2941 | 0·5682 |
| 3 | ENSG00000158715 | SLC45A3 | 0·1833 | 0·5679 |
| 3 | ENSG00000155629 | PIK3AP1 | 0·2317 | 0·5677 |
| 3 | ENSG00000086205 | FOLH1 | 0·3113 | 0·5675 |
| 3 | ENSG00000261795 | Lnc-PDGFA-4 | 0·1338 | 0·5672 |
| 3 | ENSG00000204252 | HLA-DOA | 0·1157 | 0·5669 |
| 3 | ENSG00000175567 | UCP2 | 0·1424 | 0·5666 |
| 3 | ENSG00000204161 | TMEM273 | 0·1158 | 0·5659 |
| 3 | ENSG00000175785 | PRIMA1 | 0·1161 | 0·5650 |
| 3 | ENSG00000265531 | FCGR1CP | 0·1028 | 0·5650 |
| 3 | ENSG00000253924 | Lnc-NPBWR1-8 | 0·2240 | 0·5648 |
| 3 | ENSG00000167851 | CD300A | 0·2093 | 0·5647 |
| 3 | ENSG00000228392 | None | 0·2104 | 0·5645 |
| 3 | ENSG00000169403 | PTAFR | 0·1752 | 0·5639 |
| 3 | ENSG00000105366 | SIGLEC8 | 0·1177 | 0·5639 |
| 3 | ENSG00000269553 | Lnc-MAG-2 | 0·1194 | 0·5636 |
| 3 | ENSG00000016602 | CLCA4 | 0·1681 | 0·5630 |
| 3 | ENSG00000013297 | CLDN11 | 0·1882 | 0·5627 |
| 3 | ENSG00000186417 | GLDN | 0·2349 | 0·5612 |
| 3 | ENSG00000259104 | PTCSC3 | 0·1650 | 0·5609 |
| 3 | ENSG00000105967 | TFEC | 0·1231 | 0·5609 |
| 3 | ENSG00000110077 | MS4A6A | 0·3586 | 0·5605 |
| 3 | ENSG00000232310 | LINC03002 | 0·1724 | 0·5599 |
| 3 | ENSG00000158865 | SLC5A11 | 0·2242 | 0·5596 |
| 3 | ENSG00000101336 | HCK | 0·1635 | 0·5594 |
| 3 | ENSG00000090104 | RGS1 | 0·1631 | 0·5578 |
| 3 | ENSG00000041982 | TNC | 0·0966 | 0·5564 |
| 3 | ENSG00000255216 | Lnc-TRIM29-2 | 0·2158 | 0·5560 |
| 3 | ENSG00000138964 | PARVG | 0·1641 | 0·5553 |
| 3 | ENSG00000171860 | C3AR1 | 0·1675 | 0·5548 |
| 3 | ENSG00000275395 | FCGBP | 0·3690 | 0·5546 |
| 3 | ENSG00000204655 | MOG | 0·2646 | 0·5546 |
| 3 | ENSG00000197249 | SERPINA1 | 0·3593 | 0·5541 |
| 3 | ENSG00000187908 | DMBT1 | 0·2145 | 0·5540 |
| 3 | ENSG00000254314 | Lnc-NPBWR1-9 | 0·1663 | 0·5539 |
| 3 | ENSG00000081237 | PTPRC | 0·1697 | 0·5526 |
| 3 | ENSG00000231389 | HLA-DPA1 | 0·1207 | 0·5513 |
| 3 | ENSG00000250072 | SH3TC2-DT | 0·2859 | 0·5506 |
| 3 | ENSG00000154864 | PIEZO2 | 0·1379 | 0·5501 |
| 3 | ENSG00000134817 | APLNR | 0·1656 | 0·5494 |
| 3 | ENSG00000139292 | LGR5 | 0·1987 | 0·5487 |
| 3 | ENSG00000183160 | TMEM119 | 0·0974 | 0·5479 |
| 3 | ENSG00000225781 | OR6V1 | 0·1580 | 0·5472 |
| 3 | ENSG00000152689 | RASGRP3 | 0·1192 | 0·5470 |
| 3 | ENSG00000251138 | LINC02882 | 0·3034 | 0·5470 |
| 3 | ENSG00000174607 | UGT8 | 0·2070 | 0·5466 |
| 3 | ENSG00000197471 | SPN | 0·1518 | 0·5465 |
| 3 | ENSG00000267432 | DNAH17-AS1 | 0·2638 | 0·5465 |
| 3 | ENSG00000213186 | TRIM59 | 0·2357 | 0·5465 |
| 3 | ENSG00000071991 | CDH19 | 0·1692 | 0·5465 |
| 3 | ENSG00000171051 | FPR1 | 0·1661 | 0·5464 |
| 3 | ENSG00000136167 | LCP1 | 0·2126 | 0·5462 |
| 3 | ENSG00000228789 | HCG22 | 0·1308 | 0·5458 |
| 3 | ENSG00000142583 | SLC2A5 | 0·1205 | 0·5456 |
| 3 | ENSG00000277494 | GPIHBP1 | 0·1423 | 0·5446 |
| 3 | ENSG00000286558 | None | 0·2273 | 0·5445 |
| 3 | ENSG00000114013 | CD86 | 0·1466 | 0·5440 |
| 3 | ENSG00000081479 | LRP2 | 0·1365 | 0·5439 |
| 3 | ENSG00000148908 | RGS10 | 0·0901 | 0·5436 |
| 3 | ENSG00000042493 | CAPG | 0·1489 | 0·5434 |
| 3 | ENSG00000105383 | CD33 | 0·1443 | 0·5428 |
| 3 | ENSG00000204482 | LST1 | 0·0920 | 0·5425 |
| 3 | ENSG00000205116 | TMEM88B | 0·1989 | 0·5423 |
| 3 | ENSG00000161896 | IP6K3 | 0·1400 | 0·5423 |
| 3 | ENSG00000231131 | LNCAROD | 0·1219 | 0·5419 |
| 3 | ENSG00000134516 | DOCK2 | 0·1101 | 0·5418 |
| 3 | ENSG00000104974 | LILRA1 | 0·0889 | 0·5414 |
| 3 | ENSG00000207955 | MIR219A2HG | 0·3251 | 0·5414 |
| 3 | ENSG00000126860 | EVI2A | 0·1890 | 0·5402 |
| 3 | ENSG00000268758 | ADGRE4P | 0·0917 | 0·5401 |
| 3 | ENSG00000279419 | None | 0·2489 | 0·5400 |
| 3 | ENSG00000244062 | ACSL3 Pseudogene | 0·2810 | 0·5397 |
| 3 | ENSG00000261121 | LINC02473 | 0·1756 | 0·5397 |
| 3 | ENSG00000143226 | FCGR2A | 0·2433 | 0·5395 |
| 3 | ENSG00000172243 | CLEC7A | 0·0942 | 0·5394 |
| 3 | ENSG00000080031 | PTPRH | 0·1945 | 0·5393 |
| 3 | ENSG00000019582 | CD74 | 0·1044 | 0·5391 |
| 3 | ENSG00000103089 | FA2H | 0·2255 | 0·5390 |
| 3 | ENSG00000166091 | CMTM5 | 0·2567 | 0·5389 |
| 3 | ENSG00000198502 | HLA-DRB5 | 0·1264 | 0·5383 |
| 3 | ENSG00000170458 | CD14 | 0·2002 | 0·5383 |
| 3 | ENSG00000127412 | TRPV5 | 0·1333 | 0·5382 |
| 3 | ENSG00000148826 | NKX6-2 | 0·0973 | 0·5366 |
| 3 | ENSG00000105695 | MAG | 0·2540 | 0·5362 |
| 3 | ENSG00000188488 | SERPINA5 | 0·1245 | 0·5360 |
| 3 | ENSG00000112799 | LY86 | 0·1109 | 0·5355 |
| 3 | ENSG00000142512 | SIGLEC10 | 0·0913 | 0·5349 |
| 3 | ENSG00000160791 | CCR5 | 0·1243 | 0·5345 |
| 3 | ENSG00000261997 | None | 0·0881 | 0·5342 |
| 3 | ENSG00000270547 | LINC01235 | 0·0773 | 0·5341 |
| 3 | ENSG00000197993 | KEL | 0·1067 | 0·5334 |
| 3 | ENSG00000179178 | TMEM125 | 0·3213 | 0·5328 |
| 3 | ENSG00000204644 | ZFP57 | 0·1625 | 0·5326 |
| 3 | ENSG00000139629 | GALNT6 | 0·1594 | 0·5326 |
| 3 | ENSG00000286279 | Lnc-SLC16A7-4 | 0·2188 | 0·5319 |
| 3 | ENSG00000251429 | AIDAP2 | 0·1537 | 0·5318 |
| 3 | ENSG00000012124 | CD22 | 0·1227 | 0·5311 |
| 3 | ENSG00000140030 | GPR65 | 0·1490 | 0·5307 |
| 3 | ENSG00000180739 | S1PR5 | 0·1704 | 0·5297 |
| 3 | ENSG00000163694 | RBM47 | 0·2062 | 0·5297 |
| 3 | ENSG00000228058 | LINC01736 | 0·0948 | 0·5295 |
| 3 | ENSG00000204278 | TMEM235 | 0·1819 | 0·5292 |
| 3 | ENSG00000198019 | FCGR1B | 0·0741 | 0·5287 |
| 3 | ENSG00000172578 | KLHL6 | 0·2254 | 0·5286 |
| 3 | ENSG00000282608 | ADORA3 | 0·1216 | 0·5285 |
| 3 | ENSG00000233828 | MIR4280HG | 0·2797 | 0·5284 |
| 3 | ENSG00000116774 | OLFML3 | 0·0919 | 0·5284 |
| 3 | ENSG00000250198 | LINC02199 | 0·1212 | 0·5280 |
| 3 | ENSG00000169896 | ITGAM | 0·1538 | 0·5278 |
| 3 | ENSG00000268170 | Lnc-FAM131B-2 | 0·1720 | 0·5275 |
| 3 | ENSG00000127507 | ADGRE2 | 0·0830 | 0·5275 |
| 3 | ENSG00000187912 | CLEC17A | 0·0857 | 0·5272 |
| 3 | ENSG00000287372 | None | 0·0722 | 0·5271 |
| 3 | ENSG00000286749 | Antisense to NMUR2 | 0·1255 | 0·5271 |
| 3 | ENSG00000158859 | ADAMTS4 | 0·1169 | 0·5270 |
| 3 | ENSG00000174125 | TLR1 | 0·1138 | 0·5270 |
| 3 | ENSG00000171840 | NINJ2 | 0·1958 | 0·5261 |
| 3 | ENSG00000272808 | GCAWKR | 0·2691 | 0·5258 |
| 3 | ENSG00000064300 | NGFR | 0·0736 | 0·5257 |
| 3 | ENSG00000126266 | FFAR1 | 0·1173 | 0·5257 |
| 3 | ENSG00000236398 | TAS2R39 | 0·0990 | 0·5254 |
| 3 | ENSG00000228408 | Lnc-TAB2-1 | 0·3106 | 0·5246 |
| 3 | ENSG00000130300 | PLVAP | 0·0702 | 0·5246 |
| 3 | ENSG00000086730 | LAT2 | 0·1724 | 0·5242 |
| 3 | ENSG00000167641 | PPP1R14A | 0·1141 | 0·5234 |
| 3 | ENSG00000184357 | H1-5 | 0·2146 | 0·5231 |
| 3 | ENSG00000133321 | PLAAT4 | 0·1197 | 0·5230 |
| 3 | ENSG00000231427 | LINC01445 | 0·1206 | 0·5228 |
| 3 | ENSG00000162444 | RBP7 | 0·2111 | 0·5224 |
| 3 | ENSG00000159216 | RUNX1 | 0·1434 | 0·5221 |
| 3 | ENSG00000110934 | BIN2 | 0·1052 | 0·5219 |
| 3 | ENSG00000174123 | TLR10 | 0·0753 | 0·5211 |
| 3 | ENSG00000136960 | ENPP2 | 0·2141 | 0·5210 |
| 3 | ENSG00000254415 | SIGLEC14 | 0·0754 | 0·5208 |
| 3 | ENSG00000011426 | ANLN | 0·1141 | 0·5207 |
| 3 | ENSG00000287939 | None | 0·0793 | 0·5203 |
| 3 | ENSG00000241345 | LOC105375483 | 0·1202 | 0·5200 |
| 3 | ENSG00000169413 | RNASE6 | 0·0759 | 0·5195 |
| 3 | ENSG00000178082 | TWF1P1 | 0·1148 | 0·5192 |
| 3 | ENSG00000124915 | MYRF-AS1 | 0·0889 | 0·5184 |
| 3 | ENSG00000256618 | MTRNR2L1 | 0·1613 | 0·5184 |
| 3 | ENSG00000162949 | CAPN13 | 0·1591 | 0·5183 |
| 3 | ENSG00000249484 | LINC01470 | 0·1220 | 0·5183 |
| 3 | ENSG00000073737 | DHRS9 | 0·0817 | 0·5182 |
| 3 | ENSG00000146192 | FGD2 | 0·0924 | 0·5179 |
| 3 | ENSG00000244682 | FCGR2C | 0·1287 | 0·5177 |
| 3 | ENSG00000286403 | None | 0·1016 | 0·5177 |
| 3 | ENSG00000179468 | OR9A2 | 0·1493 | 0·5176 |
| 3 | ENSG00000101049 | SGK2 | 0·1796 | 0·5174 |
| 3 | ENSG00000051523 | CYBA | 0·1548 | 0·5162 |
| 3 | ENSG00000225217 | HSPA7 | 0·2140 | 0·5161 |
| 3 | ENSG00000183196 | CHST6 | 0·0768 | 0·5160 |
| 3 | ENSG00000180353 | HCLS1 | 0·1249 | 0·5154 |
| 3 | ENSG00000138316 | ADAMTS14 | 0·1382 | 0·5153 |
| 3 | ENSG00000175489 | LRRC25 | 0·1485 | 0·5151 |
| 3 | ENSG00000070190 | DAPP1 | 0·0724 | 0·5148 |
| 3 | ENSG00000107099 | DOCK8 | 0·0916 | 0·5146 |
| 3 | ENSG00000108798 | ABI3 | 0·0964 | 0·5144 |
| 3 | ENSG00000136867 | SLC31A2 | 0·1023 | 0·5144 |
| 3 | ENSG00000163191 | S100A11 | 0·3018 | 0·5140 |
| 3 | ENSG00000283608 | Lnc-SUMO4-6 | 0·1398 | 0·5131 |
| 3 | ENSG00000240534 | RPL34P17 | 0·2349 | 0·5130 |
| 3 | ENSG00000229391 | HLA-DRB6 | 0·0948 | 0·5126 |
| 3 | ENSG00000224389 | C4B | 0·2260 | 0·5119 |
| 3 | ENSG00000154642 | C21orf91 | 0·1296 | 0·5118 |
| 3 | ENSG00000180929 | GPR62 | 0·1292 | 0·5116 |
| 3 | ENSG00000125735 | TNFSF14 | 0·1245 | 0·5113 |
| 3 | ENSG00000167588 | GPD1 | 0·3039 | 0·5112 |
| 3 | ENSG00000137462 | TLR2 | 0·1196 | 0·5096 |
| 3 | ENSG00000262188 | LINC01978 | 0·1429 | 0·5092 |
| 3 | ENSG00000196126 | HLA-DRB1 | 0·0822 | 0·5089 |
| 3 | ENSG00000226448 | RPL7AP51 | 0·1549 | 0·5088 |
| 3 | ENSG00000184730 | APOBR | 0·1201 | 0·5084 |
| 3 | ENSG00000237166 | LINC01792 | 0·1283 | 0·5082 |
| 3 | ENSG00000227544 | LINC03013 | 0·1727 | 0·5080 |
| 3 | ENSG00000183760 | ACP7 | 0·1075 | 0·5079 |
| 3 | ENSG00000006042 | TMEM98 | 0·0994 | 0·5073 |
| 3 | ENSG00000235568 | NFAM1 | 0·1035 | 0·5073 |
| 3 | ENSG00000228793 | LOC100507336 | 0·0786 | 0·5072 |
| 3 | ENSG00000286522 | H3C2 | 0·1474 | 0·5070 |
| 3 | ENSG00000158714 | SLAMF8 | 0·2027 | 0·5063 |
| 3 | ENSG00000225924 | TAB2-AS1 | 0·0742 | 0·5062 |
| 3 | ENSG00000248208 | WDR45P1 | 0·0856 | 0·5059 |
| 3 | ENSG00000131401 | NAPSB | 0·1100 | 0·5053 |
| 3 | ENSG00000226994 | Lnc-RASGRP3-5 | 0·2097 | 0·5051 |
| 3 | ENSG00000159399 | HK2 | 0·1393 | 0·5048 |
| 3 | ENSG00000231840 | TMEM139-AS1 | 0·0943 | 0·5047 |
| 3 | ENSG00000166923 | GREM1 | 0·2348 | 0·5042 |
| 3 | ENSG00000128283 | CDC42EP1 | 0·0740 | 0·5041 |
| 3 | ENSG00000105122 | RASAL3 | 0·0911 | 0·5039 |
| 3 | ENSG00000167208 | SNX20 | 0·1051 | 0·5034 |
| 3 | ENSG00000150656 | CNDP1 | 0·2512 | 0·5034 |
| 3 | ENSG00000287609 | None | 0·0942 | 0·5025 |
| 3 | ENSG00000113396 | SLC27A6 | 0·1071 | 0·5021 |
| 3 | ENSG00000140379 | BCL2A1 | 0·1602 | 0·5020 |
| 3 | ENSG00000129757 | CDKN1C | 0·0953 | 0·5013 |
| 3 | ENSG00000283462 | LOC101930276 | 0·1841 | 0·5010 |
| 3 | ENSG00000169385 | RNASE2 | 0·0788 | 0·5005 |
| 3 | ENSG00000236700 | LINC01010 | 0·0749 | 0·5005 |
| 3 | ENSG00000136286 | MYO1G | 0·0689 | 0·5000 |
| 3 | ENSG00000221937 | TAS2R40 | 0·0786 | 0·4998 |
| 3 | ENSG00000176381 | PRR18 | 0·1319 | 0·4988 |
| 3 | ENSG00000137869 | CYP19A1 | 0·0816 | 0·4979 |
| 3 | ENSG00000173253 | DMRT2 | 0·1261 | 0·4975 |
| 3 | ENSG00000172005 | MAL | 0·1617 | 0·4968 |
| 3 | ENSG00000197430 | OPALIN | 0·2467 | 0·4965 |
| 3 | ENSG00000100365 | NCF4 | 0·1516 | 0·4964 |
| 3 | ENSG00000171631 | P2RY6 | 0·1131 | 0·4951 |
| 3 | ENSG00000119535 | CSF3R | 0·1157 | 0·4950 |
| 3 | ENSG00000232560 | LINC01549 | 0·0799 | 0·4948 |
| 3 | ENSG00000014257 | ACP3 | 0·0803 | 0·4948 |
| 3 | ENSG00000244301 | AOX3P | 0·1489 | 0·4939 |
| 3 | ENSG00000140873 | ADAMTS18 | 0·1981 | 0·4934 |
| 3 | ENSG00000129667 | RHBDF2 | 0·1048 | 0·4932 |
| 3 | ENSG00000253877 | LINC01608 | 0·1697 | 0·4931 |
| 3 | ENSG00000172548 | NIPAL4 | 0·1423 | 0·4930 |
| 3 | ENSG00000128645 | HOXD1 | 0·1054 | 0·4924 |
| 3 | ENSG00000163565 | IFI16 | 0·1153 | 0·4920 |
| 3 | ENSG00000286625 | None | 0·1128 | 0·4916 |
| 3 | ENSG00000250215 | CIR1P2 | 0·2653 | 0·4913 |
| 3 | ENSG00000170775 | GPR37 | 0·1659 | 0·4912 |
| 3 | ENSG00000130592 | LSP1 | 0·1076 | 0·4910 |
| 3 | ENSG00000251442 | LINC01094 | 0·1477 | 0·4909 |
| 3 | ENSG00000110079 | MS4A4A | 0·2060 | 0·4905 |
| 3 | ENSG00000232474 | NCKAP5-IT1 | 0·0993 | 0·4901 |
| 3 | ENSG00000254081 | LINC01299 | 0·2365 | 0·4899 |
| 3 | ENSG00000152766 | ANKRD22 | 0·1144 | 0·4899 |
| 3 | ENSG00000221887 | HMSD | 0·1194 | 0·4898 |
| 3 | ENSG00000277117 | LOC102723996 | 0·0830 | 0·4898 |
| 3 | ENSG00000237082 | COX5BP6 | 0·1287 | 0·4893 |
| 3 | ENSG00000025708 | TYMP | 0·1410 | 0·4891 |
| 3 | ENSG00000012779 | ALOX5 | 0·1019 | 0·4890 |
| 3 | ENSG00000275713 | H2BC9 | 0·1015 | 0·4889 |
| 3 | ENSG00000122367 | LDB3 | 0·1561 | 0·4886 |
| 3 | ENSG00000165131 | LLCFC1 | 0·0803 | 0·4877 |
| 3 | ENSG00000285269 | SLC35D2-HSD17B3 readthrough | 0·0822 | 0·4877 |
| 3 | ENSG00000227088 | Lnc-LRRTM4-1 | 0·1546 | 0·4877 |
| 3 | ENSG00000252312 | RN7SKP21 | 0·0857 | 0·4874 |
| 3 | ENSG00000179420 | OR6W1P | 0·1194 | 0·4869 |
| 3 | ENSG00000258948 | KRT8P1 | 0·0795 | 0·4866 |
| 3 | ENSG00000198774 | RASSF9 | 0·1114 | 0·4862 |
| 3 | ENSG00000288533 | Antisense to TYMS and CLUL1 | 0·1653 | 0·4860 |
| 3 | ENSG00000127324 | TSPAN8 | 0·0709 | 0·4858 |
| 3 | ENSG00000254863 | LINC02744 | 0·1007 | 0·4851 |
| 3 | ENSG00000279223 | None | 0·0911 | 0·4846 |
| 3 | ENSG00000285712 | None | 0·1039 | 0·4836 |
| 3 | ENSG00000128652 | HOXD3 | 0·0765 | 0·4825 |
| 3 | ENSG00000231171 | LINC01098 | 0·1112 | 0·4816 |
| 3 | ENSG00000229140 | CCDC26 | 0·0901 | 0·4812 |
| 3 | ENSG00000155926 | SLA | 0·1026 | 0·4811 |
| 3 | ENSG00000133048 | CHI3L1 | 0·1678 | 0·4809 |
| 3 | ENSG00000164342 | TLR3 | 0·0717 | 0·4808 |
| 3 | ENSG00000273259 | Thioesterase | 0·1471 | 0·4808 |
| 3 | ENSG00000286569 | None | 0·0794 | 0·4806 |
| 3 | ENSG00000157227 | MMP14 | 0·2251 | 0·4805 |
| 3 | ENSG00000257720 | ILF2P2 | 0·1511 | 0·4801 |
| 3 | ENSG00000100368 | CSF2RB | 0·1802 | 0·4799 |
| 3 | ENSG00000174837 | ADGRE1 | 0·0964 | 0·4799 |
| 3 | ENSG00000259764 | PCSK6-AS1 | 0·1262 | 0·4791 |
| 3 | ENSG00000244738 | Lnc-CLDN11-2 | 0·0942 | 0·4785 |
| 3 | ENSG00000160223 | ICOSLG | 0·0727 | 0·4773 |
| 3 | ENSG00000115523 | GNLY | 0·0901 | 0·4770 |
| 3 | ENSG00000244731 | C4A | 0·1582 | 0·4768 |
| 3 | ENSG00000118640 | VAMP8 | 0·1102 | 0·4767 |
| 3 | ENSG00000105281 | SLC1A5 | 0·1965 | 0·4765 |
| 3 | ENSG00000066336 | SPI1 | 0·1143 | 0·4761 |
| 3 | ENSG00000248529 | LOC101928651 | 0·1135 | 0·4756 |
| 3 | ENSG00000166928 | MS4A14 | 0·1456 | 0·4753 |
| 3 | ENSG00000166473 | PKD1L2 | 0·0930 | 0·4750 |
| 3 | ENSG00000163563 | MNDA | 0·1076 | 0·4747 |
| 3 | ENSG00000010327 | STAB1 | 0·2132 | 0·4740 |
| 3 | ENSG00000277775 | H3C7 | 0·0732 | 0·4734 |
| 3 | ENSG00000187554 | TLR5 | 0·0922 | 0·4718 |
| 3 | ENSG00000232504 | ST3GAL5-AS1 | 0·1183 | 0·4707 |
| 3 | ENSG00000132702 | HAPLN2 | 0·1061 | 0·4696 |
| 3 | ENSG00000129465 | RIPK3 | 0·0762 | 0·4676 |
| 3 | ENSG00000287538 | None | 0·1149 | 0·4666 |
| 3 | ENSG00000153551 | CMTM7 | 0·0872 | 0·4662 |
| 3 | ENSG00000205403 | CFI | 0·1287 | 0·4656 |
| 3 | ENSG00000207736 | MIR657 | 0·0843 | 0·4652 |
| 3 | ENSG00000137752 | CASP1 | 0·1240 | 0·4649 |
| 3 | ENSG00000128340 | RAC2 | 0·0997 | 0·4643 |
| 3 | ENSG00000287736 | PTCSC1 | 0·0953 | 0·4643 |
| 3 | ENSG00000145287 | PLAC8 | 0·1238 | 0·4637 |
| 3 | ENSG00000263154 | Lnc-BAHCC1-1 | 0·0776 | 0·4619 |
| 3 | ENSG00000203710 | CR1 | 0·0840 | 0·4616 |
| 3 | ENSG00000198835 | GJC2 | 0·0819 | 0·4607 |
| 3 | ENSG00000258227 | CLEC5A | 0·0896 | 0·4606 |
| 3 | ENSG00000090382 | LYZ | 0·1161 | 0·4600 |
| 3 | ENSG00000260922 | Antisense to ADAMTS18 | 0·0977 | 0·4583 |
| 3 | ENSG00000285885 | Antisense to MOBP | 0·0999 | 0·4581 |
| 3 | ENSG00000214688 | C10orf105 | 0·0833 | 0·4551 |
| 3 | ENSG00000286048 | Antisense to ITGA2 | 0·0949 | 0·4543 |
| 3 | ENSG00000229494 | LOC101927948 | 0·1134 | 0·4542 |
| 3 | ENSG00000197747 | S100A10 | 0·1026 | 0·4542 |
| 3 | ENSG00000136235 | GPNMB | 0·0836 | 0·4536 |
| 3 | ENSG00000267287 | Lnc-NFATC1-1 | 0·0916 | 0·4532 |
| 3 | ENSG00000185905 | C16orf54 | 0·0831 | 0·4531 |
| 3 | ENSG00000257183 | Lnc-KCNC2-2 | 0·1148 | 0·4526 |
| 3 | ENSG00000152804 | HHEX | 0·0788 | 0·4524 |
| 3 | ENSG00000129810 | SGO1 | 0·0700 | 0·4517 |
| 3 | ENSG00000132854 | KANK4 | 0·0883 | 0·4501 |
| 3 | ENSG00000286541 | None | 0·0795 | 0·4495 |
| 3 | ENSG00000283431 | Novel Zinc Finger Protein Pseudogene | 0·0805 | 0·4477 |
| 3 | ENSG00000117228 | GBP1 | 0·0698 | 0·4451 |
| 3 | ENSG00000122585 | NPY | 0·0969 | 0·4421 |
| 3 | ENSG00000101670 | LIPG | 0·0778 | 0·4418 |
| 3 | ENSG00000224397 | PELATON | 0·0792 | 0·4413 |
| 3 | ENSG00000261710 | Lnc-ADGRB1-3 | 0·0753 | 0·4369 |
| 3 | ENSG00000141526 | SLC16A3 | 0·0949 | 0·4368 |
| 3 | ENSG00000128262 | POM121L9P | 0·0709 | 0·4355 |
| 3 | ENSG00000283787 | PRR33 | 0·0741 | 0·4291 |

**Supplementary Table 3: Genetic Composition of KCL-BrainBank defined clusters.**

| **Source** | **Term Name** | **Term ID** | **Term Size** | **p-value adjusted** | **Query Size** | **Intersection Size** |
| --- | --- | --- | --- | --- | --- | --- |
| GO:MF | neuropeptide hormone activity | GO:0005184 | 22 | 9.90E-06 | 131 | 5 |
| GO:MF | hormone activity | GO:0005179 | 78 | 1.10E-05 | 131 | 7 |
| GO:BP | chemical synaptic transmission | GO:0007268 | 663 | 4.00E-04 | 131 | 14 |
| GO:BP | anterograde trans-synaptic signaling | GO:0098916 | 663 | 4.00E-04 | 131 | 14 |
| GO:BP | trans-synaptic signaling | GO:0099537 | 671 | 4.60E-04 | 131 | 14 |
| REAC | MECP2 regulates transcription of neuronal ligands | REAC:R-HSA-9022702 | 7 | 6.30E-04 | 131 | 3 |
| GO:BP | synaptic signaling | GO:0099536 | 694 | 6.90E-04 | 131 | 14 |
| GO:BP | regulation of epinephrine secretion | GO:0014060 | 7 | 3.60E-03 | 131 | 3 |
| GO:BP | epinephrine secretion | GO:0048242 | 8 | 5.70E-03 | 131 | 3 |
| KEGG | cAMP signaling pathway | KEGG:04024 | 203 | 8.00E-03 | 131 | 6 |
| KEGG | neuroactive ligand-receptor interaction | KEGG:04080 | 301 | 9.60E-03 | 131 | 7 |
| GO:BP | epinephrine transport | GO:0048241 | 11 | 1.70E-02 | 131 | 3 |
| GO:BP | mating | GO:0007618 | 35 | 1.80E-02 | 131 | 4 |
| GO:CC | neuronal cell body | GO:0043025 | 471 | 2.10E-02 | 131 | 9 |
| REAC | Transcriptional Regulation by MECP2 | REAC:R-HSA-8986944 | 58 | 2.40E-02 | 131 | 4 |
| GO:BP | positive regulation of epinephrine secretion | GO:0032812 | 2 | 2.80E-02 | 131 | 2 |
| GO:MF | receptor ligand activity | GO:0048018 | 356 | 3.50E-02 | 131 | 8 |
| TF | NRSF TTCAGCACCACGGACAGMGCC | TF:M00256_1 | 13 | 3.70E-02 | 131 | 3 |
| REAC | GPCR ligand binding | REAC:R-HSA-500792 | 388 | 3.90E-02 | 131 | 8 |
| GO:MF | signaling receptor activator activity | GO:0030546 | 363 | 4.10E-02 | 131 | 8 |
| GO:BP | neuropeptide signaling pathway | GO:0007218 | 89 | 4.40E-02 | 131 | 5 |

**Supplementary Table 4: KCL BrainBank enrichment for Cluster 1.** GO:BP – Gene Ontology Biological Process, GO:CC – Gene Ontology Cellular Components, GO:MF – Gene Ontology Molecular Function, KEGG: Kyoto Encyclopaedia of Genes and Genomes, REAC: Reactome, TF: Transfac.

| **Source** | **Term Name** | **Term ID** | **Term Size** | **p-value adjusted** | **Query Size** | **Intersection Size** |
| --- | --- | --- | --- | --- | --- | --- |
| GO:CC | cell periphery | GO:0071944 | 5188 | 1.70E-39 | 291 | 140 |
| GO:BP | response to stimulus | GO:0050896 | 7482 | 7.30E-37 | 291 | 165 |
| GO:CC | extracellular region | GO:0005576 | 3516 | 7.40E-34 | 291 | 109 |
| GO:BP | biological regulation | GO:0065007 | 10837 | 1.60E-33 | 291 | 194 |
| TF | Factor: Kid3; motif: CCACN | TF:M01160 | 17056 | 3.70E-31 | 291 | 241 |
| TF | Factor: Spi-B; motif: TTCYBC | TF:M03851 | 17110 | 6.80E-31 | 291 | 241 |
| TF | Transfac | TF:M00000 | 17114 | 7.10E-31 | 291 | 241 |
| GO:BP | response to chemical | GO:0042221 | 3698 | 2.30E-30 | 291 | 108 |
| TF | Factor: Spi-B; motif: TTCYBC; match class: 1 | TF:M03851_1 | 17095 | 2.90E-30 | 291 | 240 |
| GO:BP | cellular response to chemical stimulus | GO:0070887 | 2745 | 4.70E-30 | 291 | 93 |
| GO:CC | extracellular space | GO:0005615 | 2749 | 4.70E-30 | 291 | 92 |
| TF | Factor: CPBP; motif: SNCCCNN | TF:M01822 | 16885 | 6.80E-30 | 291 | 238 |
| TF | Factor: Kid3; motif: CCACN; match class: 1 | TF:M01160_1 | 16941 | 1.30E-29 | 291 | 238 |
| TF | Factor: PU.1; motif: AGGAAG | TF:M02031 | 15486 | 2.10E-29 | 291 | 227 |
| TF | Factor: Elf-1; motif: AGGAAG | TF:M01266 | 15486 | 2.10E-29 | 291 | 227 |
| GO:BP | regulation of biological process | GO:0050789 | 10213 | 5.60E-29 | 291 | 181 |
| TF | Factor: ZNF35; motif: SSAAKA | TF:M07479 | 16059 | 1.50E-28 | 291 | 230 |
| GO:MF | molecular_function | GO:0003674 | 16562 | 5.10E-28 | 291 | 232 |
| TF | Factor: GKLF; motif: CCTCCYN | TF:M01835 | 13756 | 6.60E-28 | 291 | 211 |
| GO:CC | cellular anatomical entity | GO:0110165 | 16869 | 1.30E-27 | 291 | 233 |
| GO:BP | biological_process | GO:0008150 | 16323 | 1.80E-27 | 291 | 230 |
| GO:CC | cellular_component | GO:0005575 | 16974 | 4.00E-27 | 291 | 233 |
| GO:BP | response to stress | GO:0006950 | 3345 | 6.10E-27 | 291 | 98 |
| GO:BP | response to organic substance | GO:0010033 | 2758 | 6.40E-27 | 291 | 89 |
| GO:BP | cellular response to stimulus | GO:0051716 | 6392 | 5.00E-26 | 291 | 136 |
| GO:MF | binding | GO:0005488 | 15390 | 5.10E-26 | 291 | 220 |
| GO:BP | multicellular organismal process | GO:0032501 | 6431 | 9.30E-26 | 291 | 136 |
| GO:BP | tissue development | GO:0009888 | 1688 | 1.10E-25 | 291 | 69 |
| TF | Factor: CPBP; motif: SNCCCNN; match class: 1 | TF:M01822_1 | 16464 | 2.50E-25 | 291 | 228 |
| TF | Factor: ZNF684; motif: MAAGGGGTGGACTGT | TF:M10550 | 13426 | 2.50E-25 | 291 | 204 |
| GO:MF | protein binding | GO:0005515 | 13368 | 4.80E-25 | 291 | 202 |
| GO:CC | plasma membrane | GO:0005886 | 4761 | 7.20E-25 | 291 | 113 |
| TF | Factor: Lyl-1; motif: NCAKCTGYTNYCNN | TF:M09977 | 11924 | 9.50E-25 | 291 | 190 |
| TF | Factor: HSF4; motif: CTGCMRN | TF:M07322 | 14568 | 1.60E-24 | 291 | 212 |
| GO:BP | cellular response to organic substance | GO:0071310 | 2188 | 3.30E-24 | 291 | 76 |
| TF | Factor: TR4; motif: ACCCCGS | TF:M04934 | 15997 | 8.10E-24 | 291 | 222 |
| MIRNA | MIRNA root | MIRNA:000000 | 13878 | 8.10E-24 | 291 | 204 |
| GO:BP | cellular process | GO:0009987 | 15441 | 1.30E-23 | 291 | 217 |
| TF | Factor: Smad2; motif: AGACAN | TF:M03580 | 13070 | 1.30E-23 | 291 | 198 |
| TF | Factor: Elf-1; motif: AGGAAG; match class: 1 | TF:M01266_1 | 11933 | 1.40E-23 | 291 | 188 |
| TF | Factor: PU.1; motif: AGGAAG; match class: 1 | TF:M02031_1 | 11933 | 1.40E-23 | 291 | 188 |
| TF | Factor: Pax-4; motif: DTTTTCCACCN | TF:M04624 | 13570 | 1.80E-23 | 291 | 202 |
| GO:BP | regulation of cellular process | GO:0050794 | 9657 | 2.30E-23 | 291 | 166 |
| TF | Factor: Pax-4; motif: NNNNNYCACCCB | TF:M00378 | 15748 | 3.60E-23 | 291 | 219 |
| GO:BP | anatomical structure development | GO:0048856 | 5153 | 6.80E-23 | 291 | 116 |
| TF | Factor: GKLF; motif: NNRRGRRNGNSNNN | TF:M07040 | 11567 | 1.30E-22 | 291 | 183 |
| GO:BP | animal organ development | GO:0048513 | 3102 | 1.50E-22 | 291 | 88 |
| TF | Factor: THAP1; motif: YTGCCCNNA | TF:M07407 | 9580 | 1.80E-22 | 291 | 164 |
| TF | Factor: myogenin; motif: CAGCTG | TF:M02101 | 11284 | 2.20E-22 | 291 | 180 |
| TF | Factor: myogenin; motif: CAGCTG; match class: 1 | TF:M02101_1 | 11284 | 2.20E-22 | 291 | 180 |
| TF | Factor: Sox-18; motif: CAAWGBB | TF:M03848 | 15043 | 2.50E-22 | 291 | 212 |
| TF | Factor: Sox-17; motif: TTGTYY | TF:M03803 | 13366 | 3.30E-22 | 291 | 198 |
| GO:BP | positive regulation of biological process | GO:0048518 | 5512 | 4.40E-22 | 291 | 119 |
| TF | Factor: ER71:E2A; motif: CASSTGNACCGGAWRYN | TF:M08568 | 15994 | 4.40E-22 | 291 | 219 |
| GO:BP | circulatory system development | GO:0072359 | 1015 | 5.20E-22 | 291 | 51 |
| GO:BP | signaling | GO:0023052 | 5550 | 8.00E-22 | 291 | 119 |
| GO:BP | developmental process | GO:0032502 | 5648 | 1.00E-21 | 291 | 120 |
| TF | Factor: ZBTB39; motif: CNCTGY | TF:M12689 | 13477 | 1.10E-21 | 291 | 198 |
| GO:BP | cell communication | GO:0007154 | 5601 | 6.70E-21 | 291 | 118 |
| TF | Factor: Zbtb44; motif: CKGTGA | TF:M05405 | 11777 | 1.60E-20 | 291 | 181 |
| GO:BP | signal transduction | GO:0007165 | 5094 | 2.20E-20 | 291 | 111 |
| GO:CC | vesicle | GO:0031982 | 3591 | 3.20E-20 | 291 | 90 |
| TF | Factor: Erg; motif: NRRSAGGAAGNGG | TF:M09907 | 10225 | 3.30E-20 | 291 | 166 |
| GO:BP | system development | GO:0048731 | 4335 | 4.10E-20 | 291 | 101 |
| TF | Factor: T3R-beta; motif: NTGACCTYRNYRAGGTCAN | TF:M11818 | 10182 | 6.60E-20 | 291 | 165 |
| GO:CC | external encapsulating structure | GO:0030312 | 498 | 1.10E-19 | 291 | 35 |
| GO:BP | multicellular organism development | GO:0007275 | 4634 | 1.20E-19 | 291 | 104 |
| GO:BP | regulation of multicellular organismal process | GO:0051239 | 2403 | 1.40E-19 | 291 | 73 |
| TF | Factor: sin3A; motif: TGTCCNNGGTGCTG | TF:M04756 | 12573 | 1.80E-19 | 291 | 186 |
| TF | Factor: ING4; motif: CCACCA | TF:M01743 | 13898 | 2.80E-19 | 291 | 197 |
| TF | Factor: CTF/NF1; motif: TTGGCN | TF:M02050 | 9395 | 3.30E-19 | 291 | 156 |
| GO:BP | anatomical structure morphogenesis | GO:0009653 | 2447 | 3.90E-19 | 291 | 73 |
| GO:BP | cell adhesion | GO:0007155 | 1388 | 4.60E-19 | 291 | 55 |
| GO:CC | collagen-containing extracellular matrix | GO:0062023 | 383 | 4.70E-19 | 291 | 31 |
| GO:BP | cell surface receptor signaling pathway | GO:0007166 | 2456 | 4.80E-19 | 291 | 73 |
| GO:BP | negative regulation of biological process | GO:0048519 | 4967 | 5.30E-19 | 291 | 107 |
| GO:BP | biological adhesion | GO:0022610 | 1393 | 5.40E-19 | 291 | 55 |
| TF | Factor: Zic3; motif: NGGGKGGTC | TF:M00450 | 12380 | 8.40E-19 | 291 | 183 |
| GO:CC | extracellular matrix | GO:0031012 | 497 | 1.00E-18 | 291 | 34 |
| REAC | REACTOME root term | REAC:0000000 | 9607 | 1.60E-18 | 291 | 155 |
| GO:BP | anatomical structure formation involved in morphogenesis | GO:0048646 | 1026 | 2.20E-18 | 291 | 47 |
| TF | Factor: T3R-beta; motif: NTGACCTYRNYRAGGTCAN; match class: 1 | TF:M11818_1 | 9678 | 2.60E-18 | 291 | 157 |
| TF | Factor: GEMIN3; motif: NCWGGRARRGRGNGNG | TF:M09727 | 7710 | 2.90E-18 | 291 | 137 |
| GO:BP | blood vessel development | GO:0001568 | 641 | 3.90E-18 | 291 | 38 |
| GO:BP | immune system process | GO:0002376 | 2161 | 4.10E-18 | 291 | 67 |
| TF | Factor: ZNF644; motif: TCCWGCCTCTSN | TF:M09737 | 6371 | 4.90E-18 | 291 | 122 |
| TF | Factor: ZNF35; motif: SSAAKA; match class: 1 | TF:M07479_1 | 13256 | 5.40E-18 | 291 | 189 |
| TF | Factor: PUR1; motif: GGGNCAGNN | TF:M01721 | 8254 | 6.30E-18 | 291 | 142 |
| TF | Factor: SALL2; motif: GGGTGGG | TF:M04595 | 11697 | 7.40E-18 | 291 | 175 |
| TF | Factor: LF-A1; motif: GGGSTCWR | TF:M00646 | 11261 | 7.70E-18 | 291 | 171 |
| GO:BP | positive regulation of cellular process | GO:0048522 | 5061 | 8.10E-18 | 291 | 106 |
| TF | Factor: NF1C; motif: WGCCARR | TF:M09763 | 9428 | 1.50E-17 | 291 | 153 |
| TF | Factor: ETV4; motif: NCAGGAAGNN | TF:M12556 | 9336 | 1.70E-17 | 291 | 152 |
| GO:BP | vasculature development | GO:0001944 | 671 | 1.90E-17 | 291 | 38 |
| TF | Factor: Pax-4; motif: NNNNNYCACCCB; match class: 1 | TF:M00378_1 | 13163 | 2.10E-17 | 291 | 187 |
| KEGG | KEGG root term | KEGG:00000 | 7044 | 2.30E-17 | 291 | 125 |
| TF | Factor: ETF; motif: CCCCGCCCCYN | TF:M07039 | 14877 | 3.20E-17 | 291 | 201 |
| TF | Factor: BEN; motif: CAGCGRNV | TF:M01240 | 15004 | 3.30E-17 | 291 | 202 |
| TF | Factor: TR4; motif: ACCCCGS; match class: 1 | TF:M04934_1 | 13946 | 4.40E-17 | 291 | 193 |
| TF | Factor: Erg; motif: NACCGGATATCCGGTN | TF:M11393 | 12672 | 4.80E-17 | 291 | 182 |
| TF | Factor: LRH-1; motif: TGACCTTGRNYCAAGGTCA | TF:M11830 | 6126 | 7.20E-17 | 291 | 117 |
| TF | Factor: GKLF; motif: NNNRGGNGNGGSN | TF:M07289 | 13294 | 7.40E-17 | 291 | 187 |
| TF | Factor: E2F-1:HES-7; motif: GGCRCGTGSYNNWNGGCGCSM | TF:M08525 | 15473 | 7.90E-17 | 291 | 205 |
| TF | Factor: CSX; motif: NKRAGWGS | TF:M10008 | 9182 | 9.00E-17 | 291 | 149 |
| GO:BP | animal organ morphogenesis | GO:0009887 | 931 | 1.00E-16 | 291 | 43 |
| TF | Factor: RREB-1; motif: CCCCAAACMMCCCC | TF:M00257 | 9419 | 1.30E-16 | 291 | 151 |
| GO:BP | response to external stimulus | GO:0009605 | 2310 | 1.40E-16 | 291 | 67 |
| TF | Factor: NMYC; motif: CAYCTG | TF:M01808 | 10951 | 2.50E-16 | 291 | 165 |
| GO:BP | response to oxygen-containing compound | GO:1901700 | 1480 | 2.60E-16 | 291 | 53 |
| TF | Factor: PEA3; motif: NNCAGGAARNN | TF:M09915 | 10849 | 2.60E-16 | 291 | 164 |
| REAC | Immune System | REAC:R-HSA-168256 | 1770 | 2.80E-16 | 291 | 57 |
| TF | Factor: p300; motif: ACNTCCG | TF:M04826 | 15885 | 3.30E-16 | 291 | 207 |
| TF | Factor: FOXO1A:ETV7; motif: NWMAACAGGAMNNNCTTCCNN | TF:M08304 | 12194 | 3.40E-16 | 291 | 176 |
| TF | Factor: RXRA; motif: RRGGTCATGACCYY | TF:M04490 | 6522 | 3.80E-16 | 291 | 120 |
| TF | Factor: egr-3; motif: GTGGGY | TF:M03818 | 11653 | 4.00E-16 | 291 | 171 |
| TF | Factor: IRF-4; motif: AAGTTTC | TF:M04855 | 6624 | 4.40E-16 | 291 | 121 |
| TF | Factor: LUMAN; motif: CYCAGCYYCY | TF:M09729 | 9533 | 4.40E-16 | 291 | 151 |
| TF | Factor: ZBTB39; motif: CNCTGY; match class: 1 | TF:M12689_1 | 8347 | 5.40E-16 | 291 | 139 |
| TF | Factor: E2F4; motif: YCCCGCCNCNNSSNNSNN | TF:M12599 | 15611 | 8.30E-16 | 291 | 204 |
| TF | Factor: HSF4; motif: CTGCMRN; match class: 1 | TF:M07322_1 | 10436 | 8.90E-16 | 291 | 159 |
| TF | Factor: IRF-4; motif: RGGAASWGR | TF:M04818 | 8202 | 9.60E-16 | 291 | 137 |
| TF | Factor: c-Ets-2; motif: CTTCCTG | TF:M01207 | 8932 | 1.40E-15 | 291 | 144 |
| TF | Factor: Elk-1; motif: CTTCCKG | TF:M07252 | 8932 | 1.40E-15 | 291 | 144 |
| GO:BP | regulation of developmental process | GO:0050793 | 2221 | 1.60E-15 | 291 | 64 |
| TF | Factor: NFATc2; motif: GGAAAA | TF:M03555 | 13979 | 1.60E-15 | 291 | 190 |
| TF | Factor: NFATc3; motif: GGAAAA | TF:M01886 | 13979 | 1.60E-15 | 291 | 190 |
| TF | Factor: NFATc2; motif: GGAAAA | TF:M01281 | 13979 | 1.60E-15 | 291 | 190 |
| TF | Factor: LRH-1; motif: TCAAGGTCRYGACCTTGR | TF:M11828 | 9272 | 2.10E-15 | 291 | 147 |
| GO:BP | regulation of cell death | GO:0010941 | 1440 | 2.30E-15 | 291 | 51 |
| TF | Factor: Sox-10; motif: CWTTGT | TF:M03138 | 11286 | 2.30E-15 | 291 | 166 |
| TF | Factor: TCF-1; motif: CTTTGW | TF:M03857 | 11286 | 2.30E-15 | 291 | 166 |
| TF | Factor: GKLF; motif: CCTCCYN; match class: 1 | TF:M01835_1 | 9081 | 2.40E-15 | 291 | 145 |
| TF | Factor: Smad2; motif: AGACAN; match class: 1 | TF:M03580_1 | 7607 | 2.40E-15 | 291 | 130 |
| TF | Factor: GATAD2A; motif: CCTKTG | TF:M09726 | 13089 | 2.50E-15 | 291 | 182 |
| TF | Factor: Pax-5; motif: RRNGRNGCAN | TF:M03577 | 7727 | 3.20E-15 | 291 | 131 |
| TF | Factor: ZXDA; motif: NAGGGTG | TF:M06203 | 6420 | 3.20E-15 | 291 | 117 |
| TF | Factor: ZXDB; motif: NAGGGTG | TF:M06202 | 6420 | 3.20E-15 | 291 | 117 |
| TF | Factor: ZNF273; motif: GAGAGGAGCTAC | TF:M10460 | 11221 | 3.60E-15 | 291 | 165 |
| TF | Factor: Kaiso; motif: GCMGGGRGCRGS | TF:M03876 | 12564 | 3.80E-15 | 291 | 177 |
| TF | Factor: NF1B; motif: CYTGGCNYNCWGCCAN | TF:M09762 | 4967 | 4.90E-15 | 291 | 100 |
| TF | Factor: GR; motif: RGNACANKNTGTNCY | TF:M09625 | 7867 | 5.20E-15 | 291 | 132 |
| TF | Factor: NR1B1; motif: NRGGNCRTGACCTN | TF:M11796 | 9466 | 5.50E-15 | 291 | 148 |
| TF | Factor: ZNFPT1; motif: YCNNCNCWGCCNY | TF:M12721 | 8164 | 5.60E-15 | 291 | 135 |
| TF | Factor: Elk-1; motif: GGAAGN | TF:M03819 | 12382 | 5.80E-15 | 291 | 175 |
| TF | Factor: Erg; motif: NACCGGATATCCGGTN; match class: 1 | TF:M11393_1 | 12292 | 7.10E-15 | 291 | 174 |
| GO:CC | membrane | GO:0016020 | 8518 | 8.50E-15 | 291 | 136 |
| GO:BP | cellular developmental process | GO:0048869 | 3759 | 1.10E-14 | 291 | 84 |
| GO:CC | organelle | GO:0043226 | 13412 | 1.20E-14 | 291 | 181 |
| TF | Factor: MAZ; motif: GGGMGGGGSSGGGGGGGGGGGG | TF:M09636 | 14816 | 1.30E-14 | 291 | 195 |
| TF | Factor: LRH-1; motif: TGACCTTGRNYCAAGGTCA; match class: 1 | TF:M11830_1 | 5296 | 1.40E-14 | 291 | 103 |
| TF | Factor: CDP:SRF; motif: NCCWTAYAAGGTMNKRATCRATN | TF:M08520 | 8843 | 1.40E-14 | 291 | 141 |
| TF | Factor: PRDM16; motif: KGGTCATRACCM | TF:M05946 | 6003 | 1.50E-14 | 291 | 111 |
| TF | Factor: Sox-9; motif: NNNNNACAAARGNNSMN | TF:M07269 | 8565 | 1.70E-14 | 291 | 138 |
| GO:BP | cell death | GO:0008219 | 1881 | 2.00E-14 | 291 | 57 |
| TF | Factor: PARP; motif: TTTCYN | TF:M02027 | 14025 | 2.00E-14 | 291 | 188 |
| GO:CC | extracellular exosome | GO:0070062 | 1901 | 2.20E-14 | 291 | 56 |
| TF | Factor: AP-2gamma:Elk-1; motif: NGCCKNRGGSGRCGGAAGTG | TF:M08441 | 14756 | 2.20E-14 | 291 | 194 |
| TF | Factor: FOXO1A:PDEF; motif: WNCCGGATGTTDN | TF:M08486 | 9927 | 2.50E-14 | 291 | 151 |
| GO:BP | response to cytokine | GO:0034097 | 833 | 2.70E-14 | 291 | 38 |
| GO:BP | inflammatory response | GO:0006954 | 651 | 2.70E-14 | 291 | 34 |
| TF | Factor: LBP-1; motif: CAGCTGS | TF:M00644 | 7824 | 2.80E-14 | 291 | 130 |
| GO:BP | cellular response to cytokine stimulus | GO:0071345 | 744 | 3.20E-14 | 291 | 36 |
| GO:BP | positive regulation of metabolic process | GO:0009893 | 3369 | 3.50E-14 | 291 | 78 |
| GO:CC | extracellular vesicle | GO:1903561 | 1921 | 3.50E-14 | 291 | 56 |
| GO:CC | extracellular membrane-bounded organelle | GO:0065010 | 1922 | 3.50E-14 | 291 | 56 |
| GO:CC | extracellular organelle | GO:0043230 | 1922 | 3.50E-14 | 291 | 56 |
| GO:CC | cytoplasm | GO:0005737 | 11064 | 3.70E-14 | 291 | 159 |
| GO:BP | cell differentiation | GO:0030154 | 3683 | 4.00E-14 | 291 | 82 |
| TF | Factor: TFII-I; motif: NAGGAAGTGN | TF:M04636 | 11048 | 4.30E-14 | 291 | 161 |
| GO:BP | negative regulation of cellular process | GO:0048523 | 4412 | 4.70E-14 | 291 | 91 |
| TF | Factor: RORA; motif: CCYTGNCCTN | TF:M12659 | 6920 | 4.70E-14 | 291 | 120 |
| TF | Factor: NF-1C; motif: NYTGGCNNYNNGCCARN | TF:M10002 | 5930 | 5.60E-14 | 291 | 109 |
| GO:BP | response to endogenous stimulus | GO:0009719 | 1439 | 5.70E-14 | 291 | 49 |
| GO:BP | localization | GO:0051179 | 5792 | 5.80E-14 | 291 | 107 |
| TF | Factor: ZNF586; motif: CAGGCCYRGAGG | TF:M10491 | 12307 | 6.30E-14 | 291 | 172 |
| TF | Factor: PURBETA; motif: NCWGRGGCTGGGG | TF:M12726 | 6869 | 7.70E-14 | 291 | 119 |
| TF | Factor: Zic1; motif: KGGGTGGTC | TF:M00448 | 9729 | 7.70E-14 | 291 | 148 |
| GO:BP | epithelium development | GO:0060429 | 1015 | 9.10E-14 | 291 | 41 |
| GO:BP | cell activation | GO:0001775 | 914 | 9.20E-14 | 291 | 39 |
| GO:BP | defense response | GO:0006952 | 1343 | 9.70E-14 | 291 | 47 |
| TF | Factor: GTF2IRD1-isoform2; motif: GGGATTRNR | TF:M01229 | 13062 | 1.20E-13 | 291 | 178 |
| TF | Factor: VDR; motif: RRGGTCANNGRGKTCA | TF:M09671 | 7096 | 1.20E-13 | 291 | 121 |
| TF | Factor: NURR1:RXR-ALPHA; motif: NRGGTCRTTGACCYN | TF:M08957 | 9681 | 1.30E-13 | 291 | 147 |
| TF | Factor: Ikaros; motif: TGGGAGN | TF:M07260 | 11832 | 1.40E-13 | 291 | 167 |
| TF | Factor: T3R-beta; motif: NRGGTCAAAGGTCRN | TF:M11814 | 12177 | 1.50E-13 | 291 | 170 |
| TF | Factor: MafB; motif: GNTGAC | TF:M01227 | 10227 | 1.60E-13 | 291 | 152 |
| GO:BP | regulation of biological quality | GO:0065008 | 3465 | 1.70E-13 | 291 | 78 |
| GO:BP | positive regulation of macromolecule metabolic process | GO:0010604 | 3089 | 1.70E-13 | 291 | 73 |
| GO:BP | regulation of programmed cell death | GO:0043067 | 1308 | 1.80E-13 | 291 | 46 |
| TF | Factor: MAFB; motif: NTCAGCN | TF:M08888 | 8591 | 1.80E-13 | 291 | 136 |
| TF | Factor: MAZ; motif: GGGMGGGGSSGGGGGGGGGGGG; match class: 1 | TF:M09636_1 | 13118 | 1.90E-13 | 291 | 178 |
| GO:BP | tube morphogenesis | GO:0035239 | 788 | 2.00E-13 | 291 | 36 |
| TF | Factor: Sox-18; motif: CAAWGBB; match class: 1 | TF:M03848_1 | 10894 | 2.10E-13 | 291 | 158 |
| TF | Factor: T3R-beta; motif: NRGGTCAAAGGTCAN | TF:M11816 | 10084 | 3.10E-13 | 291 | 150 |
| TF | Factor: IRX-1; motif: NACRYNNNNNNNNRYGNN | TF:M11018 | 15325 | 3.30E-13 | 291 | 196 |
| TF | Factor: E2F-3:TBR2; motif: ANGTGYKANGGCGCSTTNNCRNNT | TF:M08207 | 15326 | 3.30E-13 | 291 | 196 |
| GO:BP | regulation of response to stimulus | GO:0048583 | 3512 | 3.60E-13 | 291 | 78 |
| TF | Factor: PHB; motif: NCCCAGCCCCY | TF:M12725 | 8966 | 3.80E-13 | 291 | 139 |
| TF | Factor: ZGPAT; motif: GRGGCWGNGGNG | TF:M09739 | 7490 | 4.00E-13 | 291 | 124 |
| GO:BP | regulation of apoptotic process | GO:0042981 | 1281 | 4.20E-13 | 291 | 45 |
| GO:CC | membrane-bounded organelle | GO:0043227 | 12679 | 4.20E-13 | 291 | 171 |
| GO:BP | cellular response to oxygen-containing compound | GO:1901701 | 1062 | 4.30E-13 | 291 | 41 |
| GO:BP | cell migration | GO:0016477 | 1339 | 4.30E-13 | 291 | 46 |
| GO:CC | secretory granule | GO:0030141 | 760 | 4.40E-13 | 291 | 34 |
| GO:BP | wound healing | GO:0042060 | 384 | 4.60E-13 | 291 | 26 |
| TF | Factor: MEL1; motif: GATGAG | TF:M08793 | 10128 | 4.70E-13 | 291 | 150 |
| TF | Factor: NKX2-2; motif: NNNCCACTCAANNN | TF:M12456 | 8199 | 5.30E-13 | 291 | 131 |
| TF | Factor: HLTF; motif: AGSCARAAAGYRGSTGS | TF:M04613 | 6765 | 5.70E-13 | 291 | 116 |
| TF | Factor: MZF-1; motif: TGGGGAR | TF:M01733 | 10043 | 5.70E-13 | 291 | 149 |
| GO:BP | tube development | GO:0035295 | 967 | 6.00E-13 | 291 | 39 |
| TF | Factor: REST; motif: AAGGTGCT | TF:M04926 | 5504 | 6.20E-13 | 291 | 102 |
| GO:BP | response to wounding | GO:0009611 | 505 | 6.40E-13 | 291 | 29 |
| TF | Factor: DREF; motif: CTYYCWCTTCCY | TF:M09725 | 6415 | 7.10E-13 | 291 | 112 |
| GO:CC | intracellular anatomical structure | GO:0005622 | 14269 | 7.90E-13 | 291 | 184 |
| GO:MF | extracellular matrix structural constituent | GO:0005201 | 156 | 8.10E-13 | 291 | 18 |
| TF | Factor: Pax-5; motif: BCNNNRNGCANBGNTGNRTAGCSGCHNB | TF:M00143 | 10199 | 9.20E-13 | 291 | 150 |
| TF | Factor: GR; motif: NNNNNNCNNTNTGTNCTNN | TF:M00192 | 6349 | 9.60E-13 | 291 | 111 |
| TF | Factor: PAX5; motif: RNGCGTGACCNN | TF:M09817 | 11285 | 1.00E-12 | 291 | 160 |
| TF | Factor: BRN1; motif: HAATGCN | TF:M03813 | 11080 | 1.10E-12 | 291 | 158 |
| TF | Factor: TWIST; motif: CACCTGG | TF:M03582 | 7309 | 1.20E-12 | 291 | 121 |
| TF | Factor: CP2; motif: NNNNCCAGNCNN | TF:M07602 | 9815 | 1.30E-12 | 291 | 146 |
| GO:BP | blood vessel morphogenesis | GO:0048514 | 563 | 1.40E-12 | 291 | 30 |
| TF | Factor: KLF; motif: GGGNGGGG | TF:M07461 | 7906 | 1.50E-12 | 291 | 127 |
| TF | Factor: GABP-alpha; motif: AACCGGAAR | TF:M04748 | 14181 | 1.50E-12 | 291 | 185 |
| GO:BP | positive regulation of response to stimulus | GO:0048584 | 1882 | 1.60E-12 | 291 | 54 |
| TF | Factor: Fli-1; motif: NNRGGMAGGAAGGRRRGR | TF:M09920 | 8216 | 1.70E-12 | 291 | 130 |
| TF | Factor: E2F-3:Prrxl1; motif: SGCGCTAATTNN | TF:M08206 | 10480 | 1.70E-12 | 291 | 152 |
| GO:BP | extracellular matrix organization | GO:0030198 | 300 | 1.80E-12 | 291 | 23 |
| GO:BP | extracellular structure organization | GO:0043062 | 301 | 1.90E-12 | 291 | 23 |
| TF | Factor: ZNF684; motif: MAAGGGGTGGACTGT; match class: 1 | TF:M10550_1 | 8324 | 1.90E-12 | 291 | 131 |
| GO:BP | external encapsulating structure organization | GO:0045229 | 302 | 2.10E-12 | 291 | 23 |
| TF | Factor: RUNX2; motif: NRACCGCAAACCGCAN | TF:M04106 | 12597 | 2.10E-12 | 291 | 171 |
| TF | Factor: PEA3; motif: NNCAGGAARNN; match class: 1 | TF:M09915_1 | 4914 | 2.30E-12 | 291 | 94 |
| TF | Factor: MRF4; motif: CASCTGC | TF:M03831 | 7565 | 2.40E-12 | 291 | 123 |
| TF | Factor: HTF4; motif: CASCTGB | TF:M02018 | 7565 | 2.40E-12 | 291 | 123 |
| TF | Factor: TCF-7; motif: TCAAAG | TF:M00805 | 10201 | 2.50E-12 | 291 | 149 |
| TF | Factor: TCF-3; motif: CTTTGA | TF:M03858 | 10201 | 2.50E-12 | 291 | 149 |
| TF | Factor: AR; motif: ARGAACANNNTGTNC | TF:M07204 | 4006 | 2.60E-12 | 291 | 83 |
| TF | Factor: ERG; motif: ACCGGAWATCCGGT | TF:M03976 | 11502 | 2.60E-12 | 291 | 161 |
| TF | Factor: ZNF37A; motif: CCYYGGCTCCNTSCCMN | TF:M12354 | 10103 | 2.70E-12 | 291 | 148 |
| TF | Factor: KLF15; motif: RCCMCRCCCMCN | TF:M12160 | 11726 | 2.70E-12 | 291 | 163 |
| TF | Factor: ER71:E2A; motif: CASSTGNACCGGAWRYN; match class: 1 | TF:M08568_1 | 13552 | 2.80E-12 | 291 | 179 |
| TF | Factor: NF-KAPPAB1; motif: NGGKRNTTYCCCN | TF:M08952 | 7005 | 2.80E-12 | 291 | 117 |
| TF | Factor: Erg; motif: MCAGGAAA | TF:M07284 | 9694 | 2.90E-12 | 291 | 144 |
| TF | Factor: Pax-4; motif: DTTTTCCACCN; match class: 1 | TF:M04624_1 | 8077 | 3.10E-12 | 291 | 128 |
| TF | Factor: GEMIN3; motif: NCWGGRARRGRGNGNG; match class: 1 | TF:M09727_1 | 2934 | 3.10E-12 | 291 | 69 |
| TF | Factor: SMAD5; motif: GSGGCAGM | TF:M03846 | 7589 | 3.10E-12 | 291 | 123 |
| GO:BP | positive regulation of developmental process | GO:0051094 | 1182 | 3.20E-12 | 291 | 42 |
| TF | Factor: E2F-4; motif: SNGGGCGGGAANN | TF:M09894 | 14891 | 3.60E-12 | 291 | 190 |
| TF | Factor: Erm; motif: NRRSAGGAARNGRN | TF:M09916 | 5923 | 3.90E-12 | 291 | 105 |
| TF | Factor: T3R-beta; motif: NTGACCTNRNYNAGGTCAN | TF:M11820 | 4448 | 4.00E-12 | 291 | 88 |
| TF | Factor: EHF; motif: BTTCCTGC | TF:M07251 | 5394 | 4.10E-12 | 291 | 99 |
| GO:BP | programmed cell death | GO:0012501 | 1737 | 4.70E-12 | 291 | 51 |
| TF | Factor: AP-2gamma; motif: GCCYNNGGS | TF:M00470 | 8826 | 5.00E-12 | 291 | 135 |
| TF | Factor: Churchill; motif: CGGGNN | TF:M00986 | 13164 | 5.20E-12 | 291 | 175 |
| TF | Factor: LUMAN; motif: CYCAGCYYCY; match class: 1 | TF:M09729_1 | 4222 | 5.40E-12 | 291 | 85 |
| TF | Factor: RelA-p65; motif: AAASTCCC | TF:M04849 | 5419 | 5.60E-12 | 291 | 99 |
| TF | Factor: E2F-3:HES-7; motif: NNNSGCGCSNNNNNCRCGYGNN | TF:M08526 | 15314 | 5.70E-12 | 291 | 193 |
| GO:CC | endomembrane system | GO:0012505 | 4326 | 6.60E-12 | 291 | 84 |
| TF | Factor: Sox-17; motif: TTGTYY; match class: 1 | TF:M03803_1 | 7955 | 6.60E-12 | 291 | 126 |
| TF | Factor: ZNF300; motif: SWGGGGGSN | TF:M07486 | 11503 | 6.80E-12 | 291 | 160 |
| TF | Factor: NF-E4; motif: GTGAGGS | TF:M08826 | 10022 | 8.70E-12 | 291 | 146 |
| TF | Factor: GCMa:PEA3; motif: ATRCGGGCGGAAGTR | TF:M08313 | 13815 | 9.20E-12 | 291 | 180 |
| GO:BP | cellular response to endogenous stimulus | GO:0071495 | 1220 | 9.40E-12 | 291 | 42 |
| TF | Factor: ZNF777; motif: GTCCGYCCCGTCSAACAAT | TF:M08993 | 9617 | 9.80E-12 | 291 | 142 |
| GO:BP | biological process involved in interspecies interaction between organisms | GO:0044419 | 1282 | 1.10E-11 | 291 | 43 |
| GO:CC | intrinsic component of membrane | GO:0031224 | 5051 | 1.10E-11 | 291 | 92 |
| TF | Factor: Lyl-1; motif: NCAKCTGYTNYCNN; match class: 1 | TF:M09977_1 | 6570 | 1.10E-11 | 291 | 111 |
| TF | Factor: ZNF436; motif: TCCTCCAGGAAGCCY | TF:M10477 | 12825 | 1.40E-11 | 291 | 171 |
| TF | Factor: TCF-1; motif: ACATCGRGRCGCTGW | TF:M11603 | 14822 | 1.40E-11 | 291 | 188 |
| TF | Factor: ZFP14; motif: SCNNYCCNGNNSCTSCNC | TF:M12694 | 9765 | 1.50E-11 | 291 | 143 |
| TF | Factor: ZNF592; motif: NSARNATGGAGKN | TF:M09766 | 6223 | 1.50E-11 | 291 | 107 |
| GO:BP | positive regulation of multicellular organismal process | GO:0051240 | 1298 | 1.60E-11 | 291 | 43 |
| TF | Factor: HDAC1; motif: KGCARGGTC | TF:M07041 | 11165 | 1.60E-11 | 291 | 156 |
| TF | Factor: c-Fos; motif: ACTCACCA | TF:M04802 | 6417 | 1.60E-11 | 291 | 109 |
| GO:BP | negative regulation of multicellular organismal process | GO:0051241 | 913 | 1.80E-11 | 291 | 36 |
| GO:BP | locomotion | GO:0040011 | 1669 | 1.80E-11 | 291 | 49 |
| GO:BP | immune response | GO:0006955 | 1363 | 1.90E-11 | 291 | 44 |
| TF | Factor: SRY; motif: TTGTTT | TF:M03854 | 12068 | 1.90E-11 | 291 | 164 |
| TF | Factor: FOXO1A; motif: AAACAA | TF:M03823 | 12068 | 1.90E-11 | 291 | 164 |
| TF | Factor: TORC2; motif: TGGGCTKKD | TF:M07341 | 4235 | 2.00E-11 | 291 | 84 |
| TF | Factor: NR3C1; motif: NRGWACAYNRTGTWCYN | TF:M04476 | 6162 | 2.10E-11 | 291 | 106 |
| GO:BP | heart development | GO:0007507 | 535 | 2.20E-11 | 291 | 28 |
| TF | Factor: Kaiso; motif: TCTCGCGAG | TF:M04691 | 13571 | 2.20E-11 | 291 | 177 |
| TF | Factor: Zic3; motif: NGGGKGGTC; match class: 1 | TF:M00450_1 | 7106 | 2.20E-11 | 291 | 116 |
| TF | Factor: AP-2rep; motif: CAGTGGG | TF:M00468 | 6084 | 2.40E-11 | 291 | 105 |
| MIRNA | hsa-miR-335-5p | MIRNA:hsa-miR-335-5p | 2271 | 2.50E-11 | 291 | 57 |
| GO:BP | response to other organism | GO:0051707 | 1142 | 2.60E-11 | 291 | 40 |
| GO:BP | response to external biotic stimulus | GO:0043207 | 1145 | 2.80E-11 | 291 | 40 |
| TF | Factor: E2F-3; motif: NNTTTTGGCGCCAAAACT | TF:M03962 | 9734 | 2.80E-11 | 291 | 142 |
| TF | Factor: VDR:RXR-ALPHA; motif: NRGGTCANNNGGTTCNN | TF:M08980 | 9219 | 2.90E-11 | 291 | 137 |
| REAC | Neutrophil degranulation | REAC:R-HSA-6798695 | 446 | 3.00E-11 | 291 | 25 |
| GO:BP | apoptotic process | GO:0006915 | 1692 | 3.10E-11 | 291 | 49 |
| GO:BP | cell motility | GO:0048870 | 1504 | 3.10E-11 | 291 | 46 |
| GO:BP | localization of cell | GO:0051674 | 1504 | 3.10E-11 | 291 | 46 |
| TF | Factor: Fli-1:Max; motif: NCCGGAAACACGTGN | TF:M08289 | 8814 | 3.10E-11 | 291 | 133 |
| TF | Factor: E2F2; motif: AAAATGGCGCCATTTT | TF:M04517 | 8014 | 3.10E-11 | 291 | 125 |
| TF | Factor: Fli-1:FIGLA; motif: NCCGGAARCASSTGN | TF:M08284 | 6950 | 3.20E-11 | 291 | 114 |
| TF | Factor: HDAC1; motif: KGCARGGTC; match class: 1 | TF:M07041_1 | 5751 | 3.40E-11 | 291 | 101 |
| TF | Factor: GKLF; motif: NNNRGGNGNGGSN; match class: 1 | TF:M07289_1 | 9758 | 3.50E-11 | 291 | 142 |
| TF | Factor: myogenin; motif: CRSCTGTTBNNTTTGGCACGSNGCCARCH | TF:M00056 | 9551 | 3.60E-11 | 291 | 140 |
| TF | Factor: GATAD2A; motif: CCTKTG; match class: 1 | TF:M09726_1 | 7641 | 3.80E-11 | 291 | 121 |
| TF | Factor: ZNF462; motif: YYYYCTSCWG | TF:M12707 | 8440 | 4.10E-11 | 291 | 129 |
| TF | Factor: ZF5; motif: GGSGCGCGS | TF:M10438 | 14973 | 4.50E-11 | 291 | 188 |
| TF | Factor: Elk-1:PDEF; motif: NCMGGAMGGANATCCGGN | TF:M08227 | 7470 | 4.80E-11 | 291 | 119 |
| GO:CC | integral component of membrane | GO:0016021 | 4917 | 5.00E-11 | 291 | 89 |
| GO:BP | system process | GO:0003008 | 1781 | 5.20E-11 | 291 | 50 |
| TF | Factor: GCMa; motif: RTGCGGGTN | TF:M11595 | 10870 | 5.30E-11 | 291 | 152 |
| TF | Factor: Erg; motif: NRRSAGGAAGNGG; match class: 1 | TF:M09907_1 | 4480 | 5.50E-11 | 291 | 86 |
| TF | Factor: T3R-beta; motif: NTGACCTNRNYNAGGTCAN; match class: 1 | TF:M11820_1 | 4065 | 5.70E-11 | 291 | 81 |
| GO:BP | movement of cell or subcellular component | GO:0006928 | 1918 | 5.80E-11 | 291 | 52 |
| GO:CC | intracellular organelle | GO:0043229 | 12728 | 6.00E-11 | 291 | 166 |
| TF | Factor: AR; motif: RGGWACAYNGTGTWCYN | TF:M04453 | 6079 | 6.30E-11 | 291 | 104 |
| TF | Factor: PUR1; motif: GGGNCAGNN; match class: 1 | TF:M01721_1 | 3127 | 7.20E-11 | 291 | 69 |
| GO:BP | angiogenesis | GO:0001525 | 477 | 7.80E-11 | 291 | 26 |
| GO:BP | response to biotic stimulus | GO:0009607 | 1182 | 7.80E-11 | 291 | 40 |
| TF | Factor: RELA; motif: GGGRMTKYCCC | TF:M12657 | 5734 | 7.80E-11 | 291 | 100 |
| TF | Factor: PU.1; motif: WGAGGAAG | TF:M00658 | 3851 | 8.70E-11 | 291 | 78 |
| REAC | Extracellular matrix organization | REAC:R-HSA-1474244 | 276 | 9.10E-11 | 291 | 20 |
| TF | Factor: ZXDL; motif: NGGGGWS | TF:M05775 | 6205 | 9.10E-11 | 291 | 105 |
| GO:CC | secretory vesicle | GO:0099503 | 920 | 1.00E-10 | 291 | 34 |
| GO:BP | cell population proliferation | GO:0008283 | 1750 | 1.10E-10 | 291 | 49 |
| TF | Factor: MLLT10; motif: CYNNCCNNGGNGCTG | TF:M12636 | 8543 | 1.10E-10 | 291 | 129 |
| TF | Factor: HOXA3; motif: NNNNRNTAATTARY | TF:M01337 | 13550 | 1.10E-10 | 291 | 175 |
| REAC | Innate Immune System | REAC:R-HSA-168249 | 914 | 1.20E-10 | 291 | 34 |
| TF | Factor: Sox-10; motif: NACAAWG | TF:M02116 | 12073 | 1.20E-10 | 291 | 162 |
| TF | Factor: SRY; motif: AACAATNR | TF:M08976 | 11740 | 1.20E-10 | 291 | 159 |
| TF | Factor: HNRPUL1; motif: NCNCAGN | TF:M09746 | 12084 | 1.30E-10 | 291 | 162 |
| TF | Factor: GATA-5; motif: TATCTN | TF:M02006 | 7582 | 1.50E-10 | 291 | 119 |
| TF | Factor: GCMa:FOXO1A; motif: GTMAATAMGGGTRN | TF:M08318 | 10029 | 1.50E-10 | 291 | 143 |
| GO:CC | integral component of plasma membrane | GO:0005887 | 1466 | 1.60E-10 | 291 | 43 |
| TF | Factor: NR3C1; motif: NRGWACAYNRTGTWCYN; match class: 1 | TF:M04476_1 | 4480 | 1.60E-10 | 291 | 85 |
| TF | Factor: ING4; motif: CCACCA; match class: 1 | TF:M01743_1 | 8903 | 1.80E-10 | 291 | 132 |
| TF | Factor: Elk-1:OC-2; motif: RCCGGAASCGATCGATNN | TF:M08223 | 11348 | 1.80E-10 | 291 | 155 |
| TF | Factor: SREBP-1; motif: CACSCCA | TF:M00749 | 7520 | 2.00E-10 | 291 | 118 |
| GO:BP | regulation of multicellular organismal development | GO:2000026 | 1218 | 2.10E-10 | 291 | 40 |
| GO:BP | negative regulation of cell death | GO:0060548 | 884 | 2.10E-10 | 291 | 34 |
| GO:CC | intrinsic component of plasma membrane | GO:0031226 | 1541 | 2.10E-10 | 291 | 44 |
| TF | Factor: Pet-1; motif: GCNGGAAGYG | TF:M09918 | 12251 | 2.10E-10 | 291 | 163 |
| TF | Factor: TF3C-beta; motif: CCNGGAGGGCTTCCTGGAGGAG | TF:M04863 | 12366 | 2.10E-10 | 291 | 164 |
| TF | Factor: Sox-10; motif: CWTTGTN | TF:M01131 | 7621 | 2.10E-10 | 291 | 119 |
| GO:BP | negative regulation of apoptotic process | GO:0043066 | 783 | 2.30E-10 | 291 | 32 |
| TF | Factor: ZNF614; motif: NCYCWGCCYYNNN | TF:M09862 | 8627 | 2.30E-10 | 291 | 129 |
| TF | Factor: ZF5; motif: GSGCGCGR | TF:M00716 | 14856 | 2.90E-10 | 291 | 185 |
| TF | Factor: YB-1; motif: CCAMTCNGMR | TF:M03805 | 8857 | 2.90E-10 | 291 | 131 |
| TF | Factor: NF-kappaB; motif: NGGGACTTTCCA | TF:M00208 | 3300 | 3.00E-10 | 291 | 70 |
| TF | Factor: AP-4; motif: RNCAGCTGC | TF:M00927 | 5587 | 3.10E-10 | 291 | 97 |
| GO:BP | regulation of metabolic process | GO:0019222 | 6103 | 3.70E-10 | 291 | 102 |
| GO:BP | epithelial cell differentiation | GO:0030855 | 555 | 3.70E-10 | 291 | 27 |
| GO:BP | negative regulation of programmed cell death | GO:0043069 | 799 | 4.00E-10 | 291 | 32 |
| TF | Factor: sin3A; motif: TGTCCNNGGTGCTG; match class: 1 | TF:M04756_1 | 6912 | 4.00E-10 | 291 | 111 |
| TF | Factor: CTF1; motif: TGGCASCNNGCCAA | TF:M01196 | 4131 | 4.10E-10 | 291 | 80 |
| TF | Factor: ESE-1; motif: NTGTGCGGATGCN | TF:M11385 | 10801 | 4.40E-10 | 291 | 149 |
| TF | Factor: NF-1B; motif: CTGGCASGV | TF:M07051 | 3030 | 5.60E-10 | 291 | 66 |
| TF | Factor: E2F-3; motif: NTTTTGGCGCCAAAAN | TF:M11526 | 6287 | 5.80E-10 | 291 | 104 |
| TF | Factor: ZNF333; motif: ATAAT | TF:M01230 | 13185 | 5.90E-10 | 291 | 170 |
| TF | Factor: RXR-alpha; motif: NNGNNRNNNARAGGTCANNG | TF:M10055 | 5021 | 6.00E-10 | 291 | 90 |
| TF | Factor: AP-4:Fli-1; motif: RSCGGAWRCAGSTGN | TF:M08445 | 8339 | 6.60E-10 | 291 | 125 |
| TF | Factor: CP2; motif: GCHCDAMCCAG | TF:M00072 | 7846 | 7.00E-10 | 291 | 120 |
| REAC | Integrin cell surface interactions | REAC:R-HSA-216083 | 77 | 7.40E-10 | 291 | 12 |
| TF | Factor: PR; motif: NNNNNNGGNACRNNNTGTTCTNNNNNN | TF:M00954 | 4519 | 7.50E-10 | 291 | 84 |
| TF | Factor: LKLF; motif: GGGGTGGKSN | TF:M07261 | 8865 | 7.70E-10 | 291 | 130 |
| TF | Factor: MAZ; motif: GGGGAGGG | TF:M00649 | 8569 | 8.40E-10 | 291 | 127 |
| GO:BP | cell-cell adhesion | GO:0098609 | 828 | 1.00E-09 | 291 | 32 |
| GO:BP | tissue morphogenesis | GO:0048729 | 581 | 1.10E-09 | 291 | 27 |
| TF | Factor: nerf; motif: YRNCAGGAAGYRGSTBDS | TF:M00531 | 6635 | 1.10E-09 | 291 | 107 |
| GO:MF | signaling receptor binding | GO:0005102 | 1265 | 1.20E-09 | 291 | 39 |
| GO:BP | collagen fibril organization | GO:0030199 | 71 | 1.20E-09 | 291 | 12 |
| TF | Factor: c-Maf; motif: NWNNNTGCTGACKNNNNNN | TF:M01070 | 5525 | 1.20E-09 | 291 | 95 |
| TF | Factor: NFE2L1; motif: NNNATGACTCAGCANW | TF:M12499 | 8308 | 1.20E-09 | 291 | 124 |
| TF | Factor: TEF-1; motif: GRRATG | TF:M00704 | 10603 | 1.20E-09 | 291 | 146 |
| TF | Factor: SALL2; motif: GGGTGGG; match class: 1 | TF:M04595_1 | 6741 | 1.30E-09 | 291 | 108 |
| TF | Factor: IRF-7; motif: AAGWGAA | TF:M01884 | 9133 | 1.30E-09 | 291 | 132 |
| TF | Factor: AP-2gamma; motif: NTGSCCTGRGGSNN | TF:M09591 | 7528 | 1.40E-09 | 291 | 116 |
| GO:BP | response to lipid | GO:0033993 | 787 | 1.50E-09 | 291 | 31 |
| TF | Factor: CSX; motif: NKRAGWGS; match class: 1 | TF:M10008_1 | 3652 | 1.50E-09 | 291 | 73 |
| TF | Factor: E2F3; motif: NNRGMKGGAR | TF:M12598 | 11508 | 1.60E-09 | 291 | 154 |
| TF | Factor: ZNF342; motif: TRSTGGACRNT | TF:M12075 | 5465 | 1.60E-09 | 291 | 94 |
| TF | Factor: LRF; motif: RCGACCACCNN | TF:M12230 | 7744 | 1.70E-09 | 291 | 118 |
| GO:CC | cytoplasmic vesicle | GO:0031410 | 2275 | 1.80E-09 | 291 | 53 |
| TF | Factor: SMAD4; motif: GKSRKKCAGMCANCY | TF:M00733 | 7655 | 1.80E-09 | 291 | 117 |
| GO:CC | intracellular vesicle | GO:0097708 | 2277 | 1.90E-09 | 291 | 53 |
| TF | Factor: HNF-4alpha; motif: AGTCCAAR | TF:M04903 | 5939 | 1.90E-09 | 291 | 99 |
| GO:BP | leukocyte activation | GO:0045321 | 795 | 2.00E-09 | 291 | 31 |
| GO:BP | response to bacterium | GO:0009617 | 504 | 2.00E-09 | 291 | 25 |
| TF | Factor: GLI; motif: NGACCMCCCAN | TF:M07290 | 6316 | 2.00E-09 | 291 | 103 |
| TF | Factor: GKLF; motif: NNRRGRRNGNSNNN; match class: 1 | TF:M07040_1 | 7566 | 2.00E-09 | 291 | 116 |
| TF | Factor: AP-4:Max; motif: NCAGCTGNNNNNNNCACGTGN | TF:M08674 | 10025 | 2.10E-09 | 291 | 140 |
| TF | Factor: GCMa:Erg; motif: ATGCGGGCGGAARKG | TF:M08487 | 12781 | 2.10E-09 | 291 | 165 |
| GO:BP | regulation of macromolecule metabolic process | GO:0060255 | 5629 | 2.20E-09 | 291 | 95 |
| TF | Factor: FXR:RXR-ALPHA; motif: NAGKTCATTGACCYN | TF:M08954 | 5497 | 2.30E-09 | 291 | 94 |
| TF | Factor: NMYC; motif: CAYCTG; match class: 1 | TF:M01808_1 | 5049 | 2.30E-09 | 291 | 89 |
| TF | Factor: LRF; motif: GGGGKYNNB | TF:M01100 | 6997 | 2.30E-09 | 291 | 110 |
| TF | Factor: FOXO1A:Elk-1; motif: RWMAACAGGAAGTN | TF:M08299 | 6145 | 2.40E-09 | 291 | 101 |
| TF | Factor: C-Jun; motif: TGACTC | TF:M03541 | 9625 | 2.50E-09 | 291 | 136 |
| GO:BP | negative regulation of developmental process | GO:0051093 | 804 | 2.60E-09 | 291 | 31 |
| TF | Factor: Zbtb44; motif: CKGTGA; match class: 1 | TF:M05405_1 | 5881 | 2.70E-09 | 291 | 98 |
| TF | Factor: E2F-2; motif: GCGCGCGCNCS | TF:M11529 | 14928 | 2.80E-09 | 291 | 183 |
| TF | Factor: E2F-3; motif: NTTTTGGCGCCAAAAN; match class: 1 | TF:M11526_1 | 5975 | 2.80E-09 | 291 | 99 |
| TF | Factor: LRF; motif: NCGACCACCGN | TF:M12231 | 10281 | 2.90E-09 | 291 | 142 |
| TF | Factor: E2F-3; motif: NNTTTTGGCGCCAAAACT; match class: 1 | TF:M03962_1 | 5799 | 3.00E-09 | 291 | 97 |
| TF | Factor: PPARgamma:RXRalpha,; motif: AAGTAGGTCACNGTGACCYACTT | TF:M00515 | 6738 | 3.10E-09 | 291 | 107 |
| TF | Factor: SMAD3; motif: CAGACAS | TF:M07429 | 5352 | 3.30E-09 | 291 | 92 |
| GO:BP | positive regulation of signal transduction | GO:0009967 | 1394 | 3.40E-09 | 291 | 41 |
| TF | Factor: TFII-I; motif: RGAGGKAGG | TF:M00706 | 5174 | 3.40E-09 | 291 | 90 |
| TF | Factor: p300; motif: NNNGGGAGTNNNNS | TF:M00033 | 7624 | 3.40E-09 | 291 | 116 |
| TF | Factor: AP-2alpha; motif: NNNGSCCTGRGGSN | TF:M04896 | 5266 | 3.50E-09 | 291 | 91 |
| GO:BP | cytokine-mediated signaling pathway | GO:0019221 | 430 | 3.60E-09 | 291 | 23 |
| GO:BP | regulation of cell communication | GO:0010646 | 3111 | 3.60E-09 | 291 | 65 |
| TF | Factor: RelA-p65; motif: BCWGGGRANNK | TF:M04811 | 6469 | 3.60E-09 | 291 | 104 |
| TF | Factor: ER-beta; motif: RGGTCASCNTGMCCY | TF:M09910 | 8635 | 3.70E-09 | 291 | 126 |
| TF | Factor: BTEB3; motif: BNRNGGGAGGNGT | TF:M01865 | 9056 | 4.00E-09 | 291 | 130 |
| TF | Factor: p54NRB; motif: GRNNNMGGATGRMNNCGGA | TF:M07611 | 6106 | 4.10E-09 | 291 | 100 |
| TF | Factor: SATB1; motif: NTTTAT | TF:M03564 | 13565 | 4.20E-09 | 291 | 171 |
| TF | Factor: c-Ets-2; motif: NACCGGAAGYRCTTCCGGTN | TF:M11417 | 12989 | 4.20E-09 | 291 | 166 |
| GO:BP | regulation of signaling | GO:0023051 | 3125 | 4.40E-09 | 291 | 65 |
| TF | Factor: GR; motif: RGNACANMNTGTNCY | TF:M09941 | 4931 | 4.40E-09 | 291 | 87 |
| TF | Factor: KLF4; motif: NNCCCCACCCNN | TF:M12489 | 5471 | 4.50E-09 | 291 | 93 |
| TF | Factor: GLI4; motif: RGGCCTTGAATGCCANGCYMA | TF:M10514 | 11095 | 4.60E-09 | 291 | 149 |
| TF | Factor: SP2; motif: NNRRCCAATSRGNRNSNGSN | TF:M12668 | 10569 | 5.30E-09 | 291 | 144 |
| TF | Factor: Sox-4; motif: AACAAA | TF:M03849 | 12223 | 5.30E-09 | 291 | 159 |
| TF | Factor: VDR; motif: GGGKNARNRRGGWSA | TF:M00444 | 9297 | 5.30E-09 | 291 | 132 |
| GO:BP | regulation of signal transduction | GO:0009966 | 2751 | 5.50E-09 | 291 | 60 |
| TF | Factor: TBX2; motif: NNNGTGTSNN | TF:M12026 | 6418 | 5.50E-09 | 291 | 103 |
| GO:BP | regulation of cell population proliferation | GO:0042127 | 1482 | 5.80E-09 | 291 | 42 |
| TF | Factor: TFAP2A; motif: NGCCCYNNGGGCN | TF:M04148 | 9313 | 6.00E-09 | 291 | 132 |
| TF | Factor: Fli-1; motif: NACCGGAWWTCCGGTY | TF:M11399 | 11690 | 6.40E-09 | 291 | 154 |
| TF | Factor: ZNF586; motif: CAGGCCYRGAGG; match class: 1 | TF:M10491_1 | 6912 | 6.50E-09 | 291 | 108 |
| GO:CC | intracellular membrane-bounded organelle | GO:0043231 | 11785 | 6.60E-09 | 291 | 152 |
| TF | Factor: NFATc3; motif: GGAAAA; match class: 1 | TF:M01886_1 | 9439 | 7.20E-09 | 291 | 133 |
| TF | Factor: NFATc2; motif: GGAAAA; match class: 1 | TF:M03555_1 | 9439 | 7.20E-09 | 291 | 133 |
| TF | Factor: NFATc2; motif: GGAAAA; match class: 1 | TF:M01281_1 | 9439 | 7.20E-09 | 291 | 133 |
| TF | Factor: NR1B2; motif: NTGACCY | TF:M02111 | 8513 | 7.40E-09 | 291 | 124 |
| TF | Factor: ZF5; motif: NRNGNGCGCGCWN | TF:M00333 | 13999 | 7.60E-09 | 291 | 174 |
| TF | Factor: VDR,; motif: RRTGNMCYTNNTGAMCCNYNT | TF:M00966 | 8419 | 7.90E-09 | 291 | 123 |
| TF | Factor: AP-2gamma; motif: GCCYNCRGSN | TF:M03811 | 8219 | 8.00E-09 | 291 | 121 |
| GO:BP | regulation of gene expression | GO:0010468 | 4415 | 8.20E-09 | 291 | 80 |
| TF | Factor: ESE-1; motif: SATKGCGGATGCN | TF:M11381 | 13896 | 8.40E-09 | 291 | 173 |
| TF | Factor: RORbeta; motif: TGACCYA | TF:M01722 | 5168 | 8.40E-09 | 291 | 89 |
| TF | Factor: ETF; motif: CCCCGCCCCYN; match class: 1 | TF:M07039_1 | 12626 | 8.40E-09 | 291 | 162 |
| GO:BP | circulatory system process | GO:0003013 | 539 | 8.50E-09 | 291 | 25 |
| GO:BP | homeostatic process | GO:0042592 | 1631 | 8.60E-09 | 291 | 44 |
| GO:BP | positive regulation of cell communication | GO:0010647 | 1569 | 9.30E-09 | 291 | 43 |
| GO:BP | positive regulation of signaling | GO:0023056 | 1573 | 1.00E-08 | 291 | 43 |
| TF | Factor: CRX; motif: YTAATC | TF:M01712 | 9480 | 1.00E-08 | 291 | 133 |
| TF | Factor: E2F-3:TBR2; motif: ANGTGYKANGGCGCSTTNNCRNNT; match class: 1 | TF:M08207_1 | 14160 | 1.00E-08 | 291 | 175 |
| GO:BP | response to molecule of bacterial origin | GO:0002237 | 292 | 1.10E-08 | 291 | 19 |
| TF | Factor: PPARalpha:RXRalpha; motif: NNRGGTCATWGGGGTSANG | TF:M00518 | 8354 | 1.10E-08 | 291 | 122 |
| TF | Factor: ETV4; motif: NCAGGAAGNN; match class: 1 | TF:M12556_1 | 3399 | 1.10E-08 | 291 | 68 |
| REAC | Interleukin-4 and Interleukin-13 signaling | REAC:R-HSA-6785807 | 97 | 1.20E-08 | 291 | 12 |
| TF | Factor: CP2/LBP-1c/LSF; motif: GCTGGNTNGNNCYNG | TF:M00947 | 7463 | 1.20E-08 | 291 | 113 |
| TF | Factor: Erg; motif: NACCGGATATCCGGTN | TF:M11397 | 10892 | 1.20E-08 | 291 | 146 |
| TF | Factor: GATA-3; motif: AGATAA | TF:M01878 | 8472 | 1.20E-08 | 291 | 123 |
| TF | Factor: ZNF273; motif: GAGAGGAGCTAC; match class: 1 | TF:M10460_1 | 5118 | 1.30E-08 | 291 | 88 |
| TF | Factor: HOXA13; motif: ATAAMA | TF:M01292 | 11353 | 1.40E-08 | 291 | 150 |
| TF | Factor: ZFP14; motif: SCNNYCCNGNNSCTSCNC; match class: 1 | TF:M12694_1 | 4775 | 1.50E-08 | 291 | 84 |
| TF | Factor: Elk-1; motif: GGAAGN; match class: 1 | TF:M03819_1 | 6527 | 1.60E-08 | 291 | 103 |
| TF | Factor: BEN; motif: CAGCGRNV; match class: 1 | TF:M01240_1 | 12381 | 1.70E-08 | 291 | 159 |
| GO:BP | regulation of immune system process | GO:0002682 | 1217 | 1.80E-08 | 291 | 37 |
| REAC | Binding and Uptake of Ligands by Scavenger Receptors | REAC:R-HSA-2173782 | 41 | 1.80E-08 | 291 | 9 |
| TF | Factor: c-Ets-2; motif: NACCGGAAGYRCTTCCGGTN; match class: 1 | TF:M11417_1 | 12734 | 1.80E-08 | 291 | 162 |
| TF | Factor: Cdx-1; motif: TTTATK | TF:M02086 | 12282 | 1.90E-08 | 291 | 158 |
| TF | Factor: c-Myb; motif: NNWGRCAGTTRN | TF:M09990 | 9243 | 1.90E-08 | 291 | 130 |
| TF | Factor: PMX1; motif: TAATHA | TF:M03560 | 11838 | 1.90E-08 | 291 | 154 |
| TF | Factor: CP2; motif: NNNNCCAGNCNN; match class: 1 | TF:M07602_1 | 4106 | 2.00E-08 | 291 | 76 |
| TF | Factor: AREB6; motif: NNYNYACCTGWVT | TF:M00412 | 3939 | 2.00E-08 | 291 | 74 |
| TF | Factor: REST; motif: GGACAGCKC | TF:M04726 | 5256 | 2.10E-08 | 291 | 89 |
| TF | Factor: myogenin; motif: RGCAGSTG | TF:M00712 | 6086 | 2.10E-08 | 291 | 98 |
| TF | Factor: Tal-1; motif: CAGATGG | TF:M03804 | 4459 | 2.20E-08 | 291 | 80 |
| TF | Factor: SZF1-1; motif: CCAGGGTAWCAGCNG | TF:M01109 | 3781 | 2.30E-08 | 291 | 72 |
| TF | Factor: LF-A1; motif: GGGSTCWR; match class: 1 | TF:M00646_1 | 5629 | 2.30E-08 | 291 | 93 |
| GO:BP | negative regulation of nitrogen compound metabolic process | GO:0051172 | 2106 | 2.60E-08 | 291 | 50 |
| GO:BP | ossification | GO:0001503 | 388 | 2.70E-08 | 291 | 21 |
| KEGG | Cytokine-cytokine receptor interaction | KEGG:04060 | 213 | 2.80E-08 | 291 | 15 |
| TF | Factor: GR; motif: RGWACATWAYGTWCY | TF:M11846 | 5101 | 2.80E-08 | 291 | 87 |
| GO:BP | transport | GO:0006810 | 4269 | 3.00E-08 | 291 | 77 |
| TF | Factor: NF-1C; motif: NTTGGCNNNNTGCCARN | TF:M11728 | 2233 | 3.00E-08 | 291 | 52 |
| TF | Factor: SMAD; motif: TGTCTGNNN | TF:M08897 | 3891 | 3.10E-08 | 291 | 73 |
| TF | Factor: AP-2; motif: SNNNCCNCAGGCN | TF:M00915 | 8995 | 3.10E-08 | 291 | 127 |
| TF | Factor: VDR; motif: NRGGTCANNGRGKTCA | TF:M10106 | 6125 | 3.10E-08 | 291 | 98 |
| GO:BP | positive regulation of gene expression | GO:0010628 | 1001 | 3.20E-08 | 291 | 33 |
| TF | Factor: Elk-1; motif: CTTCCKG; match class: 1 | TF:M07252_1 | 3080 | 3.40E-08 | 291 | 63 |
| TF | Factor: c-Ets-2; motif: CTTCCTG; match class: 1 | TF:M01207_1 | 3080 | 3.40E-08 | 291 | 63 |
| TF | Factor: NF-E2; motif: TGCTGAGTCAY | TF:M00037 | 5577 | 3.50E-08 | 291 | 92 |
| TF | Factor: AR; motif: NGNACANNNTGTTCYNN | TF:M09589 | 4327 | 3.50E-08 | 291 | 78 |
| TF | Factor: E2F1; motif: GSGCGGGAAN | TF:M12597 | 13064 | 3.80E-08 | 291 | 164 |
| TF | Factor: E2F4; motif: YCCCGCCNCNNSSNNSNN; match class: 1 | TF:M12599_1 | 14589 | 3.90E-08 | 291 | 177 |
| GO:BP | response to lipopolysaccharide | GO:0032496 | 277 | 4.10E-08 | 291 | 18 |
| GO:BP | negative regulation of response to stimulus | GO:0048585 | 1510 | 4.10E-08 | 291 | 41 |
| TF | Factor: FOXO1A:Net; motif: RWMAACAGGAAGTN | TF:M08302 | 7909 | 4.20E-08 | 291 | 116 |
| TF | Factor: PPARALPHA:RXR-ALPHA; motif: AWNTRGGTNAAAGGTCAN | TF:M08960 | 10094 | 4.50E-08 | 291 | 137 |
| TF | Factor: MIF-1; motif: NNGTTGCWWGGYAACNGS | TF:M00279 | 9883 | 4.60E-08 | 291 | 135 |
| TF | Factor: Erm:E2A; motif: CASGTGNNNCGGAAGNN | TF:M08571 | 9890 | 4.80E-08 | 291 | 135 |
| GO:BP | muscle tissue development | GO:0060537 | 359 | 5.00E-08 | 291 | 20 |
| TF | Factor: c-Rel; motif: NRNRGGGRAATKCCA | TF:M10046 | 4101 | 5.00E-08 | 291 | 75 |
| TF | Factor: GR; motif: NNNNNNNGKACNNNNTGTTCTNNNNNN | TF:M00955 | 4102 | 5.10E-08 | 291 | 75 |
| TF | Factor: E2F-1:HES-7; motif: GGCRCGTGSYNNWNGGCGCSM; match class: 1 | TF:M08525_1 | 14157 | 5.10E-08 | 291 | 173 |
| TF | Factor: LRH-1; motif: TCAAGGTCRYGACCTTGR; match class: 1 | TF:M11828_1 | 6856 | 5.60E-08 | 291 | 105 |
| TF | Factor: ZNF692; motif: SYNGGSCCCASCCNC | TF:M09734 | 10664 | 5.70E-08 | 291 | 142 |
| TF | Factor: Tbx5; motif: TNAGGTGTKV | TF:M01020 | 9490 | 5.80E-08 | 291 | 131 |
| GO:BP | response to growth factor | GO:0070848 | 640 | 5.90E-08 | 291 | 26 |
| GO:BP | establishment of localization | GO:0051234 | 4416 | 6.00E-08 | 291 | 78 |
| REAC | Cytokine Signaling in Immune system | REAC:R-HSA-1280215 | 634 | 6.00E-08 | 291 | 25 |
| GO:BP | regulation of cell differentiation | GO:0045595 | 1400 | 6.30E-08 | 291 | 39 |
| TF | Factor: FXR:RXR-ALPHA; motif: NRGGTCANRGGKN | TF:M08955 | 8470 | 6.60E-08 | 291 | 121 |
| TF | Factor: GR; motif: RGWACATWATGTWCY | TF:M11847 | 4559 | 6.60E-08 | 291 | 80 |
| TF | Factor: ESRRA; motif: CAAGGTCANNYSAAGGTCA | TF:M04458 | 9722 | 6.80E-08 | 291 | 133 |
| TF | Factor: AP-2alpha; motif: NGCCYSNNGSN | TF:M01857 | 8679 | 6.80E-08 | 291 | 123 |
| TF | Factor: AP-4:ER81; motif: RSCGGAAGCAGSTGNN | TF:M08443 | 9517 | 7.10E-08 | 291 | 131 |
| TF | Factor: ZNF432; motif: NCAGNRCCNSRGRCAGC | TF:M12704 | 9203 | 7.10E-08 | 291 | 128 |
| GO:BP | embryo development | GO:0009790 | 976 | 7.50E-08 | 291 | 32 |
| TF | Factor: AML1; motif: TGTGGT | TF:M00751 | 10268 | 7.50E-08 | 291 | 138 |
| TF | Factor: AML1a; motif: TGTGGT | TF:M00271 | 10268 | 7.50E-08 | 291 | 138 |
| TF | Factor: AML1; motif: TGTGGT | TF:M02084 | 10268 | 7.50E-08 | 291 | 138 |
| GO:BP | positive regulation of cellular metabolic process | GO:0031325 | 2936 | 7.80E-08 | 291 | 60 |
| TF | Factor: GR; motif: RGRACATTNTGTYC | TF:M04750 | 4487 | 7.90E-08 | 291 | 79 |
| GO:BP | regulation of localization | GO:0032879 | 2549 | 8.00E-08 | 291 | 55 |
| TF | Factor: Elk-1:Pax-5; motif: ACCGGAACYACGCWTSANYG | TF:M08225 | 12715 | 8.00E-08 | 291 | 160 |
| TF | Factor: DATF1; motif: SNGGRRGCWGNGGG | TF:M09724 | 7588 | 8.20E-08 | 291 | 112 |
| GO:BP | skeletal system development | GO:0001501 | 457 | 8.40E-08 | 291 | 22 |
| GO:BP | regulation of cellular metabolic process | GO:0031323 | 5347 | 8.60E-08 | 291 | 88 |
| TF | Factor: THAP1; motif: YTGCCCNNA; match class: 1 | TF:M07407_1 | 3639 | 8.60E-08 | 291 | 69 |
| TF | Factor: GR; motif: RGWACATWATGTWCY; match class: 1 | TF:M11847_1 | 3311 | 8.70E-08 | 291 | 65 |
| TF | Factor: STAT5A; motif: TTCCNRGAANNNNNNTTCCNNGRR | TF:M00460 | 6618 | 8.90E-08 | 291 | 102 |
| GO:BP | negative regulation of cellular metabolic process | GO:0031324 | 2256 | 9.10E-08 | 291 | 51 |
| TF | Factor: TFAP2A; motif: NGCCCYNNGGGCN; match class: 1 | TF:M04148_1 | 8927 | 9.50E-08 | 291 | 125 |
| TF | Factor: Smad4; motif: NCAGACAN | TF:M07368 | 3322 | 1.00E-07 | 291 | 65 |
| GO:BP | regulation of angiogenesis | GO:0045765 | 256 | 1.10E-07 | 291 | 17 |
| GO:CC | blood microparticle | GO:0072562 | 94 | 1.10E-07 | 291 | 11 |
| TF | Factor: Sohlh2; motif: NNCACGTGNN | TF:M11075 | 10531 | 1.10E-07 | 291 | 140 |
| TF | Factor: Fli-1; motif: NACCGGATATCCGGTN | TF:M11401 | 10861 | 1.10E-07 | 291 | 143 |
| TF | Factor: DPF2; motif: NYCACYTCCYCNYYCY | TF:M09760 | 7424 | 1.10E-07 | 291 | 110 |
| TF | Factor: PEA3; motif: ACWTCCK | TF:M00655 | 8027 | 1.10E-07 | 291 | 116 |
| TF | Factor: pax-6; motif: NYACGCNYSANYGMNCN | TF:M11882 | 14157 | 1.10E-07 | 291 | 172 |
| GO:BP | negative regulation of molecular function | GO:0044092 | 935 | 1.20E-07 | 291 | 31 |
| GO:BP | response to organonitrogen compound | GO:0010243 | 937 | 1.20E-07 | 291 | 31 |
| REAC | Signaling by Interleukins | REAC:R-HSA-449147 | 411 | 1.20E-07 | 291 | 20 |
| TF | Factor: rfx3:SREBP-2; motif: ATGGYAACRTCACGTGAY | TF:M08412 | 9163 | 1.20E-07 | 291 | 127 |
| GO:BP | regulation of vasculature development | GO:1901342 | 260 | 1.30E-07 | 291 | 17 |
| KEGG | Staphylococcus aureus infection | KEGG:05150 | 60 | 1.30E-07 | 291 | 9 |
| TF | Factor: CTCF; motif: ACCAGGKGGC | TF:M04727 | 5802 | 1.30E-07 | 291 | 93 |
| TF | Factor: C/EBP; motif: NTTRCNNAANNN | TF:M00912 | 4188 | 1.40E-07 | 291 | 75 |
| GO:BP | apoptotic signaling pathway | GO:0097190 | 566 | 1.50E-07 | 291 | 24 |
| GO:BP | cellular response to growth factor stimulus | GO:0071363 | 616 | 1.50E-07 | 291 | 25 |
| TF | Factor: CDX-2; motif: TTTATN | TF:M02087 | 8261 | 1.50E-07 | 291 | 118 |
| TF | Factor: TFAP2C; motif: NGCCCNNRGGCA | TF:M04152 | 7062 | 1.50E-07 | 291 | 106 |
| TF | Factor: HES-5; motif: NCACACKY | TF:M11069 | 9505 | 1.50E-07 | 291 | 130 |
| TF | Factor: DEC1; motif: NCNCACRTGNSC | TF:M08870 | 6965 | 1.50E-07 | 291 | 105 |
| TF | Factor: Spic; motif: NGNGGAASTN | TF:M02077 | 7359 | 1.50E-07 | 291 | 109 |
| TF | Factor: E2F-1; motif: NGGGCGGGARV | TF:M07206 | 13501 | 1.50E-07 | 291 | 166 |
| GO:BP | mesenchyme development | GO:0060485 | 263 | 1.60E-07 | 291 | 17 |
| TF | Factor: TIEG1; motif: GGSGGKGNNN | TF:M08905 | 4638 | 1.60E-07 | 291 | 80 |
| TF | Factor: GLI; motif: NTGGGTGGTN | TF:M07292 | 5098 | 1.80E-07 | 291 | 85 |
| TF | Factor: NRL; motif: NNNNTGCTGAC | TF:M04224 | 3869 | 1.80E-07 | 291 | 71 |
| TF | Factor: CPBP; motif: GNNRGGGHGGGGNNGGGRN | TF:M09973 | 10173 | 1.80E-07 | 291 | 136 |
| GO:BP | muscle structure development | GO:0061061 | 573 | 1.90E-07 | 291 | 24 |
| GO:BP | regulation of body fluid levels | GO:0050878 | 345 | 1.90E-07 | 291 | 19 |
| TF | Factor: ZNF549; motif: NTGCTKYCMW | TF:M12355 | 5656 | 1.90E-07 | 291 | 91 |
| TF | Factor: AP2; motif: GCCYGSGGSN | TF:M08867 | 9433 | 1.90E-07 | 291 | 129 |
| TF | Factor: Spz1; motif: DNNGGRGGGWWNNNN | TF:M00446 | 7790 | 1.90E-07 | 291 | 113 |
| TF | Factor: Fli-1; motif: NACCGGATATCCGGTN; match class: 1 | TF:M11401_1 | 10722 | 1.90E-07 | 291 | 141 |
| GO:BP | response to nitrogen compound | GO:1901698 | 1015 | 2.00E-07 | 291 | 32 |
| TF | Factor: ER-alpha; motif: AGGTCASMNTGACCY | TF:M09909 | 5943 | 2.00E-07 | 291 | 94 |
| TF | Factor: E2F-3:FOXO6; motif: NAATGACACGCGCCCMC | TF:M08209 | 12393 | 2.00E-07 | 291 | 156 |
| GO:MF | structural molecule activity | GO:0005198 | 657 | 2.20E-07 | 291 | 25 |
| GO:BP | negative regulation of metabolic process | GO:0009892 | 2853 | 2.20E-07 | 291 | 58 |
| TF | Factor: ZABC1; motif: ATTCCNAC | TF:M01306 | 8006 | 2.20E-07 | 291 | 115 |
| TF | Factor: Hey2; motif: NNCACGYGNN | TF:M11052 | 11397 | 2.20E-07 | 291 | 147 |
| TF | Factor: Zic1; motif: KGGGTGGTC; match class: 1 | TF:M00448_1 | 3888 | 2.20E-07 | 291 | 71 |
| TF | Factor: HSF2; motif: NGAANNWTCK | TF:M00147 | 4941 | 2.20E-07 | 291 | 83 |
| TF | Factor: E2F2; motif: NNTTTTGGCGCCAAAAWN | TF:M03959 | 7017 | 2.30E-07 | 291 | 105 |
| GO:BP | regulation of cell migration | GO:0030334 | 847 | 2.40E-07 | 291 | 29 |
| TF | Factor: HNRPUL1; motif: NCNCAGN; match class: 1 | TF:M09746_1 | 6727 | 2.40E-07 | 291 | 102 |
| TF | Factor: Six-2; motif: NNGTAWCRNN | TF:M11032 | 9253 | 2.40E-07 | 291 | 127 |
| TF | Factor: CTCF; motif: CANNWRRTGGCAG | TF:M04745 | 7022 | 2.40E-07 | 291 | 105 |
| TF | Factor: Erg; motif: NACCGGATATCCGGTN; match class: 1 | TF:M11397_1 | 10645 | 2.40E-07 | 291 | 140 |
| GO:BP | cellular response to organonitrogen compound | GO:0071417 | 582 | 2.60E-07 | 291 | 24 |
| TF | Factor: DLX2; motif: AMSMCTGACKG | TF:M08873 | 6450 | 2.60E-07 | 291 | 99 |
| GO:BP | regulation of cell adhesion | GO:0030155 | 687 | 2.70E-07 | 291 | 26 |
| GO:CC | contractile fiber | GO:0043292 | 229 | 2.80E-07 | 291 | 15 |
| GO:BP | cellular response to nitrogen compound | GO:1901699 | 636 | 2.90E-07 | 291 | 25 |
| GO:BP | connective tissue development | GO:0061448 | 236 | 2.90E-07 | 291 | 16 |
| TF | Factor: AP-2; motif: MKCCCSCNGGCG | TF:M00189 | 10020 | 2.90E-07 | 291 | 134 |
| TF | Factor: MAZ; motif: GGGGGAGGGGGNGRGRRRGNRG | TF:M09984 | 8861 | 2.90E-07 | 291 | 123 |
| GO:BP | regulation of cellular component movement | GO:0051270 | 972 | 3.00E-07 | 291 | 31 |
| TF | Factor: C/EBPbeta; motif: TKNNGCAANN | TF:M01896 | 3827 | 3.00E-07 | 291 | 70 |
| TF | Factor: DB1; motif: GGRRRRGRRGGAGGGGGNGRRR | TF:M10107 | 5428 | 3.00E-07 | 291 | 88 |
| TF | Factor: Sox-4; motif: NYCTTTGTYYYN | TF:M10069 | 6179 | 3.10E-07 | 291 | 96 |
| TF | Factor: NR1H4; motif: NNAATGACCNN | TF:M09777 | 3246 | 3.10E-07 | 291 | 63 |
| REAC | Signal Transduction | REAC:R-HSA-162582 | 2365 | 3.20E-07 | 291 | 50 |
| TF | Factor: STAT1; motif: NTTCCNGGA | TF:M04721 | 6860 | 3.20E-07 | 291 | 103 |
| TF | Factor: JUNB:C-JUN; motif: KRTGACGTCATN | TF:M08945 | 8056 | 3.30E-07 | 291 | 115 |
| TF | Factor: Tax/CREB; motif: RTGACGCATAYCCCC | TF:M00115 | 8263 | 3.40E-07 | 291 | 117 |
| REAC | Assembly of collagen fibrils and other multimeric structures | REAC:R-HSA-2022090 | 56 | 3.50E-07 | 291 | 9 |
| TF | Factor: Sall1; motif: NGGTCCKRGKRA | TF:M05467 | 3757 | 3.50E-07 | 291 | 69 |
| TF | Factor: AR; motif: AGAACANNNTGTTCT; match class: 1 | TF:M08907_1 | 1804 | 3.50E-07 | 291 | 44 |
| TF | Factor: ZNF511; motif: GGRRGRGGCWGNG | TF:M09738 | 9835 | 3.50E-07 | 291 | 132 |
| GO:BP | regulation of primary metabolic process | GO:0080090 | 5129 | 3.70E-07 | 291 | 84 |
| GO:BP | blood coagulation | GO:0007596 | 205 | 3.70E-07 | 291 | 15 |
| TF | Factor: Sox-2; motif: NNCCTTTGTNYYN | TF:M10067 | 5172 | 3.70E-07 | 291 | 85 |
| TF | Factor: AP-2gamma; motif: GCCYNNGGS; match class: 1 | TF:M00470_1 | 5364 | 4.00E-07 | 291 | 87 |
| TF | Factor: IRF-2; motif: NAANYGAAASYR | TF:M08775 | 7180 | 4.00E-07 | 291 | 106 |
| REAC | Collagen degradation | REAC:R-HSA-1442490 | 57 | 4.10E-07 | 291 | 9 |
| TF | Factor: VDR; motif: GRGTTCATYGRGTTCA | TF:M04498 | 4463 | 4.10E-07 | 291 | 77 |
| TF | Factor: NF-kappaB; motif: GGGGATYCCC | TF:M00051 | 5840 | 4.40E-07 | 291 | 92 |
| TF | Factor: AP-2gamma:Max; motif: NNSCCYNRGGSNNNNCACGTGN | TF:M08664 | 2105 | 4.40E-07 | 291 | 48 |
| TF | Factor: ER71:Pax-5; motif: ACCGGAACYACGCWTSANTG | TF:M08266 | 13080 | 4.40E-07 | 291 | 161 |
| KEGG | Malaria | KEGG:05144 | 48 | 4.50E-07 | 291 | 8 |
| GO:BP | coagulation | GO:0050817 | 208 | 4.60E-07 | 291 | 15 |
| TF | Factor: NURR1; motif: YRRCCTT | TF:M01269 | 5012 | 4.60E-07 | 291 | 83 |
| TF | Factor: c-Rel; motif: NGGGAATYTCCN | TF:M03545 | 4836 | 4.80E-07 | 291 | 81 |
| GO:BP | regulation of nitrogen compound metabolic process | GO:0051171 | 4981 | 5.20E-07 | 291 | 82 |
| TF | Factor: FOXM1; motif: TRTTTATNN | TF:M08883 | 5673 | 5.30E-07 | 291 | 90 |
| TF | Factor: ZBRK1; motif: NGNNNGGTNAWAAAARRGCNG | TF:M10468 | 9361 | 5.30E-07 | 291 | 127 |
| GO:BP | positive regulation of immune system process | GO:0002684 | 764 | 5.40E-07 | 291 | 27 |
| GO:BP | hemostasis | GO:0007599 | 211 | 5.60E-07 | 291 | 15 |
| TF | Factor: DBP; motif: TTRCATAANN | TF:M07038 | 4582 | 5.60E-07 | 291 | 78 |
| TF | Factor: BTEB2; motif: WGGGTGKGGCNGGN | TF:M09972 | 8433 | 5.70E-07 | 291 | 118 |
| TF | Factor: RUNX3; motif: NRACCGCAAACCGCAN | TF:M04109 | 8951 | 5.70E-07 | 291 | 123 |
| TF | Factor: NF-1; motif: NNTTGGCNNNNNNCCNNN | TF:M00193 | 4407 | 5.70E-07 | 291 | 76 |
| TF | Factor: MTF-1; motif: NTTTTGCACACGGCNYN | TF:M12279 | 5776 | 5.80E-07 | 291 | 91 |
| TF | Factor: NF-kappaB; motif: AGGGGAWTCCCCT | TF:M04054 | 2656 | 5.90E-07 | 291 | 55 |
| TF | Factor: AP-2alphaA; motif: ANMGCCTNAGGCKNT | TF:M01047 | 7728 | 6.00E-07 | 291 | 111 |
| TF | Factor: AP-2alphaA; motif: ANMGCCTNAGGCKNT; match class: 1 | TF:M01047_1 | 7728 | 6.00E-07 | 291 | 111 |
| TF | Factor: PURBETA; motif: NCWGRGGCTGGGG; match class: 1 | TF:M12726_1 | 2201 | 6.20E-07 | 291 | 49 |
| TF | Factor: SREBP-1; motif: CACSCCA; match class: 1 | TF:M00749_1 | 2428 | 6.20E-07 | 291 | 52 |
| TF | Factor: HSF2; motif: NGAANNWTCK; match class: 1 | TF:M00147_1 | 3725 | 6.50E-07 | 291 | 68 |
| TF | Factor: AP-4; motif: AWCAGCTGWT | TF:M11206 | 6263 | 6.50E-07 | 291 | 96 |
| TF | Factor: AP-4; motif: AWCAGCTGWT; match class: 1 | TF:M11206_1 | 6263 | 6.50E-07 | 291 | 96 |
| GO:BP | leukocyte migration | GO:0050900 | 329 | 6.70E-07 | 291 | 18 |
| GO:BP | cellular response to lipid | GO:0071396 | 511 | 7.00E-07 | 291 | 22 |
| TF | Factor: TEF-3:Elk-1; motif: RMATWCCGGAWGT | TF:M08418 | 10468 | 7.00E-07 | 291 | 137 |
| TF | Factor: c-Ets-1; motif: RCAGGAAGTGNNTNS | TF:M00339 | 5893 | 7.10E-07 | 291 | 92 |
| TF | Factor: Elf5; motif: CSMGGAARNN | TF:M02057 | 9296 | 7.20E-07 | 291 | 126 |
| GO:BP | enzyme linked receptor protein signaling pathway | GO:0007167 | 889 | 7.30E-07 | 291 | 29 |
| TF | Factor: RelA-p65; motif: GGGANTTTCCNN | TF:M03563 | 4079 | 7.30E-07 | 291 | 72 |
| GO:BP | renal system development | GO:0072001 | 291 | 7.70E-07 | 291 | 17 |
| TF | Factor: Fli-1; motif: RCAGGAAGTGR | TF:M07089 | 4264 | 8.20E-07 | 291 | 74 |
| GO:BP | response to toxic substance | GO:0009636 | 217 | 8.30E-07 | 291 | 15 |
| GO:CC | collagen trimer | GO:0005581 | 87 | 8.30E-07 | 291 | 10 |
| TF | Factor: CP2; motif: NTGNCTGGNN | TF:M03868 | 4006 | 8.50E-07 | 291 | 71 |
| TF | Factor: HSF1; motif: TCYAGAANNTTC | TF:M07259 | 4006 | 8.50E-07 | 291 | 71 |
| TF | Factor: AP-4:Max; motif: NCAGCTGNNNNNNNCACGTGN; match class: 1 | TF:M08674_1 | 4802 | 8.50E-07 | 291 | 80 |
| GO:MF | extracellular matrix structural constituent conferring tensile strength | GO:0030020 | 40 | 8.70E-07 | 291 | 8 |
| TF | Factor: CP2; motif: NRRRCCGGCYNRARCCGGNN | TF:M12590 | 10284 | 8.90E-07 | 291 | 135 |
| GO:BP | positive regulation of cell differentiation | GO:0045597 | 782 | 9.00E-07 | 291 | 27 |
| TF | Factor: NR1B1; motif: NRGGTCANNRGGTCAN | TF:M11795 | 6883 | 9.00E-07 | 291 | 102 |
| REAC | Collagen chain trimerization | REAC:R-HSA-8948216 | 43 | 9.10E-07 | 291 | 8 |
| TF | Factor: ZNF300; motif: SWGGGGGSN; match class: 1 | TF:M07486_1 | 6786 | 9.10E-07 | 291 | 101 |
| GO:BP | regulation of cell motility | GO:2000145 | 898 | 9.20E-07 | 291 | 29 |
| TF | Factor: ZNF436; motif: TCCTCCAGGAAGCCY; match class: 1 | TF:M10477_1 | 7782 | 9.20E-07 | 291 | 111 |
| REAC | Collagen formation | REAC:R-HSA-1474290 | 85 | 9.30E-07 | 291 | 10 |
| MIRNA | hsa-miR-26b-5p | MIRNA:hsa-miR-26b-5p | 1739 | 9.60E-07 | 291 | 41 |
| TF | Factor: AR; motif: AGAACANNNTGTTCT | TF:M08907 | 1863 | 9.70E-07 | 291 | 44 |
| TF | Factor: GR; motif: AGAACAN | TF:M07355 | 4913 | 1.00E-06 | 291 | 81 |
| GO:BP | striated muscle tissue development | GO:0014706 | 340 | 1.10E-06 | 291 | 18 |
| TF | Factor: FOXO1A:ETV7; motif: NWMAACAGGAMNNNCTTCCNN; match class: 1 | TF:M08304_1 | 6512 | 1.10E-06 | 291 | 98 |
| TF | Factor: E2F-4; motif: GCGGGAAANA | TF:M02090 | 11183 | 1.10E-06 | 291 | 143 |
| TF | Factor: Nrf2; motif: NNTGACTCAGCAN | TF:M07265 | 4114 | 1.10E-06 | 291 | 72 |
| GO:BP | sensory organ development | GO:0007423 | 525 | 1.20E-06 | 291 | 22 |
| TF | Factor: E2F-3:T-bet; motif: AGGTGTNANGGCGCST | TF:M08529 | 10334 | 1.30E-06 | 291 | 135 |
| TF | Factor: HNF4A; motif: NNCAAAGTCCANN | TF:M12503 | 2555 | 1.30E-06 | 291 | 53 |
| TF | Factor: ER-beta; motif: NRGGTCANKSTGACCTNN | TF:M09610 | 5122 | 1.30E-06 | 291 | 83 |
| GO:MF | protein-containing complex binding | GO:0044877 | 1204 | 1.40E-06 | 291 | 33 |
| GO:BP | muscle organ development | GO:0007517 | 303 | 1.40E-06 | 291 | 17 |
| TF | Factor: SRY; motif: TTGTTT; match class: 1 | TF:M03854_1 | 7628 | 1.40E-06 | 291 | 109 |
| TF | Factor: FOXO1A; motif: AAACAA; match class: 1 | TF:M03823_1 | 7628 | 1.40E-06 | 291 | 109 |
| TF | Factor: NR1B1; motif: NRGGNCRTGACCTN; match class: 1 | TF:M11796_1 | 4315 | 1.40E-06 | 291 | 74 |
| TF | Factor: c-ets-1; motif: GGAGTTG | TF:M04822 | 4763 | 1.40E-06 | 291 | 79 |
| TF | Factor: ERF:FOXO1A; motif: RTMAACAGGAARNS | TF:M08241 | 6745 | 1.50E-06 | 291 | 100 |
| TF | Factor: LHX3; motif: ATTAAW | TF:M02097 | 10034 | 1.50E-06 | 291 | 132 |
| TF | Factor: MAZ; motif: NKGGGAGGGGRGGR | TF:M02023 | 6650 | 1.50E-06 | 291 | 99 |
| TF | Factor: c-Ets-2; motif: NNNRGGAARNRRR | TF:M09912 | 4412 | 1.50E-06 | 291 | 75 |
| TF | Factor: FLI1; motif: ACCGGAAATCCGGT | TF:M03989 | 9505 | 1.50E-06 | 291 | 127 |
| GO:BP | chronic inflammatory response | GO:0002544 | 14 | 1.60E-06 | 291 | 6 |
| REAC | Collagen biosynthesis and modifying enzymes | REAC:R-HSA-1650814 | 66 | 1.60E-06 | 291 | 9 |
| TF | Factor: C/EBPalpha; motif: NGWVTKNKGYAAKNSAYA | TF:M00201 | 3296 | 1.60E-06 | 291 | 62 |
| TF | Factor: HMGIY; motif: NNKKNAWTTTNYTNN | TF:M01010 | 6852 | 1.60E-06 | 291 | 101 |
| TF | Factor: MOVO-B; motif: GNGGGGG | TF:M01104 | 9727 | 1.60E-06 | 291 | 129 |
| GO:BP | metabolic process | GO:0008152 | 11098 | 1.70E-06 | 291 | 141 |
| TF | Factor: Ik-3; motif: TNYTGGGAATACC | TF:M00088 | 5052 | 1.70E-06 | 291 | 82 |
| TF | Factor: ER-alpha; motif: NAGGTCACSGYGACCTN | TF:M11844 | 4779 | 1.70E-06 | 291 | 79 |
| TF | Factor: MAZ; motif: CCCTCCCYCYN | TF:M07297 | 2734 | 1.70E-06 | 291 | 55 |
| TF | Factor: JunB; motif: GATGACGTCAYC | TF:M11273 | 7663 | 1.80E-06 | 291 | 109 |
| TF | Factor: ZBTB44; motif: MACWGCAGS | TF:M12352 | 5619 | 1.80E-06 | 291 | 88 |
| KEGG | TNF signaling pathway | KEGG:04668 | 108 | 1.90E-06 | 291 | 10 |
| TF | Factor: TWIST1; motif: NNNCCAGATGTNN | TF:M09790 | 2428 | 1.90E-06 | 291 | 51 |
| TF | Factor: AR; motif: RGGWACAYNGTGTWCYN; match class: 1 | TF:M04453_1 | 3232 | 2.00E-06 | 291 | 61 |
| GO:MF | collagen binding | GO:0005518 | 64 | 2.10E-06 | 291 | 9 |
| TF | Factor: Elk-1:HOXA3; motif: ACCGGWAATKRNNTNWCNNATTAN | TF:M08220 | 10624 | 2.10E-06 | 291 | 137 |
| TF | Factor: FOXO1A:Elf-1; motif: NAGAAAACCGAANM | TF:M08295 | 10193 | 2.10E-06 | 291 | 133 |
| GO:BP | regulation of inflammatory response | GO:0050727 | 313 | 2.30E-06 | 291 | 17 |
| GO:CC | Z disc | GO:0030018 | 125 | 2.30E-06 | 291 | 11 |
| TF | Factor: AP2; motif: GCCYGSGGSN; match class: 1 | TF:M08867_1 | 4993 | 2.30E-06 | 291 | 81 |
| GO:BP | regulation of molecular function | GO:0065009 | 2797 | 2.40E-06 | 291 | 55 |
| GO:BP | regulation of locomotion | GO:0040012 | 937 | 2.40E-06 | 291 | 29 |
| TF | Factor: SP1; motif: GGGGYGGGGNS | TF:M01303 | 7502 | 2.40E-06 | 291 | 107 |
| TF | Factor: MTF-1; motif: NTTTTGCACACGGCNNN | TF:M12280 | 6032 | 2.50E-06 | 291 | 92 |
| TF | Factor: rfx3:SRF; motif: TRGCAACNNNNNCCNWATANGGN | TF:M08623 | 12775 | 2.50E-06 | 291 | 156 |
| TF | Factor: AP-2gamma:Elk-1; motif: NGCCKNRGGSGRCGGAAGTG; match class: 1 | TF:M08441_1 | 11870 | 2.50E-06 | 291 | 148 |
| TF | Factor: VDR; motif: GGGKNARNRRGGWSA; match class: 1 | TF:M00444_1 | 4198 | 2.60E-06 | 291 | 72 |
| TF | Factor: Sp3; motif: ASMCTTGGGSRGGG | TF:M00665 | 7012 | 2.60E-06 | 291 | 102 |
| TF | Factor: AP-4; motif: WGARYCAGCTGYGGNCNK | TF:M00005 | 4645 | 2.60E-06 | 291 | 77 |
| REAC | ECM proteoglycans | REAC:R-HSA-3000178 | 70 | 2.70E-06 | 291 | 9 |
| TF | Factor: c-Rel; motif: SGGRNTTTCC | TF:M00053 | 3259 | 2.70E-06 | 291 | 61 |
| TF | Factor: BTEB3; motif: CCNNSCCNSCCCCKCCCCC | TF:M09826 | 10774 | 2.80E-06 | 291 | 138 |
| TF | Factor: TIEG1; motif: NCCCNSNCCCCGCCCCC | TF:M12351 | 11551 | 2.80E-06 | 291 | 145 |
| TF | Factor: MEL1; motif: GARGAT | TF:M08794 | 9275 | 2.90E-06 | 291 | 124 |
| TF | Factor: NF-1A; motif: NGCCARN | TF:M03554 | 4210 | 2.90E-06 | 291 | 72 |
| GO:BP | response to inorganic substance | GO:0010035 | 502 | 3.00E-06 | 291 | 21 |
| KEGG | Amoebiasis | KEGG:05146 | 85 | 3.00E-06 | 291 | 9 |
| GO:BP | intracellular signal transduction | GO:0035556 | 2497 | 3.10E-06 | 291 | 51 |
| GO:BP | blood circulation | GO:0008015 | 455 | 3.20E-06 | 291 | 20 |
| GO:BP | negative regulation of cell population proliferation | GO:0008285 | 660 | 3.20E-06 | 291 | 24 |
| TF | Factor: CKROX; motif: SCCCTCCCC | TF:M01175 | 6740 | 3.20E-06 | 291 | 99 |
| TF | Factor: AR; motif: GGAACGGWACATGTTCT | TF:M08190 | 6740 | 3.20E-06 | 291 | 99 |
| TF | Factor: TEF-1; motif: ACATTCCWSNN | TF:M07340 | 3273 | 3.20E-06 | 291 | 61 |
| TF | Factor: IRF-4; motif: NAANGRGGAASTGAAASN | TF:M09959 | 2865 | 3.30E-06 | 291 | 56 |
| TF | Factor: KLF15; motif: NCCMCGCCCMCN | TF:M12158 | 9935 | 3.30E-06 | 291 | 130 |
| TF | Factor: Elk-1:SREBP-2; motif: RTCACGTGACCGGAAGN | TF:M08229 | 8564 | 3.30E-06 | 291 | 117 |
| GO:BP | negative regulation of macromolecule metabolic process | GO:0010605 | 2662 | 3.40E-06 | 291 | 53 |
| GO:BP | odontogenesis | GO:0042476 | 109 | 3.40E-06 | 291 | 11 |
| TF | Factor: six-4; motif: ANNNATGACACCNNNNN | TF:M01374 | 5406 | 3.40E-06 | 291 | 85 |
| TF | Factor: MAFA; motif: TCAGCAN | TF:M01709 | 5314 | 3.40E-06 | 291 | 84 |
| GO:BP | positive regulation of nitrogen compound metabolic process | GO:0051173 | 2747 | 3.60E-06 | 291 | 54 |
| TF | Factor: ZNFPT1; motif: YCNNCNCWGCCNY; match class: 1 | TF:M12721_1 | 2874 | 3.70E-06 | 291 | 56 |
| TF | Factor: NFATc1; motif: TTTCCWN | TF:M01718 | 5045 | 3.70E-06 | 291 | 81 |
| TF | Factor: IRX-1; motif: NACRYNNNNNNNNRYGNN; match class: 1 | TF:M11018_1 | 14591 | 3.90E-06 | 291 | 171 |
| TF | Factor: TEF; motif: YACATTCCWSNG | TF:M01305 | 3207 | 3.90E-06 | 291 | 60 |
| GO:BP | urogenital system development | GO:0001655 | 325 | 4.10E-06 | 291 | 17 |
| KEGG | Viral protein interaction with cytokine and cytokine receptor | KEGG:04061 | 63 | 4.10E-06 | 291 | 8 |
| TF | Factor: NF-1C; motif: NYYTGGCWNNNNKCCMN | TF:M09640 | 2406 | 4.10E-06 | 291 | 50 |
| TF | Factor: KLF; motif: GGGNGGGG; match class: 1 | TF:M07461_1 | 3129 | 4.10E-06 | 291 | 59 |
| REAC | Degradation of the extracellular matrix | REAC:R-HSA-1474228 | 128 | 4.20E-06 | 291 | 11 |
| GO:CC | complex of collagen trimers | GO:0098644 | 21 | 4.30E-06 | 291 | 6 |
| GO:CC | sarcomere | GO:0030017 | 201 | 4.30E-06 | 291 | 13 |
| TF | Factor: AP-4:Dlx-3; motif: NCAGCTGNNNNNNGTAATKR | TF:M08671 | 3638 | 4.40E-06 | 291 | 65 |
| TF | Factor: ROX; motif: GNNNCASGTGGS | TF:M09764 | 5157 | 4.40E-06 | 291 | 82 |
| GO:MF | immune receptor activity | GO:0140375 | 122 | 4.50E-06 | 291 | 11 |
| TF | Factor: YB-1; motif: NNNNCCAATNN | TF:M03862 | 7885 | 4.50E-06 | 291 | 110 |
| TF | Factor: ZNF37A; motif: CCYYGGCTCCNTSCCMN; match class: 1 | TF:M12354_1 | 5347 | 4.60E-06 | 291 | 84 |
| TF | Factor: ERF:SREBP-2; motif: NSCGGAARTCACGTGAT | TF:M08453 | 8820 | 4.70E-06 | 291 | 119 |
| TF | Factor: GR; motif: CNNNNTGTYCTNN | TF:M01836 | 4890 | 4.80E-06 | 291 | 79 |
| GO:BP | vascular process in circulatory system | GO:0003018 | 247 | 4.90E-06 | 291 | 15 |
| TF | Factor: SMAD4; motif: GKSRKKCAGMCANCY; match class: 1 | TF:M00733_1 | 2421 | 5.00E-06 | 291 | 50 |
| TF | Factor: Thap1; motif: SCGCCATSTTKGNTNMGGGCARNN | TF:M04723 | 10537 | 5.10E-06 | 291 | 135 |
| TF | Factor: Sox-9; motif: RNACAAAGGVN | TF:M01284 | 5079 | 5.10E-06 | 291 | 81 |
| TF | Factor: SMAD3; motif: CAGACAS; match class: 1 | TF:M07429_1 | 1137 | 5.30E-06 | 291 | 32 |
| TF | Factor: MaFA; motif: TCTGCTGASCNN | TF:M07325 | 4356 | 5.30E-06 | 291 | 73 |
| GO:BP | organic substance metabolic process | GO:0071704 | 10615 | 5.40E-06 | 291 | 135 |
| TF | Factor: P50; motif: GGRRANTCCCNN | TF:M03557 | 4359 | 5.40E-06 | 291 | 73 |
| TF | Factor: SREBP-2; motif: NNGYCACNNSMN | TF:M01177 | 7910 | 5.40E-06 | 291 | 110 |
| TF | Factor: Oct-2; motif: ATTTGCA | TF:M04715 | 5273 | 5.50E-06 | 291 | 83 |
| TF | Factor: TWIST1; motif: NNNCCAGATGTNN | TF:M12577 | 2350 | 5.50E-06 | 291 | 49 |
| TF | Factor: TEF-1; motif: CRCATWCCN | TF:M12041 | 4007 | 5.50E-06 | 291 | 69 |
| TF | Factor: MTF-1; motif: GTGTGCANMACTTTGCGCAC | TF:M01242 | 3492 | 5.70E-06 | 291 | 63 |
| TF | Factor: ATF-1; motif: CYYTGACGTCA | TF:M00691 | 4277 | 5.80E-06 | 291 | 72 |
| TF | Factor: Pax-6; motif: CTGACCTGGAACTM | TF:M00979 | 8330 | 5.90E-06 | 291 | 114 |
| TF | Factor: NF-1; motif: TTGGCNN | TF:M07364 | 4189 | 5.90E-06 | 291 | 71 |
| TF | Factor: Sox-4; motif: AACAAA; match class: 1 | TF:M03849_1 | 7618 | 6.00E-06 | 291 | 107 |
| GO:BP | cellular response to tumor necrosis factor | GO:0071356 | 213 | 6.10E-06 | 291 | 14 |
| REAC | Hemostasis | REAC:R-HSA-109582 | 570 | 6.10E-06 | 291 | 21 |
| TF | Factor: Erg; motif: NACCGGATATCCGGTN | TF:M11391 | 9170 | 6.10E-06 | 291 | 122 |
| MIRNA | hsa-miR-146a-5p | MIRNA:hsa-miR-146a-5p | 194 | 6.10E-06 | 291 | 13 |
| GO:CC | secretory granule lumen | GO:0034774 | 289 | 6.50E-06 | 291 | 15 |
| TF | Factor: LKLF; motif: CNCCACCCS | TF:M08819 | 5105 | 6.50E-06 | 291 | 81 |
| TF | Factor: E2F-2; motif: GCGCGCGCNCS; match class: 1 | TF:M11529_1 | 13618 | 6.50E-06 | 291 | 162 |
| GO:CC | I band | GO:0031674 | 138 | 6.60E-06 | 291 | 11 |
| TF | Factor: Fli-1; motif: NACCGGAWWTCCGGTY; match class: 1 | TF:M11399_1 | 10467 | 6.60E-06 | 291 | 134 |
| TF | Factor: ZXDL; motif: GSGSCNNGGGMRGCNCCGGGS | TF:M12722 | 9932 | 6.80E-06 | 291 | 129 |
| GO:CC | endoplasmic reticulum lumen | GO:0005788 | 291 | 7.10E-06 | 291 | 15 |
| GO:CC | cytoplasmic vesicle lumen | GO:0060205 | 291 | 7.10E-06 | 291 | 15 |
| TF | Factor: T-bet; motif: NNGNRGGTGTGRN | TF:M10089 | 5395 | 7.10E-06 | 291 | 84 |
| TF | Factor: E2F-4; motif: NTTTCSCGCC | TF:M07380 | 12143 | 7.10E-06 | 291 | 149 |
| TF | Factor: MAZ; motif: GGGMGGGGS | TF:M10432 | 8669 | 7.20E-06 | 291 | 117 |
| TF | Factor: Pax-3; motif: TCGTCACRCTTHM | TF:M00360 | 10921 | 7.30E-06 | 291 | 138 |
| TF | Factor: SRY; motif: AAACWAM | TF:M00148 | 7951 | 7.40E-06 | 291 | 110 |
| TF | Factor: Miz-1; motif: NNRGGWGGGGGAGGGGMRR | TF:M10112 | 8054 | 7.40E-06 | 291 | 111 |
| GO:BP | embryonic morphogenesis | GO:0048598 | 530 | 7.70E-06 | 291 | 21 |
| GO:CC | vesicle lumen | GO:0031983 | 293 | 7.70E-06 | 291 | 15 |
| GO:BP | positive regulation of angiogenesis | GO:0045766 | 148 | 7.80E-06 | 291 | 12 |
| GO:BP | positive regulation of vasculature development | GO:1904018 | 148 | 7.80E-06 | 291 | 12 |
| GO:BP | artery morphogenesis | GO:0048844 | 67 | 8.10E-06 | 291 | 9 |
| TF | Factor: PR; motif: RGNACATTYTGTNCTN | TF:M10042 | 2695 | 8.20E-06 | 291 | 53 |
| TF | Factor: NFATc2; motif: NTTTCCRTNNAYGGAAAN | TF:M11984 | 9531 | 8.20E-06 | 291 | 125 |
| REAC | Smooth Muscle Contraction | REAC:R-HSA-445355 | 37 | 8.30E-06 | 291 | 7 |
| TF | Factor: FOXP1; motif: TNTGTTTMY | TF:M09933 | 4223 | 8.30E-06 | 291 | 71 |
| TF | Factor: TEF-3:E2A; motif: NCAGSTGNGWATGYN | TF:M08438 | 4948 | 8.30E-06 | 291 | 79 |
| TF | Factor: AP-2; motif: GSCCSCRGGCNRNRNN | TF:M00800 | 8588 | 8.50E-06 | 291 | 116 |
| TF | Factor: PARP; motif: TTTCYN; match class: 1 | TF:M02027_1 | 10180 | 8.50E-06 | 291 | 131 |
| GO:BP | embryonic organ development | GO:0048568 | 387 | 8.70E-06 | 291 | 18 |
| GO:MF | growth factor binding | GO:0019838 | 130 | 8.80E-06 | 291 | 11 |
| TF | Factor: Hey1; motif: NGCRCGYGYN | TF:M11058 | 12064 | 8.80E-06 | 291 | 148 |
| TF | Factor: E2F-3:FOXI1; motif: NGACACCGCGCCCAC | TF:M08208 | 11509 | 9.10E-06 | 291 | 143 |
| TF | Factor: C-JUN:FRA-2; motif: NRTGACGTMAT | TF:M08941 | 5804 | 9.30E-06 | 291 | 88 |
| TF | Factor: Pasx-5; motif: GTCACGSTT | TF:M04765 | 9656 | 9.30E-06 | 291 | 126 |
| GO:BP | regulation of apoptotic signaling pathway | GO:2001233 | 344 | 9.60E-06 | 291 | 17 |
| TF | Factor: pax-6; motif: NYACGCNTSRNYGCNYN | TF:M11883 | 12304 | 9.60E-06 | 291 | 150 |
| GO:BP | artery development | GO:0060840 | 93 | 1.00E-05 | 291 | 10 |
| GO:BP | regulation of response to stress | GO:0080134 | 1192 | 1.00E-05 | 291 | 32 |
| GO:CC | supramolecular polymer | GO:0099081 | 834 | 1.00E-05 | 291 | 25 |
| GO:BP | regulation of anatomical structure morphogenesis | GO:0022603 | 878 | 1.10E-05 | 291 | 27 |
| GO:BP | myeloid leukocyte migration | GO:0097529 | 186 | 1.10E-05 | 291 | 13 |
| REAC | Non-integrin membrane-ECM interactions | REAC:R-HSA-3000171 | 58 | 1.10E-05 | 291 | 8 |
| GO:MF | platelet-derived growth factor binding | GO:0048407 | 11 | 1.20E-05 | 291 | 5 |
| KEGG | AGE-RAGE signaling pathway in diabetic complications | KEGG:04933 | 100 | 1.20E-05 | 291 | 9 |
| TF | Factor: SMAD3; motif: TGTCTGTCT | TF:M00701 | 3555 | 1.20E-05 | 291 | 63 |
| TF | Factor: pax-6; motif: NYACGCNTSANYGCNYN | TF:M11881 | 11996 | 1.20E-05 | 291 | 147 |
| TF | Factor: NFATc2; motif: NTTTCCRTNNAYGGAAAN; match class: 1 | TF:M11984_1 | 9477 | 1.20E-05 | 291 | 124 |
| TF | Factor: NF1C; motif: WGCCARR; match class: 1 | TF:M09763_1 | 3644 | 1.20E-05 | 291 | 64 |
| TF | Factor: C/EBPdelta; motif: TTGCNNMAN | TF:M03571 | 3137 | 1.20E-05 | 291 | 58 |
| MIRNA | hsa-miR-124-3p | MIRNA:hsa-miR-124-3p | 1393 | 1.20E-05 | 291 | 34 |
| GO:CC | myofibril | GO:0030016 | 220 | 1.30E-05 | 291 | 13 |
| TF | Factor: AP4; motif: NMNCAGCTGGN | TF:M12580 | 5085 | 1.30E-05 | 291 | 80 |
| TF | Factor: DRRS; motif: GNNGGGWGGG | TF:M10056 | 4356 | 1.30E-05 | 291 | 72 |
| TF | Factor: C/EBPalpha; motif: NRTTGTGCAAYNN | TF:M09596 | 4356 | 1.30E-05 | 291 | 72 |
| TF | Factor: MafB; motif: GNTGAC; match class: 1 | TF:M01227_1 | 4179 | 1.30E-05 | 291 | 70 |
| TF | Factor: Sp1; motif: GGGGCGGGGT | TF:M00008 | 9493 | 1.30E-05 | 291 | 124 |
| TF | Factor: rax; motif: NNCRTTAN | TF:M10948 | 10354 | 1.30E-05 | 291 | 132 |
| GO:BP | cellular homeostasis | GO:0019725 | 828 | 1.40E-05 | 291 | 26 |
| TF | Factor: SREBP-1; motif: RTCRCGTGAY | TF:M11081 | 9930 | 1.40E-05 | 291 | 128 |
| TF | Factor: STAT3; motif: NNTTCCRGGAANNNNN | TF:M01595 | 6729 | 1.40E-05 | 291 | 97 |
| TF | Factor: Smad3; motif: NGNCAGACASNNN | TF:M01888 | 3489 | 1.40E-05 | 291 | 62 |
| TF | Factor: GR; motif: GGTACAANNTGTYCTK | TF:M00205 | 5664 | 1.40E-05 | 291 | 86 |
| GO:BP | response to hormone | GO:0009725 | 774 | 1.50E-05 | 291 | 25 |
| TF | Factor: IRF-7; motif: AAGWGAA; match class: 1 | TF:M01884_1 | 3752 | 1.50E-05 | 291 | 65 |
| TF | Factor: SATB2; motif: GSCGCTGTCCNNGGTGCTGN | TF:M12660 | 5015 | 1.50E-05 | 291 | 79 |
| TF | Factor: BCL-11A; motif: NAAAGAGGAAGTGARAN | TF:M09595 | 3159 | 1.60E-05 | 291 | 58 |
| TF | Factor: Egr-1; motif: NGCGTGCGY | TF:M04950 | 10165 | 1.60E-05 | 291 | 130 |
| TF | Factor: E2F-3:HES-7; motif: NNNSGCGCSNNNNNCRCGYGNN; match class: 1 | TF:M08526_1 | 14003 | 1.60E-05 | 291 | 164 |
| TF | Factor: Lhx2; motif: WATTAN | TF:M07047 | 9102 | 1.60E-05 | 291 | 120 |
| GO:BP | cellular ion homeostasis | GO:0006873 | 609 | 1.70E-05 | 291 | 22 |
| TF | Factor: Fli-1; motif: NNRGGMAGGAAGGRRRGR; match class: 1 | TF:M09920_1 | 2997 | 1.70E-05 | 291 | 56 |
| TF | Factor: c-Myc; motif: CACGTGGC | TF:M03867 | 6456 | 1.70E-05 | 291 | 94 |
| TF | Factor: RELB; motif: NNATTCCCCNN | TF:M09784 | 4657 | 1.70E-05 | 291 | 75 |
| TF | Factor: p300; motif: CTGNCTYMAN | TF:M07266 | 6955 | 1.70E-05 | 291 | 99 |
| GO:BP | regulation of response to external stimulus | GO:0032101 | 839 | 1.80E-05 | 291 | 26 |
| GO:BP | regulation of cellular protein metabolic process | GO:0032268 | 2160 | 1.80E-05 | 291 | 45 |
| TF | Factor: p300; motif: ACNTCCG; match class: 1 | TF:M04826_1 | 14020 | 1.80E-05 | 291 | 164 |
| TF | Factor: C/EBP; motif: NNATTGCNNAANNN | TF:M00190 | 4665 | 1.80E-05 | 291 | 75 |
| TF | Factor: NF-1C; motif: NTTGGCNNNNTGCCARN | TF:M11729 | 1469 | 1.80E-05 | 291 | 36 |
| TF | Factor: ZNF460; motif: NNACNCCCCCCNN | TF:M12313 | 5505 | 1.90E-05 | 291 | 84 |
| TF | Factor: E2F-3; motif: NNGGCGGGAAA | TF:M09893 | 5600 | 1.90E-05 | 291 | 85 |
| TF | Factor: ZNF614; motif: NCYCWGCCYYNNN; match class: 1 | TF:M09862_1 | 3950 | 1.90E-05 | 291 | 67 |
| TF | Factor: AR; motif: GGNACRNNRTGTWCT | TF:M01201 | 5602 | 1.90E-05 | 291 | 85 |
| TF | Factor: CHD2; motif: TCTCGCGAG | TF:M04710 | 12870 | 1.90E-05 | 291 | 154 |
| TF | Factor: E2F-3; motif: GGCGGGN | TF:M02089 | 12418 | 2.00E-05 | 291 | 150 |
| TF | Factor: ZF5; motif: GSGCGCGR; match class: 1 | TF:M00716_1 | 13336 | 2.00E-05 | 291 | 158 |
| TF | Factor: SUHW1; motif: TCTCTCCAGTRTGAATTCTCTGAT | TF:M10541 | 6086 | 2.00E-05 | 291 | 90 |
| GO:BP | response to tumor necrosis factor | GO:0034612 | 235 | 2.10E-05 | 291 | 14 |
| TF | Factor: LTF; motif: GKVACTTNC | TF:M01692 | 3701 | 2.20E-05 | 291 | 64 |
| TF | Factor: egr-3; motif: GTGGGY; match class: 1 | TF:M03818_1 | 5816 | 2.30E-05 | 291 | 87 |
| TF | Factor: TFAP2A; motif: YGCCCNNRGGCN | TF:M04146 | 6009 | 2.30E-05 | 291 | 89 |
| TF | Factor: Smad2; motif: TGTCTGNCWCCT | TF:M10059 | 5342 | 2.30E-05 | 291 | 82 |
| TF | Factor: E2A; motif: CACCTGNY | TF:M00973 | 4692 | 2.30E-05 | 291 | 75 |
| TF | Factor: RAR-gamma; motif: RGGTCANNNTGNCCNNNN | TF:M10045 | 6400 | 2.30E-05 | 291 | 93 |
| TF | Factor: Kaiso; motif: GCMGGGRGCRGS; match class: 1 | TF:M03876_1 | 8628 | 2.30E-05 | 291 | 115 |
| TF | Factor: PBX2; motif: TGANTGACAGN | TF:M09736 | 4240 | 2.40E-05 | 291 | 70 |
| GO:BP | response to transforming growth factor beta | GO:0071559 | 238 | 2.50E-05 | 291 | 14 |
| TF | Factor: ZNF462; motif: YYYYCTSCWG; match class: 1 | TF:M12707_1 | 3033 | 2.50E-05 | 291 | 56 |
| TF | Factor: RUNX2; motif: WRACCGCANWAACCGCAN | TF:M04107 | 11232 | 2.60E-05 | 291 | 139 |
| TF | Factor: ZXDL; motif: NGGGGWS; match class: 1 | TF:M05775_1 | 1561 | 2.60E-05 | 291 | 37 |
| GO:CC | anchoring junction | GO:0070161 | 815 | 2.70E-05 | 291 | 24 |
| TF | Factor: RBPJK; motif: NSNSTGGGAAN | TF:M10085 | 3038 | 2.70E-05 | 291 | 56 |
| TF | Factor: ZNF592; motif: NSARNATGGAGKN; match class: 1 | TF:M09766_1 | 1421 | 2.70E-05 | 291 | 35 |
| TF | Factor: Sp1; motif: GGNDGGRGGCGGGG | TF:M04953 | 8128 | 2.70E-05 | 291 | 110 |
| TF | Factor: E2F-1; motif: NTTTTGGCGCCAWWWN | TF:M11533 | 12131 | 2.70E-05 | 291 | 147 |
| TF | Factor: ITF-2; motif: NGCAGSTGKS | TF:M09962 | 4254 | 2.70E-05 | 291 | 70 |
| TF | Factor: HEB; motif: RCCWGCTG | TF:M00698 | 2470 | 2.70E-05 | 291 | 49 |
| TF | Factor: SRY; motif: AACAATANCATTGTT | TF:M04555 | 5745 | 2.80E-05 | 291 | 86 |
| GO:BP | cellular chemical homeostasis | GO:0055082 | 741 | 2.90E-05 | 291 | 24 |
| GO:BP | epithelial cell proliferation | GO:0050673 | 371 | 2.90E-05 | 291 | 17 |
| TF | Factor: ZNF148; motif: NNCCCCTCCCCC | TF:M12464 | 6431 | 3.00E-05 | 291 | 93 |
| GO:BP | intrinsic apoptotic signaling pathway | GO:0097193 | 283 | 3.10E-05 | 291 | 15 |
| TF | Factor: SATB1; motif: NTTTAT; match class: 1 | TF:M03564_1 | 10054 | 3.10E-05 | 291 | 128 |
| TF | Factor: Gscl; motif: NTAATCCN | TF:M10924 | 5762 | 3.20E-05 | 291 | 86 |
| TF | Factor: BRCA1:USF2; motif: KTNNGTTG | TF:M01082 | 6054 | 3.30E-05 | 291 | 89 |
| TF | Factor: TEF-3:C/EBPbeta; motif: RTTGCGYAAYNNNNNGGAATGY | TF:M08626 | 5958 | 3.30E-05 | 291 | 88 |
| TF | Factor: GATA-1; motif: SNNGATNNNN | TF:M00075 | 6252 | 3.40E-05 | 291 | 91 |
| TF | Factor: ZIC4; motif: NNCCNCCCRYNGYGN | TF:M12227 | 10284 | 3.40E-05 | 291 | 130 |
| TF | Factor: ipf1; motif: TGATTGATK | TF:M10031 | 7448 | 3.50E-05 | 291 | 103 |
| TF | Factor: HES-7; motif: GNCACGYGNN | TF:M11066 | 10511 | 3.60E-05 | 291 | 132 |
| TF | Factor: C/EBPbeta; motif: RTTGCGCAA | TF:M04690 | 3752 | 3.70E-05 | 291 | 64 |
| TF | Factor: Erg; motif: MCAGGAAA; match class: 1 | TF:M07284_1 | 3752 | 3.70E-05 | 291 | 64 |
| TF | Factor: Egr-2; motif: CCCMCNCN | TF:M02092 | 4289 | 3.80E-05 | 291 | 70 |
| TF | Factor: Erm:C/EBPdelta; motif: RSCGGAWRTTGCGYAAY | TF:M08458 | 8491 | 3.80E-05 | 291 | 113 |
| TF | Factor: BTEB4; motif: NCCACGCCCM | TF:M12186 | 8491 | 3.80E-05 | 291 | 113 |
| GO:CC | secretory granule membrane | GO:0030667 | 287 | 4.10E-05 | 291 | 14 |
| KEGG | PI3K-Akt signaling pathway | KEGG:04151 | 319 | 4.10E-05 | 291 | 14 |
| TF | Factor: PLAG1; motif: CCCCCKWNNNGGSCCC | TF:M01973 | 5790 | 4.10E-05 | 291 | 86 |
| TF | Factor: HOXA3; motif: NNNNRNTAATTARY; match class: 1 | TF:M01337_1 | 10095 | 4.10E-05 | 291 | 128 |
| TF | Factor: AP-2beta; motif: NSCCNNNGGSN | TF:M11478 | 9237 | 4.10E-05 | 291 | 120 |
| GO:BP | cellular metal ion homeostasis | GO:0006875 | 531 | 4.20E-05 | 291 | 20 |
| TF | Factor: C/EBPalpha; motif: NNNTTNNGCAANN | TF:M01866 | 4299 | 4.20E-05 | 291 | 70 |
| TF | Factor: ZEB1; motif: CAGGTGN | TF:M04831 | 6971 | 4.20E-05 | 291 | 98 |
| TF | Factor: TFIIB; motif: YTNTMTGMSN | TF:M08904 | 5698 | 4.20E-05 | 291 | 85 |
| TF | Factor: AP-4; motif: YCAGCTGNKN | TF:M10097 | 5226 | 4.30E-05 | 291 | 80 |
| TF | Factor: MZF-1; motif: TGGGGAR; match class: 1 | TF:M01733_1 | 4394 | 4.30E-05 | 291 | 71 |
| TF | Factor: Sp1; motif: NGGGGCGGGGN | TF:M07395 | 10215 | 4.40E-05 | 291 | 129 |
| TF | Factor: TEAD1; motif: NNACATTCCAGNN | TF:M12500 | 5045 | 4.60E-05 | 291 | 78 |
| GO:BP | regulation of cellular biosynthetic process | GO:0031326 | 3648 | 4.70E-05 | 291 | 62 |
| TF | Factor: XBP-1; motif: WNNGMCACGTC | TF:M01770 | 9900 | 4.70E-05 | 291 | 126 |
| TF | Factor: AP-2; motif: NNGCCTK | TF:M01859 | 6687 | 4.70E-05 | 291 | 95 |
| TF | Factor: NFATc1; motif: TTTTCCATGGAAAN | TF:M04053 | 7187 | 4.70E-05 | 291 | 100 |
| TF | Factor: RARG; motif: RAGGTCAASYARAGGTCA | TF:M04485 | 4222 | 4.70E-05 | 291 | 69 |
| GO:BP | negative regulation of apoptotic signaling pathway | GO:2001234 | 211 | 4.80E-05 | 291 | 13 |
| GO:BP | regulation of cytokine production | GO:0001817 | 646 | 4.90E-05 | 291 | 22 |
| TF | Factor: Nfe2l1; motif: TGYNNAGTCATT | TF:M07390 | 5626 | 5.10E-05 | 291 | 84 |
| GO:BP | negative regulation of cellular protein metabolic process | GO:0032269 | 885 | 5.20E-05 | 291 | 26 |
| TF | Factor: Tbx3; motif: NRAGGTGNNAR | TF:M10090 | 4322 | 5.20E-05 | 291 | 70 |
| GO:BP | chemical homeostasis | GO:0048878 | 1078 | 5.30E-05 | 291 | 29 |
| GO:BP | positive regulation of cell death | GO:0010942 | 539 | 5.40E-05 | 291 | 20 |
| TF | Factor: Fli-1:E2A; motif: NCCGGAWRCASCTGN | TF:M08472 | 7411 | 5.50E-05 | 291 | 102 |
| TF | Factor: ZNF383; motif: SSNGGGMGGNGSNGGS | TF:M12703 | 8544 | 5.50E-05 | 291 | 113 |
| TF | Factor: E2F-4; motif: SNGGGCGGGAANN; match class: 1 | TF:M09894_1 | 13166 | 5.50E-05 | 291 | 155 |
| TF | Factor: RXRALPHA; motif: CNNTGRCCTYTNN | TF:M09833 | 4604 | 5.60E-05 | 291 | 73 |
| TF | Factor: MLR1; motif: NTGNMCYYTGNNCYN | TF:M12637 | 3795 | 5.70E-05 | 291 | 64 |
| TF | Factor: ZF5; motif: GGSGCGCGS; match class: 1 | TF:M10438_1 | 13986 | 5.70E-05 | 291 | 162 |
| GO:BP | lymphocyte activation | GO:0046649 | 652 | 5.80E-05 | 291 | 22 |
| GO:BP | cytokine production | GO:0001816 | 652 | 5.80E-05 | 291 | 22 |
| GO:BP | cellular cation homeostasis | GO:0030003 | 596 | 5.80E-05 | 291 | 21 |
| GO:BP | chordate embryonic development | GO:0043009 | 596 | 5.80E-05 | 291 | 21 |
| TF | Factor: Erm; motif: NRRSAGGAARNGRN; match class: 1 | TF:M09916_1 | 1466 | 5.80E-05 | 291 | 35 |
| GO:BP | cell chemotaxis | GO:0060326 | 255 | 6.00E-05 | 291 | 14 |
| GO:BP | morphogenesis of an epithelium | GO:0002009 | 490 | 6.00E-05 | 291 | 19 |
| TF | Factor: CREB1; motif: NNNNSSGGCGCSSNNNNRTGACGTCAC | TF:M12591 | 12377 | 6.00E-05 | 291 | 148 |
| TF | Factor: Pax-6; motif: TTTCACGCWTGANTGMNYN | TF:M04065 | 9085 | 6.10E-05 | 291 | 118 |
| TF | Factor: BTEB2; motif: RGGGNGKGGN | TF:M07277 | 8145 | 6.20E-05 | 291 | 109 |
| TF | Factor: Nrf-2; motif: NTGCTGAGTCAKN | TF:M00821 | 3984 | 6.30E-05 | 291 | 66 |
| TF | Factor: KLF15; motif: RCCMCRCCCMCN; match class: 1 | TF:M12160_1 | 7532 | 6.30E-05 | 291 | 103 |
| GO:MF | cytokine binding | GO:0019955 | 124 | 6.50E-05 | 291 | 10 |
| TF | Factor: IRF-8; motif: NYGAAACYGAAACTN | TF:M11684 | 3986 | 6.50E-05 | 291 | 66 |
| TF | Factor: E2F-2; motif: NWTTTGGCGCCAWWNN | TF:M11530 | 13542 | 6.50E-05 | 291 | 158 |
| TF | Factor: IRF-8; motif: AGTTTCW | TF:M01665 | 3988 | 6.60E-05 | 291 | 66 |
| TF | Factor: LRF; motif: NRGGGKCKY | TF:M10115 | 6932 | 6.60E-05 | 291 | 97 |
| TF | Factor: SPI1; motif: AGGAAGT | TF:M02278 | 5850 | 6.60E-05 | 291 | 86 |
| TF | Factor: Fli-1; motif: MGGAAGT | TF:M07382 | 5850 | 6.60E-05 | 291 | 86 |
| TF | Factor: PEA3; motif: AGGAAGT | TF:M03579 | 5850 | 6.60E-05 | 291 | 86 |
| TF | Factor: C-ets-1; motif: AGGAAGN | TF:M01870 | 5850 | 6.60E-05 | 291 | 86 |
| GO:BP | response to peptide | GO:1901652 | 442 | 6.70E-05 | 291 | 18 |
| TF | Factor: Six-2; motif: NCGTATCRNN | TF:M11031 | 7846 | 6.70E-05 | 291 | 106 |
| GO:MF | molecular transducer activity | GO:0060089 | 1139 | 6.80E-05 | 291 | 29 |
| GO:MF | signaling receptor activity | GO:0038023 | 1139 | 6.80E-05 | 291 | 29 |
| TF | Factor: SRY; motif: TCAATAMCATTGA | TF:M04557 | 8365 | 6.80E-05 | 291 | 111 |
| TF | Factor: SREBP-2; motif: NTCACCYNNNN | TF:M03852 | 7238 | 6.80E-05 | 291 | 100 |
| TF | Factor: Ikaros; motif: TGGGAGN; match class: 1 | TF:M07260_1 | 6245 | 6.90E-05 | 291 | 90 |
| GO:BP | muscle system process | GO:0003012 | 394 | 7.00E-05 | 291 | 17 |
| GO:BP | immune system development | GO:0002520 | 899 | 7.00E-05 | 291 | 26 |
| TF | Factor: AP-2beta; motif: GCNNNGGSCNGVGGGN | TF:M01858 | 7042 | 7.10E-05 | 291 | 98 |
| TF | Factor: MafA; motif: GGTCAGCAGH | TF:M03796 | 2710 | 7.20E-05 | 291 | 51 |
| GO:BP | positive regulation of cell migration | GO:0030335 | 496 | 7.30E-05 | 291 | 19 |
| TF | Factor: E2F-1; motif: NNNNGGCGGGAARN | TF:M09892 | 12641 | 7.40E-05 | 291 | 150 |
| TF | Factor: E2F-3; motif: NTTTTGGCGCCAAAAN | TF:M11527 | 6161 | 7.70E-05 | 291 | 89 |
| TF | Factor: ZNF333; motif: ATAAT; match class: 1 | TF:M01230_1 | 9656 | 7.80E-05 | 291 | 123 |
| TF | Factor: RXR:RAR; motif: RGKTCANNNRGAGGTCA | TF:M02272 | 5486 | 7.80E-05 | 291 | 82 |
| GO:BP | negative regulation of catalytic activity | GO:0043086 | 607 | 7.90E-05 | 291 | 21 |
| KEGG | Protein digestion and absorption | KEGG:04974 | 92 | 8.00E-05 | 291 | 8 |
| TF | Factor: Spz1; motif: DNNGGRGGGWWNNNN; match class: 1 | TF:M00446_1 | 2638 | 8.10E-05 | 291 | 50 |
| GO:BP | ion homeostasis | GO:0050801 | 723 | 8.20E-05 | 291 | 23 |
| GO:BP | transforming growth factor beta receptor signaling pathway | GO:0007179 | 183 | 8.40E-05 | 291 | 12 |
| TF | Factor: c-Jun; motif: NATGACGTCAYN | TF:M11266 | 7368 | 8.40E-05 | 291 | 101 |
| TF | Factor: CEBPD; motif: NNTTGCACAAYNN | TF:M12567 | 1780 | 8.40E-05 | 291 | 39 |
| GO:BP | regulation of biosynthetic process | GO:0009889 | 3706 | 8.50E-05 | 291 | 62 |
| GO:BP | hematopoietic or lymphoid organ development | GO:0048534 | 846 | 8.60E-05 | 291 | 25 |
| TF | Factor: BRN1; motif: HAATGCN; match class: 1 | TF:M03813_1 | 5787 | 8.70E-05 | 291 | 85 |
| TF | Factor: Erm; motif: RSAGGAAGGAAGTN | TF:M11423 | 4288 | 8.80E-05 | 291 | 69 |
| TF | Factor: HELIOS; motif: RNARRRGGAASTGARAN | TF:M09745 | 2894 | 9.00E-05 | 291 | 53 |
| TF | Factor: IRF-4; motif: NYGAAASYGAAACYN | TF:M11686 | 2895 | 9.10E-05 | 291 | 53 |
| GO:BP | negative regulation of signal transduction | GO:0009968 | 1173 | 9.20E-05 | 291 | 30 |
| TF | Factor: pax-6; motif: NYACGCWTSANYGMNCN | TF:M11880 | 11884 | 9.20E-05 | 291 | 143 |
| TF | Factor: p53; motif: RGRCWWGYCYNGRCWWGYYY | TF:M01652 | 7281 | 9.30E-05 | 291 | 100 |
| TF | Factor: ARP-1; motif: TGARCCYTTGAMCCCW | TF:M00155 | 4662 | 9.40E-05 | 291 | 73 |
| TF | Factor: MIBP1; motif: WNWCCCCAGCTR | TF:M03878 | 2488 | 9.40E-05 | 291 | 48 |
| TF | Factor: Oct-1; motif: MKVATTTGCATATT | TF:M00161 | 2408 | 9.50E-05 | 291 | 47 |
| TF | Factor: Sox-10; motif: NACAAWG; match class: 1 | TF:M02116_1 | 6582 | 9.50E-05 | 291 | 93 |
| GO:BP | embryo development ending in birth or egg hatching | GO:0009792 | 614 | 9.60E-05 | 291 | 21 |
| TF | Factor: CTF/NF1; motif: TTGGCN; match class: 1 | TF:M02050_1 | 3413 | 9.90E-05 | 291 | 59 |
| TF | Factor: FOXP3; motif: NNNVAAACANWD | TF:M07419 | 5230 | 9.90E-05 | 291 | 79 |
| TF | Factor: HMX2; motif: SCACTTANC | TF:M10646 | 6195 | 1.00E-04 | 291 | 89 |
| GO:MF | interleukin-1 receptor activity | GO:0004908 | 7 | 1.10E-04 | 291 | 4 |
| TF | Factor: E2F; motif: TTTSGCGS | TF:M00426 | 11014 | 1.10E-04 | 291 | 135 |
| TF | Factor: TCF-1; motif: ACATCGRGRCGCTGW | TF:M11601 | 13629 | 1.10E-04 | 291 | 158 |
| TF | Factor: E2F-1; motif: WWTGGCGCCAAA | TF:M04515 | 12480 | 1.10E-04 | 291 | 148 |
| GO:BP | response to oxidative stress | GO:0006979 | 409 | 1.20E-04 | 291 | 17 |
| GO:BP | mesangial cell development | GO:0072143 | 6 | 1.20E-04 | 291 | 4 |
| REAC | Scavenging by Class A Receptors | REAC:R-HSA-3000480 | 18 | 1.20E-04 | 291 | 5 |
| TF | Factor: Pax-8; motif: NCNNTNNTGCRTGANNNN | TF:M00328 | 8029 | 1.20E-04 | 291 | 107 |
| TF | Factor: FXR; motif: NRGGKCANTGRCCNNNNGG | TF:M10014 | 5159 | 1.20E-04 | 291 | 78 |
| TF | Factor: Oct-2; motif: NYATGCAAATN | TF:M10034 | 5065 | 1.20E-04 | 291 | 77 |
| TF | Factor: ZBTB49; motif: NGSCGCTGTCCNNGGTGCTGAN | TF:M12692 | 1957 | 1.20E-04 | 291 | 41 |
| REAC | Erythrocytes take up oxygen and release carbon dioxide | REAC:R-HSA-1247673 | 8 | 1.30E-04 | 291 | 4 |
| TF | Factor: E2F-1:Elk-1; motif: SGCGCSNNAMCGGAAGT | TF:M08205 | 13426 | 1.30E-04 | 291 | 156 |
| TF | Factor: Pax-5; motif: BCNNNRNGCANBGNTGNRTAGCSGCHNB; match class: 1 | TF:M00143_1 | 4608 | 1.30E-04 | 291 | 72 |
| TF | Factor: PPARGAMMA; motif: NWNTRGGTYANN | TF:M08961 | 4518 | 1.30E-04 | 291 | 71 |
| GO:BP | positive regulation of cell motility | GO:2000147 | 516 | 1.40E-04 | 291 | 19 |
| GO:BP | muscle contraction | GO:0006936 | 317 | 1.40E-04 | 291 | 15 |
| GO:BP | regulation of protein metabolic process | GO:0051246 | 2316 | 1.40E-04 | 291 | 45 |
| GO:CC | supramolecular fiber | GO:0099512 | 826 | 1.40E-04 | 291 | 23 |
| TF | Factor: NF-1B; motif: KCCAGANWN | TF:M08825 | 4990 | 1.40E-04 | 291 | 76 |
| TF | Factor: RelB:p50; motif: RGAAANTCCCYNNHGC | TF:M03882 | 3891 | 1.40E-04 | 291 | 64 |
| TF | Factor: E2F-2; motif: NTTTTGGCGCCAWWWN | TF:M11528 | 9539 | 1.40E-04 | 291 | 121 |
| GO:BP | cellular response to transforming growth factor beta stimulus | GO:0071560 | 232 | 1.50E-04 | 291 | 13 |
| GO:BP | leukocyte chemotaxis | GO:0030595 | 193 | 1.50E-04 | 291 | 12 |
| GO:BP | granulocyte migration | GO:0097530 | 123 | 1.50E-04 | 291 | 10 |
| GO:BP | epithelial cell differentiation involved in kidney development | GO:0035850 | 45 | 1.50E-04 | 291 | 7 |
| KEGG | Complement and coagulation cascades | KEGG:04610 | 70 | 1.50E-04 | 291 | 7 |
| TF | Factor: Sp2; motif: GGGGCGGGG | TF:M10435 | 7447 | 1.50E-04 | 291 | 101 |
| TF | Factor: TF3C-beta; motif: CCNGGAGGGCTTCCTGGAGGAG; match class: 1 | TF:M04863_1 | 7966 | 1.50E-04 | 291 | 106 |
| TF | Factor: Fli-1:HOXB13; motif: NNCGGAARYNRTWAA | TF:M08471 | 4441 | 1.50E-04 | 291 | 70 |
| TF | Factor: NF-1; motif: NTGGNNNNNNGCCAANN | TF:M00806 | 3720 | 1.50E-04 | 291 | 62 |
| TF | Factor: PU.1; motif: NRAAAGAGGAAGTGARA | TF:M09659 | 2208 | 1.50E-04 | 291 | 44 |
| TF | Factor: AP-2gamma; motif: NSCCYNNRGSN | TF:M11482 | 6252 | 1.50E-04 | 291 | 89 |
| TF | Factor: ehf; motif: NRANNAGGAARNRRNNN | TF:M09604 | 1976 | 1.60E-04 | 291 | 41 |
| TF | Factor: HOXB5; motif: GTCATTAN | TF:M10701 | 4817 | 1.60E-04 | 291 | 74 |
| TF | Factor: PEA3; motif: NTCGTAAATGCA | TF:M11431 | 9024 | 1.60E-04 | 291 | 116 |
| TF | Factor: AR; motif: NNNGNRRGNACANNGTGTTCTNNNNNN | TF:M00953 | 1978 | 1.60E-04 | 291 | 41 |
| TF | Factor: Olf-1; motif: NNCDABTCCCYAGRGARBNKGN | TF:M00261 | 3203 | 1.60E-04 | 291 | 56 |
| TF | Factor: MAZ; motif: GGGGAGGG; match class: 1 | TF:M00649_1 | 3905 | 1.60E-04 | 291 | 64 |
| TF | Factor: SREBP-1; motif: KATCACCCCAC | TF:M00221 | 6557 | 1.60E-04 | 291 | 92 |
| MIRNA | hsa-miR-29a-3p | MIRNA:hsa-miR-29a-3p | 256 | 1.60E-04 | 291 | 13 |
| TF | Factor: C/EBPgamma:Elf-1; motif: TKRCGHAATWSCGGAAGT | TF:M08676 | 7465 | 1.70E-04 | 291 | 101 |
| TF | Factor: NF-KAPPAB1; motif: NGGKRNTTYCCCN; match class: 1 | TF:M08952_1 | 3120 | 1.70E-04 | 291 | 55 |
| TF | Factor: TFII-I; motif: RGAGGKAGG; match class: 1 | TF:M00706_1 | 1461 | 1.70E-04 | 291 | 34 |
| TF | Factor: ESR1; motif: NNNNMAGGTCACCCTGACCY | TF:M02261 | 4090 | 1.70E-04 | 291 | 66 |
| TF | Factor: AP-2gamma; motif: GCCYNCRGSN; match class: 1 | TF:M03811_1 | 3646 | 1.80E-04 | 291 | 61 |
| TF | Factor: POU2F1; motif: ATTTGCATN | TF:M07058 | 2707 | 1.80E-04 | 291 | 50 |
| GO:MF | integrin binding | GO:0005178 | 139 | 1.90E-04 | 291 | 10 |
| GO:BP | regulation of intrinsic apoptotic signaling pathway | GO:2001242 | 160 | 1.90E-04 | 291 | 11 |
| GO:BP | negative regulation of protein metabolic process | GO:0051248 | 947 | 1.90E-04 | 291 | 26 |
| TF | Factor: CTF1; motif: TGGCASCNNGCCAA; match class: 1 | TF:M01196_1 | 1324 | 1.90E-04 | 291 | 32 |
| TF | Factor: ER-beta; motif: RGGTCASCNTGMCCY; match class: 1 | TF:M09910_1 | 3740 | 1.90E-04 | 291 | 62 |
| TF | Factor: C/EBPbeta; motif: RTTGCGCAA; match class: 1 | TF:M04690_1 | 3216 | 1.90E-04 | 291 | 56 |
| TF | Factor: Gli2; motif: GACCACCCANG | TF:M01703 | 3303 | 1.90E-04 | 291 | 57 |
| TF | Factor: Sp1; motif: NGGGGGCGGGGCCNGGGGGGGG | TF:M10071 | 8206 | 1.90E-04 | 291 | 108 |
| TF | Factor: znf136; motif: CAAGAATWCTATAYCCAG | TF:M10535 | 6878 | 1.90E-04 | 291 | 95 |
| GO:BP | epithelial cell apoptotic process | GO:1904019 | 96 | 2.00E-04 | 291 | 9 |
| GO:BP | positive regulation of cellular component movement | GO:0051272 | 529 | 2.00E-04 | 291 | 19 |
| GO:BP | positive regulation of locomotion | GO:0040017 | 530 | 2.00E-04 | 291 | 19 |
| REAC | Signaling by Receptor Tyrosine Kinases | REAC:R-HSA-9006934 | 472 | 2.00E-04 | 291 | 17 |
| TF | Factor: TEF-1; motif: GRRATG; match class: 1 | TF:M00704_1 | 4564 | 2.00E-04 | 291 | 71 |
| GO:BP | kidney development | GO:0001822 | 282 | 2.10E-04 | 291 | 14 |
| GO:BP | pattern specification process | GO:0007389 | 376 | 2.10E-04 | 291 | 16 |
| TF | Factor: Lhx8; motif: TGATTG | TF:M07476 | 7600 | 2.10E-04 | 291 | 102 |
| TF | Factor: Freac-7; motif: WNNANATAAAYANNNN | TF:M00293 | 3753 | 2.10E-04 | 291 | 62 |
| TF | Factor: C/EBPalpha; motif: NRTTGTGCAAYN | TF:M09880 | 2890 | 2.20E-04 | 291 | 52 |
| TF | Factor: Dlx-5; motif: AATTAN | TF:M03546 | 9177 | 2.20E-04 | 291 | 117 |
| TF | Factor: DRI1; motif: AATTAA | TF:M01654 | 9177 | 2.20E-04 | 291 | 117 |
| TF | Factor: AP-2; motif: SNNNCCNCAGGCN; match class: 1 | TF:M00915_1 | 4390 | 2.20E-04 | 291 | 69 |
| TF | Factor: AHR; motif: CACGCN | TF:M01855 | 7712 | 2.20E-04 | 291 | 103 |
| TF | Factor: nerf; motif: YRNCAGGAAGYRGSTBDS; match class: 1 | TF:M00531_1 | 1624 | 2.30E-04 | 291 | 36 |
| TF | Factor: CRX; motif: YTAATC; match class: 1 | TF:M01712_1 | 4120 | 2.30E-04 | 291 | 66 |
| TF | Factor: AR; motif: AGWACATNWTGTTCT | TF:M00447 | 3496 | 2.30E-04 | 291 | 59 |
| TF | Factor: C-FOS; motif: NNNKATGACGTCATNNN | TF:M08947 | 5921 | 2.50E-04 | 291 | 85 |
| TF | Factor: HOXA7; motif: GYMATTAN | TF:M10690 | 8983 | 2.50E-04 | 291 | 115 |
| TF | Factor: TCF-1; motif: ACATCGRGRCGCTGW; match class: 1 | TF:M11603_1 | 12396 | 2.50E-04 | 291 | 146 |
| TF | Factor: HOXD4; motif: NNYMATTANN | TF:M08774 | 3332 | 2.50E-04 | 291 | 57 |
| GO:BP | neutrophil migration | GO:1990266 | 99 | 2.60E-04 | 291 | 9 |
| TF | Factor: Sox-10; motif: CWTTGT; match class: 1 | TF:M03138_1 | 5442 | 2.60E-04 | 291 | 80 |
| TF | Factor: TCF-1; motif: CTTTGW; match class: 1 | TF:M03857_1 | 5442 | 2.60E-04 | 291 | 80 |
| TF | Factor: C/EBPgamma; motif: YTBATTTCARAAW | TF:M00622 | 5635 | 2.60E-04 | 291 | 82 |
| TF | Factor: Elf-1; motif: RNWMBAGGAART | TF:M00746 | 2905 | 2.60E-04 | 291 | 52 |
| TF | Factor: E2F-1; motif: TTTGGCGCCAAA | TF:M04516 | 10723 | 2.60E-04 | 291 | 131 |
| TF | Factor: LRF; motif: NGNAGNGGGTYN | TF:M04617 | 5639 | 2.60E-04 | 291 | 82 |
| GO:CC | non-membrane-bounded organelle | GO:0043228 | 4977 | 2.70E-04 | 291 | 72 |
| GO:CC | intracellular non-membrane-bounded organelle | GO:0043232 | 4977 | 2.70E-04 | 291 | 72 |
| TF | Factor: MAF; motif: GCTGAGTCAN | TF:M07296 | 4877 | 2.70E-04 | 291 | 74 |
| TF | Factor: BTEB1; motif: GGGGGCGGGGCNGSGGGNGS | TF:M09723 | 9530 | 2.70E-04 | 291 | 120 |
| TF | Factor: FOXM1; motif: NAGASTGATTA | TF:M04611 | 7843 | 2.70E-04 | 291 | 104 |
| TF | Factor: AR; motif: GNNCNNNNTGTTCTN | TF:M01996 | 2495 | 2.70E-04 | 291 | 47 |
| GO:BP | regulation of cell activation | GO:0050865 | 541 | 2.80E-04 | 291 | 19 |
| GO:BP | granulocyte chemotaxis | GO:0071621 | 100 | 2.80E-04 | 291 | 9 |
| TF | Factor: RBPJK; motif: SYGTGRGAANN | TF:M09665 | 3871 | 2.80E-04 | 291 | 63 |
| TF | Factor: B-ATF; motif: NNATGACACN | TF:M11300 | 7440 | 2.80E-04 | 291 | 100 |
| TF | Factor: Rarb; motif: AGGTCANNYARAGGTCA | TF:M08035 | 4979 | 2.80E-04 | 291 | 75 |
| GO:BP | mesangial cell differentiation | GO:0072007 | 7 | 2.90E-04 | 291 | 4 |
| KEGG | IL-17 signaling pathway | KEGG:04657 | 77 | 2.90E-04 | 291 | 7 |
| TF | Factor: NKX25; motif: NNANCCACTTRAAWTT | TF:M01414 | 8271 | 2.90E-04 | 291 | 108 |
| TF | Factor: RREB-1; motif: CCCCAAACMMCCCC; match class: 1 | TF:M00257_1 | 4057 | 2.90E-04 | 291 | 65 |
| TF | Factor: NF-kappaB; motif: GGGAMTTYCC | TF:M00054 | 2179 | 2.90E-04 | 291 | 43 |
| GO:BP | regulation of epithelial cell apoptotic process | GO:1904035 | 73 | 3.00E-04 | 291 | 8 |
| TF | Factor: LBX2; motif: CTNRANSTAATTA | TF:M04383 | 6738 | 3.00E-04 | 291 | 93 |
| TF | Factor: AR; motif: NGNACANNNTGTTCYNN; match class: 1 | TF:M09589_1 | 945 | 3.00E-04 | 291 | 26 |
| GO:BP | metal ion homeostasis | GO:0055065 | 601 | 3.10E-04 | 291 | 20 |
| GO:BP | defense response to other organism | GO:0098542 | 842 | 3.10E-04 | 291 | 24 |
| TF | Factor: E2F-3; motif: NTTTTGGCGCCAAAAN; match class: 1 | TF:M11527_1 | 5953 | 3.10E-04 | 291 | 85 |
| GO:BP | organonitrogen compound metabolic process | GO:1901564 | 5820 | 3.20E-04 | 291 | 83 |
| GO:BP | nephron development | GO:0072006 | 133 | 3.20E-04 | 291 | 10 |
| TF | Factor: Tbx3; motif: NAGGTGTSAN | TF:M12024 | 3800 | 3.30E-04 | 291 | 62 |
| TF | Factor: NKX2-2; motif: NNNCCACTCAANNN; match class: 1 | TF:M12456_1 | 2512 | 3.30E-04 | 291 | 47 |
| GO:BP | supramolecular fiber organization | GO:0097435 | 722 | 3.40E-04 | 291 | 22 |
| TF | Factor: GKLF; motif: GCCMCRCCCNNN | TF:M01588 | 8294 | 3.40E-04 | 291 | 108 |
| TF | Factor: RXRA; motif: RRGGTCATGACCYY; match class: 1 | TF:M04490_1 | 3016 | 3.40E-04 | 291 | 53 |
| TF | Factor: E2F-2; motif: NWTTTGGCGCCAWWNN; match class: 1 | TF:M11530_1 | 12570 | 3.50E-04 | 291 | 147 |
| TF | Factor: TGIF; motif: TGACAGS | TF:M10101 | 4444 | 3.50E-04 | 291 | 69 |
| TF | Factor: ZNF394; motif: NRARWRGAANNNAMWGNAAK | TF:M10147 | 4537 | 3.50E-04 | 291 | 70 |
| TF | Factor: GLI; motif: NGACCMCCCAN; match class: 1 | TF:M07290_1 | 1730 | 3.60E-04 | 291 | 37 |
| TF | Factor: EGR3; motif: NCMCRCCCACTNMN | TF:M08877 | 5486 | 3.60E-04 | 291 | 80 |
| TF | Factor: WT1; motif: NNGGGNGGGSGN | TF:M07436 | 6072 | 3.70E-04 | 291 | 86 |
| TF | Factor: CTCF; motif: NNYGCCCYCTRSTGGN | TF:M09744 | 3458 | 3.70E-04 | 291 | 58 |
| TF | Factor: AP-4; motif: NCAGCTGYNGNCN | TF:M01860 | 5298 | 3.70E-04 | 291 | 78 |
| TF | Factor: PPARalpha; motif: NWSTRGGNSAAAGGTCA | TF:M10037 | 5298 | 3.70E-04 | 291 | 78 |
| GO:BP | mesenchyme morphogenesis | GO:0072132 | 51 | 3.80E-04 | 291 | 7 |
| TF | Factor: EVX1; motif: NTCATTAN | TF:M10864 | 4360 | 3.80E-04 | 291 | 68 |
| TF | Factor: SNA; motif: NNRGCAGGTGCNNNN | TF:M10528 | 3905 | 3.80E-04 | 291 | 63 |
| TF | Factor: T3R-beta; motif: NRGGTCAAAGGTCRN; match class: 1 | TF:M11814_1 | 6374 | 3.80E-04 | 291 | 89 |
| TF | Factor: Pax-3; motif: NNNNNNCGTCACGSTYNNNNN | TF:M00327 | 11907 | 3.80E-04 | 291 | 141 |
| GO:MF | cytokine receptor activity | GO:0004896 | 85 | 3.90E-04 | 291 | 8 |
| GO:BP | cellular divalent inorganic cation homeostasis | GO:0072503 | 444 | 3.90E-04 | 291 | 17 |
| TF | Factor: ZXDA; motif: NGGGGTC | TF:M06190 | 5303 | 3.90E-04 | 291 | 78 |
| TF | Factor: ZXDB; motif: NGGGGTC | TF:M06191 | 5303 | 3.90E-04 | 291 | 78 |
| KEGG | ECM-receptor interaction | KEGG:04512 | 81 | 4.00E-04 | 291 | 7 |
| TF | Factor: ER-beta; motif: NRGGTCANKSTGACCTNN; match class: 1 | TF:M09610_1 | 1369 | 4.00E-04 | 291 | 32 |
| TF | Factor: NF-E4; motif: GTGAGGS; match class: 1 | TF:M08826_1 | 4182 | 4.00E-04 | 291 | 66 |
| TF | Factor: Oct-1; motif: TATGCAAATN | TF:M00342 | 2446 | 4.00E-04 | 291 | 46 |
| TF | Factor: ZNF563; motif: NGNNTCMTNNCNGGCAGCTGY | TF:M10158 | 5212 | 4.00E-04 | 291 | 77 |
| TF | Factor: HEY1; motif: NYYYYATCTNN | TF:M12614 | 4742 | 4.10E-04 | 291 | 72 |
| TF | Factor: EAR2; motif: YGNNCTTTGNCCTK | TF:M01728 | 5407 | 4.10E-04 | 291 | 79 |
| GO:BP | cation homeostasis | GO:0055080 | 671 | 4.20E-04 | 291 | 21 |
| TF | Factor: C/EBPbeta; motif: NNTTKCNNMAN | TF:M07315 | 2449 | 4.20E-04 | 291 | 46 |
| TF | Factor: Elk-1:ETV7; motif: ANSCGGACGGATDTCCGGNT | TF:M08214 | 7191 | 4.20E-04 | 291 | 97 |
| GO:BP | macromolecule metabolic process | GO:0043170 | 9254 | 4.30E-04 | 291 | 116 |
| GO:BP | positive regulation of cellular biosynthetic process | GO:0031328 | 1776 | 4.30E-04 | 291 | 37 |
| KEGG | African trypanosomiasis | KEGG:05143 | 31 | 4.30E-04 | 291 | 5 |
| TF | Factor: C/EBPalpha; motif: NNNNNTTKCNNAAN | TF:M07037 | 2783 | 4.30E-04 | 291 | 50 |
| GO:MF | cell adhesion molecule binding | GO:0050839 | 535 | 4.40E-04 | 291 | 18 |
| GO:MF | molecular function regulator | GO:0098772 | 1692 | 4.40E-04 | 291 | 35 |
| GO:BP | T cell activation | GO:0042110 | 448 | 4.40E-04 | 291 | 17 |
| GO:BP | response to abiotic stimulus | GO:0009628 | 1055 | 4.40E-04 | 291 | 27 |
| REAC | Interleukin-10 signaling | REAC:R-HSA-6783783 | 41 | 4.40E-04 | 291 | 6 |
| TF | Factor: Pax-2; motif: NNNNGTCANGNRTKANNNN | TF:M00098 | 6494 | 4.40E-04 | 291 | 90 |
| TF | Factor: LXR;; motif: NTGACCKNNAGTRACCYNN | TF:M03795 | 5609 | 4.40E-04 | 291 | 81 |
| TF | Factor: SF-1; motif: NYYCAAGGYCA | TF:M10084 | 3390 | 4.50E-04 | 291 | 57 |
| TF | Factor: MAFB; motif: NTCAGCN; match class: 1 | TF:M08888_1 | 2789 | 4.60E-04 | 291 | 50 |
| TF | Factor: AP-2beta; motif: NSCCYNRGGSN | TF:M11477 | 7511 | 4.60E-04 | 291 | 100 |
| TF | Factor: NF1B; motif: CYTGGCNYNCWGCCAN; match class: 1 | TF:M09762_1 | 1309 | 4.70E-04 | 291 | 31 |
| TF | Factor: P53; motif: NNNGRCATGCCCAGRCATGYCY | TF:M12647 | 5044 | 4.80E-04 | 291 | 75 |
| TF | Factor: PPARgamma:RXRalpha,; motif: AAGTAGGTCACNGTGACCYACTT; match class: 1 | TF:M00515_1 | 3841 | 4.80E-04 | 291 | 62 |
| TF | Factor: Sp1; motif: RGGGMGGRGSNGGGG | TF:M10529 | 6507 | 4.80E-04 | 291 | 90 |
| TF | Factor: Octamer; motif: TNATTTGCATN | TF:M00795 | 3752 | 4.90E-04 | 291 | 61 |
| TF | Factor: Fra-1; motif: NATGAWTCAYN | TF:M11286 | 2546 | 4.90E-04 | 291 | 47 |
| TF | Factor: MafG; motif: CMATGACTCAGCAGA | TF:M07048 | 5916 | 4.90E-04 | 291 | 84 |
| KEGG | Proteoglycans in cancer | KEGG:05205 | 197 | 5.00E-04 | 291 | 10 |
| TF | Factor: E2F3; motif: NNRGMKGGAR; match class: 1 | TF:M12598_1 | 6610 | 5.00E-04 | 291 | 91 |
| TF | Factor: c-MAF; motif: CNNNCTCAGCA | TF:M03816 | 4956 | 5.10E-04 | 291 | 74 |
| TF | Factor: WT1; motif: NGCGGGGGGGTSMMCYN | TF:M05327 | 5338 | 5.10E-04 | 291 | 78 |
| TF | Factor: RelA-p65; motif: GGGRNTTTCCM | TF:M09668 | 3228 | 5.10E-04 | 291 | 55 |
| TF | Factor: Sp1; motif: NGGGGGCGGGGYN | TF:M00196 | 9855 | 5.20E-04 | 291 | 122 |
| TF | Factor: SAP-1a; motif: CWTCCKGT | TF:M03844 | 2306 | 5.20E-04 | 291 | 44 |
| TF | Factor: ER71; motif: CWTCCTGT | TF:M07282 | 2306 | 5.20E-04 | 291 | 44 |
| GO:BP | regulation of immune response | GO:0050776 | 741 | 5.30E-04 | 291 | 22 |
| GO:BP | negative regulation of cell communication | GO:0010648 | 1273 | 5.30E-04 | 291 | 30 |
| TF | Factor: IRF-4; motif: AAGTTTC; match class: 1 | TF:M04855_1 | 1681 | 5.30E-04 | 291 | 36 |
| TF | Factor: Six-1; motif: CTCARRTTWCN | TF:M07466 | 2887 | 5.30E-04 | 291 | 51 |
| TF | Factor: JunD; motif: NRTGACGTCATS | TF:M11270 | 7948 | 5.30E-04 | 291 | 104 |
| TF | Factor: GCMa:C/EBPbeta; motif: ATGCGGGTNNRTTGCGCAAY | TF:M08575 | 5928 | 5.40E-04 | 291 | 84 |
| GO:BP | inorganic ion homeostasis | GO:0098771 | 682 | 5.50E-04 | 291 | 21 |
| GO:BP | negative regulation of signaling | GO:0023057 | 1275 | 5.50E-04 | 291 | 30 |
| GO:BP | neutrophil chemotaxis | GO:0030593 | 79 | 5.60E-04 | 291 | 8 |
| GO:BP | cell differentiation involved in kidney development | GO:0061005 | 54 | 5.60E-04 | 291 | 7 |
| TF | Factor: ER-beta; motif: ANNSTGACCYRGNN | TF:M03821 | 5542 | 5.60E-04 | 291 | 80 |
| TF | Factor: HFH8; motif: NNNTGTTTATNTR | TF:M00294 | 2978 | 5.60E-04 | 291 | 52 |
| TF | Factor: AP-2alpha; motif: NSCCNCRGGSN | TF:M07348 | 7543 | 5.60E-04 | 291 | 100 |
| TF | Factor: RARA; motif: AGGTCANNYAAAGGTCA | TF:M08019 | 4689 | 5.80E-04 | 291 | 71 |
| TF | Factor: Sp1; motif: GGGGCGGGGC | TF:M00931 | 9875 | 5.80E-04 | 291 | 122 |
| GO:MF | identical protein binding | GO:0042802 | 1948 | 5.90E-04 | 291 | 38 |
| GO:BP | cartilage development | GO:0051216 | 179 | 5.90E-04 | 291 | 11 |
| GO:BP | regulation of DNA-binding transcription factor activity | GO:0051090 | 406 | 6.00E-04 | 291 | 16 |
| GO:BP | immune effector process | GO:0002252 | 513 | 6.10E-04 | 291 | 18 |
| GO:CC | fibrillar collagen trimer | GO:0005583 | 12 | 6.20E-04 | 291 | 4 |
| GO:CC | banded collagen fibril | GO:0098643 | 12 | 6.20E-04 | 291 | 4 |
| TF | Factor: COUP-TF1; motif: RAGGTCANTGACCTY | TF:M11745 | 5556 | 6.20E-04 | 291 | 80 |
| GO:CC | ficolin-1-rich granule | GO:0101002 | 175 | 6.30E-04 | 291 | 10 |
| TF | Factor: Nkx2-3; motif: NNCGTTRWS | TF:M10638 | 9672 | 6.30E-04 | 291 | 120 |
| TF | Factor: IRF-8; motif: NYGAAASYGAAACYN | TF:M11682 | 3251 | 6.50E-04 | 291 | 55 |
| GO:BP | positive regulation of biosynthetic process | GO:0009891 | 1807 | 6.60E-04 | 291 | 37 |
| TF | Factor: En-2; motif: NTCRTTARN | TF:M10858 | 7670 | 6.60E-04 | 291 | 101 |
| GO:BP | divalent inorganic cation homeostasis | GO:0072507 | 462 | 6.70E-04 | 291 | 17 |
| GO:BP | hemopoiesis | GO:0030097 | 815 | 6.80E-04 | 291 | 23 |
| TF | Factor: POU3F2; motif: NTATGCWAATKAG | TF:M11909 | 4151 | 6.80E-04 | 291 | 65 |
| TF | Factor: SMAD; motif: TNGNCAGACWN | TF:M00974 | 3788 | 6.80E-04 | 291 | 61 |
| TF | Factor: AP-2gamma; motif: NNNNWGCCYNCRGSCN | TF:M07349 | 2998 | 6.90E-04 | 291 | 52 |
| TF | Factor: TR4; motif: NCCCTGACCYB | TF:M08906 | 3971 | 6.90E-04 | 291 | 63 |
| TF | Factor: TWIST; motif: CACCTGG; match class: 1 | TF:M03582_1 | 2249 | 7.00E-04 | 291 | 43 |
| TF | Factor: GR; motif: RGNACANMNTGTNCY; match class: 1 | TF:M09941_1 | 1701 | 7.00E-04 | 291 | 36 |
| KEGG | Relaxin signaling pathway | KEGG:04926 | 123 | 7.10E-04 | 291 | 8 |
| TF | Factor: Churchill; motif: CGGGNN; match class: 1 | TF:M00986_1 | 9909 | 7.10E-04 | 291 | 122 |
| TF | Factor: SMAD5; motif: GSGGCAGM; match class: 1 | TF:M03846_1 | 2251 | 7.20E-04 | 291 | 43 |
| TF | Factor: HES-5; motif: NCACACKY; match class: 1 | TF:M11069_1 | 3616 | 7.30E-04 | 291 | 59 |
| TF | Factor: NFATc1; motif: NTTTCCRTNNAYGGAAAN | TF:M11979 | 7686 | 7.30E-04 | 291 | 101 |
| GO:BP | regulation of epithelial cell proliferation | GO:0050678 | 313 | 7.40E-04 | 291 | 14 |
| TF | Factor: NF-1C; motif: NYTGGCNNYNNGCCARN; match class: 1 | TF:M10002_1 | 1481 | 7.40E-04 | 291 | 33 |
| TF | Factor: RUNX3; motif: NRACCGCANWAACCRCAN | TF:M04110 | 8526 | 7.50E-04 | 291 | 109 |
| TF | Factor: STAT3; motif: NNYTTCCMRGAA | TF:M10081 | 3093 | 7.60E-04 | 291 | 53 |
| GO:BP | cellular response to chemical stress | GO:0062197 | 314 | 7.70E-04 | 291 | 14 |
| TF | Factor: PR; motif: NNNNNNRGNACNNKNTGTTCTNNNNNN | TF:M00957 | 3356 | 7.70E-04 | 291 | 56 |
| TF | Factor: NF-kappaB; motif: NGGGANTTYCCMNNNN | TF:M00774 | 1632 | 7.70E-04 | 291 | 35 |
| GO:BP | muscle cell proliferation | GO:0033002 | 184 | 7.80E-04 | 291 | 11 |
| TF | Factor: TBP; motif: NGNNTATAAAA | TF:M03581 | 5782 | 7.80E-04 | 291 | 82 |
| TF | Factor: E2F-1; motif: NTTSGCGG | TF:M00430 | 7491 | 7.90E-04 | 291 | 99 |
| TF | Factor: RelA-p65; motif: AAATCCCCT | TF:M04814 | 3986 | 7.90E-04 | 291 | 63 |
| TF | Factor: TCF-3; motif: CTTTGA; match class: 1 | TF:M03858_1 | 4541 | 8.00E-04 | 291 | 69 |
| TF | Factor: TCF-7; motif: TCAAAG; match class: 1 | TF:M00805_1 | 4541 | 8.00E-04 | 291 | 69 |
| TF | Factor: Egr; motif: GTGGGSGCRRS | TF:M00807 | 4079 | 8.00E-04 | 291 | 64 |
| TF | Factor: Sp1; motif: CCCCGCCCCN | TF:M00933 | 9282 | 8.10E-04 | 291 | 116 |
| TF | Factor: Erm:E2A; motif: CASGTGNNNCGGAAGNN; match class: 1 | TF:M08571_1 | 3898 | 8.10E-04 | 291 | 62 |
| GO:BP | regulation of heart contraction | GO:0008016 | 185 | 8.20E-04 | 291 | 11 |
| TF | Factor: C/EBP; motif: NNNTKNNGNAAN | TF:M00770 | 4639 | 8.30E-04 | 291 | 70 |
| TF | Factor: NF-kappaB; motif: NGGGGAMTTTCCNN | TF:M00194 | 2103 | 8.30E-04 | 291 | 41 |
| TF | Factor: E2F-4; motif: NNTTCCCGCCNN | TF:M04823 | 11934 | 8.30E-04 | 291 | 140 |
| REAC | Immunoregulatory interactions between a Lymphoid and a non-Lymphoid cell | REAC:R-HSA-198933 | 101 | 8.40E-04 | 291 | 8 |
| TF | Factor: AP-2beta; motif: NSCCNNNGGSN; match class: 1 | TF:M11478_1 | 7500 | 8.40E-04 | 291 | 99 |
| TF | Factor: Sox-6; motif: NARACAAAARN | TF:M03888 | 2932 | 8.50E-04 | 291 | 51 |
| TF | Factor: ctcf; motif: YGGCCACCAGRKGGCRSYN | TF:M09890 | 7814 | 8.60E-04 | 291 | 102 |
| GO:MF | anion transmembrane transporter activity | GO:0008509 | 294 | 8.70E-04 | 291 | 13 |
| GO:BP | regulation of wound healing | GO:0061041 | 114 | 8.70E-04 | 291 | 9 |
| REAC | Syndecan interactions | REAC:R-HSA-3000170 | 26 | 8.70E-04 | 291 | 5 |
| TF | Factor: GCMa; motif: RTGCGGGTN; match class: 1 | TF:M11595_1 | 5310 | 8.70E-04 | 291 | 77 |
| TF | Factor: ATF3; motif: CBCTGACGTCANCS | TF:M00513 | 5504 | 8.70E-04 | 291 | 79 |
| REAC | Erythrocytes take up carbon dioxide and release oxygen | REAC:R-HSA-1237044 | 12 | 8.80E-04 | 291 | 4 |
| REAC | O2/CO2 exchange in erythrocytes | REAC:R-HSA-1480926 | 12 | 8.80E-04 | 291 | 4 |
| TF | Factor: MEF-2C; motif: TATTTWT | TF:M02025 | 10824 | 8.80E-04 | 291 | 130 |
| TF | Factor: ZNF692; motif: SYNGGSCCCASCCNC; match class: 1 | TF:M09734_1 | 5798 | 8.80E-04 | 291 | 82 |
| TF | Factor: Sp3; motif: GGGGCGGGGSNN | TF:M07615 | 6593 | 8.90E-04 | 291 | 90 |
| TF | Factor: SP1; motif: GGCCCCGCCCCCN | TF:M12666 | 8342 | 8.90E-04 | 291 | 107 |
| GO:BP | regulation of macromolecule biosynthetic process | GO:0010556 | 3510 | 9.00E-04 | 291 | 57 |
| TF | Factor: Pbx; motif: NKTGATTGACRKSN | TF:M01967 | 4369 | 9.00E-04 | 291 | 67 |
| TF | Factor: CDP:SRF; motif: NCCWTAYAAGGTMNKRATCRATN; match class: 1 | TF:M08520_1 | 3199 | 9.10E-04 | 291 | 54 |
| GO:BP | negative regulation of cell differentiation | GO:0045596 | 585 | 9.20E-04 | 291 | 19 |
| TF | Factor: E2F-4; motif: TTTGGCGCCAAA | TF:M04519 | 4279 | 9.20E-04 | 291 | 66 |
| TF | Factor: SP2; motif: GNNGGGGGCGGGGSN | TF:M03807 | 8142 | 9.50E-04 | 291 | 105 |
| TF | Factor: STAT3; motif: TTCYGGGAAN | TF:M07065 | 4283 | 9.50E-04 | 291 | 66 |
| TF | Factor: NR1B1:RXR-ALPHA; motif: RRGGTCANNNNNRGGTCA | TF:M08964 | 6106 | 9.60E-04 | 291 | 85 |
| TF | Factor: RELA; motif: GGGRMTKYCCC; match class: 1 | TF:M12657_1 | 1353 | 9.70E-04 | 291 | 31 |
| GO:MF | transmembrane signaling receptor activity | GO:0004888 | 947 | 1.00E-03 | 291 | 24 |
| GO:BP | cellular response to organic cyclic compound | GO:0071407 | 531 | 1.00E-03 | 291 | 18 |
| GO:BP | heart morphogenesis | GO:0003007 | 230 | 1.00E-03 | 291 | 12 |
| GO:BP | skeletal system morphogenesis | GO:0048705 | 189 | 1.00E-03 | 291 | 11 |
| GO:BP | pericyte cell differentiation | GO:1904238 | 9 | 1.00E-03 | 291 | 4 |
| GO:BP | glomerulus development | GO:0032835 | 59 | 1.00E-03 | 291 | 7 |
| TF | Factor: COUP-TF1; motif: GRGGKSARAGGTCAGNG | TF:M09886 | 4855 | 1.00E-03 | 291 | 72 |
| TF | Factor: E2F-1; motif: NNNNGGCGGGAARN; match class: 1 | TF:M09892_1 | 8790 | 1.00E-03 | 291 | 111 |
| TF | Factor: NURR1:RXR-ALPHA; motif: NRGGTCRTTGACCYN; match class: 1 | TF:M08957_1 | 4480 | 1.00E-03 | 291 | 68 |
| TF | Factor: AP-2alpha; motif: NGCCYSNNGSN; match class: 1 | TF:M01857_1 | 4480 | 1.00E-03 | 291 | 68 |
| TF | Factor: Sp1; motif: NNGGGGCGGGGNN | TF:M00932 | 9977 | 1.10E-03 | 291 | 122 |
| TF | Factor: CTCF; motif: ACCAGGKGGC; match class: 1 | TF:M04727_1 | 1359 | 1.10E-03 | 291 | 31 |
| TF | Factor: C/EBPbeta; motif: NKNTTGCNYAAYNN | TF:M00117 | 3480 | 1.10E-03 | 291 | 57 |
| TF | Factor: NFATc1; motif: NTTTCCRTNNAYGGAAAN; match class: 1 | TF:M11979_1 | 7539 | 1.10E-03 | 291 | 99 |
| TF | Factor: CPBP; motif: GNNRGGGHGGGGNNGGGRN; match class: 1 | TF:M09973_1 | 6323 | 1.10E-03 | 291 | 87 |
| TF | Factor: POU2F1; motif: ATGCAAATN | TF:M07059 | 1361 | 1.10E-03 | 291 | 31 |
| TF | Factor: GATA-1; motif: NTGNNNNNNNSAGATAAGR | TF:M09621 | 4581 | 1.10E-03 | 291 | 69 |
| TF | Factor: Pax-6; motif: NNNNTTCACGCWTGANTKNNN | TF:M00097 | 9552 | 1.10E-03 | 291 | 118 |
| TF | Factor: Erg; motif: NACCGGATATCCGGTN; match class: 1 | TF:M11391_1 | 8275 | 1.10E-03 | 291 | 106 |
| GO:BP | cardiac chamber development | GO:0003205 | 153 | 1.20E-03 | 291 | 10 |
| GO:BP | chemotaxis | GO:0006935 | 538 | 1.20E-03 | 291 | 18 |
| GO:BP | cochlea morphogenesis | GO:0090103 | 21 | 1.20E-03 | 291 | 5 |
| GO:CC | cell surface | GO:0009986 | 741 | 1.20E-03 | 291 | 20 |
| KEGG | Fluid shear stress and atherosclerosis | KEGG:05418 | 132 | 1.20E-03 | 291 | 8 |
| TF | Factor: CP2/LBP-1c/LSF; motif: GCTGGNTNGNNCYNG; match class: 1 | TF:M00947_1 | 2373 | 1.20E-03 | 291 | 44 |
| TF | Factor: HIC1; motif: NNNGGKTGCCCSNNNNNN | TF:M01073 | 3847 | 1.20E-03 | 291 | 61 |
| TF | Factor: Pax-5; motif: NGTCACGCWTSANTGMNY | TF:M04064 | 9027 | 1.20E-03 | 291 | 113 |
| TF | Factor: E2F; motif: TTTCGCGC | TF:M00425 | 10329 | 1.20E-03 | 291 | 125 |
| TF | Factor: CLOCK; motif: NNCAYGYGYN | TF:M11046 | 2134 | 1.20E-03 | 291 | 41 |
| TF | Factor: IRF-4; motif: GAAARTA | TF:M01883 | 6944 | 1.20E-03 | 291 | 93 |
| TF | Factor: Dlx-5; motif: NTCRTTAN | TF:M10612 | 6843 | 1.20E-03 | 291 | 92 |
| TF | Factor: Dlx-7; motif: NTCRTTAN | TF:M10603 | 6843 | 1.20E-03 | 291 | 92 |
| TF | Factor: TIEG1; motif: NCCCNSNCCCCGCCCCC; match class: 1 | TF:M12351_1 | 7871 | 1.20E-03 | 291 | 102 |
| GO:BP | ureteric bud development | GO:0001657 | 88 | 1.30E-03 | 291 | 8 |
| GO:BP | cellular response to peptide | GO:1901653 | 329 | 1.30E-03 | 291 | 14 |
| GO:BP | taxis | GO:0042330 | 542 | 1.30E-03 | 291 | 18 |
| GO:BP | regulation of RNA biosynthetic process | GO:2001141 | 3024 | 1.30E-03 | 291 | 51 |
| GO:CC | supramolecular complex | GO:0099080 | 1157 | 1.30E-03 | 291 | 26 |
| REAC | Muscle contraction | REAC:R-HSA-397014 | 183 | 1.30E-03 | 291 | 10 |
| TF | Factor: ETS1; motif: GCCGGAWGTACTTCCGGN | TF:M03978 | 11000 | 1.30E-03 | 291 | 131 |
| TF | Factor: pax-2; motif: NCGTCACGCNYSRNYGCNYN | TF:M11877 | 10450 | 1.30E-03 | 291 | 126 |
| TF | Factor: KLF3; motif: NNNNNNGGGCGGGGCNNGN | TF:M09970 | 7465 | 1.30E-03 | 291 | 98 |
| GO:BP | cardiac muscle tissue development | GO:0048738 | 195 | 1.40E-03 | 291 | 11 |
| GO:BP | mesonephric tubule development | GO:0072164 | 89 | 1.40E-03 | 291 | 8 |
| GO:BP | mesonephric epithelium development | GO:0072163 | 89 | 1.40E-03 | 291 | 8 |
| GO:BP | regulation of proteolysis | GO:0030162 | 662 | 1.40E-03 | 291 | 20 |
| GO:CC | endoplasmic reticulum | GO:0005783 | 1854 | 1.40E-03 | 291 | 35 |
| GO:CC | tertiary granule | GO:0070820 | 151 | 1.40E-03 | 291 | 9 |
| TF | Factor: CEBPD; motif: RTTRCGCAAY | TF:M04202 | 4325 | 1.40E-03 | 291 | 66 |
| TF | Factor: IRF-2; motif: NGAAASYGAAAS | TF:M11665 | 2063 | 1.40E-03 | 291 | 40 |
| TF | Factor: Ik-2; motif: NNNTGGGAWNNC | TF:M00087 | 3067 | 1.40E-03 | 291 | 52 |
| GO:BP | transmembrane receptor protein serine/threonine kinase signaling pathway | GO:0007178 | 333 | 1.50E-03 | 291 | 14 |
| TF | Factor: CTCF; motif: WGCGCCMYCTAGYGGYN | TF:M03895 | 8633 | 1.50E-03 | 291 | 109 |
| TF | Factor: STAT3; motif: NTTCCYGGAAN | TF:M09664 | 4899 | 1.50E-03 | 291 | 72 |
| TF | Factor: AP-2alpha; motif: NGCCTSAGGCN; match class: 1 | TF:M11480_1 | 5380 | 1.50E-03 | 291 | 77 |
| TF | Factor: BCL-6; motif: NYGCTTTCKAGGAANN | TF:M12341 | 5187 | 1.50E-03 | 291 | 75 |
| TF | Factor: RUNX2; motif: NRACCGCAAACCGCAN; match class: 1 | TF:M04106_1 | 7901 | 1.50E-03 | 291 | 102 |
| TF | Factor: C/EBPdelta; motif: TKNNGCAATN | TF:M07316 | 2731 | 1.50E-03 | 291 | 48 |
| TF | Factor: Smad2; motif: TGTCTGNCWCCT; match class: 1 | TF:M10059_1 | 1029 | 1.50E-03 | 291 | 26 |
| TF | Factor: TEF-1; motif: NRCATWCCN | TF:M12042 | 4433 | 1.50E-03 | 291 | 67 |
| TF | Factor: ZNF432; motif: NCAGNRCCNSRGRCAGC; match class: 1 | TF:M12704_1 | 4435 | 1.60E-03 | 291 | 67 |
| TF | Factor: PEBP2beta; motif: TGTGGTY | TF:M03841 | 6476 | 1.60E-03 | 291 | 88 |
| TF | Factor: PEA3; motif: ACAGGAAGGAAGTN | TF:M11429 | 4815 | 1.60E-03 | 291 | 71 |
| TF | Factor: GR; motif: RGNACANKNTGTNCY; match class: 1 | TF:M09625_1 | 3523 | 1.60E-03 | 291 | 57 |
| TF | Factor: ctcf; motif: CCNCNAGRKGGCRSTN | TF:M07249 | 6280 | 1.60E-03 | 291 | 86 |
| TF | Factor: DBP; motif: TTWTGYAA | TF:M01872 | 2158 | 1.60E-03 | 291 | 41 |
| TF | Factor: TEF-3:Elf-1; motif: RGAATGCGGAAGTN | TF:M08415 | 8231 | 1.60E-03 | 291 | 105 |
| TF | Factor: Smad4; motif: NCAGACAN; match class: 1 | TF:M07368_1 | 423 | 1.60E-03 | 291 | 16 |
| GO:BP | detoxification | GO:0098754 | 123 | 1.70E-03 | 291 | 9 |
| GO:BP | ion transport | GO:0006811 | 1420 | 1.70E-03 | 291 | 31 |
| GO:BP | regulation of defense response | GO:0031347 | 551 | 1.70E-03 | 291 | 18 |
| GO:BP | reproductive structure development | GO:0048608 | 387 | 1.70E-03 | 291 | 15 |
| TF | Factor: C/EBPbeta; motif: ATTGCGYAAT | TF:M07413 | 2826 | 1.70E-03 | 291 | 49 |
| TF | Factor: TFAP2C; motif: NGCCCNNRGGCA; match class: 1 | TF:M04152_1 | 5107 | 1.70E-03 | 291 | 74 |
| TF | Factor: GKLF; motif: NNCCMCRCCCN | TF:M12173 | 9407 | 1.70E-03 | 291 | 116 |
| TF | Factor: HSF1; motif: GAANNTTCTRGNAN | TF:M02017 | 3440 | 1.70E-03 | 291 | 56 |
| TF | Factor: E2F-3; motif: GGCGGGN; match class: 1 | TF:M02089_1 | 9086 | 1.70E-03 | 291 | 113 |
| TF | Factor: AP-2alpha; motif: NGCCTSAGGCN | TF:M11480 | 5694 | 1.70E-03 | 291 | 80 |
| TF | Factor: Oct-2; motif: NTATGCWAATN | TF:M11896 | 4354 | 1.70E-03 | 291 | 66 |
| TF | Factor: ER-alpha; motif: AGGTCANMNTGACC | TF:M04738 | 3712 | 1.70E-03 | 291 | 59 |
| TF | Factor: TGIF; motif: TGACAGS; match class: 1 | TF:M10101_1 | 712 | 1.70E-03 | 291 | 21 |
| GO:BP | gene expression | GO:0010467 | 6162 | 1.80E-03 | 291 | 84 |
| GO:BP | leukocyte cell-cell adhesion | GO:0007159 | 338 | 1.80E-03 | 291 | 14 |
| GO:BP | proteolysis | GO:0006508 | 1576 | 1.80E-03 | 291 | 33 |
| REAC | Scavenging of heme from plasma | REAC:R-HSA-2168880 | 14 | 1.80E-03 | 291 | 4 |
| TF | Factor: POU2F1; motif: NNATTTGCATNN | TF:M03561 | 1770 | 1.80E-03 | 291 | 36 |
| TF | Factor: BTEB3; motif: CCNNSCCNSCCCCKCCCCC; match class: 1 | TF:M09826_1 | 7207 | 1.80E-03 | 291 | 95 |
| TF | Factor: GR; motif: RGWACATWAYGTWCY; match class: 1 | TF:M11846_1 | 3358 | 1.80E-03 | 291 | 55 |
| GO:BP | ear development | GO:0043583 | 201 | 1.90E-03 | 291 | 11 |
| GO:BP | reproductive system development | GO:0061458 | 390 | 1.90E-03 | 291 | 15 |
| GO:BP | negative regulation of epithelial cell apoptotic process | GO:1904036 | 41 | 1.90E-03 | 291 | 6 |
| TF | Factor: PLAGL2; motif: AAGGACC | TF:M05548 | 3631 | 1.90E-03 | 291 | 58 |
| TF | Factor: Pax-5; motif: GTYAYGCTTSRCTGVNYN | TF:M04754 | 4648 | 1.90E-03 | 291 | 69 |
| TF | Factor: TBP; motif: MTATAAAARS | TF:M10088 | 3905 | 1.90E-03 | 291 | 61 |
| GO:BP | mesonephros development | GO:0001823 | 93 | 2.00E-03 | 291 | 8 |
| GO:BP | regulation of system process | GO:0044057 | 500 | 2.00E-03 | 291 | 17 |
| GO:BP | glomerulus vasculature development | GO:0072012 | 23 | 2.00E-03 | 291 | 5 |
| TF | Factor: Elk-1:Pax-9; motif: ACCGGAACYACGCWYSANTG | TF:M08226 | 10969 | 2.00E-03 | 291 | 130 |
| TF | Factor: Sp1; motif: NGGGGGCGGGGYN; match class: 1 | TF:M00196_1 | 5813 | 2.00E-03 | 291 | 81 |
| TF | Factor: SREBP-1; motif: RTCACCCCAY | TF:M07405 | 5912 | 2.00E-03 | 291 | 82 |
| TF | Factor: ZXDB; motif: NAGGGTG; match class: 1 | TF:M06202_1 | 1701 | 2.00E-03 | 291 | 35 |
| TF | Factor: ZXDA; motif: NAGGGTG; match class: 1 | TF:M06203_1 | 1701 | 2.00E-03 | 291 | 35 |
| TF | Factor: C/EBPbeta; motif: NATTGCRYAAYN | TF:M09597 | 2506 | 2.00E-03 | 291 | 45 |
| TF | Factor: NR1B2; motif: RAGGTCATGACCTN | TF:M11803 | 2760 | 2.00E-03 | 291 | 48 |
| GO:BP | regulation of catalytic activity | GO:0050790 | 2138 | 2.10E-03 | 291 | 40 |
| TF | Factor: TTF-1; motif: NTKGAGTGSN | TF:M10007 | 3916 | 2.10E-03 | 291 | 61 |
| TF | Factor: c-Jun; motif: NATGACGTCAYN; match class: 1 | TF:M11266_1 | 5332 | 2.10E-03 | 291 | 76 |
| GO:BP | RNA biosynthetic process | GO:0032774 | 3164 | 2.20E-03 | 291 | 52 |
| TF | Factor: NR1B2; motif: NTGACCY; match class: 1 | TF:M02111_1 | 2939 | 2.20E-03 | 291 | 50 |
| TF | Factor: rfx3:SRF; motif: TRGCAACNNNNNCCNWATANGGN; match class: 1 | TF:M08623_1 | 7240 | 2.20E-03 | 291 | 95 |
| GO:BP | regulation of nucleobase-containing compound metabolic process | GO:0019219 | 3523 | 2.30E-03 | 291 | 56 |
| GO:BP | myeloid leukocyte activation | GO:0002274 | 205 | 2.30E-03 | 291 | 11 |
| TF | Factor: CTCF; motif: NNNGCCASCAGRKGGCRSNN | TF:M01200 | 3651 | 2.30E-03 | 291 | 58 |
| TF | Factor: HMGIY; motif: NGWWATTN | TF:M01653 | 5150 | 2.30E-03 | 291 | 74 |
| TF | Factor: Oct-2; motif: NNNATTATGCAW | TF:M11894 | 2946 | 2.30E-03 | 291 | 50 |
| GO:BP | leukocyte differentiation | GO:0002521 | 507 | 2.40E-03 | 291 | 17 |
| TF | Factor: MEL1; motif: GATGAG; match class: 1 | TF:M08793_1 | 4116 | 2.40E-03 | 291 | 63 |
| TF | Factor: FEZF1; motif: NYTGYYCTTTTN | TF:M09919 | 2030 | 2.40E-03 | 291 | 39 |
| GO:BP | morphogenesis of a branching epithelium | GO:0061138 | 166 | 2.50E-03 | 291 | 10 |
| TF | Factor: GATA-5; motif: TATCTN; match class: 1 | TF:M02006_1 | 2441 | 2.50E-03 | 291 | 44 |
| TF | Factor: E2F-1; motif: TTTGGCGCCAAA; match class: 1 | TF:M04516_1 | 9692 | 2.50E-03 | 291 | 118 |
| TF | Factor: PU.1; motif: NNNNYYYACTTCCTCTTTY | TF:M01172 | 3663 | 2.50E-03 | 291 | 58 |
| GO:BP | regulation of muscle system process | GO:0090257 | 208 | 2.60E-03 | 291 | 11 |
| TF | Factor: KLF8; motif: NGGGGTGYGG | TF:M08818 | 1417 | 2.60E-03 | 291 | 31 |
| TF | Factor: Foxc1; motif: TTVYTTTNW | TF:M07254 | 6449 | 2.60E-03 | 291 | 87 |
| TF | Factor: ATF-3; motif: GATGAYGTCATN | TF:M11288 | 4312 | 2.60E-03 | 291 | 65 |
| TF | Factor: PHB; motif: NCCCAGCCCCY; match class: 1 | TF:M12725_1 | 4127 | 2.60E-03 | 291 | 63 |
| GO:CC | platelet alpha granule lumen | GO:0031093 | 59 | 2.70E-03 | 291 | 6 |
| TF | Factor: NFATc1; motif: TTTCCAYWRTGGAAA | TF:M04052 | 5658 | 2.70E-03 | 291 | 79 |
| TF | Factor: Fra-1; motif: GRTGACGTCATC | TF:M11285 | 6557 | 2.70E-03 | 291 | 88 |
| TF | Factor: T3R-beta; motif: NRRGGTCRTGACCYYN | TF:M11817 | 4694 | 2.70E-03 | 291 | 69 |
| TF | Factor: ER-alpha; motif: NAGGTCANSNTGACCYN | TF:M11845 | 3225 | 2.70E-03 | 291 | 53 |
| GO:BP | regulation of epithelial cell differentiation | GO:0030856 | 131 | 2.80E-03 | 291 | 9 |
| TF | Factor: AP-2gamma:Dlx-3; motif: NSCCYNNRGGCANNNNYAATTA | TF:M08660 | 3138 | 2.80E-03 | 291 | 52 |
| TF | Factor: Sox-10; motif: MAANRRNNNCWTTGTT | TF:M10065 | 5861 | 2.80E-03 | 291 | 81 |
| TF | Factor: ZGPAT; motif: GRGGCWGNGGNG; match class: 1 | TF:M09739_1 | 2537 | 2.80E-03 | 291 | 45 |
| TF | Factor: NF-kappaB; motif: GGGRATTTCC | TF:M00052 | 1650 | 2.80E-03 | 291 | 34 |
| GO:CC | focal adhesion | GO:0005925 | 413 | 2.90E-03 | 291 | 14 |
| TF | Factor: MZF-1; motif: KNGNKAGGGGNAA | TF:M00084 | 5964 | 2.90E-03 | 291 | 82 |
| TF | Factor: ER71:C/EBPdelta; motif: NNCGGAWRTTRCGYAAN | TF:M08246 | 7178 | 2.90E-03 | 291 | 94 |
| TF | Factor: BRN1; motif: NANNTATGCATAATNNA | TF:M01324 | 4046 | 2.90E-03 | 291 | 62 |
| TF | Factor: CDX-2; motif: TTTATN; match class: 1 | TF:M02087_1 | 3954 | 2.90E-03 | 291 | 61 |
| GO:BP | regulation of transcription, DNA-templated | GO:0006355 | 3018 | 3.00E-03 | 291 | 50 |
| GO:BP | regulation of nucleic acid-templated transcription | GO:1903506 | 3019 | 3.00E-03 | 291 | 50 |
| TF | Factor: IRF-8; motif: NCGAAACCGAAACYN | TF:M11683 | 4049 | 3.00E-03 | 291 | 62 |
| TF | Factor: Smad1; motif: NGGCAGACN | TF:M03845 | 3958 | 3.00E-03 | 291 | 61 |
| TF | Factor: c-Myc; motif: NCCACGTGCNN | TF:M09992 | 4899 | 3.00E-03 | 291 | 71 |
| GO:BP | cell adhesion mediated by integrin | GO:0033627 | 69 | 3.10E-03 | 291 | 7 |
| GO:BP | regulation of RNA metabolic process | GO:0051252 | 3290 | 3.10E-03 | 291 | 53 |
| TF | Factor: Fli-1:C/EBPdelta; motif: NNCGGAWATTGCGCAAT | TF:M08469 | 5776 | 3.10E-03 | 291 | 80 |
| TF | Factor: IRF-1; motif: TTCACTT | TF:M00747 | 4332 | 3.10E-03 | 291 | 65 |
| TF | Factor: CDP:T-bet; motif: NTCACACNYYRATCRATM | TF:M08203 | 5093 | 3.10E-03 | 291 | 73 |
| TF | Factor: Cdx-2; motif: NRTCGTAANNNN | TF:M10849 | 9841 | 3.10E-03 | 291 | 119 |
| TF | Factor: Oct-2; motif: NNNATTTGCATRT | TF:M07120 | 3962 | 3.10E-03 | 291 | 61 |
| GO:BP | kidney vasculature development | GO:0061440 | 25 | 3.20E-03 | 291 | 5 |
| GO:BP | renal system vasculature development | GO:0061437 | 25 | 3.20E-03 | 291 | 5 |
| GO:BP | response to steroid hormone | GO:0048545 | 304 | 3.20E-03 | 291 | 13 |
| GO:BP | humoral immune response | GO:0006959 | 171 | 3.20E-03 | 291 | 10 |
| TF | Factor: TEAD4; motif: NRCATTCCWN | TF:M04145 | 1213 | 3.20E-03 | 291 | 28 |
| GO:BP | response to organic cyclic compound | GO:0014070 | 828 | 3.30E-03 | 291 | 22 |
| GO:BP | extrinsic apoptotic signaling pathway | GO:0097191 | 213 | 3.30E-03 | 291 | 11 |
| TF | Factor: C/EBPdelta; motif: NRTTGCGYAAYN | TF:M11321 | 4812 | 3.30E-03 | 291 | 70 |
| TF | Factor: Sox-9; motif: NNNNNACAAARGNNSMN; match class: 1 | TF:M07269_1 | 2893 | 3.30E-03 | 291 | 49 |
| TF | Factor: sp4; motif: NNGNARGRGGCGGRGCNNRR | TF:M10072 | 10068 | 3.30E-03 | 291 | 121 |
| TF | Factor: Erg; motif: NACCGGAWWTCCGGTN | TF:M11395 | 7927 | 3.30E-03 | 291 | 101 |
| GO:BP | regulation of endothelial cell apoptotic process | GO:2000351 | 45 | 3.40E-03 | 291 | 6 |
| GO:BP | regulation of extrinsic apoptotic signaling pathway via death domain receptors | GO:1902041 | 45 | 3.40E-03 | 291 | 6 |
| TF | Factor: SP2; motif: GGGCGGGAC | TF:M01783 | 8669 | 3.40E-03 | 291 | 108 |
| TF | Factor: LBP9; motif: ACYRGTNNNNACYRGT | TF:M11492 | 6898 | 3.40E-03 | 291 | 91 |
| TF | Factor: LHX4; motif: NNCRTTAN | TF:M11005 | 9424 | 3.40E-03 | 291 | 115 |
| TF | Factor: MEF-2D; motif: WAAATAR | TF:M02026 | 6393 | 3.50E-03 | 291 | 86 |
| TF | Factor: Pitx1; motif: NNNNRGGGATTAAMNNN | TF:M01484 | 2900 | 3.50E-03 | 291 | 49 |
| GO:BP | cellular response to stress | GO:0033554 | 1782 | 3.60E-03 | 291 | 35 |
| GO:CC | cell-substrate junction | GO:0030055 | 421 | 3.60E-03 | 291 | 14 |
| KEGG | Lipid and atherosclerosis | KEGG:05417 | 199 | 3.60E-03 | 291 | 9 |
| REAC | Cell surface interactions at the vascular wall | REAC:R-HSA-202733 | 123 | 3.60E-03 | 291 | 8 |
| TF | Factor: TFAP2A; motif: YGCCCNNRGGCN; match class: 1 | TF:M04146_1 | 3522 | 3.60E-03 | 291 | 56 |
| TF | Factor: GR; motif: GGTACAANNTGTYCTK; match class: 1 | TF:M00205_1 | 1220 | 3.60E-03 | 291 | 28 |
| TF | Factor: FXR; motif: NAGGTCAWNKN | TF:M08953 | 4074 | 3.70E-03 | 291 | 62 |
| TF | Factor: MEIS1A:HOXA9; motif: TGACAGKTTTAYGA | TF:M00420 | 4639 | 3.70E-03 | 291 | 68 |
| TF | Factor: SREBP-1; motif: RTCRCGTGAY; match class: 1 | TF:M11081_1 | 7116 | 3.70E-03 | 291 | 93 |
| GO:BP | negative regulation of cell adhesion | GO:0007162 | 261 | 3.80E-03 | 291 | 12 |
| GO:BP | positive regulation of intracellular signal transduction | GO:1902533 | 902 | 3.80E-03 | 291 | 23 |
| KEGG | Rheumatoid arthritis | KEGG:05323 | 78 | 3.80E-03 | 291 | 6 |
| TF | Factor: AML3; motif: NWAACCACRAAAACCACRAN | TF:M11999 | 5905 | 3.80E-03 | 291 | 81 |
| GO:BP | negative regulation of endothelial cell apoptotic process | GO:2000352 | 26 | 3.90E-03 | 291 | 5 |
| GO:BP | negative regulation of extrinsic apoptotic signaling pathway via death domain receptors | GO:1902042 | 26 | 3.90E-03 | 291 | 5 |
| GO:BP | mesenchymal cell differentiation | GO:0048762 | 217 | 4.00E-03 | 291 | 11 |
| TF | Factor: SP3; motif: NCCACGCCCMC | TF:M03921 | 3536 | 4.00E-03 | 291 | 56 |
| GO:BP | heart contraction | GO:0060047 | 218 | 4.10E-03 | 291 | 11 |
| GO:CC | cytoskeleton | GO:0005856 | 2118 | 4.10E-03 | 291 | 37 |
| GO:CC | actin cytoskeleton | GO:0015629 | 484 | 4.10E-03 | 291 | 15 |
| TF | Factor: PARP; motif: YDRGAAAWAS | TF:M01211 | 2156 | 4.10E-03 | 291 | 40 |
| TF | Factor: Tbx20; motif: SGAGGTGTGAGGSGR | TF:M07474 | 3181 | 4.10E-03 | 291 | 52 |
| TF | Factor: C-JUN:FRA-2; motif: NNRTGAGTCAYN | TF:M08940 | 3007 | 4.20E-03 | 291 | 50 |
| TF | Factor: ESRRA; motif: AAGGTCATNCAAGGTCA | TF:M04457 | 3008 | 4.30E-03 | 291 | 50 |
| TF | Factor: TFII-I; motif: NAGGAAGTGN; match class: 1 | TF:M04636_1 | 4849 | 4.30E-03 | 291 | 70 |
| GO:BP | transcription, DNA-templated | GO:0006351 | 3149 | 4.40E-03 | 291 | 51 |
| GO:BP | nucleic acid-templated transcription | GO:0097659 | 3150 | 4.40E-03 | 291 | 51 |
| TF | Factor: ER-alpha; motif: TGACCYN | TF:M03547 | 4003 | 4.40E-03 | 291 | 61 |
| GO:BP | odontogenesis of dentin-containing tooth | GO:0042475 | 73 | 4.50E-03 | 291 | 7 |
| KEGG | Estrogen signaling pathway | KEGG:04915 | 117 | 4.50E-03 | 291 | 7 |
| REAC | Phase I - Functionalization of compounds | REAC:R-HSA-211945 | 91 | 4.50E-03 | 291 | 7 |
| TF | Factor: Pet-1; motif: GCNGGAAGYG; match class: 1 | TF:M09918_1 | 6634 | 4.50E-03 | 291 | 88 |
| TF | Factor: HOXA7; motif: YCAATCT | TF:M01108 | 2497 | 4.50E-03 | 291 | 44 |
| GO:BP | cellular component organization | GO:0016043 | 6205 | 4.60E-03 | 291 | 83 |
| TF | Factor: HOXA10; motif: NGTCGTAAAAN | TF:M10795 | 5936 | 4.70E-03 | 291 | 81 |
| TF | Factor: NF-kappaB; motif: GGGGATYCCC; match class: 1 | TF:M00051_1 | 2586 | 4.70E-03 | 291 | 45 |
| TF | Factor: SREBP-2; motif: RTGGGGTGAY | TF:M07406 | 5054 | 4.70E-03 | 291 | 72 |
| GO:BP | cellular response to oxidative stress | GO:0034599 | 267 | 4.80E-03 | 291 | 12 |
| GO:BP | morphogenesis of a branching structure | GO:0001763 | 179 | 4.80E-03 | 291 | 10 |
| TF | Factor: ZF5; motif: NRNGNGCGCGCWN; match class: 1 | TF:M00333_1 | 11811 | 4.80E-03 | 291 | 136 |
| TF | Factor: Smad4; motif: TGTCTGN | TF:M01889 | 4482 | 4.80E-03 | 291 | 66 |
| TF | Factor: E2F-1; motif: WWTGGCGCCAAA; match class: 1 | TF:M04515_1 | 11473 | 4.80E-03 | 291 | 133 |
| TF | Factor: FLI-1; motif: CMGGAWGTSAN | TF:M01208 | 5544 | 4.80E-03 | 291 | 77 |
| TF | Factor: POU6F1; motif: NNNNATAATGAGSTNNN | TF:M01462 | 4868 | 5.00E-03 | 291 | 70 |
| TF | Factor: POU3F1; motif: RNYBCATTTGCATTWCAA | TF:M03842 | 4299 | 5.00E-03 | 291 | 64 |
| TF | Factor: AP-4; motif: AWCAGCTGWT | TF:M11208 | 3562 | 5.10E-03 | 291 | 56 |
| MIRNA | hsa-miR-29b-3p | MIRNA:hsa-miR-29b-3p | 249 | 5.10E-03 | 291 | 11 |
| TF | Factor: NeuroD; motif: NNSCWGCTGNSY | TF:M01288 | 4210 | 5.20E-03 | 291 | 63 |
| TF | Factor: MafB; motif: TGCTGASTNNN | TF:M09978 | 2768 | 5.20E-03 | 291 | 47 |
| GO:BP | positive regulation of RNA metabolic process | GO:0051254 | 1577 | 5.30E-03 | 291 | 32 |
| GO:BP | cellular response to hormone stimulus | GO:0032870 | 538 | 5.30E-03 | 291 | 17 |
| TF | Factor: ER-alpha; motif: GNCNNNNTGACCYN | TF:M07283 | 2597 | 5.30E-03 | 291 | 45 |
| TF | Factor: JunD; motif: NRTGAGTCAYN | TF:M09964 | 4213 | 5.30E-03 | 291 | 63 |
| TF | Factor: SP1; motif: NCCCCKCCCCC | TF:M07226 | 7901 | 5.30E-03 | 291 | 100 |
| GO:MF | ion binding | GO:0043167 | 5669 | 5.40E-03 | 291 | 76 |
| GO:BP | positive regulation of nucleobase-containing compound metabolic process | GO:0045935 | 1735 | 5.40E-03 | 291 | 34 |
| GO:BP | transmembrane transport | GO:0055085 | 1426 | 5.40E-03 | 291 | 30 |
| GO:BP | positive regulation of programmed cell death | GO:0043068 | 481 | 5.50E-03 | 291 | 16 |
| GO:MF | NAD+ nucleotidase, cyclic ADP-ribose generating | GO:0061809 | 16 | 5.60E-03 | 291 | 4 |
| GO:MF | NAD(P)+ nucleosidase activity | GO:0050135 | 16 | 5.60E-03 | 291 | 4 |
| GO:CC | hemoglobin complex | GO:0005833 | 7 | 5.60E-03 | 291 | 3 |
| GO:CC | haptoglobin-hemoglobin complex | GO:0031838 | 7 | 5.60E-03 | 291 | 3 |
| TF | Factor: NF-1C; motif: TTGGCNN | TF:M07300 | 5371 | 5.60E-03 | 291 | 75 |
| TF | Factor: LRF; motif: NGKGGGTSNCN | TF:M07387 | 4792 | 5.70E-03 | 291 | 69 |
| TF | Factor: Oct3; motif: WTATGCGCATAW; match class: 1 | TF:M11934_1 | 7810 | 5.80E-03 | 291 | 99 |
| TF | Factor: LBP-1; motif: CAGCTGS; match class: 1 | TF:M00644_1 | 3578 | 5.80E-03 | 291 | 56 |
| TF | Factor: HoxA5; motif: RTCATTAN | TF:M10704 | 4413 | 5.80E-03 | 291 | 65 |
| TF | Factor: Pax-5; motif: RRMSWGANWYCTNRAGCGKRACSRYNSM | TF:M00144 | 12083 | 6.00E-03 | 291 | 138 |
| GO:BP | heart process | GO:0003015 | 227 | 6.10E-03 | 291 | 11 |
| TF | Factor: CTCF; motif: NNRSYGCCMCCTGSTGGCCN | TF:M12593 | 8029 | 6.10E-03 | 291 | 101 |
| TF | Factor: LKLF; motif: GGGGTGGKSN; match class: 1 | TF:M07261_1 | 3767 | 6.10E-03 | 291 | 58 |
| TF | Factor: Oct-2; motif: NNNTATGCAAATNNNN | TF:M01368 | 1947 | 6.10E-03 | 291 | 37 |
| GO:BP | regulation of response to wounding | GO:1903034 | 144 | 6.20E-03 | 291 | 9 |
| GO:BP | ear morphogenesis | GO:0042471 | 108 | 6.20E-03 | 291 | 8 |
| GO:BP | innate immune response | GO:0045087 | 667 | 6.30E-03 | 291 | 19 |
| TF | Factor: STAT2; motif: NRGAAANNGAAACTNA | TF:M09733 | 2444 | 6.30E-03 | 291 | 43 |
| GO:BP | regulation of blood circulation | GO:1903522 | 228 | 6.40E-03 | 291 | 11 |
| GO:BP | organic cyclic compound biosynthetic process | GO:1901362 | 3736 | 6.40E-03 | 291 | 57 |
| GO:BP | endothelial cell apoptotic process | GO:0072577 | 50 | 6.40E-03 | 291 | 6 |
| TF | Factor: OCT-x; motif: CTNATTTGCATAY | TF:M00210 | 3408 | 6.40E-03 | 291 | 54 |
| TF | Factor: Pax-4; motif: SWAATWAN | TF:M04063 | 3957 | 6.40E-03 | 291 | 60 |
| GO:CC | cytoplasmic vesicle membrane | GO:0030659 | 1119 | 6.50E-03 | 291 | 24 |
| TF | Factor: GTF2IRD1-isoform2; motif: GGGATTRNR; match class: 1 | TF:M01229_1 | 7936 | 6.50E-03 | 291 | 100 |
| GO:BP | regulation of intracellular signal transduction | GO:1902531 | 1594 | 6.60E-03 | 291 | 32 |
| TF | Factor: Sp1; motif: GGGGCGGGGC; match class: 1 | TF:M00931_1 | 5788 | 6.60E-03 | 291 | 79 |
| TF | Factor: AP-1; motif: NTGASTCAG | TF:M00199 | 4054 | 6.60E-03 | 291 | 61 |
| TF | Factor: FOSB:JUND; motif: NNTNACTNATN | TF:M08928 | 5006 | 6.70E-03 | 291 | 71 |
| TF | Factor: FRA-2; motif: RTGANTCA | TF:M08914 | 3234 | 6.70E-03 | 291 | 52 |
| REAC | Transport of small molecules | REAC:R-HSA-382551 | 679 | 6.80E-03 | 291 | 18 |
| TF | Factor: P50; motif: GGRRANTCCCNN; match class: 1 | TF:M03557_1 | 1188 | 6.80E-03 | 291 | 27 |
| TF | Factor: Sox-9; motif: MACARWGNNNYCNTTNW | TF:M08838 | 3505 | 6.80E-03 | 291 | 55 |
| TF | Factor: ATF-2; motif: RRTGANGTCAY | TF:M09867 | 3596 | 6.80E-03 | 291 | 56 |
| GO:MF | cation binding | GO:0043169 | 4050 | 6.90E-03 | 291 | 59 |
| TF | Factor: ESE-1; motif: NTGTGCGGATGCN; match class: 1 | TF:M11385_1 | 4913 | 6.90E-03 | 291 | 70 |
| TF | Factor: AR; motif: GGTACANNRTGTTCT; match class: 1 | TF:M00481_1 | 1957 | 6.90E-03 | 291 | 37 |
| TF | Factor: WT1; motif: RGGNGGGGGAGGRGGNGGRG | TF:M10108 | 6095 | 6.90E-03 | 291 | 82 |
| TF | Factor: E2F2; motif: AAAAATGGCGCCAAAAWG | TF:M03958 | 9442 | 6.90E-03 | 291 | 114 |
| GO:BP | regulation of peptidase activity | GO:0052547 | 379 | 7.00E-03 | 291 | 14 |
| TF | Factor: TAFII250; motif: RARRWGGCGGMGGNGR | TF:M10086 | 8689 | 7.00E-03 | 291 | 107 |
| GO:MF | calcium ion binding | GO:0005509 | 653 | 7.10E-03 | 291 | 18 |
| GO:BP | metanephros development | GO:0001656 | 78 | 7.10E-03 | 291 | 7 |
| TF | Factor: AP-2beta; motif: GCNNNGGSCNGVGGGN; match class: 1 | TF:M01858_1 | 3063 | 7.10E-03 | 291 | 50 |
| GO:BP | negative regulation of endopeptidase activity | GO:0010951 | 187 | 7.20E-03 | 291 | 10 |
| TF | Factor: FAC1; motif: NNNCAMAACACRNA | TF:M00456 | 6812 | 7.30E-03 | 291 | 89 |
| TF | Factor: PATZ; motif: GGGGNGGGGGMKGGRRNGGNRN | TF:M10026 | 7954 | 7.30E-03 | 291 | 100 |
| TF | Factor: AR; motif: NNNNNNRGNACRNNGTGTTCTNNNNNN | TF:M00956 | 2207 | 7.30E-03 | 291 | 40 |
| TF | Factor: AP-2beta; motif: NSCCYNRGGSN; match class: 1 | TF:M11477_1 | 6305 | 7.30E-03 | 291 | 84 |
| TF | Factor: LXR-alpha; motif: NNRRGGTYACTNNAGGNCA | TF:M10013 | 2716 | 7.30E-03 | 291 | 46 |
| GO:BP | positive regulation of macromolecule biosynthetic process | GO:0010557 | 1682 | 7.40E-03 | 291 | 33 |
| TF | Factor: RORA; motif: CCYTGNCCTN; match class: 1 | TF:M12659_1 | 1963 | 7.40E-03 | 291 | 37 |
| TF | Factor: GATA-1; motif: NTGNNNNNNNNAGATAAGN | TF:M09936 | 3607 | 7.40E-03 | 291 | 56 |
| TF | Factor: ipf1; motif: CATTAR | TF:M01275 | 7333 | 7.50E-03 | 291 | 94 |
| TF | Factor: p53; motif: GGACATGCCCGGGCATGTCY | TF:M00034 | 3977 | 7.50E-03 | 291 | 60 |
| TF | Factor: FXR:RXR-ALPHA; motif: NAGKTCATTGACCYN; match class: 1 | TF:M08954_1 | 1884 | 7.50E-03 | 291 | 36 |
| MIRNA | hsa-miR-1238-3p | MIRNA:hsa-miR-1238-3p | 128 | 7.50E-03 | 291 | 8 |
| GO:MF | metal ion binding | GO:0046872 | 3968 | 7.60E-03 | 291 | 58 |
| GO:BP | cardiac chamber morphogenesis | GO:0003206 | 111 | 7.60E-03 | 291 | 8 |
| GO:BP | biomineral tissue development | GO:0031214 | 148 | 7.70E-03 | 291 | 9 |
| GO:BP | extrinsic apoptotic signaling pathway via death domain receptors | GO:0008625 | 79 | 7.70E-03 | 291 | 7 |
| GO:BP | regionalization | GO:0003002 | 280 | 7.80E-03 | 291 | 12 |
| GO:BP | positive regulation of cell activation | GO:0050867 | 330 | 7.80E-03 | 291 | 13 |
| TF | Factor: AP-2gamma; motif: NTGSCCTGRGGSNN; match class: 1 | TF:M09591_1 | 2550 | 7.80E-03 | 291 | 44 |
| TF | Factor: Sp2; motif: TGGGCGCGCCCA | TF:M06948 | 8390 | 7.80E-03 | 291 | 104 |
| GO:BP | response to reactive oxygen species | GO:0000302 | 189 | 7.90E-03 | 291 | 10 |
| TF | Factor: TFAP4; motif: AWCAGCTGWT | TF:M04192 | 4548 | 7.90E-03 | 291 | 66 |
| TF | Factor: A-Myb:Elf-1; motif: NMCCGGAACCGTTA | TF:M08390 | 10013 | 7.90E-03 | 291 | 119 |
| GO:MF | haptoglobin binding | GO:0031720 | 6 | 8.00E-03 | 291 | 3 |
| TF | Factor: SP1; motif: GGGGYGGGGNS; match class: 1 | TF:M01303_1 | 3433 | 8.00E-03 | 291 | 54 |
| TF | Factor: TCF-1; motif: NNNSWWCAAAGN | TF:M07433 | 4743 | 8.10E-03 | 291 | 68 |
| GO:BP | biomineralization | GO:0110148 | 149 | 8.20E-03 | 291 | 9 |
| TF | Factor: Sp2; motif: NYSGCCCCGCCCCCY | TF:M03567 | 7451 | 8.20E-03 | 291 | 95 |
| TF | Factor: HOXA13; motif: ATAAMA; match class: 1 | TF:M01292_1 | 6832 | 8.20E-03 | 291 | 89 |
| TF | Factor: HSF1; motif: GAANNTTCTNGN | TF:M07459 | 2902 | 8.20E-03 | 291 | 48 |
| TF | Factor: FPM315; motif: SRGGGAGGAGGN | TF:M01587 | 2990 | 8.20E-03 | 291 | 49 |
| TF | Factor: AP-3; motif: TKGAAAKN | TF:M04609 | 3528 | 8.30E-03 | 291 | 55 |
| TF | Factor: VDR:RXR-ALPHA; motif: NRGGTCANNNGGTTCNN; match class: 1 | TF:M08980_1 | 3529 | 8.30E-03 | 291 | 55 |
| GO:CC | vesicle membrane | GO:0012506 | 1138 | 8.50E-03 | 291 | 24 |
| GO:CC | specific granule | GO:0042581 | 145 | 8.50E-03 | 291 | 8 |
| TF | Factor: ZNF511; motif: GGRRGRGGCWGNG; match class: 1 | TF:M09738_1 | 4367 | 8.50E-03 | 291 | 64 |
| GO:BP | cardiac ventricle development | GO:0003231 | 113 | 8.60E-03 | 291 | 8 |
| TF | Factor: CTCF; motif: NNNNANASYGCCMYCTAGTGG | TF:M04689 | 4847 | 8.60E-03 | 291 | 69 |
| GO:BP | positive regulation of cell population proliferation | GO:0008284 | 813 | 8.80E-03 | 291 | 21 |
| TF | Factor: DEC1; motif: NGCACGTGAS | TF:M09876 | 7777 | 8.80E-03 | 291 | 98 |
| TF | Factor: Sox-15; motif: ACAAWGGG | TF:M03847 | 1353 | 8.90E-03 | 291 | 29 |
| TF | Factor: ER-beta; motif: GTCANASTGRCCYNR | TF:M01875 | 5536 | 8.90E-03 | 291 | 76 |
| GO:BP | regulation of leukocyte activation | GO:0002694 | 500 | 9.00E-03 | 291 | 16 |
| TF | Factor: IRF; motif: NNGAAANTGAAANN | TF:M08887 | 1063 | 9.00E-03 | 291 | 25 |
| TF | Factor: PPARgamma; motif: NWSTRGGKSARAGGKCA | TF:M10038 | 5836 | 9.10E-03 | 291 | 79 |
| TF | Factor: AP-1; motif: NNNTGAGTCAKCN | TF:M00517 | 4189 | 9.20E-03 | 291 | 62 |
| TF | Factor: STAT; motif: NNNNNTTCTKGGA | TF:M00777 | 4378 | 9.20E-03 | 291 | 64 |
| GO:BP | regulation of transport | GO:0051049 | 1622 | 9.40E-03 | 291 | 32 |
| REAC | SLC-mediated transmembrane transport | REAC:R-HSA-425407 | 229 | 9.40E-03 | 291 | 10 |
| TF | Factor: AR; motif: GGTACANNRTGTTCT | TF:M00481 | 4286 | 9.40E-03 | 291 | 63 |
| TF | Factor: CPBP; motif: NGGGCGG | TF:M05444 | 8104 | 9.40E-03 | 291 | 101 |
| TF | Factor: Sp6; motif: WGGGCGG | TF:M05361 | 8104 | 9.40E-03 | 291 | 101 |
| TF | Factor: Sp2; motif: WGGGCGG | TF:M05332 | 8104 | 9.40E-03 | 291 | 101 |
| TF | Factor: NR3C1; motif: RRGAACATWMYGTYCTRN | TF:M02219 | 5643 | 9.40E-03 | 291 | 77 |
| TF | Factor: TFAP2C; motif: NGCCTNAGGCN | TF:M04154 | 3363 | 9.50E-03 | 291 | 53 |
| TF | Factor: GLI2; motif: TGGGTGGTCNS | TF:M07291 | 2656 | 9.50E-03 | 291 | 45 |
| GO:BP | aorta morphogenesis | GO:0035909 | 31 | 9.70E-03 | 291 | 5 |
| TF | Factor: Tbx5; motif: NNAGGTGTNANN | TF:M01019 | 4960 | 9.70E-03 | 291 | 70 |
| TF | Factor: Msx-1; motif: CNGTAWNTG | TF:M00394 | 7584 | 9.70E-03 | 291 | 96 |
| TF | Factor: Oct3; motif: NNWTATGYWAATKANN | TF:M11935 | 2402 | 9.70E-03 | 291 | 42 |
| TF | Factor: NFATc1; motif: TTTTCCATGGAAAN; match class: 1 | TF:M04053_1 | 4962 | 9.80E-03 | 291 | 70 |
| GO:BP | negative regulation of peptidase activity | GO:0010466 | 194 | 9.90E-03 | 291 | 10 |
| GO:CC | membrane-enclosed lumen | GO:0031974 | 5613 | 9.90E-03 | 291 | 73 |
| GO:CC | intracellular organelle lumen | GO:0070013 | 5613 | 9.90E-03 | 291 | 73 |
| GO:CC | organelle lumen | GO:0043233 | 5613 | 9.90E-03 | 291 | 73 |
| GO:CC | external side of plasma membrane | GO:0009897 | 291 | 9.90E-03 | 291 | 11 |
| TF | Factor: c-Ets; motif: KRCAGGAARTRNKT | TF:M00340 | 4483 | 9.90E-03 | 291 | 65 |
| GO:BP | homotypic cell-cell adhesion | GO:0034109 | 82 | 1.00E-02 | 291 | 7 |
| GO:BP | tissue migration | GO:0090130 | 287 | 1.00E-02 | 291 | 12 |
| GO:BP | glomerular mesangial cell development | GO:0072144 | 5 | 1.00E-02 | 291 | 3 |
| TF | Factor: SNA; motif: NRCAGGTGCA | TF:M12258 | 2236 | 1.00E-02 | 291 | 40 |
| TF | Factor: B-ATF; motif: NNAYGACACN; match class: 1 | TF:M11301_1 | 1669 | 1.00E-02 | 291 | 33 |
| TF | Factor: TEF-3; motif: GNTATTTTT | TF:M07270 | 8541 | 1.00E-02 | 291 | 105 |
| TF | Factor: Oct-1; motif: NNNNATGCAAATNAN | TF:M00195 | 3014 | 1.00E-02 | 291 | 49 |
| TF | Factor: HFH2; motif: NNWAYRTAAACW | TF:M11555 | 3923 | 1.00E-02 | 291 | 59 |
| GO:MF | transition metal ion binding | GO:0046914 | 1017 | 1.10E-02 | 291 | 23 |
| GO:BP | nucleobase-containing compound biosynthetic process | GO:0034654 | 3527 | 1.10E-02 | 291 | 54 |
| GO:BP | cellular response to cadmium ion | GO:0071276 | 32 | 1.10E-02 | 291 | 5 |
| TF | Factor: Sp1; motif: GGGGCGGGGC | TF:M07063 | 6059 | 1.10E-02 | 291 | 81 |
| TF | Factor: AP-4; motif: AWCAGCTGWT; match class: 1 | TF:M11208_1 | 3285 | 1.10E-02 | 291 | 52 |
| TF | Factor: NFATc2; motif: TTTTCCA | TF:M02265 | 6464 | 1.10E-02 | 291 | 85 |
| TF | Factor: NFATc1; motif: NTTTCCRTNNAYGGAAAN | TF:M11981 | 5462 | 1.10E-02 | 291 | 75 |
| TF | Factor: TFAP2A; motif: NNNNGCCYSAGGGCA | TF:M07231 | 4780 | 1.10E-02 | 291 | 68 |
| TF | Factor: NKX2-5; motif: NNCACTCAANN | TF:M12495 | 1914 | 1.10E-02 | 291 | 36 |
| TF | Factor: HOXD4; motif: NYMATTAN | TF:M10712 | 3380 | 1.10E-02 | 291 | 53 |
| TF | Factor: SMAD; motif: AGACNBCNN | TF:M00792 | 3290 | 1.10E-02 | 291 | 52 |
| TF | Factor: ESE-1; motif: NWTRCGGAWGCN | TF:M11382 | 9421 | 1.10E-02 | 291 | 113 |
| TF | Factor: AR; motif: ARGAACANNNTGTNC; match class: 1 | TF:M07204_1 | 736 | 1.10E-02 | 291 | 20 |
| TF | Factor: gli3; motif: GACCACCCANG | TF:M01704 | 3113 | 1.10E-02 | 291 | 50 |
| TF | Factor: POU2F1:PEA3; motif: ACCGGATATGCAN | TF:M08402 | 6988 | 1.10E-02 | 291 | 90 |
| TF | Factor: TFEA; motif: RGTCACGTGA | TF:M10100 | 5277 | 1.10E-02 | 291 | 73 |
| TF | Factor: HOXD1; motif: NATYCAKCAN | TF:M12625 | 4694 | 1.10E-02 | 291 | 67 |
| TF | Factor: SMAD3; motif: YGTCTAGACA | TF:M04026 | 5872 | 1.10E-02 | 291 | 79 |
| GO:BP | mononuclear cell differentiation | GO:1903131 | 397 | 1.20E-02 | 291 | 14 |
| GO:BP | negative regulation of hydrolase activity | GO:0051346 | 293 | 1.20E-02 | 291 | 12 |
| TF | Factor: Pbx; motif: GATTGATKGNNS | TF:M00998 | 4313 | 1.20E-02 | 291 | 63 |
| TF | Factor: NF-kappaB2; motif: NGGGGAANYACCN | TF:M11997 | 328 | 1.20E-02 | 291 | 13 |
| TF | Factor: MLLT10; motif: CYNNCCNNGGNGCTG; match class: 1 | TF:M12636_1 | 3206 | 1.20E-02 | 291 | 51 |
| TF | Factor: Sp1; motif: GGGGCGGGGT; match class: 1 | TF:M00008_1 | 4794 | 1.20E-02 | 291 | 68 |
| TF | Factor: MIF-1; motif: NNGTTGCWWGGYAACNGS; match class: 1 | TF:M00279_1 | 4699 | 1.20E-02 | 291 | 67 |
| TF | Factor: NFATc1; motif: TTTCCAYWRTGGAAA; match class: 1 | TF:M04052_1 | 3299 | 1.20E-02 | 291 | 52 |
| TF | Factor: AREB6; motif: VNRCACCTGKNC | TF:M00414 | 1527 | 1.20E-02 | 291 | 31 |
| TF | Factor: FOSL1; motif: NNATGACTCATNN; match class: 1 | TF:M12518_1 | 1608 | 1.20E-02 | 291 | 32 |
| GO:BP | aorta development | GO:0035904 | 56 | 1.30E-02 | 291 | 6 |
| GO:BP | ion transmembrane transport | GO:0034220 | 1045 | 1.30E-02 | 291 | 24 |
| TF | Factor: ZNF775; motif: RGRGAGAAGN | TF:M12720 | 3038 | 1.30E-02 | 291 | 49 |
| TF | Factor: MYB; motif: NAACNGNCN | TF:M00913 | 4422 | 1.30E-02 | 291 | 64 |
| TF | Factor: COUP-TF1; motif: RRGGTCRNTGACCYY | TF:M11741 | 3398 | 1.30E-02 | 291 | 53 |
| TF | Factor: JunD; motif: NRTGASTCATN | TF:M07104 | 2177 | 1.30E-02 | 291 | 39 |
| TF | Factor: JUNB:C-JUN; motif: KRTGACGTCATN; match class: 1 | TF:M08945_1 | 4907 | 1.30E-02 | 291 | 69 |
| TF | Factor: CLOCK; motif: CNGNCACGTGNNNM | TF:M09884 | 7117 | 1.30E-02 | 291 | 91 |
| TF | Factor: IRF-4; motif: RGGAASWGR; match class: 1 | TF:M04818_1 | 2691 | 1.30E-02 | 291 | 45 |
| TF | Factor: AP-4; motif: YCAGCTGNKN; match class: 1 | TF:M10097_1 | 1307 | 1.30E-02 | 291 | 28 |
| TF | Factor: AP-4; motif: AWCAGCTGWT | TF:M11210 | 3956 | 1.30E-02 | 291 | 59 |
| TF | Factor: AP-4; motif: AWCAGCTGWT; match class: 1 | TF:M11210_1 | 3956 | 1.30E-02 | 291 | 59 |
| TF | Factor: IRF-4; motif: NCGAAACCGAAACYN | TF:M11687 | 3133 | 1.30E-02 | 291 | 50 |
| GO:MF | serine-type endopeptidase inhibitor activity | GO:0004867 | 67 | 1.40E-02 | 291 | 6 |
| GO:BP | negative regulation of nucleobase-containing compound metabolic process | GO:0045934 | 1341 | 1.40E-02 | 291 | 28 |
| GO:BP | adaptive immune response based on somatic recombination of immune receptors built from immunoglobulin superfamily domains | GO:0002460 | 247 | 1.40E-02 | 291 | 11 |
| GO:BP | nitrogen compound metabolic process | GO:0006807 | 9442 | 1.40E-02 | 291 | 112 |
| GO:BP | transmembrane receptor protein tyrosine kinase signaling pathway | GO:0007169 | 579 | 1.40E-02 | 291 | 17 |
| KEGG | NF-kappa B signaling pathway | KEGG:04064 | 99 | 1.40E-02 | 291 | 6 |
| TF | Factor: OVOL; motif: ANRTAACGG | TF:M08894 | 7120 | 1.40E-02 | 291 | 91 |
| TF | Factor: Isl2; motif: CTAATKR | TF:M02079 | 4718 | 1.40E-02 | 291 | 67 |
| TF | Factor: FOXO1A; motif: NNNYTGTTTNCN | TF:M09931 | 5699 | 1.40E-02 | 291 | 77 |
| TF | Factor: JunB; motif: GATGACGTCAYC; match class: 1 | TF:M11273_1 | 5600 | 1.40E-02 | 291 | 76 |
| TF | Factor: FPM315; motif: GGAGGAGGRRGRGGRGGRRGR | TF:M07141 | 2017 | 1.40E-02 | 291 | 37 |
| TF | Factor: ATF-3; motif: NATGATGTCATN | TF:M11290 | 5304 | 1.40E-02 | 291 | 73 |
| TF | Factor: LRF; motif: GGGGKYNNB; match class: 1 | TF:M01100_1 | 2609 | 1.40E-02 | 291 | 44 |
| TF | Factor: Oct-2; motif: NTGCATATGCAN | TF:M11899 | 4532 | 1.40E-02 | 291 | 65 |
| GO:MF | enzyme inhibitor activity | GO:0004857 | 327 | 1.50E-02 | 291 | 12 |
| GO:MF | inorganic anion transmembrane transporter activity | GO:0015103 | 138 | 1.50E-02 | 291 | 8 |
| GO:BP | sensory organ morphogenesis | GO:0090596 | 250 | 1.50E-02 | 291 | 11 |
| GO:BP | negative regulation of cellular biosynthetic process | GO:0031327 | 1427 | 1.50E-02 | 291 | 29 |
| REAC | Transport of bile salts and organic acids, metal ions and amine compounds | REAC:R-HSA-425366 | 75 | 1.50E-02 | 291 | 6 |
| TF | Factor: Oct3; motif: WTATGCGCATAW | TF:M11934 | 8394 | 1.50E-02 | 291 | 103 |
| TF | Factor: NF-kappaB1; motif: KGGRNTTTCCM | TF:M07221 | 2105 | 1.50E-02 | 291 | 38 |
| TF | Factor: Sox-9; motif: NNNNAACAATRGNN | TF:M00410 | 3323 | 1.50E-02 | 291 | 52 |
| TF | Factor: ZABC1; motif: NNTGNMCTTTGNNCYNNN | TF:M12683 | 3689 | 1.50E-02 | 291 | 56 |
| TF | Factor: TEF-3:C/EBPdelta; motif: RGWATGYNRTTRCGYAAY | TF:M08413 | 5118 | 1.50E-02 | 291 | 71 |
| TF | Factor: c-Ets-2; motif: NNCTTCCTNNN | TF:M07379 | 2966 | 1.50E-02 | 291 | 48 |
| TF | Factor: Oct-1; motif: NNNNNNNWATGCAAATNNNWNNA | TF:M00138 | 2024 | 1.50E-02 | 291 | 37 |
| TF | Factor: CEBPA; motif: NNATTGCACAATNN | TF:M12502 | 2530 | 1.50E-02 | 291 | 43 |
| TF | Factor: Fra-2; motif: TGASTCANCN | TF:M03870 | 3056 | 1.50E-02 | 291 | 49 |
| TF | Factor: oct-2; motif: NNWNATGCAAATNN | TF:M03836 | 3147 | 1.50E-02 | 291 | 50 |
| GO:BP | regulation of blood coagulation | GO:0030193 | 58 | 1.60E-02 | 291 | 6 |
| GO:BP | muscle tissue morphogenesis | GO:0060415 | 58 | 1.60E-02 | 291 | 6 |
| GO:BP | cellular component organization or biogenesis | GO:0071840 | 6398 | 1.60E-02 | 291 | 83 |
| GO:BP | lymphocyte differentiation | GO:0030098 | 353 | 1.60E-02 | 291 | 13 |
| GO:BP | anion transport | GO:0006820 | 465 | 1.60E-02 | 291 | 15 |
| TF | Factor: c-Fos; motif: NNTGASTCATN | TF:M07090 | 3604 | 1.60E-02 | 291 | 55 |
| TF | Factor: E2F-1:TBR2; motif: NGGTGNNANGGCGCNNTNNCRNNN | TF:M08523 | 11259 | 1.60E-02 | 291 | 129 |
| TF | Factor: JUND; motif: NNATGACTCATNN | TF:M12524 | 3514 | 1.60E-02 | 291 | 54 |
| TF | Factor: MAF; motif: NGCTGAGTCAN | TF:M00983 | 5128 | 1.60E-02 | 291 | 71 |
| TF | Factor: ctcf; motif: NNNCCASYAGRKGGCRSYNN | TF:M09602 | 6837 | 1.60E-02 | 291 | 88 |
| TF | Factor: NF-1A; motif: NGCCARN; match class: 1 | TF:M03554_1 | 626 | 1.60E-02 | 291 | 18 |
| TF | Factor: BTEB2; motif: GNAGGGGGNGGGSSNN | TF:M03814 | 4838 | 1.60E-02 | 291 | 68 |
| TF | Factor: VDR; motif: RRGGTCANNGRGKTCA; match class: 1 | TF:M09671_1 | 2116 | 1.60E-02 | 291 | 38 |
| GO:BP | regulation of endopeptidase activity | GO:0052548 | 354 | 1.70E-02 | 291 | 13 |
| GO:BP | positive regulation of cell adhesion | GO:0045785 | 409 | 1.70E-02 | 291 | 14 |
| GO:BP | heart valve development | GO:0003170 | 59 | 1.70E-02 | 291 | 6 |
| TF | Factor: Tbx3; motif: AGGTGTNR | TF:M08786 | 3157 | 1.70E-02 | 291 | 50 |
| TF | Factor: Tax/CREB; motif: RTGACGCATAYCCCC; match class: 1 | TF:M00115_1 | 2627 | 1.70E-02 | 291 | 44 |
| TF | Factor: c-Fos; motif: RTGASTCAY | TF:M04693 | 2202 | 1.70E-02 | 291 | 39 |
| TF | Factor: AhR:Arnt; motif: GRGKATYGCGTGMCWNSCC | TF:M00237 | 5832 | 1.70E-02 | 291 | 78 |
| TF | Factor: E2F-7; motif: GRGGCGGGAANNN | TF:M09896 | 9398 | 1.70E-02 | 291 | 112 |
| MIRNA | hsa-miR-670-3p | MIRNA:hsa-miR-670-3p | 186 | 1.70E-02 | 291 | 9 |
| GO:BP | positive regulation of cellular protein metabolic process | GO:0032270 | 1284 | 1.80E-02 | 291 | 27 |
| GO:BP | positive regulation of apoptotic process | GO:0043065 | 469 | 1.80E-02 | 291 | 15 |
| GO:BP | cellular zinc ion homeostasis | GO:0006882 | 35 | 1.80E-02 | 291 | 5 |
| GO:BP | middle ear morphogenesis | GO:0042474 | 17 | 1.80E-02 | 291 | 4 |
| GO:BP | kidney mesenchyme development | GO:0072074 | 17 | 1.80E-02 | 291 | 4 |
| GO:BP | negative regulation of cell migration | GO:0030336 | 255 | 1.80E-02 | 291 | 11 |
| GO:CC | platelet alpha granule | GO:0031091 | 82 | 1.80E-02 | 291 | 6 |
| TF | Factor: DATF1; motif: SNGGRRGCWGNGGG; match class: 1 | TF:M09724_1 | 2633 | 1.80E-02 | 291 | 44 |
| TF | Factor: AP-2alpha; motif: GCCNNNRGS | TF:M00469 | 4949 | 1.80E-02 | 291 | 69 |
| TF | Factor: p53; motif: RGRCWWGYCYNGRCWWGYYY; match class: 1 | TF:M01652_1 | 4757 | 1.80E-02 | 291 | 67 |
| TF | Factor: C-JUN:FOSB; motif: NATGACTCAY | TF:M08936 | 2723 | 1.80E-02 | 291 | 45 |
| TF | Factor: Sox-3; motif: SNNACAATRK | TF:M11617 | 3258 | 1.80E-02 | 291 | 51 |
| CORUM | Calprotectin heterotetramer | CORUM:6826 | 2 | 1.80E-02 | 291 | 2 |
| CORUM | NOS3-CAV1 complex | CORUM:5714 | 2 | 1.80E-02 | 291 | 2 |
| GO:BP | mononuclear cell migration | GO:0071674 | 165 | 1.90E-02 | 291 | 9 |
| GO:BP | regulation of hemostasis | GO:1900046 | 60 | 1.90E-02 | 291 | 6 |
| GO:BP | heterocycle biosynthetic process | GO:0018130 | 3595 | 1.90E-02 | 291 | 54 |
| GO:BP | regulation of tube diameter | GO:0035296 | 126 | 1.90E-02 | 291 | 8 |
| GO:BP | blood vessel diameter maintenance | GO:0097746 | 126 | 1.90E-02 | 291 | 8 |
| GO:CC | membrane raft | GO:0045121 | 312 | 1.90E-02 | 291 | 11 |
| GO:CC | membrane microdomain | GO:0098857 | 313 | 1.90E-02 | 291 | 11 |
| KEGG | Focal adhesion | KEGG:04510 | 195 | 1.90E-02 | 291 | 8 |
| REAC | Metallothioneins bind metals | REAC:R-HSA-5661231 | 9 | 1.90E-02 | 291 | 3 |
| TF | Factor: alpha-CP1; motif: CAGCCAATGAG | TF:M00687 | 2044 | 1.90E-02 | 291 | 37 |
| TF | Factor: YY1; motif: NGCCATYTTKGRCNWWNNGTGCK | TF:M12276 | 8868 | 1.90E-02 | 291 | 107 |
| TF | Factor: NF-1C; motif: NTTGGCNNNNTGCCARN; match class: 1 | TF:M11728_1 | 967 | 1.90E-02 | 291 | 23 |
| TF | Factor: C/EBPbeta; motif: RNRTKDNGMAAKNN | TF:M00109 | 2642 | 1.90E-02 | 291 | 44 |
| TF | Factor: Oct3; motif: NNTTATGYWAATKARN | TF:M11932 | 2470 | 1.90E-02 | 291 | 42 |
| TF | Factor: GATA-1; motif: NNCWGATARNNNN | TF:M00128 | 2048 | 1.90E-02 | 291 | 37 |
| TF | Factor: PBX3; motif: TGANTGRCRGS | TF:M10030 | 1884 | 1.90E-02 | 291 | 35 |
| GO:BP | regulation of lymphocyte activation | GO:0051249 | 415 | 2.00E-02 | 291 | 14 |
| GO:BP | negative regulation of extrinsic apoptotic signaling pathway | GO:2001237 | 91 | 2.00E-02 | 291 | 7 |
| GO:BP | autocrine signaling | GO:0035425 | 6 | 2.00E-02 | 291 | 3 |
| GO:BP | glomerular mesangial cell differentiation | GO:0072008 | 6 | 2.00E-02 | 291 | 3 |
| GO:BP | regulation of tube size | GO:0035150 | 127 | 2.00E-02 | 291 | 8 |
| GO:CC | side of membrane | GO:0098552 | 492 | 2.00E-02 | 291 | 14 |
| GO:CC | collagen type I trimer | GO:0005584 | 2 | 2.00E-02 | 291 | 2 |
| GO:CC | interleukin-18 receptor complex | GO:0045092 | 2 | 2.00E-02 | 291 | 2 |
| TF | Factor: Kaiso; motif: NTCCTGCNAN | TF:M01119 | 3633 | 2.00E-02 | 291 | 55 |
| TF | Factor: PPARGAMMA:RXR-ALPHA; motif: NNRGGTCAAWAGGTCAN | TF:M08962 | 4100 | 2.00E-02 | 291 | 60 |
| TF | Factor: SNA; motif: GCCACCTGNCNGYN | TF:M03566 | 1969 | 2.00E-02 | 291 | 36 |
| TF | Factor: PMX1; motif: TAATHA; match class: 1 | TF:M03560_1 | 8561 | 2.00E-02 | 291 | 104 |
| TF | Factor: MAZ; motif: GGGMGGGGS; match class: 1 | TF:M10432_1 | 4198 | 2.00E-02 | 291 | 61 |
| TF | Factor: ESR1; motif: NNNNMAGGTCACCCTGACCY; match class: 1 | TF:M02261_1 | 2053 | 2.00E-02 | 291 | 37 |
| GO:BP | aromatic compound biosynthetic process | GO:0019438 | 3605 | 2.10E-02 | 291 | 54 |
| GO:BP | response to progesterone | GO:0032570 | 36 | 2.10E-02 | 291 | 5 |
| GO:BP | semi-lunar valve development | GO:1905314 | 36 | 2.10E-02 | 291 | 5 |
| GO:BP | protein metabolic process | GO:0019538 | 4947 | 2.10E-02 | 291 | 68 |
| TF | Factor: p73; motif: NNRCAWGYCCARRCWTGYC | TF:M10025 | 5463 | 2.10E-02 | 291 | 74 |
| TF | Factor: beta-catenin; motif: CTTTGATN; match class: 1 | TF:M07599_1 | 1809 | 2.10E-02 | 291 | 34 |
| TF | Factor: ZNF394; motif: NRARWRGAANNNAMWGNAAK; match class: 1 | TF:M10147_1 | 1493 | 2.10E-02 | 291 | 30 |
| TF | Factor: TFAP2A; motif: NGCCYSAGGCN | TF:M04147 | 4684 | 2.10E-02 | 291 | 66 |
| GO:BP | cellular response to lipopolysaccharide | GO:0071222 | 168 | 2.20E-02 | 291 | 9 |
| GO:BP | negative regulation of biosynthetic process | GO:0009890 | 1454 | 2.20E-02 | 291 | 29 |
| GO:BP | positive regulation of nucleic acid-templated transcription | GO:1903508 | 1456 | 2.20E-02 | 291 | 29 |
| GO:BP | positive regulation of transcription, DNA-templated | GO:0045893 | 1456 | 2.20E-02 | 291 | 29 |
| TF | Factor: C/EBPbeta; motif: NRTTRYGCAATN | TF:M09881 | 2226 | 2.20E-02 | 291 | 39 |
| TF | Factor: AP4; motif: NMNCAGCTGGN; match class: 1 | TF:M12580_1 | 1119 | 2.20E-02 | 291 | 25 |
| TF | Factor: SOX10; motif: NNAACAAAGNN; match class: 1 | TF:M09819_1 | 906 | 2.20E-02 | 291 | 22 |
| TF | Factor: AR; motif: NNNGNRRGNACANNGTGTTCTNNNNNN; match class: 1 | TF:M00953_1 | 159 | 2.20E-02 | 291 | 9 |
| TF | Factor: WT1; motif: SMCNCCNSC | TF:M01118 | 6380 | 2.20E-02 | 291 | 83 |
| TF | Factor: SF1; motif: NTGRCCTTGAMCT | TF:M12661 | 1979 | 2.20E-02 | 291 | 36 |
| GO:BP | kidney epithelium development | GO:0072073 | 129 | 2.30E-02 | 291 | 8 |
| GO:BP | positive regulation of RNA biosynthetic process | GO:1902680 | 1458 | 2.30E-02 | 291 | 29 |
| TF | Factor: E2F1; motif: GSGCGGGAAN; match class: 1 | TF:M12597_1 | 9447 | 2.30E-02 | 291 | 112 |
| TF | Factor: KLF15; motif: GAGNNGGGGNGTDG | TF:M01714 | 1981 | 2.30E-02 | 291 | 36 |
| TF | Factor: STAT5A; motif: NAWTTCYNGGAANYN | TF:M00457 | 3468 | 2.30E-02 | 291 | 53 |
| TF | Factor: DEC1; motif: CNCACRTGASC | TF:M03572 | 5982 | 2.30E-02 | 291 | 79 |
| TF | Factor: ER-alpha; motif: ARGSTGACC | TF:M04881 | 3378 | 2.30E-02 | 291 | 52 |
| TF | Factor: NKX2B; motif: CACTTNA | TF:M02109 | 8588 | 2.30E-02 | 291 | 104 |
| GO:BP | zinc ion homeostasis | GO:0055069 | 37 | 2.40E-02 | 291 | 5 |
| GO:BP | response to purine-containing compound | GO:0014074 | 130 | 2.40E-02 | 291 | 8 |
| GO:BP | regulation of hydrolase activity | GO:0051336 | 870 | 2.40E-02 | 291 | 21 |
| GO:CC | specific granule membrane | GO:0035579 | 86 | 2.40E-02 | 291 | 6 |
| KEGG | Bladder cancer | KEGG:05219 | 39 | 2.40E-02 | 291 | 4 |
| TF | Factor: CEBPA; motif: ATTGCAYAAYN | TF:M07205 | 2066 | 2.40E-02 | 291 | 37 |
| TF | Factor: HIF-1alpha; motif: NCACGT | TF:M02012 | 5985 | 2.40E-02 | 291 | 79 |
| TF | Factor: ZBTB37; motif: TGGCTTTGGY | TF:M12688 | 3380 | 2.40E-02 | 291 | 52 |
| TF | Factor: Six-2; motif: NNGTATCRNN | TF:M11030 | 5986 | 2.40E-02 | 291 | 79 |
| TF | Factor: NR1B1; motif: NRGGTCANRRGGTCAN | TF:M11792 | 4126 | 2.40E-02 | 291 | 60 |
| TF | Factor: Pax-5; motif: RRNGRNGCAN; match class: 1 | TF:M03577_1 | 2236 | 2.40E-02 | 291 | 39 |
| GO:BP | regulation of phosphorylation | GO:0042325 | 1159 | 2.50E-02 | 291 | 25 |
| GO:BP | regulation of coagulation | GO:0050818 | 63 | 2.50E-02 | 291 | 6 |
| KEGG | Human papillomavirus infection | KEGG:05165 | 314 | 2.50E-02 | 291 | 10 |
| TF | Factor: NFATc1; motif: NATGGAAANWNANTTTYCMN | TF:M04051 | 4704 | 2.50E-02 | 291 | 66 |
| TF | Factor: NFATc2; motif: NTTTCCATNNATGGAAAN | TF:M11986 | 5194 | 2.50E-02 | 291 | 71 |
| TF | Factor: GR; motif: RGRACATTNTGTYC; match class: 1 | TF:M04750_1 | 1428 | 2.50E-02 | 291 | 29 |
| TF | Factor: POU3F2; motif: WTATGCWAATKAG | TF:M11904 | 2582 | 2.50E-02 | 291 | 43 |
| TF | Factor: DPRX; motif: NNGGATTANN | TF:M04306 | 4131 | 2.50E-02 | 291 | 60 |
| TF | Factor: POU4F3; motif: CTAATYW | TF:M07061 | 5993 | 2.50E-02 | 291 | 79 |
| TF | Factor: p63; motif: NNRCAWGYCTGGRCWTGYN | TF:M09643 | 3386 | 2.50E-02 | 291 | 52 |
| TF | Factor: Fli-1:ETV7; motif: NSMGGAMGGATNTCCGNN | TF:M08283 | 2670 | 2.50E-02 | 291 | 44 |
| TF | Factor: ER71:SREBP-2; motif: NTSACGTGACGGAARY | TF:M08457 | 10905 | 2.50E-02 | 291 | 125 |
| TF | Factor: TTF-1; motif: NNNCNCTTGASNNN | TF:M02034 | 3206 | 2.50E-02 | 291 | 50 |
| GO:BP | negative regulation of ion transport | GO:0043271 | 131 | 2.60E-02 | 291 | 8 |
| GO:BP | negative regulation of intrinsic apoptotic signaling pathway | GO:2001243 | 95 | 2.60E-02 | 291 | 7 |
| TF | Factor: SREBP; motif: NNNNYCACNCCANNN | TF:M01168 | 5699 | 2.60E-02 | 291 | 76 |
| TF | Factor: ATF2; motif: NTGACRTCAYC | TF:M12581 | 3762 | 2.60E-02 | 291 | 56 |
| TF | Factor: Fli-1:C/EBPbeta; motif: RNCGGAWRTTGCGCAAY | TF:M08281 | 6613 | 2.60E-02 | 291 | 85 |
| TF | Factor: DEC; motif: SCCCAMGTGAAGN | TF:M00997 | 7759 | 2.60E-02 | 291 | 96 |
| TF | Factor: Gli2; motif: NTGGGTGGTCNN | TF:M07455 | 2502 | 2.60E-02 | 291 | 42 |
| GO:BP | regulation of actin filament-based movement | GO:1903115 | 38 | 2.70E-02 | 291 | 5 |
| GO:BP | positive regulation of molecular function | GO:0044093 | 1394 | 2.70E-02 | 291 | 28 |
| GO:CC | ficolin-1-rich granule membrane | GO:0101003 | 55 | 2.70E-02 | 291 | 5 |
| TF | Factor: POU2F2; motif: NTATGCWAATN | TF:M04072 | 1912 | 2.70E-02 | 291 | 35 |
| TF | Factor: c-Jun; motif: NNNNRRTGASTCAN | TF:M07102 | 4236 | 2.70E-02 | 291 | 61 |
| TF | Factor: TATA; motif: STATAAAWRNNNNNN | TF:M00252 | 3672 | 2.70E-02 | 291 | 55 |
| TF | Factor: DB1; motif: GGRRRRGRRGGAGGGGGNGRRR; match class: 1 | TF:M10107_1 | 2504 | 2.70E-02 | 291 | 42 |
| TF | Factor: beta-catenin; motif: CTTTGATN | TF:M07599 | 6721 | 2.70E-02 | 291 | 86 |
| TF | Factor: p73; motif: NGRCATGYYTGNRCWTGYYN | TF:M09644 | 3034 | 2.70E-02 | 291 | 48 |
| GO:BP | muscle organ morphogenesis | GO:0048644 | 64 | 2.80E-02 | 291 | 6 |
| GO:BP | negative regulation of ion transmembrane transporter activity | GO:0032413 | 64 | 2.80E-02 | 291 | 6 |
| GO:BP | negative regulation of cell motility | GO:2000146 | 267 | 2.80E-02 | 291 | 11 |
| TF | Factor: GABP-alpha; motif: AACCGGAAR; match class: 1 | TF:M04748_1 | 9377 | 2.80E-02 | 291 | 111 |
| TF | Factor: Sp1; motif: NNGGGGCGGGGNN; match class: 1 | TF:M00932_1 | 5909 | 2.80E-02 | 291 | 78 |
| TF | Factor: C/EBPepsilon; motif: HAANMTTKCNWMAC | TF:M01868 | 2507 | 2.80E-02 | 291 | 42 |
| TF | Factor: Sox-18; motif: NACAAYGS | TF:M11623 | 4529 | 2.80E-02 | 291 | 64 |
| TF | Factor: ATF3; motif: GRTGACKCA | TF:M12582 | 6319 | 2.80E-02 | 291 | 82 |
| GO:MF | SMAD binding | GO:0046332 | 76 | 2.90E-02 | 291 | 6 |
| GO:BP | positive regulation of leukocyte activation | GO:0002696 | 319 | 2.90E-02 | 291 | 12 |
| TF | Factor: GATA-2; motif: NTGNNNNNNNNAGATAAGN | TF:M09937 | 2338 | 2.90E-02 | 291 | 40 |
| TF | Factor: ESR1; motif: STGACCTN | TF:M12602 | 2862 | 2.90E-02 | 291 | 46 |
| TF | Factor: NFATc2; motif: NTTTCCGCGGAAAN | TF:M11983 | 7146 | 2.90E-02 | 291 | 90 |
| TF | Factor: E2A; motif: NRMCASCTGCNNN | TF:M02088 | 2511 | 2.90E-02 | 291 | 42 |
| TF | Factor: Helios; motif: NNTWGGGANNN | TF:M01003 | 3131 | 2.90E-02 | 291 | 49 |
| TF | Factor: Dlx-7; motif: NTCRTTAN | TF:M10600 | 6220 | 2.90E-02 | 291 | 81 |
| TF | Factor: RFX1; motif: NNGTNRCNWRGYAACNN | TF:M00280 | 5716 | 2.90E-02 | 291 | 76 |
| TF | Factor: LRH-1; motif: TCAAGGTCRTGACCTTGA | TF:M11831 | 3042 | 2.90E-02 | 291 | 48 |
| TF | Factor: Prep-1; motif: GRBTGANAGATN | TF:M07464 | 3683 | 2.90E-02 | 291 | 55 |
| TF | Factor: TEAD3; motif: RCATTCCW | TF:M04144 | 994 | 2.90E-02 | 291 | 23 |
| TF | Factor: Oct-2; motif: NTGCATATGCAN; match class: 1 | TF:M11899_1 | 3591 | 2.90E-02 | 291 | 54 |
| TF | Factor: Msx-1; motif: WNGNAATTANV | TF:M08822 | 5618 | 2.90E-02 | 291 | 75 |
| GO:BP | leukocyte mediated immunity | GO:0002443 | 320 | 3.00E-02 | 291 | 12 |
| TF | Factor: T-box; motif: NASGTGTNAN | TF:M12021 | 3685 | 3.00E-02 | 291 | 55 |
| TF | Factor: JUNB:FRA-2; motif: NRTGASTCAT | TF:M08923 | 2428 | 3.00E-02 | 291 | 41 |
| TF | Factor: STAT1; motif: TTCNRGGAAN | TF:M07064 | 2258 | 3.00E-02 | 291 | 39 |
| GO:BP | negative regulation of cation channel activity | GO:2001258 | 39 | 3.10E-02 | 291 | 5 |
| GO:BP | programmed necrotic cell death | GO:0097300 | 39 | 3.10E-02 | 291 | 5 |
| GO:BP | cellular response to molecule of bacterial origin | GO:0071219 | 176 | 3.10E-02 | 291 | 9 |
| TF | Factor: GLI; motif: MCVNNGACCACCCAV | TF:M03871 | 3048 | 3.10E-02 | 291 | 48 |
| TF | Factor: LRF; motif: RCGACCACCNN; match class: 1 | TF:M12230_1 | 2432 | 3.10E-02 | 291 | 41 |
| TF | Factor: ERR1; motif: NTGACCTTGRNN | TF:M04873 | 2694 | 3.10E-02 | 291 | 44 |
| TF | Factor: Oct3; motif: NATGCAANNN | TF:M01307 | 3322 | 3.10E-02 | 291 | 51 |
| GO:CC | apical part of cell | GO:0045177 | 391 | 3.20E-02 | 291 | 12 |
| REAC | Signaling by PDGF | REAC:R-HSA-186797 | 53 | 3.20E-02 | 291 | 5 |
| TF | Factor: RARA; motif: AGGTCAANNARAGGTCA | TF:M04483 | 3600 | 3.20E-02 | 291 | 54 |
| TF | Factor: C/EBPgamma; motif: NRTKRSRMAAKN | TF:M01869 | 2608 | 3.20E-02 | 291 | 43 |
| TF | Factor: AP-1; motif: RGTGACTMANN | TF:M00188 | 2609 | 3.20E-02 | 291 | 43 |
| TF | Factor: MR; motif: NGNACRNNNYGTNCN | TF:M11849 | 3788 | 3.20E-02 | 291 | 56 |
| GO:MF | RAGE receptor binding | GO:0050786 | 9 | 3.30E-02 | 291 | 3 |
| TF | Factor: foxm1; motif: TGTTTRCTYWNN | TF:M09930 | 5734 | 3.30E-02 | 291 | 76 |
| TF | Factor: RAR-gamma; motif: TGACCTBYNKN | TF:M03562 | 3697 | 3.30E-02 | 291 | 55 |
| TF | Factor: HES-1; motif: GNCACGTGNC; match class: 1 | TF:M08767_1 | 6962 | 3.30E-02 | 291 | 88 |
| TF | Factor: RUNX2; motif: WRACCGCANWAACCGCAN; match class: 1 | TF:M04107_1 | 6039 | 3.30E-02 | 291 | 79 |
| TF | Factor: STAT6; motif: NNYTTCCY | TF:M00500 | 4076 | 3.30E-02 | 291 | 59 |
| TF | Factor: HAIRYLIKE; motif: NNNNCANGTG | TF:M08885 | 5339 | 3.40E-02 | 291 | 72 |
| TF | Factor: Brachyury; motif: NTNNCANNNNRGTGTGAANN | TF:M09878 | 3240 | 3.40E-02 | 291 | 50 |
| TF | Factor: Fra-1; motif: NATGASTCAYM | TF:M11284 | 2615 | 3.40E-02 | 291 | 43 |
| TF | Factor: NF-1C; motif: NTTGGCNNNNTGCCARN; match class: 1 | TF:M11729_1 | 794 | 3.40E-02 | 291 | 20 |
| TF | Factor: Fra-2; motif: NNRTGAGTCAYN | TF:M09923 | 3611 | 3.40E-02 | 291 | 54 |
| GO:BP | response to oxygen levels | GO:0070482 | 325 | 3.50E-02 | 291 | 12 |
| TF | Factor: HOXB5; motif: RTCRTTAN | TF:M10698 | 3800 | 3.50E-02 | 291 | 56 |
| TF | Factor: Dlx-3; motif: NTCRTTAN | TF:M10609 | 3800 | 3.50E-02 | 291 | 56 |
| TF | Factor: E2F-4; motif: NGGCGGGAARN | TF:M07084 | 10196 | 3.50E-02 | 291 | 118 |
| GO:BP | growth | GO:0040007 | 893 | 3.60E-02 | 291 | 21 |
| GO:BP | pulmonary valve development | GO:0003177 | 20 | 3.60E-02 | 291 | 4 |
| REAC | Biological oxidations | REAC:R-HSA-211859 | 169 | 3.60E-02 | 291 | 8 |
| REAC | Dissolution of Fibrin Clot | REAC:R-HSA-75205 | 11 | 3.60E-02 | 291 | 3 |
| TF | Factor: NFATc2; motif: TTTTCCATGGAAAA; match class: 1 | TF:M11985_1 | 3803 | 3.60E-02 | 291 | 56 |
| TF | Factor: C/EBPalpha; motif: ATTGCGYAAY | TF:M07412 | 2888 | 3.60E-02 | 291 | 46 |
| GO:BP | cellular nitrogen compound biosynthetic process | GO:0044271 | 4249 | 3.70E-02 | 291 | 60 |
| GO:BP | negative regulation of cellular component movement | GO:0051271 | 275 | 3.70E-02 | 291 | 11 |
| TF | Factor: ZNF342; motif: NRGTGGWCANT | TF:M12076 | 1229 | 3.70E-02 | 291 | 26 |
| TF | Factor: Fra-1; motif: NNNTGAGTCAYN | TF:M09922 | 3807 | 3.70E-02 | 291 | 56 |
| TF | Factor: T3R-beta; motif: NNRGGTCRTGACCYNN | TF:M11815 | 4668 | 3.70E-02 | 291 | 65 |
| GO:BP | in utero embryonic development | GO:0001701 | 383 | 3.80E-02 | 291 | 13 |
| GO:BP | response to metal ion | GO:0010038 | 328 | 3.80E-02 | 291 | 12 |
| GO:CC | plasma membrane region | GO:0098590 | 1176 | 3.80E-02 | 291 | 23 |
| TF | Factor: DPF2; motif: NYCACYTCCYCNYYCY; match class: 1 | TF:M09760_1 | 2803 | 3.80E-02 | 291 | 45 |
| TF | Factor: GCMa:Erg; motif: ATGCGGGCGGAARKG; match class: 1 | TF:M08487_1 | 8249 | 3.80E-02 | 291 | 100 |
| TF | Factor: POU5F1; motif: NNATGCAAANN | TF:M09782 | 2982 | 3.80E-02 | 291 | 47 |
| TF | Factor: PDEF; motif: MCCGGATNTN | TF:M02075 | 2453 | 3.80E-02 | 291 | 41 |
| TF | Factor: SPI1; motif: NGRGGAAGTN | TF:M02078 | 3073 | 3.80E-02 | 291 | 48 |
| TF | Factor: MSANTD3; motif: SNNCACTCAC | TF:M12406 | 3999 | 3.80E-02 | 291 | 58 |
| TF | Factor: FOXO1A:Net; motif: RWMAACAGGAAGTN; match class: 1 | TF:M08302_1 | 2282 | 3.80E-02 | 291 | 39 |
| GO:BP | nephron epithelium development | GO:0072009 | 101 | 3.90E-02 | 291 | 7 |
| TF | Factor: DMBX1; motif: NNGGATTANN | TF:M04305 | 4383 | 3.90E-02 | 291 | 62 |
| TF | Factor: BRCA2; motif: TGMCYCWGN | TF:M12723 | 3259 | 3.90E-02 | 291 | 50 |
| GO:BP | negative regulation of blood coagulation | GO:0030195 | 41 | 4.00E-02 | 291 | 5 |
| TF | Factor: IRF; motif: RAAANTGAAAN | TF:M00972 | 1623 | 4.00E-02 | 291 | 31 |
| TF | Factor: AML2; motif: CCRCACCAYDN | TF:M01854 | 2372 | 4.00E-02 | 291 | 40 |
| TF | Factor: CDP:HOXA13; motif: ATCGATYAYSYCRTAAA | TF:M08201 | 6582 | 4.00E-02 | 291 | 84 |
| GO:CC | basement membrane | GO:0005604 | 95 | 4.10E-02 | 291 | 6 |
| TF | Factor: REST; motif: NNNNGGNGCTGTCCATGGTGCT | TF:M01256 | 2374 | 4.10E-02 | 291 | 40 |
| TF | Factor: ER-alpha; motif: NAGGTCACSGTGACCTN | TF:M11843 | 3172 | 4.10E-02 | 291 | 49 |
| TF | Factor: HOXA6; motif: NYMATTAN | TF:M08772 | 6482 | 4.10E-02 | 291 | 83 |
| TF | Factor: TEF-1; motif: NRCATWCCN; match class: 1 | TF:M12042_1 | 672 | 4.20E-02 | 291 | 18 |
| TF | Factor: Cdx-2; motif: NRTYRTAAAN | TF:M10851 | 1953 | 4.20E-02 | 291 | 35 |
| TF | Factor: STAT3; motif: NGNNATTTCCSGGAARTGNNN | TF:M00225 | 2815 | 4.20E-02 | 291 | 45 |
| TF | Factor: E2A; motif: CAGNTGNN | TF:M07353 | 2292 | 4.20E-02 | 291 | 39 |
| GO:BP | defense response to bacterium | GO:0042742 | 183 | 4.30E-02 | 291 | 9 |
| TF | Factor: MR; motif: NGNACRNNNYGTNCN; match class: 1 | TF:M11849_1 | 3732 | 4.30E-02 | 291 | 55 |
| TF | Factor: Sox-4; motif: BCWTTGT | TF:M07268 | 4591 | 4.30E-02 | 291 | 64 |
| TF | Factor: E2F-4; motif: NNTTCCCGCCNN; match class: 1 | TF:M04823_1 | 7533 | 4.30E-02 | 291 | 93 |
| GO:BP | negative regulation of proteolysis | GO:0045861 | 280 | 4.40E-02 | 291 | 11 |
| GO:BP | entry into host | GO:0044409 | 141 | 4.40E-02 | 291 | 8 |
| GO:BP | cell development | GO:0048468 | 1919 | 4.40E-02 | 291 | 34 |
| REAC | MET activates PTK2 signaling | REAC:R-HSA-8874081 | 30 | 4.40E-02 | 291 | 4 |
| TF | Factor: FOXJ2; motif: NNTGTTGTAAAYAN | TF:M11584 | 3923 | 4.40E-02 | 291 | 57 |
| TF | Factor: REST; motif: CCNNGGTGCTGAA | TF:M03883 | 3090 | 4.40E-02 | 291 | 48 |
| TF | Factor: Spic; motif: NGNGGAASTN; match class: 1 | TF:M02077_1 | 2042 | 4.40E-02 | 291 | 36 |
| MIRNA | hsa-miR-204-5p | MIRNA:hsa-miR-204-5p | 372 | 4.40E-02 | 291 | 12 |
| GO:BP | response to interferon-alpha | GO:0035455 | 21 | 4.50E-02 | 291 | 4 |
| GO:BP | negative regulation of hemostasis | GO:1900047 | 42 | 4.50E-02 | 291 | 5 |
| GO:BP | anion transmembrane transport | GO:0098656 | 231 | 4.50E-02 | 291 | 10 |
| GO:CC | aggresome | GO:0016235 | 33 | 4.50E-02 | 291 | 4 |
| TF | Factor: AP-2alpha; motif: NTNSCCTGRGGSNAN | TF:M09590 | 4694 | 4.50E-02 | 291 | 65 |
| TF | Factor: NF-E4; motif: CHCCCTCKCCWG | TF:M02105 | 2471 | 4.50E-02 | 291 | 41 |
| TF | Factor: Hey2; motif: NNCACGYGNN; match class: 1 | TF:M11052_1 | 8713 | 4.50E-02 | 291 | 104 |
| TF | Factor: Nkx3-2; motif: TRAGTG | TF:M01181 | 8070 | 4.50E-02 | 291 | 98 |
| TF | Factor: NFATc2; motif: NTTTCCATNNATGGAAAN; match class: 1 | TF:M11986_1 | 4990 | 4.50E-02 | 291 | 68 |
| GO:BP | positive regulation of response to external stimulus | GO:0032103 | 390 | 4.60E-02 | 291 | 13 |
| TF | Factor: B-ATF; motif: NNAYGACACN | TF:M11301 | 6607 | 4.60E-02 | 291 | 84 |
| TF | Factor: isx; motif: NTCRTTAA | TF:M10956 | 8396 | 4.60E-02 | 291 | 101 |
| GO:MF | oxygen carrier activity | GO:0005344 | 10 | 4.70E-02 | 291 | 3 |
| GO:BP | negative regulation of transcription by RNA polymerase II | GO:0000122 | 841 | 4.80E-02 | 291 | 20 |
| REAC | Response to metal ions | REAC:R-HSA-5660526 | 12 | 4.80E-02 | 291 | 3 |
| TF | Factor: ERF:C/EBPdelta; motif: NNCGGAWRTTGCGCAAY | TF:M08233 | 4997 | 4.80E-02 | 291 | 68 |
| TF | Factor: myogenin; motif: CRSCTGTTBNNTTTGGCACGSNGCCARCH; match class: 1 | TF:M00056_1 | 3842 | 4.80E-02 | 291 | 56 |
| TF | Factor: Sox-9; motif: MACARWGNNNYCNTTNW; match class: 1 | TF:M08838_1 | 552 | 4.80E-02 | 291 | 16 |
| GO:BP | response to corticosteroid | GO:0031960 | 143 | 4.90E-02 | 291 | 8 |
| GO:BP | lymphocyte mediated immunity | GO:0002449 | 233 | 4.90E-02 | 291 | 10 |
| TF | Factor: E2F-4; motif: AATGGCGCCAAA | TF:M04518 | 4902 | 4.90E-02 | 291 | 67 |
| TF | Factor: BEN; motif: CWGCGAYA | TF:M01241 | 6926 | 4.90E-02 | 291 | 87 |
| TF | Factor: HSF1; motif: RGAANRTTCYRGAAN | TF:M11656 | 1721 | 4.90E-02 | 291 | 32 |
| GO:MF | interleukin-18 receptor activity | GO:0042008 | 2 | 5.00E-02 | 291 | 2 |
| GO:MF | heme binding | GO:0020037 | 121 | 5.00E-02 | 291 | 7 |

**Supplementary Table 5: KCL BrainBank enrichment for Cluster 2.** GO:BP – Gene Ontology Biological Process, GO:CC – Gene Ontology Cellular Components, GO:MF – Gene Ontology Molecular Function, KEGG: Kyoto Encyclopaedia of Genes and Genomes, REAC: Reactome, TF: Transfac. MIRNA: miRTarBase CORUM: CORUM database of protein complexes.

| **Source** | **Term Name** | **Term ID** | **Term Size** | **p-value adjusted** | **Query Size** | **Intersection Size** |
| --- | --- | --- | --- | --- | --- | --- |
| GO:BP | immune response | GO:0006955 | 1363 | 6.00E-58 | 372 | 106 |
| GO:BP | immune system process | GO:0002376 | 2161 | 5.50E-57 | 372 | 127 |
| GO:BP | regulation of immune system process | GO:0002682 | 1217 | 2.60E-47 | 372 | 91 |
| GO:CC | cell periphery | GO:0071944 | 5188 | 2.20E-46 | 372 | 173 |
| GO:BP | defense response | GO:0006952 | 1343 | 7.40E-42 | 372 | 89 |
| GO:CC | plasma membrane | GO:0005886 | 4761 | 7.20E-41 | 372 | 158 |
| REAC | Immune System | REAC:R-HSA-168256 | 1770 | 1.60E-40 | 372 | 98 |
| GO:BP | regulation of immune response | GO:0050776 | 741 | 8.30E-40 | 372 | 68 |
| GO:CC | integral component of membrane | GO:0016021 | 4917 | 4.20E-37 | 372 | 155 |
| GO:BP | leukocyte activation | GO:0045321 | 795 | 8.10E-37 | 372 | 67 |
| GO:CC | intrinsic component of membrane | GO:0031224 | 5051 | 2.50E-36 | 372 | 156 |
| GO:BP | cell activation | GO:0001775 | 914 | 5.80E-35 | 372 | 69 |
| GO:BP | positive regulation of immune system process | GO:0002684 | 764 | 7.70E-35 | 372 | 64 |
| GO:CC | membrane | GO:0016020 | 8518 | 3.60E-34 | 372 | 203 |
| REAC | Innate Immune System | REAC:R-HSA-168249 | 914 | 8.80E-33 | 372 | 66 |
| GO:BP | response to external stimulus | GO:0009605 | 2310 | 2.40E-32 | 372 | 101 |
| GO:BP | response to stimulus | GO:0050896 | 7482 | 1.60E-30 | 372 | 184 |
| GO:BP | inflammatory response | GO:0006954 | 651 | 1.20E-28 | 372 | 54 |
| GO:BP | immune effector process | GO:0002252 | 513 | 1.50E-28 | 372 | 49 |
| GO:BP | regulation of cell activation | GO:0050865 | 541 | 1.60E-28 | 372 | 50 |
| GO:MF | signaling receptor activity | GO:0038023 | 1139 | 5.80E-28 | 372 | 67 |
| GO:MF | molecular transducer activity | GO:0060089 | 1139 | 5.80E-28 | 372 | 67 |
| GO:MF | transmembrane signaling receptor activity | GO:0004888 | 947 | 2.40E-27 | 372 | 61 |
| GO:BP | response to biotic stimulus | GO:0009607 | 1182 | 3.60E-27 | 372 | 68 |
| GO:BP | defense response to other organism | GO:0098542 | 842 | 1.10E-26 | 372 | 58 |
| GO:BP | positive regulation of response to stimulus | GO:0048584 | 1882 | 1.30E-26 | 372 | 84 |
| KEGG | Staphylococcus aureus infection | KEGG:05150 | 60 | 1.40E-26 | 372 | 22 |
| GO:BP | response to other organism | GO:0051707 | 1142 | 2.20E-26 | 372 | 66 |
| GO:BP | response to external biotic stimulus | GO:0043207 | 1145 | 2.60E-26 | 372 | 66 |
| GO:BP | regulation of response to stimulus | GO:0048583 | 3512 | 4.20E-26 | 372 | 115 |
| GO:BP | regulation of leukocyte activation | GO:0002694 | 500 | 6.40E-26 | 372 | 46 |
| GO:BP | leukocyte activation involved in immune response | GO:0002366 | 253 | 3.60E-25 | 372 | 35 |
| GO:BP | biological process involved in interspecies interaction between organisms | GO:0044419 | 1282 | 4.40E-25 | 372 | 68 |
| GO:BP | cell activation involved in immune response | GO:0002263 | 257 | 6.20E-25 | 372 | 35 |
| GO:BP | lymphocyte activation | GO:0046649 | 652 | 1.00E-24 | 372 | 50 |
| GO:BP | biological adhesion | GO:0022610 | 1393 | 1.60E-24 | 372 | 70 |
| GO:BP | positive regulation of immune response | GO:0050778 | 464 | 3.50E-24 | 372 | 43 |
| GO:CC | intrinsic component of plasma membrane | GO:0031226 | 1541 | 3.60E-24 | 372 | 72 |
| GO:CC | integral component of plasma membrane | GO:0005887 | 1466 | 5.70E-24 | 372 | 70 |
| GO:BP | cell adhesion | GO:0007155 | 1388 | 7.60E-24 | 372 | 69 |
| GO:BP | regulation of multicellular organismal process | GO:0051239 | 2403 | 2.70E-23 | 372 | 90 |
| GO:BP | regulation of cytokine production | GO:0001817 | 646 | 5.10E-23 | 372 | 48 |
| GO:BP | cytokine production | GO:0001816 | 652 | 7.70E-23 | 372 | 48 |
| REAC | Neutrophil degranulation | REAC:R-HSA-6798695 | 446 | 1.80E-22 | 372 | 40 |
| GO:BP | innate immune response | GO:0045087 | 667 | 2.10E-22 | 372 | 48 |
| GO:BP | signaling | GO:0023052 | 5550 | 3.40E-22 | 372 | 141 |
| GO:BP | myeloid leukocyte activation | GO:0002274 | 205 | 5.50E-22 | 372 | 30 |
| GO:BP | response to stress | GO:0006950 | 3345 | 7.90E-22 | 372 | 105 |
| GO:BP | cell communication | GO:0007154 | 5601 | 8.50E-22 | 372 | 141 |
| GO:BP | positive regulation of biological process | GO:0048518 | 5512 | 1.90E-21 | 372 | 139 |
| GO:BP | biological regulation | GO:0065007 | 10837 | 2.70E-21 | 372 | 209 |
| GO:BP | signal transduction | GO:0007165 | 5094 | 4.50E-21 | 372 | 132 |
| GO:BP | regulation of biological process | GO:0050789 | 10213 | 4.60E-21 | 372 | 201 |
| GO:CC | extracellular region | GO:0005576 | 3516 | 6.80E-21 | 372 | 105 |
| GO:BP | adaptive immune response | GO:0002250 | 390 | 8.10E-21 | 372 | 37 |
| GO:CC | vesicle | GO:0031982 | 3591 | 9.60E-21 | 372 | 106 |
| GO:BP | activation of immune response | GO:0002253 | 298 | 1.90E-20 | 372 | 33 |
| GO:BP | negative regulation of immune system process | GO:0002683 | 375 | 2.30E-20 | 372 | 36 |
| GO:BP | multicellular organismal process | GO:0032501 | 6431 | 2.80E-20 | 372 | 150 |
| GO:BP | cellular response to stimulus | GO:0051716 | 6392 | 4.60E-20 | 372 | 149 |
| GO:CC | cytoplasmic vesicle | GO:0031410 | 2275 | 6.90E-20 | 372 | 81 |
| GO:CC | intracellular vesicle | GO:0097708 | 2277 | 7.20E-20 | 372 | 81 |
| GO:BP | response to bacterium | GO:0009617 | 504 | 8.50E-20 | 372 | 40 |
| GO:BP | regulation of cellular process | GO:0050794 | 9657 | 1.40E-19 | 372 | 191 |
| GO:BP | immune response-regulating signaling pathway | GO:0002764 | 396 | 1.50E-19 | 372 | 36 |
| GO:CC | cell surface | GO:0009986 | 741 | 1.80E-19 | 372 | 46 |
| GO:BP | leukocyte mediated immunity | GO:0002443 | 320 | 1.90E-19 | 372 | 33 |
| GO:BP | phagocytosis | GO:0006909 | 229 | 2.40E-19 | 372 | 29 |
| GO:BP | positive regulation of multicellular organismal process | GO:0051240 | 1298 | 1.10E-18 | 372 | 60 |
| TF | Factor: GATAD2A; motif: CCTKTG; match class: 1 | TF:M09726_1 | 7641 | 2.00E-18 | 372 | 163 |
| TF | Factor: PEA3; motif: ACWTCCK | TF:M00655 | 8027 | 2.20E-18 | 372 | 168 |
| TF | Factor: AML1; motif: TGTGGT | TF:M02084 | 10268 | 7.00E-18 | 372 | 195 |
| TF | Factor: AML1; motif: TGTGGT | TF:M00751 | 10268 | 7.00E-18 | 372 | 195 |
| TF | Factor: AML1a; motif: TGTGGT | TF:M00271 | 10268 | 7.00E-18 | 372 | 195 |
| GO:BP | T cell activation | GO:0042110 | 448 | 9.30E-18 | 372 | 36 |
| TF | Factor: Smad2; motif: AGACAN; match class: 1 | TF:M03580_1 | 7607 | 1.00E-17 | 372 | 161 |
| REAC | REACTOME root term | REAC:0000000 | 9607 | 1.90E-17 | 372 | 184 |
| GO:CC | secretory granule | GO:0030141 | 760 | 2.20E-17 | 372 | 44 |
| GO:CC | secretory granule membrane | GO:0030667 | 287 | 2.40E-17 | 372 | 29 |
| TF | Factor: SRY; motif: AACAATNR | TF:M08976 | 11740 | 2.70E-17 | 372 | 211 |
| TF | Factor: ZBTB39; motif: CNCTGY | TF:M12689 | 13477 | 4.60E-17 | 372 | 230 |
| TF | Factor: PEA3; motif: NNCAGGAARNN | TF:M09915 | 10849 | 4.90E-17 | 372 | 200 |
| GO:BP | leukocyte migration | GO:0050900 | 329 | 5.60E-17 | 372 | 31 |
| TF | Factor: NMYC; motif: CAYCTG | TF:M01808 | 10951 | 5.90E-17 | 372 | 201 |
| GO:BP | regulation of lymphocyte activation | GO:0051249 | 415 | 6.60E-17 | 372 | 34 |
| GO:BP | positive regulation of cytokine production | GO:0001819 | 416 | 7.10E-17 | 372 | 34 |
| TF | Factor: GATAD2A; motif: CCTKTG | TF:M09726 | 13089 | 9.30E-17 | 372 | 225 |
| TF | Factor: Spi-B; motif: TTCYBC; match class: 1 | TF:M03851_1 | 17095 | 9.60E-17 | 372 | 267 |
| TF | Factor: Spi-B; motif: TTCYBC | TF:M03851 | 17110 | 1.10E-16 | 372 | 267 |
| GO:BP | leukocyte cell-cell adhesion | GO:0007159 | 338 | 1.20E-16 | 372 | 31 |
| TF | Transfac | TF:M00000 | 17114 | 1.20E-16 | 372 | 267 |
| GO:BP | leukocyte proliferation | GO:0070661 | 286 | 1.30E-16 | 372 | 29 |
| GO:BP | myeloid cell activation involved in immune response | GO:0002275 | 87 | 1.30E-16 | 372 | 19 |
| GO:CC | extracellular space | GO:0005615 | 2749 | 1.50E-16 | 372 | 84 |
| GO:BP | cell surface receptor signaling pathway | GO:0007166 | 2456 | 1.70E-16 | 372 | 80 |
| KEGG | Systemic lupus erythematosus | KEGG:05322 | 104 | 1.90E-16 | 372 | 19 |
| GO:CC | vesicle membrane | GO:0012506 | 1138 | 2.30E-16 | 372 | 52 |
| GO:CC | cellular anatomical entity | GO:0110165 | 16869 | 4.70E-16 | 372 | 261 |
| TF | Factor: PU.1; motif: AGGAAG | TF:M02031 | 15486 | 5.00E-16 | 372 | 249 |
| TF | Factor: Elf-1; motif: AGGAAG | TF:M01266 | 15486 | 5.00E-16 | 372 | 249 |
| GO:BP | cell-cell adhesion | GO:0098609 | 828 | 5.40E-16 | 372 | 45 |
| GO:CC | cytoplasmic vesicle membrane | GO:0030659 | 1119 | 5.80E-16 | 372 | 51 |
| GO:BP | positive regulation of cell activation | GO:0050867 | 330 | 6.40E-16 | 372 | 30 |
| TF | Factor: Sox-18; motif: CAAWGBB | TF:M03848 | 15043 | 7.10E-16 | 372 | 244 |
| TF | Factor: ZBTB39; motif: CNCTGY; match class: 1 | TF:M12689_1 | 8347 | 1.10E-15 | 372 | 166 |
| TF | Factor: ZNF35; motif: SSAAKA | TF:M07479 | 16059 | 1.20E-15 | 372 | 254 |
| GO:CC | cellular_component | GO:0005575 | 16974 | 1.30E-15 | 372 | 261 |
| TF | Factor: Kid3; motif: CCACN | TF:M01160 | 17056 | 1.30E-15 | 372 | 264 |
| KEGG | KEGG root term | KEGG:00000 | 7044 | 1.40E-15 | 372 | 145 |
| KEGG | Leishmaniasis | KEGG:05140 | 71 | 2.70E-15 | 372 | 16 |
| TF | Factor: Smad2; motif: AGACAN | TF:M03580 | 13070 | 3.40E-15 | 372 | 221 |
| KEGG | Tuberculosis | KEGG:05152 | 162 | 4.80E-15 | 372 | 21 |
| GO:BP | cell population proliferation | GO:0008283 | 1750 | 6.00E-15 | 372 | 64 |
| GO:BP | regulation of developmental process | GO:0050793 | 2221 | 6.10E-15 | 372 | 73 |
| GO:BP | regulation of immune effector process | GO:0002697 | 302 | 6.10E-15 | 372 | 28 |
| TF | Factor: NMYC; motif: CAYCTG; match class: 1 | TF:M01808_1 | 5049 | 7.70E-15 | 372 | 119 |
| GO:MF | molecular_function | GO:0003674 | 16562 | 8.40E-15 | 372 | 255 |
| TF | Factor: ING4; motif: CCACCA | TF:M01743 | 13898 | 9.40E-15 | 372 | 229 |
| GO:BP | lymphocyte proliferation | GO:0046651 | 261 | 1.70E-14 | 372 | 26 |
| GO:CC | side of membrane | GO:0098552 | 492 | 1.70E-14 | 372 | 33 |
| REAC | Adaptive Immune System | REAC:R-HSA-1280218 | 709 | 2.20E-14 | 372 | 39 |
| GO:BP | mononuclear cell proliferation | GO:0032943 | 264 | 2.30E-14 | 372 | 26 |
| GO:MF | immune receptor activity | GO:0140375 | 122 | 2.30E-14 | 372 | 19 |
| TF | Factor: Sox-18; motif: CAAWGBB; match class: 1 | TF:M03848_1 | 10894 | 2.40E-14 | 372 | 194 |
| GO:BP | positive regulation of leukocyte activation | GO:0002696 | 319 | 2.60E-14 | 372 | 28 |
| GO:CC | secretory vesicle | GO:0099503 | 920 | 2.80E-14 | 372 | 44 |
| TF | Factor: ETV4; motif: NCAGGAAGNN | TF:M12556 | 9336 | 2.80E-14 | 372 | 175 |
| GO:BP | regulation of response to external stimulus | GO:0032101 | 839 | 3.10E-14 | 372 | 43 |
| KEGG | Neutrophil extracellular trap formation | KEGG:04613 | 159 | 4.90E-14 | 372 | 20 |
| TF | Factor: Kid3; motif: CCACN; match class: 1 | TF:M01160_1 | 16941 | 5.00E-14 | 372 | 259 |
| TF | Factor: PU.1; motif: NNNNYYYACTTCCTCTTTY | TF:M01172 | 3663 | 5.80E-14 | 372 | 96 |
| GO:BP | regulation of leukocyte cell-cell adhesion | GO:1903037 | 302 | 6.30E-14 | 372 | 27 |
| GO:BP | immune response-regulating cell surface receptor signaling pathway | GO:0002768 | 251 | 7.50E-14 | 372 | 25 |
| TF | Factor: Zbtb44; motif: CKGTGA | TF:M05405 | 11777 | 7.50E-14 | 372 | 203 |
| TF | Factor: PARP; motif: TTTCYN | TF:M02027 | 14025 | 8.10E-14 | 372 | 228 |
| KEGG | Phagosome | KEGG:04145 | 143 | 9.40E-14 | 372 | 19 |
| TF | Factor: BRN1; motif: HAATGCN | TF:M03813 | 11080 | 1.70E-13 | 372 | 194 |
| GO:BP | positive regulation of immune effector process | GO:0002699 | 211 | 1.90E-13 | 372 | 23 |
| GO:BP | regulation of tumor necrosis factor superfamily cytokine production | GO:1903555 | 145 | 2.00E-13 | 372 | 20 |
| GO:BP | tumor necrosis factor superfamily cytokine production | GO:0071706 | 145 | 2.00E-13 | 372 | 20 |
| GO:BP | response to chemical | GO:0042221 | 3698 | 2.30E-13 | 372 | 95 |
| GO:BP | regulation of cell population proliferation | GO:0042127 | 1482 | 2.50E-13 | 372 | 56 |
| REAC | Immunoregulatory interactions between a Lymphoid and a non-Lymphoid cell | REAC:R-HSA-198933 | 101 | 2.70E-13 | 372 | 17 |
| TF | Factor: ZNF35; motif: SSAAKA; match class: 1 | TF:M07479_1 | 13256 | 3.30E-13 | 372 | 218 |
| TF | Factor: Lyl-1; motif: NCAKCTGYTNYCNN | TF:M09977 | 11924 | 3.30E-13 | 372 | 203 |
| GO:BP | cellular process | GO:0009987 | 15441 | 3.60E-13 | 372 | 241 |
| GO:CC | external side of plasma membrane | GO:0009897 | 291 | 4.30E-13 | 372 | 25 |
| GO:BP | regulation of leukocyte proliferation | GO:0070663 | 219 | 4.40E-13 | 372 | 23 |
| GO:BP | anatomical structure development | GO:0048856 | 5153 | 5.60E-13 | 372 | 116 |
| TF | Factor: IRF-4; motif: GAAARTA | TF:M01883 | 6944 | 6.30E-13 | 372 | 141 |
| GO:CC | endomembrane system | GO:0012505 | 4326 | 6.50E-13 | 372 | 102 |
| GO:BP | positive regulation of leukocyte cell-cell adhesion | GO:1903039 | 223 | 6.60E-13 | 372 | 23 |
| GO:BP | negative regulation of biological process | GO:0048519 | 4967 | 7.60E-13 | 372 | 113 |
| TF | Factor: FOXO1A:Elk-1; motif: RWMAACAGGAAGTN | TF:M08299 | 6145 | 7.60E-13 | 372 | 130 |
| GO:BP | immune response-activating cell surface receptor signaling pathway | GO:0002429 | 226 | 8.80E-13 | 372 | 23 |
| GO:BP | immune response-activating signal transduction | GO:0002757 | 226 | 8.80E-13 | 372 | 23 |
| GO:BP | biological_process | GO:0008150 | 16323 | 9.90E-13 | 372 | 249 |
| GO:BP | cellular response to biotic stimulus | GO:0071216 | 203 | 1.00E-12 | 372 | 22 |
| GO:BP | regulation of cell-cell adhesion | GO:0022407 | 400 | 1.10E-12 | 372 | 29 |
| TF | Factor: SRY; motif: AACAATANCATTGTT | TF:M04555 | 5745 | 1.20E-12 | 372 | 124 |
| GO:BP | regulation of leukocyte mediated immunity | GO:0002703 | 205 | 1.30E-12 | 372 | 22 |
| GO:BP | chemotaxis | GO:0006935 | 538 | 1.30E-12 | 372 | 33 |
| TF | Factor: c-Ets-1; motif: RCAGGAAGTGNNTNS | TF:M00339 | 5893 | 1.30E-12 | 372 | 126 |
| TF | Factor: FOXO1A:PDEF; motif: WNCCGGATGTTDN | TF:M08486 | 9927 | 1.30E-12 | 372 | 178 |
| TF | Factor: PARP; motif: TTTCYN; match class: 1 | TF:M02027_1 | 10180 | 1.30E-12 | 372 | 181 |
| GO:BP | developmental process | GO:0032502 | 5648 | 1.50E-12 | 372 | 122 |
| GO:BP | taxis | GO:0042330 | 542 | 1.70E-12 | 372 | 33 |
| GO:BP | regulation of myeloid leukocyte mediated immunity | GO:0002886 | 57 | 1.80E-12 | 372 | 14 |
| GO:BP | regulation of tumor necrosis factor production | GO:0032680 | 141 | 1.80E-12 | 372 | 19 |
| GO:BP | tumor necrosis factor production | GO:0032640 | 141 | 1.80E-12 | 372 | 19 |
| TF | Factor: PU.1; motif: AGGAAG; match class: 1 | TF:M02031_1 | 11933 | 2.00E-12 | 372 | 201 |
| TF | Factor: Elf-1; motif: AGGAAG; match class: 1 | TF:M01266_1 | 11933 | 2.00E-12 | 372 | 201 |
| TF | Factor: ZNF333; motif: ATAAT | TF:M01230 | 13185 | 2.20E-12 | 372 | 215 |
| TF | Factor: Sox-17; motif: TTGTYY | TF:M03803 | 13366 | 2.20E-12 | 372 | 217 |
| GO:BP | regulation of multicellular organismal development | GO:2000026 | 1218 | 2.30E-12 | 372 | 49 |
| TF | Factor: SMAD3; motif: TGTCTGTCT | TF:M00701 | 3555 | 2.30E-12 | 372 | 91 |
| TF | Factor: sin3A; motif: TGTCCNNGGTGCTG | TF:M04756 | 12573 | 2.50E-12 | 372 | 208 |
| GO:BP | regulation of localization | GO:0032879 | 2549 | 2.70E-12 | 372 | 74 |
| GO:BP | positive regulation of cellular process | GO:0048522 | 5061 | 2.90E-12 | 372 | 113 |
| GO:BP | negative regulation of cell activation | GO:0050866 | 190 | 3.20E-12 | 372 | 21 |
| TF | Factor: Cdx-1; motif: TTTATK | TF:M02086 | 12282 | 4.60E-12 | 372 | 204 |
| TF | Factor: Sox-17; motif: TTGTYY; match class: 1 | TF:M03803_1 | 7955 | 4.70E-12 | 372 | 152 |
| TF | Factor: SMAD3; motif: CAGACAS | TF:M07429 | 5352 | 4.90E-12 | 372 | 117 |
| GO:BP | negative regulation of cellular process | GO:0048523 | 4412 | 5.00E-12 | 372 | 103 |
| TF | Factor: SATB1; motif: NTTTAT | TF:M03564 | 13565 | 6.00E-12 | 372 | 218 |
| TF | Factor: CPBP; motif: SNCCCNN | TF:M01822 | 16885 | 6.40E-12 | 372 | 253 |
| GO:BP | regulation of leukocyte migration | GO:0002685 | 197 | 6.70E-12 | 372 | 21 |
| GO:BP | regulation of T cell activation | GO:0050863 | 304 | 6.80E-12 | 372 | 25 |
| TF | Factor: myogenin; motif: CAGCTG | TF:M02101 | 11284 | 7.30E-12 | 372 | 192 |
| TF | Factor: myogenin; motif: CAGCTG; match class: 1 | TF:M02101_1 | 11284 | 7.30E-12 | 372 | 192 |
| GO:MF | binding | GO:0005488 | 15390 | 7.60E-12 | 372 | 235 |
| KEGG | Hematopoietic cell lineage | KEGG:04640 | 78 | 7.80E-12 | 372 | 14 |
| GO:BP | macrophage activation | GO:0042116 | 94 | 7.90E-12 | 372 | 16 |
| TF | Factor: PEBP2beta; motif: TGTGGTY | TF:M03841 | 6476 | 7.90E-12 | 372 | 132 |
| GO:BP | regulation of cell adhesion | GO:0030155 | 687 | 8.00E-12 | 372 | 36 |
| GO:BP | cellular response to molecule of bacterial origin | GO:0071219 | 176 | 9.00E-12 | 372 | 20 |
| TF | Factor: C-Jun; motif: TGACTC | TF:M03541 | 9625 | 9.40E-12 | 372 | 172 |
| TF | Factor: GR; motif: AGAACAN | TF:M07355 | 4913 | 9.90E-12 | 372 | 110 |
| TF | Factor: MEL1; motif: GATGAG | TF:M08793 | 10128 | 9.90E-12 | 372 | 178 |
| GO:BP | hemopoiesis | GO:0030097 | 815 | 1.00E-11 | 372 | 39 |
| GO:BP | myeloid leukocyte mediated immunity | GO:0002444 | 96 | 1.10E-11 | 372 | 16 |
| GO:BP | vesicle-mediated transport | GO:0016192 | 1466 | 1.10E-11 | 372 | 53 |
| GO:BP | leukocyte differentiation | GO:0002521 | 507 | 1.20E-11 | 372 | 31 |
| GO:BP | regulation of lymphocyte proliferation | GO:0050670 | 203 | 1.20E-11 | 372 | 21 |
| KEGG | Cell adhesion molecules | KEGG:04514 | 139 | 1.20E-11 | 372 | 17 |
| TF | Factor: c-Ets-2; motif: CTTCCTG | TF:M01207 | 8932 | 1.40E-11 | 372 | 163 |
| TF | Factor: Elk-1; motif: CTTCCKG | TF:M07252 | 8932 | 1.40E-11 | 372 | 163 |
| GO:BP | regulation of mononuclear cell proliferation | GO:0032944 | 205 | 1.50E-11 | 372 | 21 |
| TF | Factor: Pax-4; motif: DTTTTCCACCN | TF:M04624 | 13570 | 1.50E-11 | 372 | 217 |
| GO:BP | cellular developmental process | GO:0048869 | 3759 | 1.60E-11 | 372 | 92 |
| GO:BP | positive regulation of cell-cell adhesion | GO:0022409 | 259 | 1.70E-11 | 372 | 23 |
| TF | Factor: Smad2; motif: TGTCTGNCACCT | TF:M09656 | 5444 | 1.70E-11 | 372 | 117 |
| GO:BP | defense response to bacterium | GO:0042742 | 183 | 1.90E-11 | 372 | 20 |
| TF | Factor: Smad3; motif: NGNCAGACASNNN | TF:M01888 | 3489 | 1.90E-11 | 372 | 88 |
| TF | Factor: Pax-4; motif: NNNNNYCACCCB | TF:M00378 | 15748 | 1.90E-11 | 372 | 240 |
| TF | Factor: AP-4; motif: AWCAGCTGWT | TF:M11206 | 6263 | 2.20E-11 | 372 | 128 |
| TF | Factor: AP-4; motif: AWCAGCTGWT; match class: 1 | TF:M11206_1 | 6263 | 2.20E-11 | 372 | 128 |
| GO:BP | response to molecule of bacterial origin | GO:0002237 | 292 | 2.50E-11 | 372 | 24 |
| TF | Factor: p300; motif: CTGNCTYMAN | TF:M07266 | 6955 | 2.80E-11 | 372 | 137 |
| GO:BP | system development | GO:0048731 | 4335 | 3.20E-11 | 372 | 100 |
| GO:BP | hematopoietic or lymphoid organ development | GO:0048534 | 846 | 3.40E-11 | 372 | 39 |
| TF | Factor: GKLF; motif: CCTCCYN | TF:M01835 | 13756 | 3.40E-11 | 372 | 218 |
| TF | Factor: ETV4; motif: NCAGGAAGNN; match class: 1 | TF:M12556_1 | 3399 | 3.50E-11 | 372 | 86 |
| GO:BP | cell differentiation | GO:0030154 | 3683 | 3.80E-11 | 372 | 90 |
| KEGG | Complement and coagulation cascades | KEGG:04610 | 70 | 3.80E-11 | 372 | 13 |
| TF | Factor: Oct3; motif: NYWTTSWTATGCAAAT | TF:M09646 | 4663 | 3.80E-11 | 372 | 105 |
| GO:MF | protein binding | GO:0005515 | 13368 | 4.40E-11 | 372 | 211 |
| TF | Factor: Zbtb44; motif: CKGTGA; match class: 1 | TF:M05405_1 | 5881 | 4.60E-11 | 372 | 122 |
| TF | Factor: PEA3; motif: ACWTCCK; match class: 1 | TF:M00655_1 | 2384 | 5.00E-11 | 372 | 69 |
| GO:MF | carbohydrate binding | GO:0030246 | 236 | 5.20E-11 | 372 | 21 |
| GO:BP | negative regulation of leukocyte activation | GO:0002695 | 169 | 5.40E-11 | 372 | 19 |
| TF | Factor: nerf; motif: YRNCAGGAAGYRGSTBDS | TF:M00531 | 6635 | 5.40E-11 | 372 | 132 |
| GO:BP | regulation of phagocytosis | GO:0050764 | 88 | 5.60E-11 | 372 | 15 |
| TF | Factor: SPI1; motif: NNNAAAGAGGAAGTGANNNN | TF:M12497 | 1992 | 5.60E-11 | 372 | 62 |
| TF | Factor: Elk-1; motif: GGAAGN | TF:M03819 | 12382 | 6.10E-11 | 372 | 202 |
| TF | Factor: MAFB; motif: NTCAGCN | TF:M08888 | 8591 | 6.40E-11 | 372 | 157 |
| GO:BP | humoral immune response | GO:0006959 | 171 | 6.70E-11 | 372 | 19 |
| TF | Factor: PEA3; motif: NNCAGGAARNN; match class: 1 | TF:M09915_1 | 4914 | 6.90E-11 | 372 | 108 |
| GO:BP | leukocyte degranulation | GO:0043299 | 73 | 7.40E-11 | 372 | 14 |
| TF | Factor: NF1C; motif: WGCCARR | TF:M09763 | 9428 | 8.30E-11 | 372 | 167 |
| TF | Factor: STAT; motif: NNNNNTTCTKGGA | TF:M00777 | 4378 | 8.60E-11 | 372 | 100 |
| GO:BP | cellular response to cytokine stimulus | GO:0071345 | 744 | 8.80E-11 | 372 | 36 |
| TF | Factor: FOXO1A:Net; motif: RWMAACAGGAAGTN | TF:M08302 | 7909 | 9.00E-11 | 372 | 148 |
| TF | Factor: JUNB:C-JUN; motif: NATGACKCAT | TF:M08944 | 4454 | 9.60E-11 | 372 | 101 |
| GO:BP | response to cytokine | GO:0034097 | 833 | 1.00E-10 | 372 | 38 |
| GO:CC | tertiary granule membrane | GO:0070821 | 68 | 1.10E-10 | 372 | 13 |
| TF | Factor: NFATc2; motif: GGAAAA | TF:M01281 | 13979 | 1.10E-10 | 372 | 219 |
| TF | Factor: NFATc3; motif: GGAAAA | TF:M01886 | 13979 | 1.10E-10 | 372 | 219 |
| TF | Factor: NFATc2; motif: GGAAAA | TF:M03555 | 13979 | 1.10E-10 | 372 | 219 |
| TF | Factor: Sox-9; motif: NNNACAATRG | TF:M11626 | 5446 | 1.10E-10 | 372 | 115 |
| GO:BP | multicellular organism development | GO:0007275 | 4634 | 1.20E-10 | 372 | 103 |
| TF | Factor: IRF-7; motif: AAGWGAA | TF:M01884 | 9133 | 1.20E-10 | 372 | 163 |
| TF | Factor: PMX1; motif: TAATHA | TF:M03560 | 11838 | 1.20E-10 | 372 | 195 |
| TF | Factor: PU.1; motif: NRAAAGAGGAAGTGRNN | TF:M10073 | 2088 | 1.40E-10 | 372 | 63 |
| TF | Factor: AML1; motif: TGTGGT; match class: 1 | TF:M00751_1 | 4420 | 1.60E-10 | 372 | 100 |
| TF | Factor: AML1; motif: TGTGGT; match class: 1 | TF:M02084_1 | 4420 | 1.60E-10 | 372 | 100 |
| TF | Factor: AML1a; motif: TGTGGT; match class: 1 | TF:M00271_1 | 4420 | 1.60E-10 | 372 | 100 |
| GO:BP | positive regulation of cell communication | GO:0010647 | 1569 | 1.70E-10 | 372 | 53 |
| GO:BP | positive regulation of signaling | GO:0023056 | 1573 | 1.90E-10 | 372 | 53 |
| GO:CC | extracellular vesicle | GO:1903561 | 1921 | 1.90E-10 | 372 | 58 |
| GO:CC | extracellular organelle | GO:0043230 | 1922 | 1.90E-10 | 372 | 58 |
| GO:CC | extracellular membrane-bounded organelle | GO:0065010 | 1922 | 1.90E-10 | 372 | 58 |
| TF | Factor: SPI1; motif: AGGAAGT | TF:M02278 | 5850 | 1.90E-10 | 372 | 120 |
| TF | Factor: PEA3; motif: AGGAAGT | TF:M03579 | 5850 | 1.90E-10 | 372 | 120 |
| TF | Factor: C-ets-1; motif: AGGAAGN | TF:M01870 | 5850 | 1.90E-10 | 372 | 120 |
| TF | Factor: Fli-1; motif: MGGAAGT | TF:M07382 | 5850 | 1.90E-10 | 372 | 120 |
| GO:CC | bounding membrane of organelle | GO:0098588 | 2037 | 2.00E-10 | 372 | 60 |
| TF | Factor: T3R-beta; motif: NTGACCTYRNYRAGGTCAN | TF:M11818 | 10182 | 2.00E-10 | 372 | 175 |
| GO:CC | tertiary granule | GO:0070820 | 151 | 2.10E-10 | 372 | 17 |
| TF | Factor: AP-4; motif: ANCATATGNT | TF:M11207 | 2574 | 2.10E-10 | 372 | 71 |
| TF | Factor: YY1; motif: NGCCATYTTKGRCNWWNNGTGCK | TF:M12276 | 8868 | 2.10E-10 | 372 | 159 |
| TF | Factor: GTF2IRD1-isoform2; motif: GGGATTRNR | TF:M01229 | 13062 | 2.20E-10 | 372 | 208 |
| GO:BP | T cell proliferation | GO:0042098 | 183 | 2.30E-10 | 372 | 19 |
| GO:BP | immune system development | GO:0002520 | 899 | 2.30E-10 | 372 | 39 |
| TF | Factor: FOSB:JUND; motif: NNTNACTNATN | TF:M08928 | 5006 | 2.40E-10 | 372 | 108 |
| TF | Factor: MEL1; motif: GATGAG; match class: 1 | TF:M08793_1 | 4116 | 2.60E-10 | 372 | 95 |
| GO:BP | positive regulation of response to external stimulus | GO:0032103 | 390 | 2.70E-10 | 372 | 26 |
| TF | Factor: Pax-6; motif: CTGACCTGGAACTM | TF:M00979 | 8330 | 2.70E-10 | 372 | 152 |
| TF | Factor: HOXA3; motif: NNNNRNTAATTARY | TF:M01337 | 13550 | 3.00E-10 | 372 | 213 |
| GO:BP | myeloid leukocyte migration | GO:0097529 | 186 | 3.10E-10 | 372 | 19 |
| TF | Factor: SPI1; motif: AAAAAGCGGAAGTW | TF:M03994 | 1851 | 3.40E-10 | 372 | 58 |
| TF | Factor: ZNF462; motif: YYYYCTSCWG | TF:M12707 | 8440 | 3.70E-10 | 372 | 153 |
| GO:BP | negative regulation of multicellular organismal process | GO:0051241 | 913 | 3.80E-10 | 372 | 39 |
| GO:CC | extracellular exosome | GO:0070062 | 1901 | 4.10E-10 | 372 | 57 |
| GO:MF | protein-containing complex binding | GO:0044877 | 1204 | 4.20E-10 | 372 | 44 |
| TF | Factor: AP-4; motif: AHCATRTGKT | TF:M11213 | 3359 | 4.20E-10 | 372 | 83 |
| TF | Factor: SRY; motif: TTGTTT | TF:M03854 | 12068 | 4.20E-10 | 372 | 196 |
| TF | Factor: FOXO1A; motif: AAACAA | TF:M03823 | 12068 | 4.20E-10 | 372 | 196 |
| GO:BP | locomotion | GO:0040011 | 1669 | 5.30E-10 | 372 | 54 |
| GO:BP | cellular response to lipopolysaccharide | GO:0071222 | 168 | 6.00E-10 | 372 | 18 |
| GO:BP | adaptive immune response based on somatic recombination of immune receptors built from immunoglobulin superfamily domains | GO:0002460 | 247 | 6.10E-10 | 372 | 21 |
| GO:BP | B cell activation | GO:0042113 | 247 | 6.10E-10 | 372 | 21 |
| GO:BP | leukocyte chemotaxis | GO:0030595 | 193 | 6.10E-10 | 372 | 19 |
| GO:BP | regulation of transport | GO:0051049 | 1622 | 6.20E-10 | 372 | 53 |
| GO:BP | positive regulation of lymphocyte activation | GO:0051251 | 277 | 6.50E-10 | 372 | 22 |
| GO:CC | endocytic vesicle membrane | GO:0030666 | 187 | 6.70E-10 | 372 | 18 |
| TF | Factor: SMAD; motif: TNGNCAGACWN | TF:M00974 | 3788 | 7.60E-10 | 372 | 89 |
| TF | Factor: DREF; motif: CTYYCWCTTCCY | TF:M09725 | 6415 | 7.90E-10 | 372 | 126 |
| GO:BP | positive regulation of cell adhesion | GO:0045785 | 409 | 8.00E-10 | 372 | 26 |
| TF | Factor: c-Ets-2; motif: CTTCCTG; match class: 1 | TF:M01207_1 | 3080 | 8.00E-10 | 372 | 78 |
| TF | Factor: Elk-1; motif: CTTCCKG; match class: 1 | TF:M07252_1 | 3080 | 8.00E-10 | 372 | 78 |
| TF | Factor: HES-5; motif: NCACACKY | TF:M11069 | 9505 | 8.90E-10 | 372 | 165 |
| GO:BP | localization | GO:0051179 | 5792 | 9.40E-10 | 372 | 117 |
| TF | Factor: T3R-beta; motif: NTGACCTYRNYRAGGTCAN; match class: 1 | TF:M11818_1 | 9678 | 9.50E-10 | 372 | 167 |
| TF | Factor: GATA-5; motif: TATCTN | TF:M02006 | 7582 | 9.50E-10 | 372 | 141 |
| TF | Factor: MafG; motif: CMATGACTCAGCAGA | TF:M07048 | 5916 | 1.00E-09 | 372 | 119 |
| GO:BP | cellular response to chemical stimulus | GO:0070887 | 2745 | 1.10E-09 | 372 | 72 |
| GO:BP | positive regulation of developmental process | GO:0051094 | 1182 | 1.10E-09 | 372 | 44 |
| TF | Factor: egr-3; motif: GTGGGY | TF:M03818 | 11653 | 1.10E-09 | 372 | 190 |
| TF | Factor: TFII-I; motif: NAGGAAGTGN | TF:M04636 | 11048 | 1.10E-09 | 372 | 183 |
| TF | Factor: C/EBP; motif: NNNTKNNGNAAN | TF:M00770 | 4639 | 1.20E-09 | 372 | 101 |
| GO:BP | positive regulation of T cell activation | GO:0050870 | 202 | 1.40E-09 | 372 | 19 |
| GO:BP | positive regulation of signal transduction | GO:0009967 | 1394 | 1.50E-09 | 372 | 48 |
| GO:BP | positive regulation of gene expression | GO:0010628 | 1001 | 1.50E-09 | 372 | 40 |
| TF | Factor: HSF4; motif: CTGCMRN | TF:M07322 | 14568 | 1.50E-09 | 372 | 222 |
| GO:BP | transport | GO:0006810 | 4269 | 1.60E-09 | 372 | 95 |
| TF | Factor: SRY; motif: TTGTTT; match class: 1 | TF:M03854_1 | 7628 | 1.60E-09 | 372 | 141 |
| TF | Factor: FOXO1A; motif: AAACAA; match class: 1 | TF:M03823_1 | 7628 | 1.60E-09 | 372 | 141 |
| TF | Factor: SRY; motif: AACAATNR; match class: 1 | TF:M08976_1 | 6555 | 1.60E-09 | 372 | 127 |
| TF | Factor: Sox-4; motif: AACAAA | TF:M03849 | 12223 | 1.70E-09 | 372 | 196 |
| GO:BP | establishment of localization | GO:0051234 | 4416 | 1.80E-09 | 372 | 97 |
| GO:BP | positive regulation of tumor necrosis factor superfamily cytokine production | GO:1903557 | 91 | 1.80E-09 | 372 | 14 |
| TF | Factor: THAP1; motif: YTGCCCNNA | TF:M07407 | 9580 | 1.80E-09 | 372 | 165 |
| TF | Factor: Erg; motif: MCAGGAAA | TF:M07284 | 9694 | 2.40E-09 | 372 | 166 |
| GO:BP | regulation of molecular function | GO:0065009 | 2797 | 2.60E-09 | 372 | 72 |
| TF | Factor: SREBP-1; motif: CACSCCA | TF:M00749 | 7520 | 2.60E-09 | 372 | 139 |
| TF | Factor: Six-6; motif: NNSTATCRNN | TF:M11037 | 4351 | 2.70E-09 | 372 | 96 |
| GO:CC | endocytic vesicle | GO:0030139 | 325 | 2.80E-09 | 372 | 22 |
| TF | Factor: FOXM1; motif: NAGASTGATTA | TF:M04611 | 7843 | 2.80E-09 | 372 | 143 |
| GO:BP | positive regulation of transport | GO:0051050 | 838 | 2.90E-09 | 372 | 36 |
| TF | Factor: BCL-11A; motif: NAAAGAGGAAGTGARAN | TF:M09595 | 3159 | 2.90E-09 | 372 | 78 |
| TF | Factor: RARG; motif: RAGGTCAASYARAGGTCA | TF:M04485 | 4222 | 3.00E-09 | 372 | 94 |
| TF | Factor: POU2F1:Gscl; motif: NNGATTANNTATKCANNNN | TF:M08404 | 5279 | 3.10E-09 | 372 | 109 |
| TF | Factor: HLTF; motif: AGSCARAAAGYRGSTGS | TF:M04613 | 6765 | 3.10E-09 | 372 | 129 |
| TF | Factor: Erg; motif: NRRSAGGAAGNGG | TF:M09907 | 10225 | 3.20E-09 | 372 | 172 |
| TF | Factor: MEF-2C; motif: TATTTWT | TF:M02025 | 10824 | 3.30E-09 | 372 | 179 |
| TF | Factor: Sox-10; motif: NACAAWG | TF:M02116 | 12073 | 4.40E-09 | 372 | 193 |
| TF | Factor: ZXDA; motif: NAGGGTG | TF:M06203 | 6420 | 4.50E-09 | 372 | 124 |
| TF | Factor: ZXDB; motif: NAGGGTG | TF:M06202 | 6420 | 4.50E-09 | 372 | 124 |
| TF | Factor: ZBRK1; motif: NGNNNGGTNAWAAAARRGCNG | TF:M10468 | 9361 | 5.20E-09 | 372 | 161 |
| TF | Factor: SREBP; motif: VNNVTCACCCYA | TF:M00776 | 5467 | 5.20E-09 | 372 | 111 |
| GO:BP | response to lipopolysaccharide | GO:0032496 | 277 | 5.60E-09 | 372 | 21 |
| GO:BP | regulation of cell differentiation | GO:0045595 | 1400 | 6.30E-09 | 372 | 47 |
| GO:BP | neutrophil activation involved in immune response | GO:0002283 | 17 | 6.80E-09 | 372 | 8 |
| TF | Factor: RelA-p65; motif: AAASTCCC | TF:M04849 | 5419 | 7.00E-09 | 372 | 110 |
| TF | Factor: myogenin; motif: RGCAGSTG | TF:M00712 | 6086 | 7.20E-09 | 372 | 119 |
| TF | Factor: JUND; motif: NATGAGTCAT | TF:M08934 | 3610 | 7.30E-09 | 372 | 84 |
| GO:BP | regulation of hydrolase activity | GO:0051336 | 870 | 8.50E-09 | 372 | 36 |
| GO:BP | regulation of leukocyte degranulation | GO:0043300 | 50 | 8.60E-09 | 372 | 11 |
| GO:BP | regulation of catalytic activity | GO:0050790 | 2138 | 8.70E-09 | 372 | 60 |
| GO:BP | synapse pruning | GO:0098883 | 11 | 9.40E-09 | 372 | 7 |
| TF | Factor: SRY; motif: TCAATAMCATTGA | TF:M04557 | 8365 | 9.50E-09 | 372 | 148 |
| GO:BP | cell chemotaxis | GO:0060326 | 255 | 1.00E-08 | 372 | 20 |
| TF | Factor: SPI1; motif: NNAAWGNGGAASTNNNN | TF:M01203 | 2304 | 1.00E-08 | 372 | 63 |
| TF | Factor: AP-4; motif: ANCATATGNT | TF:M11209 | 2930 | 1.20E-08 | 372 | 73 |
| TF | Factor: PU.1; motif: WGAGGAAG | TF:M00658 | 3851 | 1.30E-08 | 372 | 87 |
| GO:BP | lymphocyte activation involved in immune response | GO:0002285 | 175 | 1.40E-08 | 372 | 17 |
| TF | Factor: Erg; motif: NRRSAGGAAGNGG; match class: 1 | TF:M09907_1 | 4480 | 1.50E-08 | 372 | 96 |
| TF | Factor: NFATc2; motif: NTTTCCRTNNAYGGAAAN; match class: 1 | TF:M11984_1 | 9477 | 1.50E-08 | 372 | 161 |
| TF | Factor: DREF; motif: CTYYCWCTTCCY; match class: 1 | TF:M09725_1 | 1747 | 1.50E-08 | 372 | 53 |
| TF | Factor: ING4; motif: CCACCA; match class: 1 | TF:M01743_1 | 8903 | 1.50E-08 | 372 | 154 |
| TF | Factor: NFATc1; motif: NTTTCCRTNNAYGGAAAN; match class: 1 | TF:M11979_1 | 7539 | 1.50E-08 | 372 | 137 |
| GO:BP | cell killing | GO:0001906 | 128 | 1.60E-08 | 372 | 15 |
| GO:BP | animal organ development | GO:0048513 | 3102 | 1.70E-08 | 372 | 75 |
| GO:CC | specific granule | GO:0042581 | 145 | 1.70E-08 | 372 | 15 |
| TF | Factor: MEIS1B:HOXA9; motif: TGACASTTTWAYRR | TF:M00421 | 5493 | 1.70E-08 | 372 | 110 |
| TF | Factor: STAT5A; motif: TTCCNRGAANNNNNNTTCCNNGRR | TF:M00460 | 6618 | 1.70E-08 | 372 | 125 |
| GO:BP | cell migration | GO:0016477 | 1339 | 1.80E-08 | 372 | 45 |
| GO:BP | mononuclear cell differentiation | GO:1903131 | 397 | 1.90E-08 | 372 | 24 |
| GO:BP | regulation of vesicle-mediated transport | GO:0060627 | 509 | 1.90E-08 | 372 | 27 |
| GO:BP | positive regulation of tumor necrosis factor production | GO:0032760 | 88 | 2.00E-08 | 372 | 13 |
| KEGG | Intestinal immune network for IgA production | KEGG:04672 | 39 | 2.00E-08 | 372 | 9 |
| GO:BP | regulation of defense response | GO:0031347 | 551 | 2.10E-08 | 372 | 28 |
| KEGG | Pertussis | KEGG:05133 | 72 | 2.20E-08 | 372 | 11 |
| TF | Factor: GR; motif: GGTACAANNTGTYCTK | TF:M00205 | 5664 | 2.20E-08 | 372 | 112 |
| REAC | Cytokine Signaling in Immune system | REAC:R-HSA-1280215 | 634 | 2.30E-08 | 372 | 29 |
| TF | Factor: SPDEF; motif: NASATCCKGNW | TF:M12650 | 1491 | 2.30E-08 | 372 | 48 |
| TF | Factor: NFATc2; motif: NTTTCCRTNNAYGGAAAN | TF:M11984 | 9531 | 2.40E-08 | 372 | 161 |
| TF | Factor: NFE2L1; motif: NNNATGACTCAGCANW | TF:M12499 | 8308 | 2.60E-08 | 372 | 146 |
| TF | Factor: Spic; motif: NGNGGAASTN | TF:M02077 | 7359 | 2.70E-08 | 372 | 134 |
| KEGG | Viral myocarditis | KEGG:05416 | 56 | 3.00E-08 | 372 | 10 |
| TF | Factor: Tbx5; motif: TNAGGTGTKV; match class: 1 | TF:M01020_1 | 3443 | 3.00E-08 | 372 | 80 |
| GO:BP | response to organic substance | GO:0010033 | 2758 | 3.10E-08 | 372 | 69 |
| GO:CC | MHC class II protein complex | GO:0042613 | 15 | 3.10E-08 | 372 | 7 |
| TF | Factor: GATA-3; motif: AGATAAGATCT | TF:M12193 | 5254 | 3.10E-08 | 372 | 106 |
| TF | Factor: Nfe2l1; motif: TGYNNAGTCATT | TF:M07390 | 5626 | 3.30E-08 | 372 | 111 |
| TF | Factor: six-4; motif: ANNNATGACACCNNNNN | TF:M01374 | 5406 | 3.30E-08 | 372 | 108 |
| GO:BP | regulation of hemopoiesis | GO:1903706 | 338 | 3.40E-08 | 372 | 22 |
| TF | Factor: Sox-4; motif: AACAAA; match class: 1 | TF:M03849_1 | 7618 | 3.40E-08 | 372 | 137 |
| GO:BP | interleukin-8 production | GO:0032637 | 73 | 3.50E-08 | 372 | 12 |
| GO:BP | regulation of interleukin-8 production | GO:0032677 | 73 | 3.50E-08 | 372 | 12 |
| TF | Factor: Tbx5; motif: TNAGGTGTKV | TF:M01020 | 9490 | 3.60E-08 | 372 | 160 |
| TF | Factor: c-Ets; motif: KRCAGGAARTRNKT | TF:M00340 | 4483 | 3.70E-08 | 372 | 95 |
| TF | Factor: BRN1; motif: HAATGCN; match class: 1 | TF:M03813_1 | 5787 | 3.80E-08 | 372 | 113 |
| TF | Factor: NKX2-2; motif: NNNCCACTCAANNN | TF:M12456 | 8199 | 4.20E-08 | 372 | 144 |
| TF | Factor: Spi-B; motif: NRAAAGAGGAAGTGARA | TF:M10074 | 1415 | 4.60E-08 | 372 | 46 |
| TF | Factor: PU.1; motif: NRAAAGAGGAAGTGARA | TF:M09659 | 2208 | 4.70E-08 | 372 | 60 |
| TF | Factor: ZNF273; motif: GAGAGGAGCTAC | TF:M10460 | 11221 | 4.70E-08 | 372 | 180 |
| TF | Factor: GCMa:FOXO1A; motif: GTMAATAMGGGTRN | TF:M08318 | 10029 | 4.90E-08 | 372 | 166 |
| TF | Factor: AP-4; motif: ANCATATGNT | TF:M11211 | 2515 | 5.00E-08 | 372 | 65 |
| TF | Factor: c-Jun; motif: NATGACKCATN | TF:M11267 | 2641 | 5.10E-08 | 372 | 67 |
| GO:BP | antigen processing and presentation of peptide antigen via MHC class II | GO:0002495 | 31 | 5.20E-08 | 372 | 9 |
| TF | Factor: NR1B1; motif: NRGGTCANNRGGTCAN | TF:M11795 | 6883 | 5.40E-08 | 372 | 127 |
| KEGG | Rheumatoid arthritis | KEGG:05323 | 78 | 5.50E-08 | 372 | 11 |
| TF | Factor: FOXP1; motif: CTGTTTNYTYTKN | TF:M09855 | 6424 | 5.50E-08 | 372 | 121 |
| TF | Factor: SMAD; motif: TGTCTGNNN | TF:M08897 | 3891 | 5.60E-08 | 372 | 86 |
| TF | Factor: NFATc4; motif: NGGAAAAN | TF:M07301 | 3354 | 5.70E-08 | 372 | 78 |
| GO:BP | mononuclear cell migration | GO:0071674 | 165 | 5.90E-08 | 372 | 16 |
| TF | Factor: C/EBPbeta; motif: TKNNGCAANN | TF:M01896 | 3827 | 6.00E-08 | 372 | 85 |
| TF | Factor: Elf-1; motif: RNWMBAGGAART | TF:M00746 | 2905 | 6.20E-08 | 372 | 71 |
| GO:BP | negative regulation of response to external stimulus | GO:0032102 | 349 | 6.40E-08 | 372 | 22 |
| GO:MF | sialic acid binding | GO:0033691 | 16 | 6.50E-08 | 372 | 7 |
| TF | Factor: NFATc1; motif: NTTTCCRTNNAYGGAAAN | TF:M11979 | 7686 | 6.50E-08 | 372 | 137 |
| KEGG | Osteoclast differentiation | KEGG:04380 | 124 | 6.60E-08 | 372 | 13 |
| GO:BP | secretion | GO:0046903 | 843 | 6.90E-08 | 372 | 34 |
| TF | Factor: Spi-B; motif: NAWGRGGAAGTR | TF:M11374 | 2170 | 6.90E-08 | 372 | 59 |
| TF | Factor: VDR,; motif: RRTGNMCYTNNTGAMCCNYNT | TF:M00966 | 8419 | 7.20E-08 | 372 | 146 |
| GO:BP | microglial cell activation | GO:0001774 | 45 | 7.40E-08 | 372 | 10 |
| GO:BP | humoral immune response mediated by circulating immunoglobulin | GO:0002455 | 45 | 7.40E-08 | 372 | 10 |
| TF | Factor: p53; motif: NGRCWTGYCY | TF:M00272 | 3572 | 7.40E-08 | 372 | 81 |
| TF | Factor: Erm; motif: NRRSAGGAARNGRN | TF:M09916 | 5923 | 7.40E-08 | 372 | 114 |
| GO:BP | lymphocyte differentiation | GO:0030098 | 353 | 7.90E-08 | 372 | 22 |
| TF | Factor: NKX25; motif: NNANCCACTTRAAWTT | TF:M01414 | 8271 | 8.20E-08 | 372 | 144 |
| TF | Factor: NFATc2; motif: TTTTCCA | TF:M02265 | 6464 | 8.30E-08 | 372 | 121 |
| TF | Factor: TEF-1; motif: GRRATG | TF:M00704 | 10603 | 8.50E-08 | 372 | 172 |
| TF | Factor: HMGIY; motif: NNKKNAWTTTNYTNN | TF:M01010 | 6852 | 8.60E-08 | 372 | 126 |
| GO:BP | positive regulation of phagocytosis | GO:0050766 | 61 | 8.70E-08 | 372 | 11 |
| TF | Factor: Sox-9; motif: NNNNAACAATRGNN | TF:M00410 | 3323 | 9.40E-08 | 372 | 77 |
| GO:BP | neutrophil activation | GO:0042119 | 33 | 9.70E-08 | 372 | 9 |
| GO:BP | complement activation, classical pathway | GO:0006958 | 33 | 9.70E-08 | 372 | 9 |
| REAC | GPCR ligand binding | REAC:R-HSA-500792 | 388 | 1.00E-07 | 372 | 22 |
| TF | Factor: IRF-2; motif: NAANYGAAASYR | TF:M08775 | 7180 | 1.00E-07 | 372 | 130 |
| TF | Factor: FOXO1A:ETV7; motif: NWMAACAGGAMNNNCTTCCNN | TF:M08304 | 12194 | 1.00E-07 | 372 | 190 |
| GO:BP | regulation of leukocyte differentiation | GO:1902105 | 259 | 1.10E-07 | 372 | 19 |
| GO:MF | immunoglobulin binding | GO:0019865 | 17 | 1.10E-07 | 372 | 7 |
| TF | Factor: T3R-beta; motif: RTGACCTYACRTGACCTYA | TF:M11819 | 5885 | 1.10E-07 | 372 | 113 |
| TF | Factor: ZNF436; motif: TCCTCCAGGAAGCCY | TF:M10477 | 12825 | 1.10E-07 | 372 | 197 |
| GO:BP | antigen processing and presentation of peptide or polysaccharide antigen via MHC class II | GO:0002504 | 34 | 1.30E-07 | 372 | 9 |
| TF | Factor: AP-4; motif: AHCATRTGKT; match class: 1 | TF:M11213_1 | 2446 | 1.30E-07 | 372 | 63 |
| TF | Factor: ER71:E2A; motif: CASSTGNACCGGAWRYN | TF:M08568 | 15994 | 1.30E-07 | 372 | 231 |
| TF | Factor: ZABC1; motif: ATTCCNAC | TF:M01306 | 8006 | 1.40E-07 | 372 | 140 |
| TF | Factor: CRX; motif: YTAATC | TF:M01712 | 9480 | 1.40E-07 | 372 | 158 |
| TF | Factor: GLI4; motif: RGGCCTTGAATGCCANGCYMA | TF:M10514 | 11095 | 1.40E-07 | 372 | 177 |
| TF | Factor: ZNF644; motif: TCCWGCCTCTSN | TF:M09737 | 6371 | 1.50E-07 | 372 | 119 |
| GO:BP | regulation of metabolic process | GO:0019222 | 6103 | 1.60E-07 | 372 | 115 |
| TF | Factor: rfx3:SREBP-2; motif: ATGGYAACRTCACGTGAY | TF:M08412 | 9163 | 1.60E-07 | 372 | 154 |
| TF | Factor: C/EBPgamma:Elf-1; motif: TKRCGHAATWSCGGAAGT | TF:M08676 | 7465 | 1.60E-07 | 372 | 133 |
| TF | Factor: GR; motif: CNNNNTGTYCTNN | TF:M01836 | 4890 | 1.60E-07 | 372 | 99 |
| TF | Factor: Cdx-1; motif: TTTATK; match class: 1 | TF:M02086_1 | 8108 | 1.70E-07 | 372 | 141 |
| KEGG | Asthma | KEGG:05310 | 22 | 1.80E-07 | 372 | 7 |
| GO:BP | regulation of T cell proliferation | GO:0042129 | 152 | 1.90E-07 | 372 | 15 |
| TF | Factor: Smad2; motif: TGTCTGNCWCCT | TF:M10059 | 5342 | 1.90E-07 | 372 | 105 |
| GO:BP | cellular response to organic substance | GO:0071310 | 2188 | 2.00E-07 | 372 | 58 |
| TF | Factor: TFAP4; motif: AWCAGCTGWT | TF:M04192 | 4548 | 2.00E-07 | 372 | 94 |
| TF | Factor: NFATc2; motif: GGAAAA; match class: 1 | TF:M03555_1 | 9439 | 2.00E-07 | 372 | 157 |
| TF | Factor: NFATc3; motif: GGAAAA; match class: 1 | TF:M01886_1 | 9439 | 2.00E-07 | 372 | 157 |
| TF | Factor: NFATc2; motif: GGAAAA; match class: 1 | TF:M01281_1 | 9439 | 2.00E-07 | 372 | 157 |
| GO:BP | positive regulation of leukocyte migration | GO:0002687 | 128 | 2.10E-07 | 372 | 14 |
| TF | Factor: c-Jun; motif: NATGACTCATN | TF:M11265 | 3855 | 2.10E-07 | 372 | 84 |
| GO:BP | positive regulation of molecular function | GO:0044093 | 1394 | 2.40E-07 | 372 | 44 |
| TF | Factor: LBP-1; motif: CAGCTGS | TF:M00644 | 7824 | 2.40E-07 | 372 | 137 |
| TF | Factor: Smad4; motif: NCAGACAN | TF:M07368 | 3322 | 2.40E-07 | 372 | 76 |
| GO:BP | positive regulation of catalytic activity | GO:0043085 | 1031 | 2.50E-07 | 372 | 37 |
| TF | Factor: TTF1; motif: ASTCAAGTRK | TF:M00432 | 4425 | 2.50E-07 | 372 | 92 |
| TF | Factor: CPBP; motif: SNCCCNN; match class: 1 | TF:M01822_1 | 16464 | 2.50E-07 | 372 | 235 |
| TF | Factor: C/EBPalpha; motif: NRTTGTGCAAYNN | TF:M09596 | 4356 | 2.50E-07 | 372 | 91 |
| TF | Factor: Tbx3; motif: NAGGTGTSAN | TF:M12024 | 3800 | 2.60E-07 | 372 | 83 |
| TF | Factor: ITF-2; motif: NNCACCTGNN | TF:M04191 | 4572 | 2.60E-07 | 372 | 94 |
| TF | Factor: B-ATF; motif: NNATGACACN | TF:M11300 | 7440 | 2.70E-07 | 372 | 132 |
| GO:BP | negative regulation of response to stimulus | GO:0048585 | 1510 | 2.80E-07 | 372 | 46 |
| TF | Factor: PPARgamma:RXRalpha,; motif: AAGTAGGTCACNGTGACCYACTT | TF:M00515 | 6738 | 2.80E-07 | 372 | 123 |
| TF | Factor: HOXA13; motif: ATAAMA | TF:M01292 | 11353 | 2.80E-07 | 372 | 179 |
| TF | Factor: SPI1; motif: NGRGGAAGTN | TF:M02078 | 3073 | 2.90E-07 | 372 | 72 |
| GO:BP | positive regulation of metabolic process | GO:0009893 | 3369 | 3.20E-07 | 372 | 76 |
| TF | Factor: ZNF342; motif: TRSTGGACRNT | TF:M12075 | 5465 | 3.30E-07 | 372 | 106 |
| TF | Factor: Elk-1; motif: GGAAGN; match class: 1 | TF:M03819_1 | 6527 | 3.40E-07 | 372 | 120 |
| GO:BP | regulation of cell communication | GO:0010646 | 3111 | 3.50E-07 | 372 | 72 |
| GO:MF | G protein-coupled receptor activity | GO:0004930 | 495 | 3.50E-07 | 372 | 24 |
| TF | Factor: ZNF684; motif: MAAGGGGTGGACTGT | TF:M10550 | 13426 | 3.50E-07 | 372 | 202 |
| REAC | Class A/1 (Rhodopsin-like receptors) | REAC:R-HSA-373076 | 272 | 3.90E-07 | 372 | 18 |
| TF | Factor: Ikaros; motif: TGGGAGN | TF:M07260 | 11832 | 3.90E-07 | 372 | 184 |
| GO:BP | regulation of inflammatory response | GO:0050727 | 313 | 4.00E-07 | 372 | 20 |
| TF | Factor: AP-4:Max; motif: NCAGCTGNNNNNNNCACGTGN | TF:M08674 | 10025 | 4.00E-07 | 372 | 163 |
| TF | Factor: SMAD3; motif: CAGACAS; match class: 1 | TF:M07429_1 | 1137 | 4.00E-07 | 372 | 39 |
| TF | Factor: NR3C1; motif: NRGWACAYNRTGTWCYN | TF:M04476 | 6162 | 4.10E-07 | 372 | 115 |
| TF | Factor: RXR:RAR; motif: RGKTCANNNRGAGGTCA | TF:M02272 | 5486 | 4.10E-07 | 372 | 106 |
| GO:BP | regulation of signaling | GO:0023051 | 3125 | 4.30E-07 | 372 | 72 |
| TF | Factor: Ets; motif: ACTTCCTS | TF:M00971 | 2036 | 4.60E-07 | 372 | 55 |
| TF | Factor: MZF-1; motif: TGGGGAR | TF:M01733 | 10043 | 4.70E-07 | 372 | 163 |
| GO:BP | cell death | GO:0008219 | 1881 | 4.90E-07 | 372 | 52 |
| GO:BP | granulocyte activation | GO:0036230 | 39 | 5.10E-07 | 372 | 9 |
| GO:BP | toll-like receptor signaling pathway | GO:0002224 | 113 | 5.10E-07 | 372 | 13 |
| TF | Factor: NFATc1; motif: TTTCCAYWRTGGAAA | TF:M04052 | 5658 | 5.30E-07 | 372 | 108 |
| TF | Factor: gsh-1; motif: NTMATNRN | TF:M10750 | 6650 | 5.30E-07 | 372 | 121 |
| TF | Factor: TR4; motif: ACCCCGS | TF:M04934 | 15997 | 5.40E-07 | 372 | 229 |
| TF | Factor: AP-4; motif: RNCAGCTGC | TF:M00927 | 5587 | 5.50E-07 | 372 | 107 |
| GO:BP | antigen processing and presentation of exogenous peptide antigen via MHC class II | GO:0019886 | 27 | 5.70E-07 | 372 | 8 |
| TF | Factor: Oct-2; motif: NTGCATATGCAN; match class: 1 | TF:M11899_1 | 3591 | 5.90E-07 | 372 | 79 |
| TF | Factor: SRY; motif: AACAATANCATTGTT; match class: 1 | TF:M04555_1 | 2669 | 6.00E-07 | 372 | 65 |
| TF | Factor: AR; motif: GGAACGGWACATGTTCT | TF:M08190 | 6740 | 6.00E-07 | 372 | 122 |
| TF | Factor: ipf1; motif: TGATTGATK | TF:M10031 | 7448 | 6.10E-07 | 372 | 131 |
| GO:BP | negative regulation of immune response | GO:0050777 | 165 | 6.20E-07 | 372 | 15 |
| GO:BP | positive regulation of interleukin-8 production | GO:0032757 | 55 | 6.20E-07 | 372 | 10 |
| GO:BP | lipopolysaccharide-mediated signaling pathway | GO:0031663 | 55 | 6.20E-07 | 372 | 10 |
| GO:BP | secretion by cell | GO:0032940 | 728 | 6.30E-07 | 372 | 30 |
| TF | Factor: c-Maf; motif: NWNNNTGCTGACKNNNNNN | TF:M01070 | 5525 | 6.30E-07 | 372 | 106 |
| TF | Factor: ERF:FOXO1A; motif: RTMAACAGGAARNS | TF:M08241 | 6745 | 6.30E-07 | 372 | 122 |
| TF | Factor: GTF2IRD1-isoform2; motif: GGGATTRNR; match class: 1 | TF:M01229_1 | 7936 | 6.60E-07 | 372 | 137 |
| GO:CC | specific granule membrane | GO:0035579 | 86 | 7.00E-07 | 372 | 11 |
| TF | Factor: T3R-beta; motif: NRGGTCAAAGGTCRN | TF:M11814 | 12177 | 7.20E-07 | 372 | 187 |
| TF | Factor: AP-4; motif: ANCATATGNT; match class: 1 | TF:M11209_1 | 2183 | 7.40E-07 | 372 | 57 |
| KEGG | B cell receptor signaling pathway | KEGG:04662 | 77 | 7.50E-07 | 372 | 10 |
| TF | Factor: ARP-1; motif: TGARCCYTTGAMCCCW | TF:M00155 | 4662 | 7.50E-07 | 372 | 94 |
| GO:BP | negative regulation of lymphocyte activation | GO:0051250 | 141 | 7.60E-07 | 372 | 14 |
| GO:CC | MHC protein complex | GO:0042611 | 22 | 7.70E-07 | 372 | 7 |
| GO:BP | cell motility | GO:0048870 | 1504 | 7.90E-07 | 372 | 45 |
| GO:BP | localization of cell | GO:0051674 | 1504 | 7.90E-07 | 372 | 45 |
| TF | Factor: Sox-18; motif: NACAAYGS | TF:M11623 | 4529 | 8.50E-07 | 372 | 92 |
| TF | Factor: c-Ets-1; motif: RCAGGAAGTGNNTNS; match class: 1 | TF:M00339_1 | 1273 | 8.60E-07 | 372 | 41 |
| GO:BP | regulation of secretion | GO:0051046 | 562 | 9.00E-07 | 372 | 26 |
| GO:BP | interferon-gamma production | GO:0032609 | 96 | 9.60E-07 | 372 | 12 |
| TF | Factor: NFATc2; motif: NTTTCCATNNATGGAAAN | TF:M11986 | 5194 | 9.60E-07 | 372 | 101 |
| GO:BP | G protein-coupled receptor signaling pathway | GO:0007186 | 836 | 9.90E-07 | 372 | 32 |
| TF | Factor: Nkx3-2; motif: TRAGTG | TF:M01181 | 8070 | 1.10E-06 | 372 | 138 |
| TF | Factor: c-Ets-2; motif: NNCTTCCTNNN | TF:M07379 | 2966 | 1.10E-06 | 372 | 69 |
| TF | Factor: STAT3; motif: NNTTCCRGGAANNNNN | TF:M01595 | 6729 | 1.10E-06 | 372 | 121 |
| GO:BP | T cell activation involved in immune response | GO:0002286 | 98 | 1.20E-06 | 372 | 12 |
| GO:CC | organelle membrane | GO:0031090 | 3469 | 1.20E-06 | 372 | 74 |
| GO:MF | signaling receptor binding | GO:0005102 | 1265 | 1.20E-06 | 372 | 39 |
| TF | Factor: TFII-I; motif: NAGGAAGTGN; match class: 1 | TF:M04636_1 | 4849 | 1.20E-06 | 372 | 96 |
| TF | Factor: FOXJ2; motif: NNTGTTGTAAAYAN | TF:M11584 | 3923 | 1.20E-06 | 372 | 83 |
| TF | Factor: ZNF333; motif: ATAAT; match class: 1 | TF:M01230_1 | 9656 | 1.20E-06 | 372 | 157 |
| GO:BP | phagocytosis, engulfment | GO:0006911 | 59 | 1.30E-06 | 372 | 10 |
| GO:MF | MHC class II protein complex binding | GO:0023026 | 23 | 1.30E-06 | 372 | 7 |
| TF | Factor: c-Myb; motif: NNWGRCAGTTRN | TF:M09990 | 9243 | 1.30E-06 | 372 | 152 |
| TF | Factor: MYB; motif: NAACNGNCN | TF:M00913 | 4422 | 1.30E-06 | 372 | 90 |
| TF | Factor: Sox-10; motif: MAANRRNNNCWTTGTT; match class: 1 | TF:M10065_1 | 1512 | 1.30E-06 | 372 | 45 |
| GO:BP | regulation of B cell activation | GO:0050864 | 122 | 1.40E-06 | 372 | 13 |
| TF | Factor: JunD; motif: NATGASTCATS | TF:M11268 | 2342 | 1.40E-06 | 372 | 59 |
| TF | Factor: HNRPUL1; motif: NCNCAGN | TF:M09746 | 12084 | 1.40E-06 | 372 | 185 |
| TF | Factor: NR1B2; motif: NTGACCY | TF:M02111 | 8513 | 1.40E-06 | 372 | 143 |
| TF | Factor: sin3A; motif: TGTCCNNGGTGCTG; match class: 1 | TF:M04756_1 | 6912 | 1.50E-06 | 372 | 123 |
| GO:BP | negative regulation of leukocyte proliferation | GO:0070664 | 79 | 1.60E-06 | 372 | 11 |
| GO:BP | positive regulation of intracellular signal transduction | GO:1902533 | 902 | 1.60E-06 | 372 | 33 |
| TF | Factor: NFATc1; motif: NTTTCCRTNNAYGGAAAN | TF:M11981 | 5462 | 1.60E-06 | 372 | 104 |
| TF | Factor: NFATc1; motif: NTTTCCRTNNAYGGAAAN; match class: 1 | TF:M11981_1 | 4945 | 1.60E-06 | 372 | 97 |
| TF | Factor: RORbeta; motif: TGACCYA | TF:M01722 | 5168 | 1.60E-06 | 372 | 100 |
| TF | Factor: Six-3; motif: NNGTATCRNN | TF:M11034 | 3061 | 1.60E-06 | 372 | 70 |
| TF | Factor: Oct3; motif: CYWTTSWNATGCAAAT | TF:M10036 | 3465 | 1.70E-06 | 372 | 76 |
| TF | Factor: T-bet; motif: NNGNRGGTGTGRN | TF:M10089 | 5395 | 1.70E-06 | 372 | 103 |
| TF | Factor: c-MAF; motif: CNNNCTCAGCA | TF:M03816 | 4956 | 1.80E-06 | 372 | 97 |
| TF | Factor: MyoD; motif: CGNCAGCTGTYN | TF:M11187 | 1698 | 1.80E-06 | 372 | 48 |
| KEGG | Graft-versus-host disease | KEGG:05332 | 30 | 1.90E-06 | 372 | 7 |
| GO:BP | detection of external biotic stimulus | GO:0098581 | 20 | 2.00E-06 | 372 | 7 |
| TF | Factor: Sox-10; motif: AACAATRGNCYATTGTT | TF:M11632 | 4174 | 2.00E-06 | 372 | 86 |
| TF | Factor: CART1; motif: NGNNYTAATTARTNNNN | TF:M01362 | 3756 | 2.00E-06 | 372 | 80 |
| GO:BP | positive regulation of cell population proliferation | GO:0008284 | 813 | 2.10E-06 | 372 | 31 |
| TF | Factor: p53; motif: RGRCAWGNCY | TF:M00761 | 2560 | 2.10E-06 | 372 | 62 |
| TF | Factor: PMX1; motif: TAATHA; match class: 1 | TF:M03560_1 | 8561 | 2.20E-06 | 372 | 143 |
| TF | Factor: SMAD5; motif: GSGGCAGM | TF:M03846 | 7589 | 2.20E-06 | 372 | 131 |
| TF | Factor: C/EBPbeta; motif: RTTGCGCAA; match class: 1 | TF:M04690_1 | 3216 | 2.20E-06 | 372 | 72 |
| TF | Factor: beta-catenin; motif: GDMATCAAAGM | TF:M03539 | 3217 | 2.20E-06 | 372 | 72 |
| TF | Factor: six4; motif: NCGTATCRTN | TF:M11024 | 4469 | 2.20E-06 | 372 | 90 |
| TF | Factor: Rarb; motif: AGGTCANNYARAGGTCA | TF:M08035 | 4979 | 2.30E-06 | 372 | 97 |
| TF | Factor: AP-4; motif: ANCATATGNT; match class: 1 | TF:M11207_1 | 1827 | 2.30E-06 | 372 | 50 |
| REAC | Regulation of Complement cascade | REAC:R-HSA-977606 | 38 | 2.40E-06 | 372 | 8 |
| GO:BP | regulation of neutrophil activation | GO:1902563 | 12 | 2.50E-06 | 372 | 6 |
| GO:CC | lysosome | GO:0005764 | 688 | 2.50E-06 | 372 | 27 |
| GO:CC | lytic vacuole | GO:0000323 | 688 | 2.50E-06 | 372 | 27 |
| KEGG | Allograft rejection | KEGG:05330 | 31 | 2.50E-06 | 372 | 7 |
| TF | Factor: Sox-10; motif: CWTTGT | TF:M03138 | 11286 | 2.50E-06 | 372 | 175 |
| TF | Factor: TCF-1; motif: CTTTGW | TF:M03857 | 11286 | 2.50E-06 | 372 | 175 |
| TF | Factor: Fli-1; motif: RCAGGAAGTGR | TF:M07089 | 4264 | 2.50E-06 | 372 | 87 |
| GO:BP | respiratory burst | GO:0045730 | 32 | 2.60E-06 | 372 | 8 |
| TF | Factor: GR; motif: NNNNNNCNNTNTGTNCTNN | TF:M00192 | 6349 | 2.60E-06 | 372 | 115 |
| TF | Factor: CDP:HOXA13; motif: ATCGATYAYSYCRTAAA | TF:M08201 | 6582 | 2.60E-06 | 372 | 118 |
| TF | Factor: egr-3; motif: GTGGGY; match class: 1 | TF:M03818_1 | 5816 | 2.70E-06 | 372 | 108 |
| GO:BP | regulation of mononuclear cell migration | GO:0071675 | 105 | 2.80E-06 | 372 | 12 |
| GO:CC | membrane raft | GO:0045121 | 312 | 2.90E-06 | 372 | 18 |
| GO:BP | cell junction disassembly | GO:0150146 | 21 | 3.00E-06 | 372 | 7 |
| GO:CC | membrane microdomain | GO:0098857 | 313 | 3.00E-06 | 372 | 18 |
| TF | Factor: GR; motif: RGNACANKNTGTNCY | TF:M09625 | 7867 | 3.00E-06 | 372 | 134 |
| TF | Factor: Oct-2; motif: NTATGCWAATN | TF:M11896 | 4354 | 3.10E-06 | 372 | 88 |
| TF | Factor: SRY; motif: AAACWAM | TF:M00148 | 7951 | 3.10E-06 | 372 | 135 |
| TF | Factor: ETSLIKE; motif: NACTTCCTNN | TF:M08881 | 2714 | 3.10E-06 | 372 | 64 |
| GO:BP | export from cell | GO:0140352 | 780 | 3.20E-06 | 372 | 30 |
| TF | Factor: CSX; motif: TSYCACTTSM | TF:M01043 | 5382 | 3.20E-06 | 372 | 102 |
| GO:BP | regulation of anatomical structure morphogenesis | GO:0022603 | 878 | 3.30E-06 | 372 | 32 |
| TF | Factor: IRF-4; motif: RGGAASWGR | TF:M04818 | 8202 | 3.30E-06 | 372 | 138 |
| TF | Factor: MEL1; motif: GARGAT | TF:M08794 | 9275 | 3.30E-06 | 372 | 151 |
| TF | Factor: ER-beta; motif: GTCANASTGRCCYNR | TF:M01875 | 5536 | 3.30E-06 | 372 | 104 |
| GO:BP | positive regulation of cell differentiation | GO:0045597 | 782 | 3.40E-06 | 372 | 30 |
| GO:BP | endocytosis | GO:0006897 | 555 | 3.40E-06 | 372 | 25 |
| TF | Factor: SRY; motif: AACAATNNNCATTGTT | TF:M04556 | 6458 | 3.50E-06 | 372 | 116 |
| GO:BP | mast cell degranulation | GO:0043303 | 48 | 3.70E-06 | 372 | 9 |
| GO:BP | regulation of signal transduction | GO:0009966 | 2751 | 3.70E-06 | 372 | 64 |
| GO:BP | intracellular signal transduction | GO:0035556 | 2497 | 3.90E-06 | 372 | 60 |
| GO:MF | cargo receptor activity | GO:0038024 | 77 | 3.90E-06 | 372 | 10 |
| TF | Factor: TEF; motif: ATGTTWAYATAA | TF:M00672 | 4809 | 3.90E-06 | 372 | 94 |
| TF | Factor: BRCA2; motif: TGMCYCWGN | TF:M12723 | 3259 | 3.90E-06 | 372 | 72 |
| TF | Factor: Msx-2; motif: NNNGACYAATTAGYNNT; match class: 1 | TF:M01393_1 | 3193 | 3.90E-06 | 372 | 71 |
| GO:BP | regulation of interleukin-6 production | GO:0032675 | 133 | 4.00E-06 | 372 | 13 |
| GO:BP | interleukin-6 production | GO:0032635 | 133 | 4.00E-06 | 372 | 13 |
| GO:BP | regulation of toll-like receptor signaling pathway | GO:0034121 | 66 | 4.00E-06 | 372 | 10 |
| TF | Factor: ESRRA; motif: CAAGGTCANNYSAAGGTCA | TF:M04458 | 9722 | 4.10E-06 | 372 | 156 |
| TF | Factor: CDP; motif: NATYGATSSS | TF:M00106 | 5335 | 4.20E-06 | 372 | 101 |
| TF | Factor: rfx3:SRF; motif: TRGCAACNNNNNCCNWATANGGN | TF:M08623 | 12775 | 4.20E-06 | 372 | 191 |
| GO:BP | regulation of protein metabolic process | GO:0051246 | 2316 | 4.50E-06 | 372 | 57 |
| GO:BP | positive regulation of interleukin-6 production | GO:0032755 | 87 | 4.50E-06 | 372 | 11 |
| TF | Factor: IRX5; motif: NWACAYRACAWN | TF:M04378 | 3270 | 4.50E-06 | 372 | 72 |
| GO:BP | MHC class II protein complex assembly | GO:0002399 | 13 | 4.60E-06 | 372 | 6 |
| GO:BP | peptide antigen assembly with MHC class II protein complex | GO:0002503 | 13 | 4.60E-06 | 372 | 6 |
| TF | Factor: TEAD1; motif: NNACATTCCAGNN | TF:M12500 | 5045 | 4.60E-06 | 372 | 97 |
| TF | Factor: HOXD4; motif: RTCRTTAN | TF:M10710 | 2741 | 4.60E-06 | 372 | 64 |
| TF | Factor: c-Myc; motif: NCCACGTGCNN | TF:M09992 | 4899 | 4.60E-06 | 372 | 95 |
| GO:BP | plasma membrane invagination | GO:0099024 | 67 | 4.70E-06 | 372 | 10 |
| GO:BP | immunoglobulin mediated immune response | GO:0016064 | 110 | 4.70E-06 | 372 | 12 |
| GO:BP | regulation of leukocyte chemotaxis | GO:0002688 | 110 | 4.70E-06 | 372 | 12 |
| TF | Factor: Lyl-1; motif: NCAKCTGYTNYCNN; match class: 1 | TF:M09977_1 | 6570 | 4.80E-06 | 372 | 117 |
| TF | Factor: C/EBPbeta; motif: NKNTTGCNYAAYNN | TF:M00117 | 3480 | 4.80E-06 | 372 | 75 |
| TF | Factor: SREBP-2; motif: RTGGGGTGAY | TF:M07406 | 5054 | 5.00E-06 | 372 | 97 |
| TF | Factor: C/EBP; motif: NTTRCNNAANNN | TF:M00912 | 4188 | 5.30E-06 | 372 | 85 |
| GO:BP | mast cell activation involved in immune response | GO:0002279 | 50 | 5.40E-06 | 372 | 9 |
| GO:BP | complement activation | GO:0006956 | 50 | 5.40E-06 | 372 | 9 |
| GO:BP | mast cell mediated immunity | GO:0002448 | 50 | 5.40E-06 | 372 | 9 |
| GO:BP | regulation of cell migration | GO:0030334 | 847 | 5.40E-06 | 372 | 31 |
| REAC | Signaling by GPCR | REAC:R-HSA-372790 | 615 | 5.50E-06 | 372 | 25 |
| TF | Factor: GSX2; motif: NNYMATTANN | TF:M04328 | 5063 | 5.50E-06 | 372 | 97 |
| TF | Factor: C-Jun; motif: TGACTC; match class: 1 | TF:M03541_1 | 3560 | 5.50E-06 | 372 | 76 |
| TF | Factor: NFATc2; motif: NTTTCCATNNATGGAAAN; match class: 1 | TF:M11986_1 | 4990 | 5.60E-06 | 372 | 96 |
| TF | Factor: SAP-1a; motif: CWTCCKGT | TF:M03844 | 2306 | 5.60E-06 | 372 | 57 |
| TF | Factor: ER71; motif: CWTCCTGT | TF:M07282 | 2306 | 5.60E-06 | 372 | 57 |
| TF | Factor: Oct-2; motif: NYATGCAAATN | TF:M10034 | 5065 | 5.60E-06 | 372 | 97 |
| TF | Factor: CDP:SRF; motif: NCCWTAYAAGGTMNKRATCRATN | TF:M08520 | 8843 | 5.70E-06 | 372 | 145 |
| TF | Factor: PPARgamma:RXRalpha,; motif: AAGTAGGTCACNGTGACCYACTT; match class: 1 | TF:M00515_1 | 3841 | 5.70E-06 | 372 | 80 |
| GO:BP | B cell mediated immunity | GO:0019724 | 112 | 5.80E-06 | 372 | 12 |
| TF | Factor: Spi-B; motif: NAWGCGGAAGTN | TF:M11375 | 2309 | 5.80E-06 | 372 | 57 |
| TF | Factor: Sox-10; motif: AACAATGGNCCATTGTT | TF:M11633 | 3089 | 5.90E-06 | 372 | 69 |
| TF | Factor: Thap1; motif: SCGCCATSTTKGNTNMGGGCARNN | TF:M04723 | 10537 | 5.90E-06 | 372 | 165 |
| KEGG | Inflammatory bowel disease | KEGG:05321 | 52 | 6.10E-06 | 372 | 8 |
| KEGG | Type I diabetes mellitus | KEGG:04940 | 35 | 6.10E-06 | 372 | 7 |
| GO:BP | regulation of cell death | GO:0010941 | 1440 | 6.80E-06 | 372 | 42 |
| TF | Factor: FOXO1A:PDEF; motif: WNCCGGATGTTDN; match class: 1 | TF:M08486_1 | 3785 | 6.80E-06 | 372 | 79 |
| GO:MF | IgG binding | GO:0019864 | 9 | 6.90E-06 | 372 | 5 |
| TF | Factor: Oct-2; motif: NTATGCGCATAN | TF:M11895 | 3303 | 6.90E-06 | 372 | 72 |
| TF | Factor: CSX; motif: NNCACTTGNRN | TF:M02108 | 5994 | 7.10E-06 | 372 | 109 |
| TF | Factor: Pax-5; motif: RRNGRNGCAN | TF:M03577 | 7727 | 7.20E-06 | 372 | 131 |
| TF | Factor: SATB1; motif: NTTTAT; match class: 1 | TF:M03564_1 | 10054 | 7.40E-06 | 372 | 159 |
| TF | Factor: Oct-4; motif: ATTGWSWTGCWAAWN | TF:M01124 | 4945 | 7.50E-06 | 372 | 95 |
| TF | Factor: MEF-2C; motif: TATTTWT; match class: 1 | TF:M02025_1 | 6386 | 7.50E-06 | 372 | 114 |
| TF | Factor: ZBTB44; motif: MACWGCAGS | TF:M12352 | 5619 | 7.60E-06 | 372 | 104 |
| GO:BP | programmed cell death | GO:0012501 | 1737 | 7.80E-06 | 372 | 47 |
| TF | Factor: GATA-3; motif: AGATAA | TF:M01878 | 8472 | 7.90E-06 | 372 | 140 |
| TF | Factor: Spi-B; motif: RAAWGRGGAAGTN | TF:M11376 | 613 | 8.00E-06 | 372 | 26 |
| TF | Factor: CTCF; motif: CANNWRRTGGCAG | TF:M04745 | 7022 | 8.10E-06 | 372 | 122 |
| REAC | Interferon gamma signaling | REAC:R-HSA-877300 | 83 | 8.20E-06 | 372 | 10 |
| TF | Factor: Bcl-6; motif: NTTYCTAGRA | TF:M02085 | 4154 | 8.20E-06 | 372 | 84 |
| TF | Factor: C/EBPalpha; motif: NNNTTNNGCAANN | TF:M01866 | 4299 | 8.30E-06 | 372 | 86 |
| GO:BP | regulated exocytosis | GO:0045055 | 231 | 8.60E-06 | 372 | 16 |
| TF | Factor: Tbx5; motif: NNAGGTGTNANN | TF:M01019 | 4960 | 8.80E-06 | 372 | 95 |
| GO:BP | positive regulation of phosphate metabolic process | GO:0045937 | 817 | 9.20E-06 | 372 | 30 |
| GO:BP | positive regulation of phosphorus metabolic process | GO:0010562 | 817 | 9.20E-06 | 372 | 30 |
| GO:CC | cytoplasm | GO:0005737 | 11064 | 9.20E-06 | 372 | 167 |
| TF | Factor: Spi-B; motif: AAAAWGMGGAAGTWNSN | TF:M01204 | 663 | 9.30E-06 | 372 | 27 |
| TF | Factor: HOXD4; motif: RTCRTTAN | TF:M10713 | 3124 | 9.40E-06 | 372 | 69 |
| KEGG | Legionellosis | KEGG:05134 | 55 | 9.60E-06 | 372 | 8 |
| TF | Factor: C-FOS; motif: NATGAGTCATN | TF:M08946 | 2153 | 9.60E-06 | 372 | 54 |
| GO:BP | lymphocyte mediated immunity | GO:0002449 | 233 | 9.70E-06 | 372 | 16 |
| TF | Factor: SRY; motif: TCAATAMCATTGA; match class: 1 | TF:M04557_1 | 3956 | 9.70E-06 | 372 | 81 |
| TF | Factor: PPARGAMMA:RXR-ALPHA; motif: NNRGGTCAAWAGGTCAN | TF:M08962 | 4100 | 9.90E-06 | 372 | 83 |
| REAC | Antimicrobial peptides | REAC:R-HSA-6803157 | 30 | 1.00E-05 | 372 | 7 |
| TF | Factor: Oct-2; motif: NTGCATATGCAN | TF:M11899 | 4532 | 1.00E-05 | 372 | 89 |
| TF | Factor: SMAD3; motif: TGTCTGTCT; match class: 1 | TF:M00701_1 | 575 | 1.00E-05 | 372 | 25 |
| TF | Factor: TBX2; motif: NNNGTGTSNN | TF:M12026 | 6418 | 1.00E-05 | 372 | 114 |
| TF | Factor: FOXO1A:Net; motif: RWMAACAGGAAGTN; match class: 1 | TF:M08302_1 | 2282 | 1.00E-05 | 372 | 56 |
| TF | Factor: Fra-1; motif: ACTCATG | TF:M04897 | 2096 | 1.00E-05 | 372 | 53 |
| TF | Factor: IRF-4; motif: NAANGRGGAASTGAAASN | TF:M09959 | 2865 | 1.00E-05 | 372 | 65 |
| TF | Factor: C/EBPbeta; motif: RTTGCGCAA | TF:M04690 | 3752 | 1.10E-05 | 372 | 78 |
| TF | Factor: ZNF549; motif: NTGCTKYCMW | TF:M12355 | 5656 | 1.10E-05 | 372 | 104 |
| TF | Factor: NF-E4; motif: GTGAGGS | TF:M08826 | 10022 | 1.10E-05 | 372 | 158 |
| GO:BP | regulation of interferon-gamma production | GO:0032649 | 95 | 1.20E-05 | 372 | 11 |
| GO:BP | positive regulation of leukocyte differentiation | GO:1902107 | 146 | 1.20E-05 | 372 | 13 |
| GO:BP | positive regulation of hemopoiesis | GO:1903708 | 146 | 1.20E-05 | 372 | 13 |
| GO:CC | clathrin-coated endocytic vesicle membrane | GO:0030669 | 66 | 1.20E-05 | 372 | 9 |
| MIRNA | hsa-miR-335-5p | MIRNA:hsa-miR-335-5p | 2271 | 1.20E-05 | 372 | 54 |
| REAC | Complement cascade | REAC:R-HSA-166658 | 46 | 1.20E-05 | 372 | 8 |
| TF | Factor: TCF-7; motif: TCAAAG | TF:M00805 | 10201 | 1.20E-05 | 372 | 160 |
| TF | Factor: TCF-3; motif: CTTTGA | TF:M03858 | 10201 | 1.20E-05 | 372 | 160 |
| TF | Factor: OLIG2; motif: CCAKCTGYTYNYNNNNNN | TF:M10019 | 3416 | 1.20E-05 | 372 | 73 |
| TF | Factor: Erg; motif: NACAGGAARTN | TF:M11390 | 2358 | 1.20E-05 | 372 | 57 |
| GO:BP | response to wounding | GO:0009611 | 505 | 1.30E-05 | 372 | 23 |
| GO:BP | negative regulation of lymphocyte proliferation | GO:0050672 | 74 | 1.30E-05 | 372 | 10 |
| GO:BP | membrane invagination | GO:0010324 | 74 | 1.30E-05 | 372 | 10 |
| GO:BP | negative regulation of mononuclear cell proliferation | GO:0032945 | 74 | 1.30E-05 | 372 | 10 |
| GO:BP | peptide antigen assembly with MHC protein complex | GO:0002501 | 15 | 1.30E-05 | 372 | 6 |
| GO:MF | MHC protein complex binding | GO:0023023 | 31 | 1.30E-05 | 372 | 7 |
| TF | Factor: RXRA; motif: RRGGTCATGACCYY | TF:M04490 | 6522 | 1.30E-05 | 372 | 115 |
| KEGG | Autoimmune thyroid disease | KEGG:05320 | 39 | 1.40E-05 | 372 | 7 |
| TF | Factor: MRF4; motif: CASCTGC | TF:M03831 | 7565 | 1.40E-05 | 372 | 128 |
| TF | Factor: HTF4; motif: CASCTGB | TF:M02018 | 7565 | 1.40E-05 | 372 | 128 |
| TF | Factor: MafB; motif: GNTGAC | TF:M01227 | 10227 | 1.40E-05 | 372 | 160 |
| GO:BP | regulation of neutrophil degranulation | GO:0043313 | 8 | 1.50E-05 | 372 | 5 |
| GO:BP | positive regulation of apoptotic cell clearance | GO:2000427 | 8 | 1.50E-05 | 372 | 5 |
| TF | Factor: POU2F1:Gscl; motif: NNGATTANNTATKCANNNN; match class: 1 | TF:M08404_1 | 1404 | 1.50E-05 | 372 | 41 |
| TF | Factor: myogenin; motif: CRSCTGTTBNNTTTGGCACGSNGCCARCH | TF:M00056 | 9551 | 1.50E-05 | 372 | 152 |
| GO:BP | response to lipid | GO:0033993 | 787 | 1.60E-05 | 372 | 29 |
| TF | Factor: JunD; motif: NRTGAGTCAYN | TF:M09964 | 4213 | 1.60E-05 | 372 | 84 |
| TF | Factor: RelA-p65; motif: BCWGGGRANNK | TF:M04811 | 6469 | 1.60E-05 | 372 | 114 |
| GO:BP | antigen processing and presentation of peptide antigen | GO:0048002 | 76 | 1.70E-05 | 372 | 10 |
| GO:BP | antigen receptor-mediated signaling pathway | GO:0050851 | 179 | 1.70E-05 | 372 | 14 |
| TF | Factor: DBP; motif: TTRCATAANN | TF:M07038 | 4582 | 1.70E-05 | 372 | 89 |
| TF | Factor: Sox-8; motif: NGAACAATRN | TF:M11629 | 1030 | 1.70E-05 | 372 | 34 |
| TF | Factor: TBP; motif: NGNNTATAAAA | TF:M03581 | 5782 | 1.70E-05 | 372 | 105 |
| GO:BP | cytokine-mediated signaling pathway | GO:0019221 | 430 | 1.80E-05 | 372 | 21 |
| TF | Factor: T3R-beta; motif: NRGGTCAAAGGTCAN | TF:M11816 | 10084 | 1.80E-05 | 372 | 158 |
| TF | Factor: msc; motif: NRMCATATGNYN | TF:M11146 | 2641 | 1.80E-05 | 372 | 61 |
| TF | Factor: Pax-4; motif: NNNNNYCACCCB; match class: 1 | TF:M00378_1 | 13163 | 1.80E-05 | 372 | 193 |
| TF | Factor: Sox-30; motif: NAACAATN | TF:M11641 | 1945 | 1.80E-05 | 372 | 50 |
| TF | Factor: KLF4; motif: MCACACCCTNN | TF:M09808 | 3175 | 1.80E-05 | 372 | 69 |
| TF | Factor: C/EBPgamma; motif: YTBATTTCARAAW | TF:M00622 | 5635 | 1.80E-05 | 372 | 103 |
| TF | Factor: znf136; motif: CAAGAATWCTATAYCCAG | TF:M10535 | 6878 | 1.80E-05 | 372 | 119 |
| KEGG | Transcriptional misregulation in cancer | KEGG:05202 | 166 | 1.90E-05 | 372 | 12 |
| TF | Factor: HSF4; motif: CTGCMRN; match class: 1 | TF:M07322_1 | 10436 | 1.90E-05 | 372 | 162 |
| TF | Factor: JunD; motif: SATGACTCATN | TF:M11269 | 3179 | 1.90E-05 | 372 | 69 |
| TF | Factor: CLOCK; motif: NNCAYGYGYN | TF:M11046 | 2134 | 1.90E-05 | 372 | 53 |
| TF | Factor: HOXA3; motif: NNNNRNTAATTARY; match class: 1 | TF:M01337_1 | 10095 | 1.90E-05 | 372 | 158 |
| GO:BP | regulation of cellular protein metabolic process | GO:0032268 | 2160 | 2.00E-05 | 372 | 53 |
| TF | Factor: B-ATF; motif: NNATGACACN; match class: 1 | TF:M11300_1 | 2135 | 2.00E-05 | 372 | 53 |
| GO:BP | MHC protein complex assembly | GO:0002396 | 16 | 2.10E-05 | 372 | 6 |
| GO:BP | regulation of cell motility | GO:2000145 | 898 | 2.10E-05 | 372 | 31 |
| TF | Factor: FOXO1A:Elf-1; motif: NAGAAAACCGAANM | TF:M08295 | 10193 | 2.10E-05 | 372 | 159 |
| TF | Factor: DRI1; motif: AATTAA | TF:M01654 | 9177 | 2.10E-05 | 372 | 147 |
| TF | Factor: Dlx-5; motif: AATTAN | TF:M03546 | 9177 | 2.10E-05 | 372 | 147 |
| REAC | G alpha (i) signalling events | REAC:R-HSA-418594 | 276 | 2.20E-05 | 372 | 16 |
| TF | Factor: Pit-1; motif: NMTTCATAAWTATWNMNA | TF:M00802 | 3743 | 2.20E-05 | 372 | 77 |
| TF | Factor: HMX2; motif: SCACTTANC | TF:M10646 | 6195 | 2.30E-05 | 372 | 110 |
| TF | Factor: foxm1; motif: TGTTTRCTYWNN | TF:M09930 | 5734 | 2.30E-05 | 372 | 104 |
| TF | Factor: LHX3; motif: ATTAAW | TF:M02097 | 10034 | 2.30E-05 | 372 | 157 |
| TF | Factor: GR; motif: NNTGTYCT | TF:M00921 | 1600 | 2.30E-05 | 372 | 44 |
| GO:BP | negative regulation of cytokine production | GO:0001818 | 248 | 2.40E-05 | 372 | 16 |
| GO:BP | positive regulation of macromolecule metabolic process | GO:0010604 | 3089 | 2.40E-05 | 372 | 67 |
| GO:BP | regulation of cell killing | GO:0031341 | 79 | 2.40E-05 | 372 | 10 |
| REAC | Hemostasis | REAC:R-HSA-109582 | 570 | 2.40E-05 | 372 | 23 |
| TF | Factor: Octamer; motif: TNATTTGCATN | TF:M00795 | 3752 | 2.40E-05 | 372 | 77 |
| TF | Factor: Oct-1; motif: NNNNNNNWATGCAAATNNNWNNA | TF:M00138 | 2024 | 2.40E-05 | 372 | 51 |
| GO:BP | regulation of macromolecule metabolic process | GO:0060255 | 5629 | 2.50E-05 | 372 | 102 |
| TF | Factor: HES-5; motif: NCACACKY; match class: 1 | TF:M11069_1 | 3616 | 2.50E-05 | 372 | 75 |
| GO:BP | complement-mediated synapse pruning | GO:0150062 | 4 | 2.60E-05 | 372 | 4 |
| TF | Factor: ELF3; motif: NNCCACTTCCTGNN | TF:M12544 | 3272 | 2.60E-05 | 372 | 70 |
| GO:BP | myeloid cell differentiation | GO:0030099 | 360 | 2.70E-05 | 372 | 19 |
| TF | Factor: C/EBPdelta; motif: TTGCNNMAN | TF:M03571 | 3137 | 2.70E-05 | 372 | 68 |
| TF | Factor: TEF-3; motif: GNTATTTTT | TF:M07270 | 8541 | 2.70E-05 | 372 | 139 |
| TF | Factor: Foxc1; motif: TTVYTTTNW | TF:M07254 | 6449 | 2.70E-05 | 372 | 113 |
| TF | Factor: B-ATF; motif: NNAYGACACN | TF:M11301 | 6607 | 2.70E-05 | 372 | 115 |
| TF | Factor: AP-2; motif: NNGCCTK | TF:M01859 | 6687 | 2.80E-05 | 372 | 116 |
| TF | Factor: Fli-1; motif: NNRGGMAGGAAGGRRRGR | TF:M09920 | 8216 | 2.80E-05 | 372 | 135 |
| TF | Factor: Six-2; motif: NNGTATCRNN | TF:M11030 | 5986 | 2.80E-05 | 372 | 107 |
| TF | Factor: NR1B1; motif: NRGGNCRTGACCTN | TF:M11796 | 9466 | 2.80E-05 | 372 | 150 |
| GO:MF | peptide binding | GO:0042277 | 282 | 2.90E-05 | 372 | 16 |
| KEGG | Coronavirus disease - COVID-19 | KEGG:05171 | 206 | 2.90E-05 | 372 | 13 |
| TF | Factor: FXR:RXR-ALPHA; motif: NRGGTCANRGGKN | TF:M08955 | 8470 | 2.90E-05 | 372 | 138 |
| TF | Factor: LXR;; motif: NTGACCKNNAGTRACCYNN | TF:M03795 | 5609 | 3.00E-05 | 372 | 102 |
| TF | Factor: ipf1; motif: CATTAR | TF:M01275 | 7333 | 3.00E-05 | 372 | 124 |
| GO:BP | regulation of phosphate metabolic process | GO:0019220 | 1289 | 3.10E-05 | 372 | 38 |
| TF | Factor: Oct-1; motif: NNNNATGCAAATNAN | TF:M00195 | 3014 | 3.10E-05 | 372 | 66 |
| GO:BP | regulation of phosphorus metabolic process | GO:0051174 | 1291 | 3.20E-05 | 372 | 38 |
| GO:BP | positive regulation of superoxide anion generation | GO:0032930 | 17 | 3.20E-05 | 372 | 6 |
| GO:BP | cellular defense response | GO:0006968 | 43 | 3.20E-05 | 372 | 8 |
| GO:BP | regulation of mast cell activation | GO:0033003 | 43 | 3.20E-05 | 372 | 8 |
| TF | Factor: IRF-4; motif: AAGTTTC | TF:M04855 | 6624 | 3.20E-05 | 372 | 115 |
| TF | Factor: Six-1; motif: NCGTATCRNN | TF:M11029 | 1980 | 3.20E-05 | 372 | 50 |
| TF | Factor: AR; motif: RGGWACAYNGTGTWCYN | TF:M04453 | 6079 | 3.20E-05 | 372 | 108 |
| TF | Factor: HOXB2; motif: RTCATTAN | TF:M10739 | 3153 | 3.20E-05 | 372 | 68 |
| GO:CC | vacuole | GO:0005773 | 781 | 3.30E-05 | 372 | 27 |
| TF | Factor: MGA; motif: AGGTGTGA | TF:M04117 | 1738 | 3.30E-05 | 372 | 46 |
| TF | Factor: TBX1; motif: AGGTGTGA | TF:M04126 | 1738 | 3.30E-05 | 372 | 46 |
| TF | Factor: Tbx5; motif: AGGTGTKA | TF:M04137 | 1738 | 3.30E-05 | 372 | 46 |
| TF | Factor: TBX15; motif: AGGTGTGA | TF:M04122 | 1738 | 3.30E-05 | 372 | 46 |
| TF | Factor: TBX4; motif: AGGTGTGA | TF:M04135 | 1738 | 3.30E-05 | 372 | 46 |
| TF | Factor: MEF-2D; motif: WAAATAR | TF:M02026 | 6393 | 3.30E-05 | 372 | 112 |
| TF | Factor: GCMa:C/EBPbeta; motif: ATGCGGGTNNRTTGCGCAAY | TF:M08575 | 5928 | 3.30E-05 | 372 | 106 |
| GO:BP | mast cell activation | GO:0045576 | 61 | 3.40E-05 | 372 | 9 |
| TF | Factor: Pax-4; motif: DTTTTCCACCN; match class: 1 | TF:M04624_1 | 8077 | 3.40E-05 | 372 | 133 |
| TF | Factor: NF-1C; motif: NYTGGCNNYNNGCCARN | TF:M10002 | 5930 | 3.40E-05 | 372 | 106 |
| GO:BP | regulation of biological quality | GO:0065008 | 3465 | 3.50E-05 | 372 | 72 |
| TF | Factor: SREBP-1; motif: CACSCCA; match class: 1 | TF:M00749_1 | 2428 | 3.50E-05 | 372 | 57 |
| TF | Factor: Ikaros; motif: TGGGAGN; match class: 1 | TF:M07260_1 | 6245 | 3.50E-05 | 372 | 110 |
| TF | Factor: C/EBPalpha; motif: NRTTGTGCAAYN | TF:M09880 | 2890 | 3.60E-05 | 372 | 64 |
| GO:BP | regulation of angiogenesis | GO:0045765 | 256 | 3.70E-05 | 372 | 16 |
| REAC | Cell surface interactions at the vascular wall | REAC:R-HSA-202733 | 123 | 3.70E-05 | 372 | 11 |
| TF | Factor: HELIOS; motif: RNARRRGGAASTGARAN | TF:M09745 | 2894 | 3.80E-05 | 372 | 64 |
| TF | Factor: TCF11:MafG; motif: NNNNNATGACTCAGCANTTNNG | TF:M00284 | 3651 | 3.80E-05 | 372 | 75 |
| TF | Factor: FOXO1A:HOXA10; motif: RWMAACANCRTWAA | TF:M08482 | 3442 | 3.80E-05 | 372 | 72 |
| GO:BP | positive regulation of defense response | GO:0031349 | 257 | 3.90E-05 | 372 | 16 |
| TF | Factor: Six-1; motif: NNGTATCRNN | TF:M11026 | 5183 | 3.90E-05 | 372 | 96 |
| TF | Factor: Zic3; motif: NGGGKGGTC | TF:M00450 | 12380 | 3.90E-05 | 372 | 183 |
| TF | Factor: LBX2; motif: CTNRANSTAATTA; match class: 1 | TF:M04383_1 | 1933 | 4.10E-05 | 372 | 49 |
| TF | Factor: FAC1; motif: NNNCAMAACACRNA | TF:M00456 | 6812 | 4.10E-05 | 372 | 117 |
| TF | Factor: msc; motif: NRMCATATGNYN; match class: 1 | TF:M11146_1 | 2505 | 4.20E-05 | 372 | 58 |
| TF | Factor: Dlx-3; motif: NTCRTTAN | TF:M10609 | 3800 | 4.20E-05 | 372 | 77 |
| TF | Factor: HOXB5; motif: RTCRTTAN | TF:M10698 | 3800 | 4.20E-05 | 372 | 77 |
| TF | Factor: nanog; motif: NYWTTGWNATGCAAATN | TF:M09639 | 1458 | 4.30E-05 | 372 | 41 |
| TF | Factor: Sox-10; motif: NACAAWG; match class: 1 | TF:M02116_1 | 6582 | 4.30E-05 | 372 | 114 |
| TF | Factor: POU2F1; motif: ATTTGCATN | TF:M07058 | 2707 | 4.50E-05 | 372 | 61 |
| TF | Factor: NFATC3; motif: WNTTTCCRNN | TF:M08001 | 1404 | 4.50E-05 | 372 | 40 |
| GO:BP | regulation of vasculature development | GO:1901342 | 260 | 4.60E-05 | 372 | 16 |
| GO:BP | pattern recognition receptor signaling pathway | GO:0002221 | 163 | 4.70E-05 | 372 | 13 |
| TF | Factor: c-Jun; motif: NATGASTCATN; match class: 1 | TF:M11264_1 | 3183 | 4.70E-05 | 372 | 68 |
| TF | Factor: SALL2; motif: GGGTGGG | TF:M04595 | 11697 | 4.70E-05 | 372 | 175 |
| TF | Factor: MAF; motif: NGCTGAGTCAN | TF:M00983 | 5128 | 4.70E-05 | 372 | 95 |
| TF | Factor: Six-3; motif: NNRTATCRNN | TF:M11035 | 1522 | 4.80E-05 | 372 | 42 |
| TF | Factor: AP-4; motif: AWCAGCTGWT | TF:M11210 | 3956 | 4.80E-05 | 372 | 79 |
| TF | Factor: AP-4; motif: AWCAGCTGWT; match class: 1 | TF:M11210_1 | 3956 | 4.80E-05 | 372 | 79 |
| GO:BP | negative regulation of inflammatory response | GO:0050728 | 135 | 4.90E-05 | 372 | 12 |
| TF | Factor: FOXJ2; motif: AYMATAATATTTKN | TF:M00423 | 5662 | 4.90E-05 | 372 | 102 |
| TF | Factor: Ngn-2; motif: RACATATGTY | TF:M11132 | 673 | 5.40E-05 | 372 | 26 |
| TF | Factor: Erg; motif: MCAGGAAA; match class: 1 | TF:M07284_1 | 3752 | 5.40E-05 | 372 | 76 |
| TF | Factor: ZBTB2; motif: TCCKCCTSR | TF:M07437 | 3263 | 5.40E-05 | 372 | 69 |
| GO:BP | regulation of locomotion | GO:0040012 | 937 | 5.60E-05 | 372 | 31 |
| TF | Factor: CTCF; motif: NNNNANASYGCCMYCTAGTGG | TF:M04689 | 4847 | 5.70E-05 | 372 | 91 |
| TF | Factor: SMAD4; motif: GKSRKKCAGMCANCY | TF:M00733 | 7655 | 5.70E-05 | 372 | 127 |
| TF | Factor: Tbx1; motif: NNAGGTGTGAAN | TF:M12035 | 2399 | 5.80E-05 | 372 | 56 |
| TF | Factor: OCT-x; motif: CTNATTTGCATAY | TF:M00210 | 3408 | 5.90E-05 | 372 | 71 |
| TF | Factor: Oct3; motif: NNTTATGYWAATKARN | TF:M11932 | 2470 | 6.40E-05 | 372 | 57 |
| TF | Factor: GATA-1; motif: NNNNNGATANKGGN | TF:M00126 | 3277 | 6.40E-05 | 372 | 69 |
| TF | Factor: TEF-1; motif: GRRATG; match class: 1 | TF:M00704_1 | 4564 | 6.50E-05 | 372 | 87 |
| TF | Factor: IRF-4; motif: GAAARTA; match class: 1 | TF:M01883_1 | 2342 | 6.50E-05 | 372 | 55 |
| TF | Factor: PRDM16; motif: KGGTCATRACCM | TF:M05946 | 6003 | 6.50E-05 | 372 | 106 |
| GO:BP | regulation of apoptotic cell clearance | GO:2000425 | 10 | 6.60E-05 | 372 | 5 |
| TF | Factor: Nrf-2; motif: NTGCTGAGTCAKN | TF:M00821 | 3984 | 6.60E-05 | 372 | 79 |
| GO:BP | exocytosis | GO:0006887 | 342 | 6.70E-05 | 372 | 18 |
| TF | Factor: FOXJ2; motif: NNTGTTGTAAAYAN | TF:M11585 | 4568 | 6.70E-05 | 372 | 87 |
| REAC | FCGR activation | REAC:R-HSA-2029481 | 13 | 6.80E-05 | 372 | 5 |
| GO:BP | positive regulation of myeloid leukocyte mediated immunity | GO:0002888 | 19 | 6.90E-05 | 372 | 6 |
| TF | Factor: AP-4; motif: AWCAGCTGWT | TF:M11208 | 3562 | 6.90E-05 | 372 | 73 |
| TF | Factor: TBX15; motif: NAGGTGTGAN | TF:M12040 | 2218 | 6.90E-05 | 372 | 53 |
| TF | Factor: GSX1; motif: NNYMATTANN; match class: 1 | TF:M04327_1 | 4571 | 6.90E-05 | 372 | 87 |
| GO:BP | regulation of interleukin-1 beta production | GO:0032651 | 88 | 7.00E-05 | 372 | 10 |
| GO:BP | interleukin-1 beta production | GO:0032611 | 88 | 7.00E-05 | 372 | 10 |
| TF | Factor: FOXO1A:Elk-1; motif: RWMAACAGGAAGTN; match class: 1 | TF:M08299_1 | 1312 | 7.00E-05 | 372 | 38 |
| TF | Factor: Blimp-1; motif: NACTTTCAC | TF:M04731 | 3425 | 7.10E-05 | 372 | 71 |
| TF | Factor: Msx-2; motif: TWWTTGGDGABN | TF:M03797 | 4137 | 7.20E-05 | 372 | 81 |
| TF | Factor: NeuroD-2; motif: RMCATATGKY | TF:M01287 | 2032 | 7.30E-05 | 372 | 50 |
| TF | Factor: Oct-2; motif: NNNATTATGCAW | TF:M11894 | 2946 | 7.30E-05 | 372 | 64 |
| GO:BP | positive regulation of hydrolase activity | GO:0051345 | 511 | 7.40E-05 | 372 | 22 |
| GO:BP | regulation of secretion by cell | GO:1903530 | 511 | 7.40E-05 | 372 | 22 |
| GO:BP | cellular response to lipid | GO:0071396 | 511 | 7.40E-05 | 372 | 22 |
| TF | Factor: SPIB; motif: NNTCACTTCCTCTTTN | TF:M12498 | 1908 | 7.40E-05 | 372 | 48 |
| TF | Factor: ATF-4; motif: GGATGATGTCATCC | TF:M11334 | 5176 | 7.50E-05 | 372 | 95 |
| TF | Factor: EVX1; motif: NTCATTAN | TF:M10864 | 4360 | 7.60E-05 | 372 | 84 |
| TF | Factor: GATA-3; motif: AGATAA; match class: 1 | TF:M01878_1 | 3293 | 7.80E-05 | 372 | 69 |
| GO:BP | regulation of mast cell degranulation | GO:0043304 | 32 | 7.90E-05 | 372 | 7 |
| GO:BP | detection of biotic stimulus | GO:0009595 | 32 | 7.90E-05 | 372 | 7 |
| TF | Factor: NF1C; motif: WGCCARR; match class: 1 | TF:M09763_1 | 3644 | 7.90E-05 | 372 | 74 |
| TF | Factor: Pax-2; motif: NNNNGTCANGNRTKANNNN | TF:M00098 | 6494 | 7.90E-05 | 372 | 112 |
| GO:BP | apoptotic process | GO:0006915 | 1692 | 8.20E-05 | 372 | 44 |
| TF | Factor: PU.1; motif: ACTTCCTCT | TF:M04711 | 1495 | 8.50E-05 | 372 | 41 |
| TF | Factor: Spic; motif: NGNGGAASTN; match class: 1 | TF:M02077_1 | 2042 | 8.50E-05 | 372 | 50 |
| GO:BP | cellular response to oxygen-containing compound | GO:1901701 | 1062 | 8.60E-05 | 372 | 33 |
| GO:CC | phagocytic vesicle | GO:0045335 | 136 | 8.70E-05 | 372 | 11 |
| TF | Factor: POU3F2; motif: WTATGCWAATKA | TF:M04079 | 2624 | 8.70E-05 | 372 | 59 |
| TF | Factor: GSX2; motif: NNYMATTANN; match class: 1 | TF:M04328_1 | 3165 | 8.80E-05 | 372 | 67 |
| GO:BP | positive regulation of leukocyte mediated immunity | GO:0002705 | 115 | 8.90E-05 | 372 | 11 |
| GO:CC | clathrin-coated endocytic vesicle | GO:0045334 | 83 | 9.10E-05 | 372 | 9 |
| TF | Factor: FOXO1A:ETV7; motif: NWMAACAGGAMNNNCTTCCNN; match class: 1 | TF:M08304_1 | 6512 | 9.20E-05 | 372 | 112 |
| GO:BP | blood vessel morphogenesis | GO:0048514 | 563 | 9.30E-05 | 372 | 23 |
| TF | Factor: ZNF462; motif: YYYYCTSCWG; match class: 1 | TF:M12707_1 | 3033 | 9.30E-05 | 372 | 65 |
| GO:CC | clathrin-coated vesicle membrane | GO:0030665 | 109 | 9.50E-05 | 372 | 10 |
| TF | Factor: TORC2; motif: TGGGCTKKD | TF:M07341 | 4235 | 9.50E-05 | 372 | 82 |
| TF | Factor: Oct-1; motif: NNNRTAATNANNN | TF:M00137 | 5582 | 9.50E-05 | 372 | 100 |
| TF | Factor: c-Jun; motif: NNNNRRTGASTCAN | TF:M07102 | 4236 | 9.60E-05 | 372 | 82 |
| GO:BP | regulation of superoxide anion generation | GO:0032928 | 20 | 9.80E-05 | 372 | 6 |
| TF | Factor: Pax-6; motif: NNNNTTCACGCWTGANTKNNN | TF:M00097 | 9552 | 9.80E-05 | 372 | 149 |
| TF | Factor: GATA-1; motif: NCWGATAACA | TF:M00346 | 3594 | 9.90E-05 | 372 | 73 |
| GO:BP | negative regulation of cell population proliferation | GO:0008285 | 660 | 1.00E-04 | 372 | 25 |
| GO:BP | angiogenesis | GO:0001525 | 477 | 1.00E-04 | 372 | 21 |
| TF | Factor: CDP; motif: ATCGATNNNNNATCRAT | TF:M03950 | 3384 | 1.00E-04 | 372 | 70 |
| TF | Factor: TBR2; motif: AGGTGTGAA | TF:M01774 | 743 | 1.00E-04 | 372 | 27 |
| TF | Factor: SATB1; motif: AKWAWTAAHGRYMNWW | TF:M01723 | 2569 | 1.00E-04 | 372 | 58 |
| TF | Factor: GATA-3; motif: AGATAAGATCT; match class: 1 | TF:M12193_1 | 1274 | 1.00E-04 | 372 | 37 |
| GO:BP | positive regulation of cell killing | GO:0031343 | 50 | 1.10E-04 | 372 | 8 |
| GO:BP | regulation of endopeptidase activity | GO:0052548 | 354 | 1.10E-04 | 372 | 18 |
| MIRNA | MIRNA root | MIRNA:000000 | 13878 | 1.10E-04 | 372 | 195 |
| TF | Factor: E2F-3; motif: NNTTTTGGCGCCAAAACT | TF:M03962 | 9734 | 1.10E-04 | 372 | 151 |
| TF | Factor: CTF/NF1; motif: TTGGCN | TF:M02050 | 9395 | 1.10E-04 | 372 | 147 |
| TF | Factor: COUP-TF1; motif: TGACCTY | TF:M03542 | 4395 | 1.10E-04 | 372 | 84 |
| TF | Factor: NR1B1; motif: RAGGTCA | TF:M08963 | 4395 | 1.10E-04 | 372 | 84 |
| TF | Factor: RXR-ALPHA; motif: RAGGTCA | TF:M08968 | 4395 | 1.10E-04 | 372 | 84 |
| TF | Factor: ESE-1; motif: SATKGCGGATGCN | TF:M11381 | 13896 | 1.10E-04 | 372 | 198 |
| TF | Factor: ZNF592; motif: NSARNATGGAGKN | TF:M09766 | 6223 | 1.10E-04 | 372 | 108 |
| TF | Factor: MAF; motif: NGCTGAGTCAN; match class: 1 | TF:M00983_1 | 952 | 1.10E-04 | 372 | 31 |
| GO:BP | regulation of intracellular signal transduction | GO:1902531 | 1594 | 1.20E-04 | 372 | 42 |
| GO:BP | detection of molecule of bacterial origin | GO:0032490 | 11 | 1.20E-04 | 372 | 5 |
| GO:BP | positive regulation of protein phosphorylation | GO:0001934 | 666 | 1.20E-04 | 372 | 25 |
| GO:BP | negative regulation of defense response | GO:0031348 | 209 | 1.20E-04 | 372 | 14 |
| GO:BP | regulation of mast cell activation involved in immune response | GO:0033006 | 34 | 1.20E-04 | 372 | 7 |
| GO:CC | plasma membrane protein complex | GO:0098797 | 534 | 1.20E-04 | 372 | 21 |
| GO:MF | lipid binding | GO:0008289 | 723 | 1.20E-04 | 372 | 25 |
| GO:MF | amide binding | GO:0033218 | 354 | 1.20E-04 | 372 | 17 |
| TF | Factor: ITF-2; motif: NGCAGSTGKS | TF:M09962 | 4254 | 1.20E-04 | 372 | 82 |
| TF | Factor: AP-4; motif: NCAGCTGYNGNCN | TF:M01860 | 5298 | 1.20E-04 | 372 | 96 |
| TF | Factor: E2F2; motif: NNTTTTGGCGCCAAAAWN | TF:M03959 | 7017 | 1.20E-04 | 372 | 118 |
| TF | Factor: STAT1; motif: NTTCCNGGA | TF:M04721 | 6860 | 1.20E-04 | 372 | 116 |
| TF | Factor: TEF5; motif: NNWGGWATKTTT | TF:M08903 | 1515 | 1.20E-04 | 372 | 41 |
| TF | Factor: HLTF; motif: AGSCARAAAGYRGSTGS; match class: 1 | TF:M04613_1 | 1635 | 1.20E-04 | 372 | 43 |
| TF | Factor: T-box; motif: NASGTGTNAN | TF:M12021 | 3685 | 1.20E-04 | 372 | 74 |
| TF | Factor: AP-1; motif: NNNTGAGTCAKCN | TF:M00517 | 4189 | 1.20E-04 | 372 | 81 |
| GO:BP | regulation of cellular component movement | GO:0051270 | 972 | 1.30E-04 | 372 | 31 |
| GO:BP | anatomical structure formation involved in morphogenesis | GO:0048646 | 1026 | 1.30E-04 | 372 | 32 |
| GO:BP | regulation of response to stress | GO:0080134 | 1192 | 1.30E-04 | 372 | 35 |
| GO:BP | regulation of microglial cell mediated cytotoxicity | GO:1904149 | 5 | 1.30E-04 | 372 | 4 |
| GO:BP | microglial cell mediated cytotoxicity | GO:0090634 | 5 | 1.30E-04 | 372 | 4 |
| GO:BP | regulation of hippocampal neuron apoptotic process | GO:0110089 | 5 | 1.30E-04 | 372 | 4 |
| GO:BP | hippocampal neuron apoptotic process | GO:0110088 | 5 | 1.30E-04 | 372 | 4 |
| GO:CC | primary lysosome | GO:0005766 | 141 | 1.30E-04 | 372 | 11 |
| GO:CC | azurophil granule | GO:0042582 | 141 | 1.30E-04 | 372 | 11 |
| TF | Factor: HOXB5; motif: NNYMATTANN | TF:M04349 | 4707 | 1.30E-04 | 372 | 88 |
| TF | Factor: Six-2; motif: NNGTAWCRNN | TF:M11032 | 9253 | 1.30E-04 | 372 | 145 |
| TF | Factor: DEC; motif: SCCCAMGTGAAGN | TF:M00997 | 7759 | 1.30E-04 | 372 | 127 |
| TF | Factor: AP-2rep; motif: CAGTGGG | TF:M00468 | 6084 | 1.30E-04 | 372 | 106 |
| TF | Factor: GSX1; motif: NNYMATTANN | TF:M04327 | 4712 | 1.30E-04 | 372 | 88 |
| TF | Factor: SUHW1; motif: TCTCTCCAGTRTGAATTCTCTGAT | TF:M10541 | 6086 | 1.30E-04 | 372 | 106 |
| GO:BP | T cell differentiation | GO:0030217 | 245 | 1.40E-04 | 372 | 15 |
| GO:BP | regulation of programmed cell death | GO:0043067 | 1308 | 1.40E-04 | 372 | 37 |
| GO:CC | endosome | GO:0005768 | 949 | 1.40E-04 | 372 | 29 |
| TF | Factor: Msx-1; motif: NNNNANTAATTANTNN | TF:M01412 | 2525 | 1.40E-04 | 372 | 57 |
| TF | Factor: AP-1; motif: NTGASTCAG | TF:M00199 | 4054 | 1.40E-04 | 372 | 79 |
| TF | Factor: Six-1; motif: NCGTATCRNN | TF:M11028 | 1949 | 1.40E-04 | 372 | 48 |
| GO:BP | regulation of pattern recognition receptor signaling pathway | GO:0062207 | 95 | 1.50E-04 | 372 | 10 |
| TF | Factor: Sox-10; motif: MAANRRNNNCWTTGTT | TF:M10065 | 5861 | 1.50E-04 | 372 | 103 |
| TF | Factor: Ngn-2; motif: RACATATGTY | TF:M11131 | 708 | 1.50E-04 | 372 | 26 |
| TF | Factor: IRF-8; motif: AGTTTCW | TF:M01665 | 3988 | 1.50E-04 | 372 | 78 |
| TF | Factor: T-bet; motif: NNGNRGGTGTGRN; match class: 1 | TF:M10089_1 | 1126 | 1.50E-04 | 372 | 34 |
| TF | Factor: TEF-3:E2A; motif: NCAGSTGNGWATGYN | TF:M08438 | 4948 | 1.50E-04 | 372 | 91 |
| TF | Factor: Sox-4; motif: NYCTTTGTYYYN | TF:M10069 | 6179 | 1.50E-04 | 372 | 107 |
| TF | Factor: EAR2; motif: YGNNCTTTGNCCTK | TF:M01728 | 5407 | 1.60E-04 | 372 | 97 |
| TF | Factor: AP-1; motif: NTGACTCAN | TF:M00925 | 2147 | 1.60E-04 | 372 | 51 |
| TF | Factor: AP-4; motif: AWCAGCTGWT; match class: 1 | TF:M11208_1 | 3285 | 1.60E-04 | 372 | 68 |
| TF | Factor: NR1B1:RXR-ALPHA; motif: RRGGTCANNNNNRGGTCA | TF:M08964 | 6106 | 1.60E-04 | 372 | 106 |
| TF | Factor: NR3C1; motif: RRGAACATWMYGTYCTRN | TF:M02219 | 5643 | 1.60E-04 | 372 | 100 |
| TF | Factor: Ngn-2; motif: RACATATGTY; match class: 1 | TF:M11132_1 | 664 | 1.70E-04 | 372 | 25 |
| TF | Factor: Smad2; motif: TGTCTGNCACCT; match class: 1 | TF:M09656_1 | 1022 | 1.70E-04 | 372 | 32 |
| TF | Factor: HOXC11; motif: NGYMATWAANN | TF:M10816 | 4292 | 1.70E-04 | 372 | 82 |
| TF | Factor: Msx-2; motif: NNNGACYAATTAGYNNT | TF:M01393 | 7307 | 1.70E-04 | 372 | 121 |
| GO:BP | granulocyte migration | GO:0097530 | 123 | 1.80E-04 | 372 | 11 |
| GO:MF | NAD+ nucleosidase activity | GO:0003953 | 28 | 1.80E-04 | 372 | 6 |
| REAC | Diseases associated with the TLR signaling cascade | REAC:R-HSA-5602358 | 28 | 1.80E-04 | 372 | 6 |
| REAC | Diseases of Immune System | REAC:R-HSA-5260271 | 28 | 1.80E-04 | 372 | 6 |
| TF | Factor: SREBP-1; motif: NNNGTGGGGTGAN | TF:M04632 | 4149 | 1.80E-04 | 372 | 80 |
| TF | Factor: C/EBP; motif: NNATTGCNNAANNN | TF:M00190 | 4665 | 1.80E-04 | 372 | 87 |
| TF | Factor: PEA3; motif: ACAGGAAGGAAGTN | TF:M11429 | 4815 | 1.80E-04 | 372 | 89 |
| TF | Factor: POU3F2; motif: NTATGCWAATKAG | TF:M11909 | 4151 | 1.80E-04 | 372 | 80 |
| TF | Factor: TFAP4; motif: AWCAGCTGWT; match class: 1 | TF:M04192_1 | 3506 | 1.80E-04 | 372 | 71 |
| TF | Factor: POU3F1; motif: RNYBCATTTGCATTWCAA | TF:M03842 | 4299 | 1.80E-04 | 372 | 82 |
| GO:BP | superoxide anion generation | GO:0042554 | 36 | 1.90E-04 | 372 | 7 |
| REAC | Signal Transduction | REAC:R-HSA-162582 | 2365 | 1.90E-04 | 372 | 52 |
| TF | Factor: LRH-1; motif: TGACCTTGRNYCAAGGTCA | TF:M11830 | 6126 | 1.90E-04 | 372 | 106 |
| TF | Factor: PU.1; motif: NNNNYYYACTTCCTCTTTY; match class: 1 | TF:M01172_1 | 484 | 1.90E-04 | 372 | 21 |
| TF | Factor: DLX6; motif: NWRNGYAAWYA | TF:M12595 | 3231 | 1.90E-04 | 372 | 67 |
| TF | Factor: RORA; motif: CCYTGNCCTN | TF:M12659 | 6920 | 1.90E-04 | 372 | 116 |
| TF | Factor: HOXC-8; motif: NNNNNGTAATTANNNT; match class: 1 | TF:M01321_1 | 3583 | 1.90E-04 | 372 | 72 |
| TF | Factor: ESRRA; motif: CAAGGTCANNYSAAGGTCA; match class: 1 | TF:M04458_1 | 3797 | 1.90E-04 | 372 | 75 |
| TF | Factor: POU3F2; motif: TAATKAGNNNNTAATKA; match class: 1 | TF:M11908_1 | 975 | 1.90E-04 | 372 | 31 |
| TF | Factor: AML1; motif: TGTGGTK | TF:M01658 | 3726 | 1.90E-04 | 372 | 74 |
| GO:BP | positive regulation of cell migration | GO:0030335 | 496 | 2.00E-04 | 372 | 21 |
| GO:BP | negative regulation of myeloid leukocyte mediated immunity | GO:0002887 | 12 | 2.00E-04 | 372 | 5 |
| GO:BP | neutrophil degranulation | GO:0043312 | 12 | 2.00E-04 | 372 | 5 |
| KEGG | Toxoplasmosis | KEGG:05145 | 108 | 2.00E-04 | 372 | 9 |
| TF | Factor: C-JUN:FRA-1; motif: NNATGACTCATNN | TF:M08938 | 3094 | 2.00E-04 | 372 | 65 |
| TF | Factor: C/EBPdelta; motif: NTTGCNCMAYN | TF:M09882 | 1725 | 2.00E-04 | 372 | 44 |
| GO:BP | regulation of phosphorylation | GO:0042325 | 1159 | 2.10E-04 | 372 | 34 |
| REAC | Signaling by Interleukins | REAC:R-HSA-449147 | 411 | 2.10E-04 | 372 | 18 |
| TF | Factor: DLX2; motif: AMSMCTGACKG | TF:M08873 | 6450 | 2.10E-04 | 372 | 110 |
| TF | Factor: NeuroD-2; motif: RMCATATGKY; match class: 1 | TF:M01287_1 | 1666 | 2.10E-04 | 372 | 43 |
| GO:BP | homeostatic process | GO:0042592 | 1631 | 2.20E-04 | 372 | 42 |
| REAC | GPCR downstream signalling | REAC:R-HSA-388396 | 549 | 2.20E-04 | 372 | 21 |
| TF | Factor: VDR; motif: RRGGTCANNGRGKTCA | TF:M09671 | 7096 | 2.20E-04 | 372 | 118 |
| TF | Factor: RARA; motif: AGGTCANNYAAAGGTCA | TF:M08019 | 4689 | 2.20E-04 | 372 | 87 |
| TF | Factor: ZNF684; motif: MAAGGGGTGGACTGT; match class: 1 | TF:M10550_1 | 8324 | 2.20E-04 | 372 | 133 |
| REAC | Phosphorylation of CD3 and TCR zeta chains | REAC:R-HSA-202427 | 16 | 2.30E-04 | 372 | 5 |
| TF | Factor: SREBP-1; motif: RTCACCCCAY | TF:M07405 | 5912 | 2.30E-04 | 372 | 103 |
| TF | Factor: E2A; motif: CACCTGNY | TF:M00973 | 4692 | 2.30E-04 | 372 | 87 |
| TF | Factor: Sohlh2; motif: NNCACGTGNN | TF:M11075 | 10531 | 2.30E-04 | 372 | 159 |
| GO:BP | regulation of myeloid cell differentiation | GO:0045637 | 187 | 2.40E-04 | 372 | 13 |
| GO:BP | negative regulation of endopeptidase activity | GO:0010951 | 187 | 2.40E-04 | 372 | 13 |
| GO:BP | antigen processing and presentation of exogenous peptide antigen | GO:0002478 | 55 | 2.40E-04 | 372 | 8 |
| GO:BP | B cell receptor signaling pathway | GO:0050853 | 55 | 2.40E-04 | 372 | 8 |
| REAC | Toll-like Receptor Cascades | REAC:R-HSA-168898 | 148 | 2.40E-04 | 372 | 11 |
| GO:BP | killing of cells of another organism | GO:0031640 | 23 | 2.50E-04 | 372 | 6 |
| TF | Factor: VDR; motif: NRRGTTCA | TF:M08979 | 3966 | 2.50E-04 | 372 | 77 |
| TF | Factor: PMX2B; motif: NNNAATTAATTAANNNG | TF:M01356 | 2639 | 2.50E-04 | 372 | 58 |
| GO:BP | positive regulation of cellular protein metabolic process | GO:0032270 | 1284 | 2.60E-04 | 372 | 36 |
| TF | Factor: NFATc1; motif: NATGGAAANWNANTTTYCMN | TF:M04051 | 4704 | 2.60E-04 | 372 | 87 |
| TF | Factor: POU2F1; motif: NWTATGCWAATN | TF:M04070 | 2180 | 2.60E-04 | 372 | 51 |
| TF | Factor: IRF-7; motif: AAGWGAA; match class: 1 | TF:M01884_1 | 3752 | 2.60E-04 | 372 | 74 |
| TF | Factor: Ets; motif: ANNCACTTCCTG | TF:M00771 | 3397 | 2.60E-04 | 372 | 69 |
| GO:BP | lysosome localization | GO:0032418 | 77 | 2.70E-04 | 372 | 9 |
| GO:BP | vacuolar localization | GO:1990849 | 77 | 2.70E-04 | 372 | 9 |
| TF | Factor: TFIIB; motif: YTNTMTGMSN | TF:M08904 | 5698 | 2.70E-04 | 372 | 100 |
| TF | Factor: GR; motif: RGWACATWATGTWCY | TF:M11847 | 4559 | 2.70E-04 | 372 | 85 |
| TF | Factor: BRN1; motif: NANNTATGCATAATNNA | TF:M01324 | 4046 | 2.70E-04 | 372 | 78 |
| TF | Factor: HoxA5; motif: RTCATTAN | TF:M10704 | 4413 | 2.70E-04 | 372 | 83 |
| TF | Factor: Sox-10; motif: AACAATRGNCYATTGTT; match class: 1 | TF:M11632_1 | 3758 | 2.70E-04 | 372 | 74 |
| GO:BP | T cell migration | GO:0072678 | 56 | 2.80E-04 | 372 | 8 |
| GO:BP | positive regulation of phosphorylation | GO:0042327 | 746 | 2.80E-04 | 372 | 26 |
| TF | Factor: MafG; motif: CMATGACTCAGCAGA; match class: 1 | TF:M07048_1 | 1269 | 2.80E-04 | 372 | 36 |
| TF | Factor: HOXB2; motif: NNTMATTANN | TF:M04347 | 2782 | 2.80E-04 | 372 | 60 |
| TF | Factor: Fra-2; motif: TGASTCANCN | TF:M03870 | 3056 | 2.80E-04 | 372 | 64 |
| TF | Factor: Sox-10; motif: AACAATGGNCCATTGTT; match class: 1 | TF:M11633_1 | 2648 | 2.80E-04 | 372 | 58 |
| TF | Factor: HoxA5; motif: RTCATTAN | TF:M10707 | 2383 | 2.90E-04 | 372 | 54 |
| TF | Factor: NR1B1; motif: NRGGTCANRRGGTCAN | TF:M11792 | 4126 | 2.90E-04 | 372 | 79 |
| TF | Factor: Ngn-2; motif: RACATATGTY | TF:M11134 | 1215 | 3.00E-04 | 372 | 35 |
| TF | Factor: c-Fos; motif: ACTCACCA | TF:M04802 | 6417 | 3.00E-04 | 372 | 109 |
| TF | Factor: LKLF; motif: GGGGTGGKSN | TF:M07261 | 8865 | 3.00E-04 | 372 | 139 |
| TF | Factor: Foxm1; motif: NTGTTTRT | TF:M07255 | 4796 | 3.00E-04 | 372 | 88 |
| TF | Factor: TBX2; motif: TCACACCWN | TF:M08831 | 1049 | 3.00E-04 | 372 | 32 |
| GO:BP | negative regulation of cell adhesion | GO:0007162 | 261 | 3.10E-04 | 372 | 15 |
| GO:BP | regulation of peptidase activity | GO:0052547 | 379 | 3.10E-04 | 372 | 18 |
| TF | Factor: Oct-4; motif: YWTTSTNATGCAAAT | TF:M01125 | 1629 | 3.10E-04 | 372 | 42 |
| GO:BP | response to interferon-gamma | GO:0034341 | 130 | 3.20E-04 | 372 | 11 |
| GO:MF | complement binding | GO:0001848 | 17 | 3.20E-04 | 372 | 5 |
| TF | Factor: Erm:E2A; motif: CASGTGNNNCGGAAGNN | TF:M08571 | 9890 | 3.20E-04 | 372 | 151 |
| TF | Factor: CART1; motif: NGNNYTAATTARTNNNN; match class: 1 | TF:M01362_1 | 2860 | 3.20E-04 | 372 | 61 |
| GO:MF | identical protein binding | GO:0042802 | 1948 | 3.30E-04 | 372 | 45 |
| TF | Factor: POU2F1:PEA3; motif: ACCGGATATGCAN | TF:M08402 | 6988 | 3.30E-04 | 372 | 116 |
| GO:BP | regulation of protein phosphorylation | GO:0001932 | 1017 | 3.40E-04 | 372 | 31 |
| GO:BP | myeloid leukocyte differentiation | GO:0002573 | 193 | 3.40E-04 | 372 | 13 |
| TF | Factor: Zic1; motif: KGGGTGGTC | TF:M00448 | 9729 | 3.40E-04 | 372 | 149 |
| GO:BP | interleukin-1 production | GO:0032612 | 104 | 3.50E-04 | 372 | 10 |
| GO:BP | regulation of interleukin-1 production | GO:0032652 | 104 | 3.50E-04 | 372 | 10 |
| KEGG | Antigen processing and presentation | KEGG:04612 | 62 | 3.50E-04 | 372 | 7 |
| TF | Factor: C/EBPdelta; motif: NRTTGCGYAAYN | TF:M11321 | 4812 | 3.50E-04 | 372 | 88 |
| TF | Factor: JUND:C-FOS; motif: NNATGAGTCATNN | TF:M08933 | 3998 | 3.50E-04 | 372 | 77 |
| TF | Factor: SMAD; motif: TNGNCAGACWN; match class: 1 | TF:M00974_1 | 594 | 3.50E-04 | 372 | 23 |
| TF | Factor: NF-E2; motif: TGCTGAGTCAY | TF:M00037 | 5577 | 3.60E-04 | 372 | 98 |
| TF | Factor: Sox-4; motif: NNNNNAATTGTTNNNNN | TF:M02909 | 3641 | 3.60E-04 | 372 | 72 |
| TF | Factor: VDR; motif: NRGGTCANNGRGKTCA | TF:M10106 | 6125 | 3.60E-04 | 372 | 105 |
| GO:BP | negative regulation of peptidase activity | GO:0010466 | 194 | 3.70E-04 | 372 | 13 |
| GO:BP | response to oxygen-containing compound | GO:1901700 | 1480 | 3.70E-04 | 372 | 39 |
| GO:MF | MHC class II receptor activity | GO:0032395 | 8 | 3.70E-04 | 372 | 4 |
| TF | Factor: Oct3; motif: NNWTATGYWAATKANN | TF:M11935 | 2402 | 3.70E-04 | 372 | 54 |
| TF | Factor: Ngn-2; motif: RMCATATGYY | TF:M11133 | 1170 | 3.70E-04 | 372 | 34 |
| GO:BP | regulation of myeloid leukocyte differentiation | GO:0002761 | 105 | 3.80E-04 | 372 | 10 |
| REAC | Peptide ligand-binding receptors | REAC:R-HSA-375276 | 155 | 3.80E-04 | 372 | 11 |
| TF | Factor: Sox-30; motif: NRACAATG | TF:M11640 | 794 | 3.80E-04 | 372 | 27 |
| GO:BP | positive regulation of cell motility | GO:2000147 | 516 | 3.90E-04 | 372 | 21 |
| TF | Factor: TBX15; motif: NAGGTGTGAN | TF:M12039 | 2145 | 3.90E-04 | 372 | 50 |
| TF | Factor: Smad4; motif: NWGTCTGNCACCT | TF:M10061 | 3223 | 3.90E-04 | 372 | 66 |
| GO:BP | regulation of gene expression | GO:0010468 | 4415 | 4.00E-04 | 372 | 82 |
| TF | Factor: Oct-1; motif: MKVATTTGCATATT | TF:M00161 | 2408 | 4.00E-04 | 372 | 54 |
| GO:BP | positive regulation of protein metabolic process | GO:0051247 | 1367 | 4.10E-04 | 372 | 37 |
| GO:BP | response to fungus | GO:0009620 | 40 | 4.10E-04 | 372 | 7 |
| TF | Factor: JUN; motif: NRTGACTCA | TF:M12589 | 3227 | 4.10E-04 | 372 | 66 |
| TF | Factor: Oct3; motif: NYWTTSWTATGCAAAT; match class: 1 | TF:M09646_1 | 1119 | 4.10E-04 | 372 | 33 |
| TF | Factor: GKLF; motif: CCTCCYN; match class: 1 | TF:M01835_1 | 9081 | 4.20E-04 | 372 | 141 |
| GO:BP | positive regulation of toll-like receptor signaling pathway | GO:0034123 | 25 | 4.30E-04 | 372 | 6 |
| GO:BP | alpha-beta T cell activation | GO:0046631 | 134 | 4.30E-04 | 372 | 11 |
| TF | Factor: POU2F1; motif: ANKNRWATGSAAWYAW | TF:M10033 | 3948 | 4.40E-04 | 372 | 76 |
| TF | Factor: NF-1C; motif: TTGGCNN | TF:M07300 | 5371 | 4.40E-04 | 372 | 95 |
| TF | Factor: MYB; motif: NNCAACTGNN | TF:M09813 | 4688 | 4.50E-04 | 372 | 86 |
| TF | Factor: TCF-3; motif: CTTTGA; match class: 1 | TF:M03858_1 | 4541 | 4.60E-04 | 372 | 84 |
| TF | Factor: TCF-7; motif: TCAAAG; match class: 1 | TF:M00805_1 | 4541 | 4.60E-04 | 372 | 84 |
| TF | Factor: Fra-1; motif: NNNTGAGTCAYN | TF:M09922 | 3807 | 4.60E-04 | 372 | 74 |
| TF | Factor: POU2F1:HOXB13; motif: NGMATATACCAATAAA | TF:M08405 | 3168 | 4.60E-04 | 372 | 65 |
| TF | Factor: Lhx8; motif: TGATTG | TF:M07476 | 7600 | 4.70E-04 | 372 | 123 |
| TF | Factor: HOXD1; motif: NATYCAKCAN | TF:M12625 | 4694 | 4.70E-04 | 372 | 86 |
| TF | Factor: MIBP1; motif: WNWCCCCAGCTR | TF:M03878 | 2488 | 4.80E-04 | 372 | 55 |
| TF | Factor: AP-4; motif: YCAGCTGNKN | TF:M10097 | 5226 | 4.80E-04 | 372 | 93 |
| GO:BP | cellular component organization | GO:0016043 | 6205 | 4.90E-04 | 372 | 105 |
| TF | Factor: SREBP-1; motif: KATCACCCCAC | TF:M00221 | 6557 | 4.90E-04 | 372 | 110 |
| TF | Factor: E2F2; motif: AAAAATGGCGCCAAAAWG | TF:M03958 | 9442 | 4.90E-04 | 372 | 145 |
| TF | Factor: ATF3; motif: GRTGACKCA | TF:M12582 | 6319 | 4.90E-04 | 372 | 107 |
| GO:BP | positive regulation of response to biotic stimulus | GO:0002833 | 167 | 5.10E-04 | 372 | 12 |
| GO:BP | regulation of chemotaxis | GO:0050920 | 200 | 5.20E-04 | 372 | 13 |
| TF | Factor: MAFA; motif: TCAGCAN | TF:M01709 | 5314 | 5.30E-04 | 372 | 94 |
| TF | Factor: HNF3alpha; motif: TRTTTGYTYWN | TF:M00724 | 2167 | 5.30E-04 | 372 | 50 |
| TF | Factor: AP4; motif: NMNCAGCTGGN | TF:M12580 | 5085 | 5.30E-04 | 372 | 91 |
| TF | Factor: Sox-10; motif: NACAATRNNNNYATTGTN | TF:M11635 | 5086 | 5.40E-04 | 372 | 91 |
| GO:BP | regulation of lymphocyte differentiation | GO:0045619 | 168 | 5.50E-04 | 372 | 12 |
| GO:BP | positive regulation of chemokine production | GO:0032722 | 61 | 5.50E-04 | 372 | 8 |
| TF | Factor: TIF2; motif: ANANAGAWAAGN | TF:M09849 | 2975 | 5.50E-04 | 372 | 62 |
| TF | Factor: STAT5A; motif: NAWTTCYNGGAANYN | TF:M00457 | 3468 | 5.70E-04 | 372 | 69 |
| TF | Factor: ER-alpha; motif: AGGTCASMNTGACCY | TF:M09909 | 5943 | 5.70E-04 | 372 | 102 |
| GO:BP | chemokine production | GO:0032602 | 84 | 5.80E-04 | 372 | 9 |
| GO:BP | regulation of chemokine production | GO:0032642 | 84 | 5.80E-04 | 372 | 9 |
| GO:BP | positive regulation of cellular component movement | GO:0051272 | 529 | 5.80E-04 | 372 | 21 |
| TF | Factor: NR1B2; motif: RAGGTCRTGACCTY | TF:M11800 | 3188 | 5.80E-04 | 372 | 65 |
| TF | Factor: Sox-17; motif: NCCATTGTNNN | TF:M10066 | 2239 | 5.80E-04 | 372 | 51 |
| GO:BP | negative regulation of T cell activation | GO:0050868 | 110 | 6.00E-04 | 372 | 10 |
| GO:BP | positive regulation of locomotion | GO:0040017 | 530 | 6.00E-04 | 372 | 21 |
| TF | Factor: Sox-8; motif: NNAACAATRN | TF:M11628 | 1082 | 6.00E-04 | 372 | 32 |
| TF | Factor: Hoxa9; motif: NTTAAWTAMA | TF:M07457 | 3906 | 6.00E-04 | 372 | 75 |
| TF | Factor: GR; motif: AGAACAN; match class: 1 | TF:M07355_1 | 813 | 6.10E-04 | 372 | 27 |
| TF | Factor: VDR:RXR-ALPHA; motif: NRGGTCANNNGGTTCNN | TF:M08980 | 9219 | 6.10E-04 | 372 | 142 |
| TF | Factor: C/EBPepsilon; motif: HAANMTTKCNWMAC | TF:M01868 | 2507 | 6.10E-04 | 372 | 55 |
| GO:BP | negative regulation of cell-cell adhesion | GO:0022408 | 170 | 6.20E-04 | 372 | 12 |
| GO:BP | antigen processing and presentation of exogenous antigen | GO:0019884 | 62 | 6.30E-04 | 372 | 8 |
| TF | Factor: MRF4; motif: CASCTGC; match class: 1 | TF:M03831_1 | 2377 | 6.40E-04 | 372 | 53 |
| TF | Factor: HTF4; motif: CASCTGB; match class: 1 | TF:M02018_1 | 2377 | 6.40E-04 | 372 | 53 |
| TF | Factor: JunB; motif: TGACTCAN | TF:M03551 | 3551 | 6.40E-04 | 372 | 70 |
| TF | Factor: AP1; motif: TGACTCA | TF:M02280 | 3553 | 6.50E-04 | 372 | 70 |
| KEGG | Fc gamma R-mediated phagocytosis | KEGG:04666 | 95 | 6.60E-04 | 372 | 8 |
| GO:BP | axon ensheathment | GO:0008366 | 140 | 6.70E-04 | 372 | 11 |
| GO:BP | ensheathment of neurons | GO:0007272 | 140 | 6.70E-04 | 372 | 11 |
| GO:CC | ficolin-1-rich granule membrane | GO:0101003 | 55 | 6.80E-04 | 372 | 7 |
| TF | Factor: Pitx3; motif: GATTANA | TF:M07057 | 1864 | 6.80E-04 | 372 | 45 |
| TF | Factor: POU3F2; motif: TAATKAGNNNNTAATKA | TF:M11908 | 2993 | 6.80E-04 | 372 | 62 |
| GO:BP | regulation of inflammatory response to antigenic stimulus | GO:0002861 | 43 | 6.90E-04 | 372 | 7 |
| TF | Factor: Dlx-5; motif: NNRGYAATTRNYKNNN; match class: 1 | TF:M01388_1 | 4735 | 6.90E-04 | 372 | 86 |
| TF | Factor: Tst-1; motif: NNKGAATTAVAVTDN | TF:M00133 | 4735 | 6.90E-04 | 372 | 86 |
| GO:BP | positive regulation of secretion | GO:0051047 | 278 | 7.00E-04 | 372 | 15 |
| TF | Factor: Erm; motif: RSAGGAAGGAAGTN | TF:M11423 | 4288 | 7.00E-04 | 372 | 80 |
| TF | Factor: AP-1; motif: NTGASTCAG; match class: 1 | TF:M00199_1 | 3560 | 7.10E-04 | 372 | 70 |
| GO:BP | NIK/NF-kappaB signaling | GO:0038061 | 141 | 7.20E-04 | 372 | 11 |
| GO:BP | regulation of response to biotic stimulus | GO:0002831 | 318 | 7.20E-04 | 372 | 16 |
| TF | Factor: MTF-1; motif: GTGTGCANMACTTTGCGCAC | TF:M01242 | 3492 | 7.30E-04 | 372 | 69 |
| TF | Factor: AP-4:Dlx-3; motif: NCAGCTGNNNNNNGTAATKR | TF:M08671 | 3638 | 7.50E-04 | 372 | 71 |
| TF | Factor: ZNFPT1; motif: YCNNCNCWGCCNY | TF:M12721 | 8164 | 7.80E-04 | 372 | 129 |
| TF | Factor: ZNF586; motif: CAGGCCYRGAGG | TF:M10491 | 12307 | 7.80E-04 | 372 | 177 |
| TF | Factor: AML3; motif: NWAACCACRAAAACCACRAN | TF:M11999 | 5905 | 7.90E-04 | 372 | 101 |
| GO:BP | positive regulation of NIK/NF-kappaB signaling | GO:1901224 | 64 | 8.10E-04 | 372 | 8 |
| TF | Factor: HOXA4; motif: RTMATTAN | TF:M10716 | 3290 | 8.30E-04 | 372 | 66 |
| TF | Factor: HDAC1; motif: KGCARGGTC | TF:M07041 | 11165 | 8.30E-04 | 372 | 164 |
| TF | Factor: Ngn-2; motif: RACATATGTY; match class: 1 | TF:M11131_1 | 624 | 8.40E-04 | 372 | 23 |
| TF | Factor: gsh-1; motif: NTMATNRN; match class: 1 | TF:M10750_1 | 3221 | 8.40E-04 | 372 | 65 |
| GO:CC | plasma membrane raft | GO:0044853 | 108 | 8.60E-04 | 372 | 9 |
| TF | Factor: Lhx2; motif: WATTAN | TF:M07047 | 9102 | 8.70E-04 | 372 | 140 |
| GO:BP | regulation of superoxide metabolic process | GO:0090322 | 28 | 8.80E-04 | 372 | 6 |
| TF | Factor: Fra-1; motif: NRTGACTCATN | TF:M09615 | 3439 | 8.90E-04 | 372 | 68 |
| TF | Factor: Tbx3; motif: AGGTGTNR | TF:M08786 | 3157 | 9.00E-04 | 372 | 64 |
| GO:BP | regulation of leukocyte mediated cytotoxicity | GO:0001910 | 65 | 9.10E-04 | 372 | 8 |
| GO:BP | second-messenger-mediated signaling | GO:0019932 | 284 | 9.10E-04 | 372 | 15 |
| TF | Factor: PEA3; motif: NTCGTAAATGCA | TF:M11431 | 9024 | 9.10E-04 | 372 | 139 |
| TF | Factor: LCOR; motif: CMAAGKNCANN | TF:M12628 | 2949 | 9.10E-04 | 372 | 61 |
| TF | Factor: FOSL1; motif: NNATGACTCATNN | TF:M12518 | 3875 | 9.20E-04 | 372 | 74 |
| TF | Factor: SP2; motif: NNRRCCAATSRGNRNSNGSN | TF:M12668 | 10569 | 9.20E-04 | 372 | 157 |
| GO:BP | blood vessel development | GO:0001568 | 641 | 9.30E-04 | 372 | 23 |
| TF | Factor: POU3F1; motif: NTAATKWATGCNN | TF:M11929 | 5613 | 9.40E-04 | 372 | 97 |
| TF | Factor: NFATc3; motif: NTTTCCRYGGAAAN | TF:M11988 | 6242 | 9.40E-04 | 372 | 105 |
| GO:BP | positive regulation of T cell proliferation | GO:0042102 | 89 | 9.50E-04 | 372 | 9 |
| GO:BP | regulation of regulated secretory pathway | GO:1903305 | 145 | 9.50E-04 | 372 | 11 |
| TF | Factor: Oct-2; motif: NTATGCWAATN; match class: 1 | TF:M11896_1 | 993 | 9.50E-04 | 372 | 30 |
| TF | Factor: Fra-2; motif: RTGACTCANNN | TF:M09616 | 3952 | 9.60E-04 | 372 | 75 |
| TF | Factor: SREBP-1; motif: NSNNTCACNCCANNN | TF:M01173 | 4099 | 9.60E-04 | 372 | 77 |
| TF | Factor: six4; motif: NCGTATCRNN | TF:M11025 | 5538 | 9.60E-04 | 372 | 96 |
| TF | Factor: Spi-B; motif: NAAWGNGGAAGTN | TF:M11377 | 399 | 9.60E-04 | 372 | 18 |
| GO:BP | antigen processing and presentation | GO:0019882 | 116 | 9.80E-04 | 372 | 10 |
| TF | Factor: Tal-1; motif: CTTCCTTMTCTSY | TF:M01591 | 1889 | 9.80E-04 | 372 | 45 |
| TF | Factor: Sox-9; motif: AANGGNNNCWTTGTTY | TF:M10070 | 2212 | 9.80E-04 | 372 | 50 |
| GO:BP | movement of cell or subcellular component | GO:0006928 | 1918 | 1.00E-03 | 372 | 45 |
| GO:MF | pattern recognition receptor activity | GO:0038187 | 21 | 1.00E-03 | 372 | 5 |
| GO:MF | amyloid-beta binding | GO:0001540 | 81 | 1.00E-03 | 372 | 8 |
| REAC | Initial triggering of complement | REAC:R-HSA-166663 | 21 | 1.00E-03 | 372 | 5 |
| TF | Factor: MTF-1; motif: NTTTTGCACACGGCNYN | TF:M12279 | 5776 | 1.00E-03 | 372 | 99 |
| TF | Factor: Sox-10; motif: CWTTGTN | TF:M01131 | 7621 | 1.00E-03 | 372 | 122 |
| TF | Factor: Smad1; motif: NGGCAGACN | TF:M03845 | 3958 | 1.00E-03 | 372 | 75 |
| TF | Factor: POU3F2; motif: ATGMATWWATTCAT | TF:M00463 | 2550 | 1.00E-03 | 372 | 55 |
| GO:BP | positive regulation of protein modification process | GO:0031401 | 906 | 1.10E-03 | 372 | 28 |
| GO:BP | anatomical structure morphogenesis | GO:0009653 | 2447 | 1.10E-03 | 372 | 53 |
| GO:BP | toll-like receptor 2 signaling pathway | GO:0034134 | 16 | 1.10E-03 | 372 | 5 |
| GO:BP | detection of other organism | GO:0098543 | 16 | 1.10E-03 | 372 | 5 |
| GO:CC | ficolin-1-rich granule | GO:0101002 | 175 | 1.10E-03 | 372 | 11 |
| GO:CC | receptor complex | GO:0043235 | 376 | 1.10E-03 | 372 | 16 |
| TF | Factor: NFE2L1; motif: NNNATGACTCAGCANW; match class: 1 | TF:M12499_1 | 2824 | 1.10E-03 | 372 | 59 |
| TF | Factor: Smad4; motif: TGTCTGN | TF:M01889 | 4482 | 1.10E-03 | 372 | 82 |
| TF | Factor: POU1F1; motif: AWTATGCWAATKAG | TF:M04069 | 2623 | 1.10E-03 | 372 | 56 |
| GO:BP | positive regulation of vasculature development | GO:1904018 | 148 | 1.20E-03 | 372 | 11 |
| GO:BP | positive regulation of angiogenesis | GO:0045766 | 148 | 1.20E-03 | 372 | 11 |
| GO:BP | positive regulation of cellular metabolic process | GO:0031325 | 2936 | 1.20E-03 | 372 | 60 |
| GO:MF | peptidase regulator activity | GO:0061134 | 174 | 1.20E-03 | 372 | 11 |
| TF | Factor: OC-2; motif: TCAATA | TF:M02112 | 6190 | 1.20E-03 | 372 | 104 |
| TF | Factor: CRX; motif: YTAATC; match class: 1 | TF:M01712_1 | 4120 | 1.20E-03 | 372 | 77 |
| TF | Factor: ER-alpha; motif: AGGTCACNGTGACCT | TF:M01801 | 2834 | 1.20E-03 | 372 | 59 |
| TF | Factor: GR; motif: RGWACATWAYGTWCY | TF:M11846 | 5101 | 1.20E-03 | 372 | 90 |
| TF | Factor: Fra-2; motif: NNRTGAGTCAYN | TF:M09923 | 3611 | 1.20E-03 | 372 | 70 |
| TF | Factor: Oct3; motif: WTATGCGCATAW | TF:M11934 | 8394 | 1.20E-03 | 372 | 131 |
| TF | Factor: TEF-3:C/EBPbeta; motif: RTTGCGYAAYNNNNNGGAATGY | TF:M08626 | 5958 | 1.20E-03 | 372 | 101 |
| TF | Factor: STAT3; motif: NTTCYKGGAAN | TF:M07229 | 3185 | 1.20E-03 | 372 | 64 |
| TF | Factor: PPARALPHA:RXR-ALPHA; motif: AWNTRGGTNAAAGGTCAN | TF:M08960 | 10094 | 1.20E-03 | 372 | 151 |
| TF | Factor: AP-4; motif: ANCATATGNT; match class: 1 | TF:M11211_1 | 1841 | 1.20E-03 | 372 | 44 |
| TF | Factor: Sox-9; motif: NAACAATRN | TF:M04004 | 897 | 1.20E-03 | 372 | 28 |
| GO:BP | defense response to Gram-negative bacterium | GO:0050829 | 47 | 1.30E-03 | 372 | 7 |
| GO:BP | negative regulation of leukocyte cell-cell adhesion | GO:1903038 | 120 | 1.30E-03 | 372 | 10 |
| TF | Factor: CEBPD; motif: NNTTGCACAAYNN | TF:M12567 | 1780 | 1.30E-03 | 372 | 43 |
| TF | Factor: BCL-6; motif: NYGCTTTCKAGGAANN | TF:M12341 | 5187 | 1.30E-03 | 372 | 91 |
| TF | Factor: CDP:HOXA13; motif: ATCGATYAYSYCRTAAA; match class: 1 | TF:M08201_1 | 2433 | 1.30E-03 | 372 | 53 |
| TF | Factor: Pax-2; motif: CAYSCCTSAGT | TF:M03839 | 4355 | 1.30E-03 | 372 | 80 |
| TF | Factor: Dlx-5; motif: NNRGYAATTRNYKNNN | TF:M01388 | 6366 | 1.30E-03 | 372 | 106 |
| GO:BP | defense response to fungus | GO:0050832 | 30 | 1.40E-03 | 372 | 6 |
| TF | Factor: TCF-3; motif: NCTTTGWTNTKYW | TF:M01594 | 3987 | 1.40E-03 | 372 | 75 |
| TF | Factor: VDR,; motif: RRTGNMCYTNNTGAMCCNYNT; match class: 1 | TF:M00966_1 | 2778 | 1.40E-03 | 372 | 58 |
| TF | Factor: TR4; motif: ACCCCGS; match class: 1 | TF:M04934_1 | 13946 | 1.40E-03 | 372 | 194 |
| TF | Factor: POU2F1:HOXB13; motif: NGMATATACCAATAAA; match class: 1 | TF:M08405_1 | 744 | 1.40E-03 | 372 | 25 |
| TF | Factor: Oct-2; motif: NNNATTATGCAN | TF:M11897 | 3920 | 1.40E-03 | 372 | 74 |
| GO:BP | positive regulation of myeloid leukocyte differentiation | GO:0002763 | 48 | 1.50E-03 | 372 | 7 |
| TF | Factor: HEY1; motif: NYYYYATCTNN | TF:M12614 | 4742 | 1.50E-03 | 372 | 85 |
| TF | Factor: NFATc1; motif: TTTTCCATGGAAAN | TF:M04053 | 7187 | 1.50E-03 | 372 | 116 |
| TF | Factor: TGIF; motif: TGACAGS | TF:M10101 | 4444 | 1.50E-03 | 372 | 81 |
| TF | Factor: LF-A1; motif: GGGSTCWR | TF:M00646 | 11261 | 1.50E-03 | 372 | 164 |
| TF | Factor: Six-2; motif: NCGTATCRNN | TF:M11031 | 7846 | 1.50E-03 | 372 | 124 |
| GO:CC | lytic vacuole membrane | GO:0098852 | 387 | 1.60E-03 | 372 | 16 |
| GO:CC | lysosomal membrane | GO:0005765 | 387 | 1.60E-03 | 372 | 16 |
| TF | Factor: MITF; motif: CATGTGM | TF:M02099 | 2582 | 1.60E-03 | 372 | 55 |
| TF | Factor: Fli-1; motif: NNRGGMAGGAAGGRRRGR; match class: 1 | TF:M09920_1 | 2997 | 1.60E-03 | 372 | 61 |
| TF | Factor: ER-alpha; motif: TGACCYN | TF:M03547 | 4003 | 1.60E-03 | 372 | 75 |
| TF | Factor: SPIC; motif: NAAAAGMGGAAGTA | TF:M03996 | 550 | 1.60E-03 | 372 | 21 |
| TF | Factor: SOX13; motif: NNACAATGGNN | TF:M09787 | 550 | 1.60E-03 | 372 | 21 |
| TF | Factor: Pax-3; motif: TCGTCACRCTTHM | TF:M00360 | 10921 | 1.60E-03 | 372 | 160 |
| GO:BP | regulation of NIK/NF-kappaB signaling | GO:1901222 | 95 | 1.70E-03 | 372 | 9 |
| GO:BP | negative regulation of inflammatory response to antigenic stimulus | GO:0002862 | 31 | 1.70E-03 | 372 | 6 |
| TF | Factor: Gscl; motif: NTAATCCN | TF:M10924 | 5762 | 1.70E-03 | 372 | 98 |
| TF | Factor: Msx-2; motif: TAATTAN | TF:M07425 | 3073 | 1.70E-03 | 372 | 62 |
| TF | Factor: AP-2gamma; motif: NNNNSCCYCAGGSCN | TF:M07137 | 1078 | 1.70E-03 | 372 | 31 |
| TF | Factor: oct-2; motif: NNWNATGCAAATNN | TF:M03836 | 3147 | 1.70E-03 | 372 | 63 |
| GO:BP | response to bacterial lipoprotein | GO:0032493 | 8 | 1.80E-03 | 372 | 4 |
| GO:BP | positive regulation of lymphocyte proliferation | GO:0050671 | 124 | 1.80E-03 | 372 | 10 |
| TF | Factor: C-JUN:FRA-2; motif: NNRTGAGTCAYN | TF:M08940 | 3007 | 1.80E-03 | 372 | 61 |
| TF | Factor: DEC1; motif: NCNCACRTGNSC | TF:M08870 | 6965 | 1.80E-03 | 372 | 113 |
| TF | Factor: ZNF394; motif: NRARWRGAANNNAMWGNAAK | TF:M10147 | 4537 | 1.80E-03 | 372 | 82 |
| TF | Factor: HSF1; motif: GAANNTTCTRGNAN; match class: 1 | TF:M02017_1 | 462 | 1.80E-03 | 372 | 19 |
| REAC | Interleukin-10 signaling | REAC:R-HSA-6783783 | 41 | 1.90E-03 | 372 | 6 |
| TF | Factor: Ngn-2; motif: RACATATGTY; match class: 1 | TF:M11134_1 | 970 | 1.90E-03 | 372 | 29 |
| TF | Factor: CP2; motif: NNNNCCAGNCNN | TF:M07602 | 9815 | 1.90E-03 | 372 | 147 |
| TF | Factor: ipf1; motif: RTCATTAN | TF:M10745 | 2599 | 1.90E-03 | 372 | 55 |
| TF | Factor: HB9; motif: NTCRTTAN | TF:M10569 | 2599 | 1.90E-03 | 372 | 55 |
| GO:BP | macrophage migration | GO:1905517 | 50 | 2.00E-03 | 372 | 7 |
| GO:BP | vasculature development | GO:0001944 | 671 | 2.00E-03 | 372 | 23 |
| GO:BP | cellular response to mechanical stimulus | GO:0071260 | 72 | 2.00E-03 | 372 | 8 |
| GO:BP | regulation of apoptotic process | GO:0042981 | 1281 | 2.00E-03 | 372 | 34 |
| GO:BP | dendritic cell migration | GO:0036336 | 32 | 2.00E-03 | 372 | 6 |
| GO:MF | lipopolysaccharide binding | GO:0001530 | 24 | 2.00E-03 | 372 | 5 |
| TF | Factor: HNF-4alpha; motif: AGTCCAAR | TF:M04903 | 5939 | 2.00E-03 | 372 | 100 |
| TF | Factor: LBX2; motif: CTNRANSTAATTA | TF:M04383 | 6738 | 2.00E-03 | 372 | 110 |
| TF | Factor: ELF3; motif: NNCCACTTCCTGNN; match class: 1 | TF:M12544_1 | 334 | 2.00E-03 | 372 | 16 |
| TF | Factor: GR; motif: RGNACANMNTGTNCY | TF:M09941 | 4931 | 2.00E-03 | 372 | 87 |
| TF | Factor: TWIST; motif: CACCTGG | TF:M03582 | 7309 | 2.00E-03 | 372 | 117 |
| TF | Factor: HOXB4; motif: NTMATTAN | TF:M10720 | 1500 | 2.00E-03 | 372 | 38 |
| TF | Factor: c-Ets-2; motif: NNCTTCCTNNN; match class: 1 | TF:M07379_1 | 294 | 2.00E-03 | 372 | 15 |
| GO:BP | positive regulation of mononuclear cell proliferation | GO:0032946 | 126 | 2.10E-03 | 372 | 10 |
| GO:BP | negative regulation of cell death | GO:0060548 | 884 | 2.10E-03 | 372 | 27 |
| GO:CC | lumenal side of endoplasmic reticulum membrane | GO:0098553 | 25 | 2.10E-03 | 372 | 5 |
| GO:CC | integral component of lumenal side of endoplasmic reticulum membrane | GO:0071556 | 25 | 2.10E-03 | 372 | 5 |
| TF | Factor: nerf; motif: YRNCAGGAAGYRGSTBDS; match class: 1 | TF:M00531_1 | 1624 | 2.10E-03 | 372 | 40 |
| TF | Factor: JUNB:C-FOS; motif: NATGAGTCATNN | TF:M08925 | 2882 | 2.10E-03 | 372 | 59 |
| TF | Factor: c-Jun; motif: NRTGAGTCAYN | TF:M09965 | 3738 | 2.10E-03 | 372 | 71 |
| TF | Factor: c-Jun; motif: NATGASTCATN | TF:M11264 | 3593 | 2.10E-03 | 372 | 69 |
| TF | Factor: ipf1; motif: RNWCATTAANWN | TF:M01255 | 3449 | 2.10E-03 | 372 | 67 |
| TF | Factor: SRY; motif: AACAATNNNCATTGTT; match class: 1 | TF:M04556_1 | 5013 | 2.10E-03 | 372 | 88 |
| TF | Factor: ERF:pitx1; motif: NNCGGAWGNNNRGNTTA | TF:M08692 | 4709 | 2.10E-03 | 372 | 84 |
| TF | Factor: HOXA7; motif: GYMATTAN | TF:M10690 | 8983 | 2.10E-03 | 372 | 137 |
| GO:BP | cellular component organization or biogenesis | GO:0071840 | 6398 | 2.20E-03 | 372 | 105 |
| TF | Factor: CDP:T-bet; motif: NTCACACNYYRATCRATM | TF:M08203 | 5093 | 2.20E-03 | 372 | 89 |
| TF | Factor: JunB; motif: NRRTGASTCAK | TF:M07103 | 2010 | 2.20E-03 | 372 | 46 |
| TF | Factor: RelB:p50; motif: RGAAANTCCCYNNHGC | TF:M03882 | 3891 | 2.20E-03 | 372 | 73 |
| TF | Factor: NFATc3; motif: NTTTCCRYGGAAAN; match class: 1 | TF:M11988_1 | 5797 | 2.20E-03 | 372 | 98 |
| TF | Factor: HOXC-8; motif: NNNNNGTAATTANNNT | TF:M01321 | 4639 | 2.20E-03 | 372 | 83 |
| GO:BP | apoptotic cell clearance | GO:0043277 | 51 | 2.30E-03 | 372 | 7 |
| GO:BP | positive regulation of MAPK cascade | GO:0043410 | 435 | 2.30E-03 | 372 | 18 |
| GO:BP | neutrophil migration | GO:1990266 | 99 | 2.40E-03 | 372 | 9 |
| TF | Factor: NR1H4; motif: NNAATGACCNN | TF:M09777 | 3246 | 2.40E-03 | 372 | 64 |
| TF | Factor: Pax-5; motif: BCNNNRNGCANBGNTGNRTAGCSGCHNB | TF:M00143 | 10199 | 2.40E-03 | 372 | 151 |
| TF | Factor: C/EBPbeta; motif: ATTGCGYAAT | TF:M07413 | 2826 | 2.40E-03 | 372 | 58 |
| TF | Factor: NFATc2; motif: TTTTCCATGGAAAA | TF:M11985 | 4876 | 2.40E-03 | 372 | 86 |
| TF | Factor: ZXDL; motif: NGGGGWS | TF:M05775 | 6205 | 2.40E-03 | 372 | 103 |
| GO:BP | negative regulation of B cell activation | GO:0050869 | 33 | 2.50E-03 | 372 | 6 |
| GO:BP | vertebrate eye-specific patterning | GO:0150064 | 3 | 2.50E-03 | 372 | 3 |
| GO:BP | response to triacyl bacterial lipopeptide | GO:0071725 | 3 | 2.50E-03 | 372 | 3 |
| GO:BP | cellular response to triacyl bacterial lipopeptide | GO:0071727 | 3 | 2.50E-03 | 372 | 3 |
| GO:BP | regulation of peptidyl-tyrosine phosphorylation | GO:0050730 | 230 | 2.50E-03 | 372 | 13 |
| TF | Factor: Msx-1; motif: CNGTAWNTG | TF:M00394 | 7584 | 2.50E-03 | 372 | 120 |
| TF | Factor: MATH1; motif: NCASCTGGYN | TF:M01716 | 665 | 2.50E-03 | 372 | 23 |
| TF | Factor: ER71:C/EBPdelta; motif: NNCGGAWRTTRCGYAAN | TF:M08246 | 7178 | 2.50E-03 | 372 | 115 |
| GO:BP | granulocyte chemotaxis | GO:0071621 | 100 | 2.60E-03 | 372 | 9 |
| GO:BP | negative regulation of immune effector process | GO:0002698 | 100 | 2.60E-03 | 372 | 9 |
| TF | Factor: NKX2B; motif: CACTTNA | TF:M02109 | 8588 | 2.60E-03 | 372 | 132 |
| TF | Factor: BRCA1:USF2; motif: KTNNGTTG | TF:M01082 | 6054 | 2.60E-03 | 372 | 101 |
| TF | Factor: YY1; motif: NGCCATYTTKGRCNWWNNGTGCK; match class: 1 | TF:M12276_1 | 2903 | 2.60E-03 | 372 | 59 |
| TF | Factor: JunD; motif: NATGASTCATS; match class: 1 | TF:M11268_1 | 1455 | 2.60E-03 | 372 | 37 |
| TF | Factor: Oct-4; motif: ATTGWSWTGCWAAWN; match class: 1 | TF:M01124_1 | 1335 | 2.70E-03 | 372 | 35 |
| TF | Factor: C/EBPalpha; motif: NNNTTNNGCAANN; match class: 1 | TF:M01866_1 | 771 | 2.70E-03 | 372 | 25 |
| TF | Factor: ipf1; motif: NNRNTAATTAGYNCAN | TF:M01438 | 2697 | 2.70E-03 | 372 | 56 |
| TF | Factor: c-Fos; motif: RTGAGTCAYNN | TF:M09617 | 2224 | 2.70E-03 | 372 | 49 |
| GO:BP | ion transport | GO:0006811 | 1420 | 2.80E-03 | 372 | 36 |
| TF | Factor: HOXB5; motif: NNYMATTANN; match class: 1 | TF:M04349_1 | 3333 | 2.80E-03 | 372 | 65 |
| TF | Factor: RARA; motif: GAGGTCAAAAGGTCAAKK | TF:M08018 | 3915 | 2.80E-03 | 372 | 73 |
| TF | Factor: AR; motif: GNNCNNNNTGTTCTN | TF:M01996 | 2495 | 2.80E-03 | 372 | 53 |
| TF | Factor: HOXB5; motif: GTCATTAN | TF:M10701 | 4817 | 2.80E-03 | 372 | 85 |
| TF | Factor: znf136; motif: CAAGAATWCTATAYCCAG; match class: 1 | TF:M10535_1 | 2029 | 2.80E-03 | 372 | 46 |
| KEGG | Epstein-Barr virus infection | KEGG:05169 | 188 | 2.90E-03 | 372 | 10 |
| GO:BP | B cell differentiation | GO:0030183 | 131 | 3.00E-03 | 372 | 10 |
| GO:BP | positive regulation of lymphocyte differentiation | GO:0045621 | 102 | 3.00E-03 | 372 | 9 |
| TF | Factor: CLOCK; motif: CNGNCACGTGNNNM | TF:M09884 | 7117 | 3.00E-03 | 372 | 114 |
| TF | Factor: HFH2; motif: NNWAYRTAAACW | TF:M11555 | 3923 | 3.00E-03 | 372 | 73 |
| TF | Factor: ZNF436; motif: TCCTCCAGGAAGCCY; match class: 1 | TF:M10477_1 | 7782 | 3.10E-03 | 372 | 122 |
| GO:BP | cellular chemical homeostasis | GO:0055082 | 741 | 3.20E-03 | 372 | 24 |
| TF | Factor: HOXD12:Elk-1; motif: NRNCGGAAGTCGTAAAN | TF:M08359 | 5293 | 3.20E-03 | 372 | 91 |
| TF | Factor: GABP-alpha; motif: AACCGGAAR | TF:M04748 | 14181 | 3.20E-03 | 372 | 195 |
| TF | Factor: RAR-gamma; motif: RGGTCANNNTGNCCNNNN | TF:M10045 | 6400 | 3.20E-03 | 372 | 105 |
| TF | Factor: LRH-1; motif: TGACCTTGRNYCAAGGTCA; match class: 1 | TF:M11830_1 | 5296 | 3.20E-03 | 372 | 91 |
| TF | Factor: Blimp-1; motif: RRGNGAAAGNRANNN | TF:M10526 | 1974 | 3.20E-03 | 372 | 45 |
| KEGG | Influenza A | KEGG:05164 | 153 | 3.30E-03 | 372 | 9 |
| TF | Factor: HSF1; motif: TCYAGAANNTTC | TF:M07259 | 4006 | 3.30E-03 | 372 | 74 |
| TF | Factor: HOXC4; motif: RTCRTTAN | TF:M10718 | 2239 | 3.30E-03 | 372 | 49 |
| TF | Factor: AR; motif: AGWACATNWTGTTCT | TF:M00447 | 3496 | 3.40E-03 | 372 | 67 |
| TF | Factor: RORbeta; motif: TGACCYA; match class: 1 | TF:M01722_1 | 944 | 3.40E-03 | 372 | 28 |
| TF | Factor: VSX1; motif: NGCYAATTRNN | TF:M04450 | 5852 | 3.40E-03 | 372 | 98 |
| TF | Factor: HOXA10; motif: TTATNNYWN | TF:M07257 | 2998 | 3.50E-03 | 372 | 60 |
| TF | Factor: POU3F1; motif: NTAATKWATGCNN; match class: 1 | TF:M11929_1 | 1472 | 3.50E-03 | 372 | 37 |
| TF | Factor: NFATc4; motif: NRYGGAAANW | TF:M11995 | 578 | 3.50E-03 | 372 | 21 |
| TF | Factor: p73; motif: NNRCAWGYCCARRCWTGYC | TF:M10025 | 5463 | 3.50E-03 | 372 | 93 |
| TF | Factor: Pbx; motif: GATTGATKGNNS | TF:M00998 | 4313 | 3.50E-03 | 372 | 78 |
| GO:MF | opsonin binding | GO:0001846 | 13 | 3.60E-03 | 372 | 4 |
| REAC | Translocation of ZAP-70 to Immunological synapse | REAC:R-HSA-202430 | 13 | 3.60E-03 | 372 | 4 |
| TF | Factor: SREBP; motif: NNNNYCACNCCANNN | TF:M01168 | 5699 | 3.60E-03 | 372 | 96 |
| TF | Factor: C-JUN:FOSB; motif: NATGACTCAY | TF:M08936 | 2723 | 3.60E-03 | 372 | 56 |
| TF | Factor: AP-4; motif: NNCAGCTGNN | TF:M11212 | 2517 | 3.60E-03 | 372 | 53 |
| TF | Factor: Isl2; motif: YTAAGTG | TF:M02082 | 6418 | 3.70E-03 | 372 | 105 |
| TF | Factor: Sox-9; motif: MACARWGNNNYCNTTNW | TF:M08838 | 3505 | 3.70E-03 | 372 | 67 |
| TF | Factor: Pax-2; motif: NNNNGTCANGNRTKANNNN; match class: 1 | TF:M00098_1 | 1663 | 3.70E-03 | 372 | 40 |
| TF | Factor: Freac-3; motif: NNNNNGTAAATAAACA | TF:M00291 | 4394 | 3.70E-03 | 372 | 79 |
| TF | Factor: GATA-3; motif: AGATAACGATCW | TF:M12192 | 3947 | 3.80E-03 | 372 | 73 |
| TF | Factor: Fli-1; motif: MGGAAGT; match class: 1 | TF:M07382_1 | 1237 | 3.80E-03 | 372 | 33 |
| TF | Factor: C-ets-1; motif: AGGAAGN; match class: 1 | TF:M01870_1 | 1237 | 3.80E-03 | 372 | 33 |
| TF | Factor: SPI1; motif: AGGAAGT; match class: 1 | TF:M02278_1 | 1237 | 3.80E-03 | 372 | 33 |
| TF | Factor: PEA3; motif: AGGAAGT; match class: 1 | TF:M03579_1 | 1237 | 3.80E-03 | 372 | 33 |
| GO:MF | endopeptidase inhibitor activity | GO:0004866 | 127 | 3.90E-03 | 372 | 9 |
| TF | Factor: Nkx2-3; motif: NNCGTTRWS | TF:M10638 | 9672 | 3.90E-03 | 372 | 144 |
| TF | Factor: COUP-TF1; motif: GRGGKSARAGGTCAGNG | TF:M09886 | 4855 | 3.90E-03 | 372 | 85 |
| TF | Factor: HMGIY; motif: NNKKNAWTTTNYTNN; match class: 1 | TF:M01010_1 | 2523 | 3.90E-03 | 372 | 53 |
| TF | Factor: c-Myb; motif: NNNNCMGTTNNN | TF:M07299 | 3223 | 3.90E-03 | 372 | 63 |
| TF | Factor: HOXA13; motif: ATAAMA; match class: 1 | TF:M01292_1 | 6832 | 3.90E-03 | 372 | 110 |
| GO:BP | positive regulation of leukocyte proliferation | GO:0070665 | 135 | 4.00E-03 | 372 | 10 |
| GO:BP | regulation of reactive oxygen species metabolic process | GO:2000377 | 135 | 4.00E-03 | 372 | 10 |
| GO:BP | viral entry into host cell | GO:0046718 | 135 | 4.00E-03 | 372 | 10 |
| TF | Factor: HSF1; motif: GAANNTTCTRGNAN | TF:M02017 | 3440 | 4.00E-03 | 372 | 66 |
| TF | Factor: Sox-2; motif: NNCCTTTGTNYYN; match class: 1 | TF:M10067_1 | 1065 | 4.00E-03 | 372 | 30 |
| TF | Factor: GATA-1; motif: MNAGATAANR | TF:M00347 | 3733 | 4.00E-03 | 372 | 70 |
| TF | Factor: DMRT5; motif: NNNNGWTACANT | TF:M11513 | 1733 | 4.10E-03 | 372 | 41 |
| TF | Factor: Sox-9; motif: NNNACAATRG; match class: 1 | TF:M11626_1 | 1241 | 4.10E-03 | 372 | 33 |
| TF | Factor: SPIB; motif: NAAAAGMGGAAGTN | TF:M03995 | 634 | 4.10E-03 | 372 | 22 |
| GO:BP | neutrophil chemotaxis | GO:0030593 | 79 | 4.20E-03 | 372 | 8 |
| GO:BP | negative regulation of proteolysis | GO:0045861 | 280 | 4.20E-03 | 372 | 14 |
| GO:BP | positive regulation of interleukin-12 production | GO:0032735 | 36 | 4.20E-03 | 372 | 6 |
| TF | Factor: ERF:FOXO1A; motif: RTMAACAGGAARNS; match class: 1 | TF:M08241_1 | 1671 | 4.20E-03 | 372 | 40 |
| TF | Factor: RXRA; motif: RRGGTCATGACCYY; match class: 1 | TF:M04490_1 | 3016 | 4.20E-03 | 372 | 60 |
| GO:BP | cation transport | GO:0006812 | 1088 | 4.30E-03 | 372 | 30 |
| TF | Factor: elf5; motif: AANRAGGAAGTR | TF:M11388 | 537 | 4.30E-03 | 372 | 20 |
| TF | Factor: Oct-2; motif: NNNATTTGCATRT | TF:M07120 | 3962 | 4.30E-03 | 372 | 73 |
| TF | Factor: Sox-10; motif: NACAATRNNNNYATTGTN; match class: 1 | TF:M11635_1 | 4716 | 4.40E-03 | 372 | 83 |
| TF | Factor: MZF-1; motif: KNGNKAGGGGNAA | TF:M00084 | 5964 | 4.40E-03 | 372 | 99 |
| TF | Factor: Brn-3c; motif: NNTTATTAATKANKNC | TF:M01408 | 3964 | 4.40E-03 | 372 | 73 |
| GO:MF | glycosaminoglycan binding | GO:0005539 | 200 | 4.50E-03 | 372 | 11 |
| REAC | Disease | REAC:R-HSA-1643685 | 1493 | 4.50E-03 | 372 | 35 |
| TF | Factor: Hoxa9; motif: NTTAAWTAMA; match class: 1 | TF:M07457_1 | 1366 | 4.50E-03 | 372 | 35 |
| TF | Factor: ipf1; motif: CATTAR; match class: 1 | TF:M01275_1 | 2742 | 4.50E-03 | 372 | 56 |
| TF | Factor: DPF2; motif: NYCACYTCCYCNYYCY | TF:M09760 | 7424 | 4.50E-03 | 372 | 117 |
| REAC | Generation of second messenger molecules | REAC:R-HSA-202433 | 28 | 4.60E-03 | 372 | 5 |
| TF | Factor: RARA; motif: AGGTCAANNARAGGTCA | TF:M04483 | 3600 | 4.60E-03 | 372 | 68 |
| TF | Factor: LRH-1; motif: TCAAGGTCRYGACCTTGR | TF:M11828 | 9272 | 4.60E-03 | 372 | 139 |
| TF | Factor: AP-4:Max; motif: NCAGCTGNNNNNNNCACGTGN; match class: 1 | TF:M08674_1 | 4802 | 4.70E-03 | 372 | 84 |
| GO:BP | myelination | GO:0042552 | 138 | 4.80E-03 | 372 | 10 |
| KEGG | Toll-like receptor signaling pathway | KEGG:04620 | 92 | 4.80E-03 | 372 | 7 |
| KEGG | Th17 cell differentiation | KEGG:04659 | 92 | 4.80E-03 | 372 | 7 |
| TF | Factor: ALX3; motif: NNYAATTANN | TF:M04279 | 3243 | 4.80E-03 | 372 | 63 |
| GO:BP | negative regulation of metabolic process | GO:0009892 | 2853 | 4.90E-03 | 372 | 57 |
| TF | Factor: E2F2; motif: AAAATGGCGCCATTTT | TF:M04517 | 8014 | 4.90E-03 | 372 | 124 |
| TF | Factor: FOX; motif: KWTTGTTTRTTTW | TF:M00809 | 3102 | 4.90E-03 | 372 | 61 |
| TF | Factor: YB-1; motif: CCAMTCNGMR | TF:M03805 | 8857 | 4.90E-03 | 372 | 134 |
| GO:BP | gliogenesis | GO:0042063 | 284 | 5.00E-03 | 372 | 14 |
| GO:BP | amyloid-beta clearance | GO:0097242 | 37 | 5.00E-03 | 372 | 6 |
| TF | Factor: FOXM1; motif: NAGASTGATTA; match class: 1 | TF:M04611_1 | 2751 | 5.00E-03 | 372 | 56 |
| GO:BP | protein phosphorylation | GO:0006468 | 1459 | 5.10E-03 | 372 | 36 |
| GO:MF | lipopeptide binding | GO:0071723 | 5 | 5.10E-03 | 372 | 3 |
| GO:MF | complement component C3b binding | GO:0001851 | 5 | 5.10E-03 | 372 | 3 |
| KEGG | Acute myeloid leukemia | KEGG:05221 | 64 | 5.10E-03 | 372 | 6 |
| KEGG | Rap1 signaling pathway | KEGG:04015 | 201 | 5.10E-03 | 372 | 10 |
| TF | Factor: CDP; motif: TRATCRATAN | TF:M03952 | 3537 | 5.10E-03 | 372 | 67 |
| TF | Factor: islet1; motif: SCAMTTAN | TF:M11012 | 5352 | 5.10E-03 | 372 | 91 |
| TF | Factor: GATA-3; motif: MGATAASATCT | TF:M12195 | 3177 | 5.10E-03 | 372 | 62 |
| TF | Factor: ER-beta; motif: GTCANASTGRCCYNR; match class: 1 | TF:M01875_1 | 1196 | 5.20E-03 | 372 | 32 |
| GO:CC | secretory granule lumen | GO:0034774 | 289 | 5.30E-03 | 372 | 13 |
| GO:MF | peptidase inhibitor activity | GO:0030414 | 132 | 5.30E-03 | 372 | 9 |
| TF | Factor: HOXA4; motif: RYCATTRN | TF:M08771 | 2345 | 5.30E-03 | 372 | 50 |
| REAC | Antigen processing-Cross presentation | REAC:R-HSA-1236975 | 101 | 5.50E-03 | 372 | 8 |
| TF | Factor: Fra-2; motif: NRRTGASTCAB | TF:M07092 | 2621 | 5.50E-03 | 372 | 54 |
| TF | Factor: CEBPD; motif: NRTTGCNCAAYN | TF:M12587 | 2484 | 5.50E-03 | 372 | 52 |
| GO:BP | positive regulation of mononuclear cell migration | GO:0071677 | 58 | 5.60E-03 | 372 | 7 |
| GO:MF | antigen binding | GO:0003823 | 49 | 5.60E-03 | 372 | 6 |
| TF | Factor: Sox-3; motif: SNNACAATRK | TF:M11617 | 3258 | 5.60E-03 | 372 | 63 |
| TF | Factor: OSR2; motif: NGCTACTGNN | TF:M12051 | 2148 | 5.60E-03 | 372 | 47 |
| GO:BP | regulation of protein modification process | GO:0031399 | 1404 | 5.70E-03 | 372 | 35 |
| GO:CC | cytoplasmic vesicle lumen | GO:0060205 | 291 | 5.70E-03 | 372 | 13 |
| TF | Factor: SUHW1; motif: TCTCTCCAGTRTGAATTCTCTGAT; match class: 1 | TF:M10541_1 | 1442 | 5.70E-03 | 372 | 36 |
| GO:BP | positive regulation of ERK1 and ERK2 cascade | GO:0070374 | 174 | 5.80E-03 | 372 | 11 |
| GO:BP | regulation of proteolysis | GO:0030162 | 662 | 5.80E-03 | 372 | 22 |
| KEGG | Alcoholic liver disease | KEGG:04936 | 128 | 5.80E-03 | 372 | 8 |
| TF | Factor: HOXA7; motif: GYMATTAN; match class: 1 | TF:M10690_1 | 4980 | 5.80E-03 | 372 | 86 |
| GO:BP | entry into host | GO:0044409 | 141 | 5.90E-03 | 372 | 10 |
| GO:BP | regulation of macrophage migration | GO:1905521 | 38 | 5.90E-03 | 372 | 6 |
| TF | Factor: ESE-1; motif: NNANVAGGAAGTNN | TF:M09902 | 2697 | 5.90E-03 | 372 | 55 |
| GO:CC | coated vesicle membrane | GO:0030662 | 172 | 6.00E-03 | 372 | 10 |
| GO:CC | phagocytic vesicle membrane | GO:0030670 | 76 | 6.00E-03 | 372 | 7 |
| TF | Factor: T3R-beta; motif: NTGACCTNRNYNAGGTCAN | TF:M11820 | 4448 | 6.00E-03 | 372 | 79 |
| TF | Factor: TCF-1; motif: ASATCAAAG | TF:M11602 | 5688 | 6.00E-03 | 372 | 95 |
| GO:CC | vesicle lumen | GO:0031983 | 293 | 6.10E-03 | 372 | 13 |
| TF | Factor: p300; motif: ACNTCCG | TF:M04826 | 15885 | 6.10E-03 | 372 | 212 |
| TF | Factor: SPI1; motif: NGRGGAAGTN; match class: 1 | TF:M02078_1 | 321 | 6.10E-03 | 372 | 15 |
| TF | Factor: MOX1; motif: NTCRTTAN | TF:M10761 | 2561 | 6.10E-03 | 372 | 53 |
| GO:BP | cellular response to interferon-gamma | GO:0071346 | 111 | 6.20E-03 | 372 | 9 |
| GO:BP | MyD88-dependent toll-like receptor signaling pathway | GO:0002755 | 22 | 6.20E-03 | 372 | 5 |
| TF | Factor: GABPA; motif: NNCACTTCCTGTNN | TF:M12494 | 1762 | 6.20E-03 | 372 | 41 |
| TF | Factor: GATA-3; motif: ANAGATMWWA | TF:M00351 | 2155 | 6.20E-03 | 372 | 47 |
| TF | Factor: POU5F1; motif: NNATGCAAANN | TF:M09782 | 2982 | 6.20E-03 | 372 | 59 |
| TF | Factor: p53; motif: NGRCWTGYCY; match class: 1 | TF:M00272_1 | 974 | 6.20E-03 | 372 | 28 |
| TF | Factor: CTF/NF1; motif: TTGGCN; match class: 1 | TF:M02050_1 | 3413 | 6.20E-03 | 372 | 65 |
| GO:BP | negative regulation of leukocyte mediated immunity | GO:0002704 | 59 | 6.30E-03 | 372 | 7 |
| GO:BP | regulation of interleukin-2 production | GO:0032663 | 59 | 6.30E-03 | 372 | 7 |
| GO:BP | interleukin-2 production | GO:0032623 | 59 | 6.30E-03 | 372 | 7 |
| GO:MF | hydrolase activity, hydrolyzing N-glycosyl compounds | GO:0016799 | 50 | 6.30E-03 | 372 | 6 |
| TF | Factor: YB-1; motif: NNNNCCAATNN | TF:M03862 | 7885 | 6.30E-03 | 372 | 122 |
| TF | Factor: RARA; motif: AGGTCAANNARAGGTCA; match class: 1 | TF:M04483_1 | 502 | 6.30E-03 | 372 | 19 |
| TF | Factor: Pit-1; motif: NTAATGAKATGCRN | TF:M11950 | 4913 | 6.30E-03 | 372 | 85 |
| TF | Factor: Sox-4; motif: NYCTTTGTYYYN; match class: 1 | TF:M10069_1 | 1449 | 6.30E-03 | 372 | 36 |
| TF | Factor: CDP:SRF; motif: NCCWTAYAAGGTMNKRATCRATN; match class: 1 | TF:M08520_1 | 3199 | 6.40E-03 | 372 | 62 |
| GO:CC | lumenal side of membrane | GO:0098576 | 31 | 6.50E-03 | 372 | 5 |
| GO:BP | cellular homeostasis | GO:0019725 | 828 | 6.60E-03 | 372 | 25 |
| GO:BP | positive regulation of leukocyte chemotaxis | GO:0002690 | 84 | 6.60E-03 | 372 | 8 |
| TF | Factor: ESE-1; motif: NTGTGCGGATGCN | TF:M11385 | 10801 | 6.60E-03 | 372 | 156 |
| TF | Factor: STAT1; motif: NNTTTCYNGGAARNNNNNNNNN | TF:M01260 | 1832 | 6.70E-03 | 372 | 42 |
| TF | Factor: THAP1; motif: YTGCCCNNA; match class: 1 | TF:M07407_1 | 3639 | 6.70E-03 | 372 | 68 |
| TF | Factor: RAR-gamma; motif: NRGGTCAAAAGGTCAN | TF:M11804 | 2639 | 6.70E-03 | 372 | 54 |
| TF | Factor: CSX; motif: NCCACTTRAN | TF:M10640 | 5466 | 6.70E-03 | 372 | 92 |
| GO:BP | response to abiotic stimulus | GO:0009628 | 1055 | 6.80E-03 | 372 | 29 |
| TF | Factor: Nkx2-3; motif: NSCACTTNNC | TF:M10634 | 1641 | 6.80E-03 | 372 | 39 |
| GO:BP | viral life cycle | GO:0019058 | 292 | 6.90E-03 | 372 | 14 |
| TF | Factor: AP-2alpha; motif: NTNSCCTGRGGSNAN | TF:M09590 | 4694 | 6.90E-03 | 372 | 82 |
| TF | Factor: T3R-beta; motif: NRRGGTCRTGACCYYN | TF:M11817 | 4694 | 6.90E-03 | 372 | 82 |
| TF | Factor: RUSH-1alpha; motif: NNMCWTNKNN | TF:M01107 | 2573 | 7.00E-03 | 372 | 53 |
| GO:BP | superoxide metabolic process | GO:0006801 | 60 | 7.10E-03 | 372 | 7 |
| GO:BP | inflammatory response to antigenic stimulus | GO:0002437 | 60 | 7.10E-03 | 372 | 7 |
| GO:CC | vacuolar membrane | GO:0005774 | 436 | 7.10E-03 | 372 | 16 |
| TF | Factor: PBX2; motif: TGANTGACAGN | TF:M09736 | 4240 | 7.10E-03 | 372 | 76 |
| TF | Factor: MZF-1; motif: TGGGGAR; match class: 1 | TF:M01733_1 | 4394 | 7.20E-03 | 372 | 78 |
| TF | Factor: SOX2; motif: NNACAATGGNN | TF:M12505 | 1396 | 7.20E-03 | 372 | 35 |
| TF | Factor: Dlx-5; motif: AATTAN; match class: 1 | TF:M03546_1 | 4779 | 7.40E-03 | 372 | 83 |
| TF | Factor: DRI1; motif: AATTAA; match class: 1 | TF:M01654_1 | 4779 | 7.40E-03 | 372 | 83 |
| TF | Factor: hoxa9; motif: NCGGYCATWAAAWTANW | TF:M01351 | 1712 | 7.50E-03 | 372 | 40 |
| TF | Factor: E2A; motif: NRMCASCTGCNNN | TF:M02088 | 2511 | 7.60E-03 | 372 | 52 |
| TF | Factor: POU6F1; motif: NTAATGAKATGNNN | TF:M11958 | 2239 | 7.60E-03 | 372 | 48 |
| GO:BP | regulation of cellular localization | GO:0060341 | 781 | 7.80E-03 | 372 | 24 |
| TF | Factor: GATA-4; motif: NNGATAASNN | TF:M12207 | 2377 | 7.80E-03 | 372 | 50 |
| GO:BP | membrane raft organization | GO:0031579 | 23 | 7.90E-03 | 372 | 5 |
| TF | Factor: Pax-4; motif: WNNNYTAATTARYNSNN | TF:M01385 | 2724 | 8.00E-03 | 372 | 55 |
| TF | Factor: ESE-1; motif: ACCAGGAAGTANNNNNNNAAWAA | TF:M11383 | 3150 | 8.10E-03 | 372 | 61 |
| TF | Factor: GATA-6; motif: NGATAACGATCN | TF:M12209 | 1717 | 8.10E-03 | 372 | 40 |
| TF | Factor: POU2F2; motif: NTATGCWAATN | TF:M04072 | 1912 | 8.10E-03 | 372 | 43 |
| TF | Factor: IRF-1; motif: TTCACTT | TF:M00747 | 4332 | 8.10E-03 | 372 | 77 |
| GO:BP | phosphorylation | GO:0016310 | 1744 | 8.20E-03 | 372 | 40 |
| TF | Factor: AP-4; motif: NNCAGCTGNN; match class: 1 | TF:M11212_1 | 2449 | 8.20E-03 | 372 | 51 |
| TF | Factor: JUND; motif: NNATGACTCATNN | TF:M12524 | 3514 | 8.20E-03 | 372 | 66 |
| TF | Factor: CDX-2; motif: TTTATN | TF:M02087 | 8261 | 8.20E-03 | 372 | 126 |
| GO:MF | cell adhesion molecule binding | GO:0050839 | 535 | 8.30E-03 | 372 | 18 |
| TF | Factor: Ngn-2; motif: RACATATGTC | TF:M04183 | 822 | 8.40E-03 | 372 | 25 |
| TF | Factor: Prx2; motif: TYAWAKTAA | TF:M02115 | 2870 | 8.50E-03 | 372 | 57 |
| TF | Factor: POU3F3; motif: WTNAATAWKNAW | TF:M04082 | 2941 | 8.50E-03 | 372 | 58 |
| TF | Factor: GR; motif: NNNNNNCNNTNTGTNCTNN; match class: 1 | TF:M00192_1 | 1531 | 8.60E-03 | 372 | 37 |
| GO:BP | MAPK cascade | GO:0000165 | 732 | 8.70E-03 | 372 | 23 |
| GO:BP | positive regulation of alpha-beta T cell activation | GO:0046635 | 62 | 8.80E-03 | 372 | 7 |
| GO:BP | regulation of ERK1 and ERK2 cascade | GO:0070372 | 258 | 9.00E-03 | 372 | 13 |
| GO:BP | tube morphogenesis | GO:0035239 | 788 | 9.00E-03 | 372 | 24 |
| GO:CC | plasma membrane region | GO:0098590 | 1176 | 9.00E-03 | 372 | 29 |
| GO:MF | endopeptidase regulator activity | GO:0061135 | 141 | 9.00E-03 | 372 | 9 |
| TF | Factor: Dlx-7; motif: NTCRTTAN | TF:M10600 | 6220 | 9.00E-03 | 372 | 101 |
| REAC | DAP12 interactions | REAC:R-HSA-2172127 | 32 | 9.10E-03 | 372 | 5 |
| TF | Factor: FRA-2; motif: RTGANTCA | TF:M08914 | 3234 | 9.10E-03 | 372 | 62 |
| TF | Factor: Pit-1; motif: NTAATTTATKCGY | TF:M11949 | 4653 | 9.30E-03 | 372 | 81 |
| GO:BP | positive regulation of pattern recognition receptor signaling pathway | GO:0062208 | 41 | 9.40E-03 | 372 | 6 |
| GO:BP | positive regulation of myeloid cell differentiation | GO:0045639 | 88 | 9.40E-03 | 372 | 8 |
| GO:BP | positive regulation of T cell differentiation | GO:0045582 | 88 | 9.40E-03 | 372 | 8 |
| TF | Factor: Oct-2; motif: ATTTGCA | TF:M04715 | 5273 | 9.40E-03 | 372 | 89 |
| TF | Factor: NFATc1; motif: TTTTCCATGGAAAN; match class: 1 | TF:M04053_1 | 4962 | 9.40E-03 | 372 | 85 |
| TF | Factor: TEF-3:C/EBPdelta; motif: RGWATGYNRTTRCGYAAY | TF:M08413 | 5118 | 9.40E-03 | 372 | 87 |
| GO:CC | extracellular matrix | GO:0031012 | 497 | 9.60E-03 | 372 | 17 |
| TF | Factor: GATA-3; motif: MGATAACGATCT | TF:M12194 | 2811 | 9.60E-03 | 372 | 56 |
| TF | Factor: BCL-6; motif: WRCTTTCKAGGRAT | TF:M01185 | 5120 | 9.60E-03 | 372 | 87 |
| TF | Factor: c-Fos; motif: NNTGASTCATN | TF:M07090 | 3604 | 9.60E-03 | 372 | 67 |
| TF | Factor: HOXD12:Elk-1; motif: NRNCGGAAGTCGTAAAN; match class: 1 | TF:M08359_1 | 997 | 9.70E-03 | 372 | 28 |
| TF | Factor: MyoD; motif: YGNCAGSTGTYN | TF:M11185 | 997 | 9.70E-03 | 372 | 28 |
| TF | Factor: GATA-1; motif: NTGNNNNNNNSAGATAAGR | TF:M09621 | 4581 | 9.70E-03 | 372 | 80 |
| GO:BP | positive regulation of interferon-gamma production | GO:0032729 | 63 | 9.80E-03 | 372 | 7 |
| TF | Factor: GATA-4; motif: AGATAAN | TF:M03549 | 3977 | 9.80E-03 | 372 | 72 |
| TF | Factor: SOX; motif: CTCTTTGTTANGA | TF:M01014 | 4278 | 9.80E-03 | 372 | 76 |
| TF | Factor: c-Myb; motif: NNNAACKGNNN | TF:M01821 | 3242 | 9.80E-03 | 372 | 62 |
| GO:BP | regulation of monocyte chemotaxis | GO:0090025 | 24 | 9.90E-03 | 372 | 5 |
| GO:CC | external encapsulating structure | GO:0030312 | 498 | 9.90E-03 | 372 | 17 |
| GO:BP | positive regulation of microglial cell mediated cytotoxicity | GO:1904151 | 4 | 1.00E-02 | 372 | 3 |
| GO:BP | positive regulation of neutrophil degranulation | GO:0043315 | 4 | 1.00E-02 | 372 | 3 |
| GO:BP | interleukin-3-mediated signaling pathway | GO:0038156 | 4 | 1.00E-02 | 372 | 3 |
| GO:BP | chemical homeostasis | GO:0048878 | 1078 | 1.00E-02 | 372 | 29 |
| GO:BP | regulation of alpha-beta T cell activation | GO:0046634 | 89 | 1.00E-02 | 372 | 8 |
| TF | Factor: RARA; motif: ARGGTCAAAAGGTCA | TF:M04481 | 2675 | 1.00E-02 | 372 | 54 |
| TF | Factor: FOXJ2; motif: NNTGTTGTAAAYAN; match class: 1 | TF:M11585_1 | 1057 | 1.00E-02 | 372 | 29 |
| TF | Factor: ipf1; motif: NVSTAATTAC; match class: 1 | TF:M01235_1 | 1295 | 1.00E-02 | 372 | 33 |
| TF | Factor: NKX3A; motif: NTNAAGTGNTTN | TF:M10009 | 3102 | 1.00E-02 | 372 | 60 |
| TF | Factor: FAC1; motif: NNNCAMAACACRNA; match class: 1 | TF:M00456_1 | 2264 | 1.00E-02 | 372 | 48 |
| GO:BP | negative regulation of programmed cell death | GO:0043069 | 799 | 1.10E-02 | 372 | 24 |
| GO:BP | regulation of cellular metabolic process | GO:0031323 | 5347 | 1.10E-02 | 372 | 89 |
| GO:MF | molecular function regulator | GO:0098772 | 1692 | 1.10E-02 | 372 | 37 |
| KEGG | Natural killer cell mediated cytotoxicity | KEGG:04650 | 105 | 1.10E-02 | 372 | 7 |
| TF | Factor: AR; motif: GGTACANNRTGTTCT | TF:M00481 | 4286 | 1.10E-02 | 372 | 76 |
| TF | Factor: HNRPUL1; motif: NCNCAGN; match class: 1 | TF:M09746_1 | 6727 | 1.10E-02 | 372 | 107 |
| TF | Factor: HOXC4; motif: NTMATTAN | TF:M10717 | 2962 | 1.10E-02 | 372 | 58 |
| TF | Factor: Oct3; motif: NATGCAANNN | TF:M01307 | 3322 | 1.10E-02 | 372 | 63 |
| TF | Factor: ZNF775; motif: RGRGAGAAGN | TF:M12720 | 3038 | 1.10E-02 | 372 | 59 |
| TF | Factor: Oct3; motif: WTATGCGCATAW; match class: 1 | TF:M11934_1 | 7810 | 1.10E-02 | 372 | 120 |
| GO:BP | movement in host environment | GO:0052126 | 152 | 1.20E-02 | 372 | 10 |
| GO:BP | lymphocyte migration | GO:0072676 | 91 | 1.20E-02 | 372 | 8 |
| GO:BP | dendritic cell antigen processing and presentation | GO:0002468 | 12 | 1.20E-02 | 372 | 4 |
| GO:BP | negative regulation of leukocyte migration | GO:0002686 | 43 | 1.20E-02 | 372 | 6 |
| GO:CC | endosome membrane | GO:0010008 | 507 | 1.20E-02 | 372 | 17 |
| REAC | Interferon Signaling | REAC:R-HSA-913531 | 182 | 1.20E-02 | 372 | 10 |
| REAC | PD-1 signaling | REAC:R-HSA-389948 | 17 | 1.20E-02 | 372 | 4 |
| REAC | Leishmania infection | REAC:R-HSA-9658195 | 223 | 1.20E-02 | 372 | 11 |
| TF | Factor: ZABC1; motif: ATTCCNAC; match class: 1 | TF:M01306_1 | 2342 | 1.20E-02 | 372 | 49 |
| TF | Factor: NF-1B; motif: KCCAGANWN | TF:M08825 | 4990 | 1.20E-02 | 372 | 85 |
| TF | Factor: Msx-1; motif: WNGNAATTANV | TF:M08822 | 5618 | 1.20E-02 | 372 | 93 |
| TF | Factor: FOXO1A; motif: NNNYTGTTTNCN | TF:M09931 | 5699 | 1.20E-02 | 372 | 94 |
| TF | Factor: STAT6; motif: NNYTTCCY | TF:M00500 | 4076 | 1.20E-02 | 372 | 73 |
| TF | Factor: RAR-gamma; motif: RAGGTCATGACCTY | TF:M11807 | 1747 | 1.20E-02 | 372 | 40 |
| TF | Factor: ZNF586; motif: CAGGCCYRGAGG; match class: 1 | TF:M10491_1 | 6912 | 1.20E-02 | 372 | 109 |
| GO:BP | central nervous system development | GO:0007417 | 974 | 1.30E-02 | 372 | 27 |
| GO:BP | positive regulation of reactive oxygen species metabolic process | GO:2000379 | 66 | 1.30E-02 | 372 | 7 |
| GO:CC | clathrin-coated vesicle | GO:0030136 | 188 | 1.30E-02 | 372 | 10 |
| GO:MF | carboxylic acid binding | GO:0031406 | 147 | 1.30E-02 | 372 | 9 |
| KEGG | Malaria | KEGG:05144 | 48 | 1.30E-02 | 372 | 5 |
| KEGG | Leukocyte transendothelial migration | KEGG:04670 | 108 | 1.30E-02 | 372 | 7 |
| TF | Factor: DMBX1; motif: NNGGATTANN | TF:M04305 | 4383 | 1.30E-02 | 372 | 77 |
| TF | Factor: Cdx-2; motif: BNNATAAANRN | TF:M01659 | 1188 | 1.30E-02 | 372 | 31 |
| TF | Factor: MafB; motif: TGCTGASTNNN | TF:M09978 | 2768 | 1.30E-02 | 372 | 55 |
| TF | Factor: Oct-2; motif: NNNTATGCAAATNNNN | TF:M01368 | 1947 | 1.30E-02 | 372 | 43 |
| TF | Factor: Spi-B; motif: NGNGGAAGYN | TF:M02076 | 258 | 1.30E-02 | 372 | 13 |
| TF | Factor: Smad2:Smad3:Smad4; motif: NTGTCTGNCACCT | TF:M07126 | 3639 | 1.30E-02 | 372 | 67 |
| TF | Factor: UNCX; motif: NYAATTAN | TF:M04443 | 2702 | 1.30E-02 | 372 | 54 |
| GO:BP | calcium-mediated signaling | GO:0019722 | 191 | 1.40E-02 | 372 | 11 |
| GO:BP | positive regulation of leukocyte mediated cytotoxicity | GO:0001912 | 44 | 1.40E-02 | 372 | 6 |
| GO:BP | monocyte chemotaxis | GO:0002548 | 44 | 1.40E-02 | 372 | 6 |
| TF | Factor: ESR1; motif: NNNNMAGGTCACCCTGACCY | TF:M02261 | 4090 | 1.40E-02 | 372 | 73 |
| TF | Factor: STAT5B; motif: NAWTTCYNGGAAWTN | TF:M00459 | 2634 | 1.40E-02 | 372 | 53 |
| TF | Factor: Oct-1; motif: NNNNWTATGCAAATNTNNN | TF:M00135 | 1692 | 1.40E-02 | 372 | 39 |
| TF | Factor: MLR1; motif: NTGNMCYYTGNNCYN | TF:M12637 | 3795 | 1.40E-02 | 372 | 69 |
| TF | Factor: VDR:RXR; motif: GGGTCAWNGRGTTCA | TF:M01202 | 3426 | 1.40E-02 | 372 | 64 |
| TF | Factor: HNF-3alpha; motif: CNYTAAGTAAACAAAN | TF:M11570 | 3064 | 1.40E-02 | 372 | 59 |
| TF | Factor: CP2; motif: GCHCDAMCCAG | TF:M00072 | 7846 | 1.40E-02 | 372 | 120 |
| TF | Factor: T-bet; motif: NAGGTGTGAA | TF:M12019 | 483 | 1.40E-02 | 372 | 18 |
| GO:BP | positive regulation of leukocyte degranulation | GO:0043302 | 26 | 1.50E-02 | 372 | 5 |
| GO:BP | metal ion transport | GO:0030001 | 813 | 1.50E-02 | 372 | 24 |
| GO:MF | lipoprotein particle receptor activity | GO:0030228 | 18 | 1.50E-02 | 372 | 4 |
| GO:MF | cytokine receptor activity | GO:0004896 | 85 | 1.50E-02 | 372 | 7 |
| TF | Factor: POU3F1; motif: WTGMATAAWTNA | TF:M04077 | 2294 | 1.50E-02 | 372 | 48 |
| TF | Factor: Fli-1:C/EBPbeta; motif: RNCGGAWRTTGCGCAAY | TF:M08281 | 6613 | 1.50E-02 | 372 | 105 |
| TF | Factor: FOXL2; motif: TWAKACWAMTTT | TF:M03548 | 3875 | 1.50E-02 | 372 | 70 |
| TF | Factor: C-FOS:C-JUN; motif: ATGAGTCAYN | TF:M08942 | 2715 | 1.50E-02 | 372 | 54 |
| GO:BP | regulation of tissue remodeling | GO:0034103 | 68 | 1.60E-02 | 372 | 7 |
| TF | Factor: FXR:RXR-ALPHA; motif: NAGKTCATTGACCYN | TF:M08954 | 5497 | 1.60E-02 | 372 | 91 |
| TF | Factor: POU3F2; motif: TTATGYTAAT | TF:M00464 | 2716 | 1.60E-02 | 372 | 54 |
| TF | Factor: C/EBPalpha; motif: NRTTGTGCAAYNN; match class: 1 | TF:M09596_1 | 852 | 1.60E-02 | 372 | 25 |
| TF | Factor: MEIS1A:HOXA9; motif: TGACAGKTTTAYGA | TF:M00420 | 4639 | 1.60E-02 | 372 | 80 |
| TF | Factor: nanog; motif: NYYWTTGWNATGCAAAT | TF:M09996 | 1141 | 1.60E-02 | 372 | 30 |
| TF | Factor: Ncx; motif: NAATNAATTAATAANWW; match class: 1 | TF:M01420_1 | 1701 | 1.60E-02 | 372 | 39 |
| TF | Factor: MAFB; motif: NTCAGCN; match class: 1 | TF:M08888_1 | 2789 | 1.60E-02 | 372 | 55 |
| TF | Factor: c-Ets-2; motif: NNNRGGAARNRRR | TF:M09912 | 4412 | 1.60E-02 | 372 | 77 |
| TF | Factor: JunD; motif: NRTGACGTCATS | TF:M11270 | 7948 | 1.60E-02 | 372 | 121 |
| TF | Factor: Sox-3; motif: NNAACAATRN | TF:M11616 | 393 | 1.60E-02 | 372 | 16 |
| TF | Factor: RARA; motif: ARRGGTCANSNGAGGTCA | TF:M04482 | 3221 | 1.60E-02 | 372 | 61 |
| TF | Factor: HPX42B; motif: NNYNATTANN | TF:M10681 | 2033 | 1.60E-02 | 372 | 44 |
| TF | Factor: AP-4; motif: WGARYCAGCTGYGGNCNK | TF:M00005 | 4645 | 1.60E-02 | 372 | 80 |
| GO:BP | regulation of DNA-binding transcription factor activity | GO:0051090 | 406 | 1.70E-02 | 372 | 16 |
| GO:BP | negative regulation of mast cell activation | GO:0033004 | 13 | 1.70E-02 | 372 | 4 |
| REAC | Costimulation by the CD28 family | REAC:R-HSA-388841 | 59 | 1.70E-02 | 372 | 6 |
| TF | Factor: POU3F2; motif: WTATGCWAATKAG | TF:M11904 | 2582 | 1.70E-02 | 372 | 52 |
| TF | Factor: ZNF644; motif: TCCWGCCTCTSN; match class: 1 | TF:M09737_1 | 1577 | 1.70E-02 | 372 | 37 |
| TF | Factor: Tal-1; motif: TCCAKCTGNY | TF:M00993 | 1642 | 1.70E-02 | 372 | 38 |
| TF | Factor: OC-2; motif: TCAATA; match class: 1 | TF:M02112_1 | 1837 | 1.70E-02 | 372 | 41 |
| TF | Factor: NFATc1; motif: TTTCCAYWRTGGAAA; match class: 1 | TF:M04052_1 | 3299 | 1.70E-02 | 372 | 62 |
| TF | Factor: PRDM16; motif: KGGTCATRACCM; match class: 1 | TF:M05946_1 | 1838 | 1.70E-02 | 372 | 41 |
| TF | Factor: COUP-TF,; motif: TGAMCTTTGMMCYT | TF:M00158 | 1904 | 1.70E-02 | 372 | 42 |
| TF | Factor: Oct-1; motif: TATGCAAATN | TF:M00342 | 2446 | 1.70E-02 | 372 | 50 |
| TF | Factor: NR1B2; motif: NTGACCY; match class: 1 | TF:M02111_1 | 2939 | 1.70E-02 | 372 | 57 |
| GO:BP | cellular extravasation | GO:0045123 | 69 | 1.80E-02 | 372 | 7 |
| KEGG | Th1 and Th2 cell differentiation | KEGG:04658 | 80 | 1.80E-02 | 372 | 6 |
| REAC | Activation of C3 and C5 | REAC:R-HSA-174577 | 7 | 1.80E-02 | 372 | 3 |
| TF | Factor: GEMIN3; motif: NCWGGRARRGRGNGNG | TF:M09727 | 7710 | 1.80E-02 | 372 | 118 |
| TF | Factor: Fra-1; motif: RRTGASTCAKN | TF:M07091 | 1454 | 1.80E-02 | 372 | 35 |
| TF | Factor: TTF-1; motif: CTTGASN | TF:M03891 | 3013 | 1.80E-02 | 372 | 58 |
| TF | Factor: PPARgamma; motif: NWSTRGGKSARAGGKCA; match class: 1 | TF:M10038_1 | 1269 | 1.80E-02 | 372 | 32 |
| TF | Factor: CP2/LBP-1c/LSF; motif: GCTGGNTNGNNCYNG | TF:M00947 | 7463 | 1.80E-02 | 372 | 115 |
| TF | Factor: CIZ; motif: TTTTNNNNNNNNNNNNNAAAAA; match class: 1 | TF:M12070_1 | 2108 | 1.80E-02 | 372 | 45 |
| TF | Factor: HOXA2; motif: RTCRTTAR | TF:M10736 | 1583 | 1.80E-02 | 372 | 37 |
| TF | Factor: Oct3; motif: CYWTTSWNATGCAAAT; match class: 1 | TF:M10036_1 | 696 | 1.80E-02 | 372 | 22 |
| GO:BP | positive regulation of chemotaxis | GO:0050921 | 127 | 1.90E-02 | 372 | 9 |
| GO:BP | response to mechanical stimulus | GO:0009612 | 197 | 1.90E-02 | 372 | 11 |
| GO:BP | ERK1 and ERK2 cascade | GO:0070371 | 277 | 1.90E-02 | 372 | 13 |
| TF | Factor: HNF-3beta; motif: NTRTTTRYT | TF:M02014 | 2179 | 1.90E-02 | 372 | 46 |
| TF | Factor: HOXA6; motif: NGYMATTANN | TF:M10696 | 4053 | 1.90E-02 | 372 | 72 |
| GO:BP | activation of innate immune response | GO:0002218 | 70 | 2.00E-02 | 372 | 7 |
| GO:CC | apical plasma membrane | GO:0016324 | 328 | 2.00E-02 | 372 | 13 |
| TF | Factor: STAT3; motif: NTTCCYGGAAN | TF:M09664 | 4899 | 2.00E-02 | 372 | 83 |
| TF | Factor: NURR1:RXR-ALPHA; motif: NRGGTCRTTGACCYN | TF:M08957 | 9681 | 2.00E-02 | 372 | 141 |
| TF | Factor: CDP; motif: NNNNWGWYMAATR | TF:M04610 | 3683 | 2.00E-02 | 372 | 67 |
| TF | Factor: Pit-1; motif: NNWWATTCAT | TF:M03559 | 4362 | 2.00E-02 | 372 | 76 |
| TF | Factor: PPARGAMMA; motif: NWNTRGGTYANN | TF:M08961 | 4518 | 2.00E-02 | 372 | 78 |
| REAC | FCGR3A-mediated IL10 synthesis | REAC:R-HSA-9664323 | 38 | 2.10E-02 | 372 | 5 |
| TF | Factor: SPIB; motif: NNTCACTTCCTCTTTN; match class: 1 | TF:M12498_1 | 123 | 2.10E-02 | 372 | 9 |
| TF | Factor: PMX2B; motif: NNNAATTAATTAANNNG; match class: 1 | TF:M01356_1 | 1722 | 2.10E-02 | 372 | 39 |
| TF | Factor: Erm; motif: NRRSAGGAARNGRN; match class: 1 | TF:M09916_1 | 1466 | 2.10E-02 | 372 | 35 |
| TF | Factor: Pbx; motif: NKTGATTGACRKSN | TF:M01967 | 4369 | 2.10E-02 | 372 | 76 |
| TF | Factor: Prop-1; motif: TAATTNNRTTA | TF:M10928 | 1404 | 2.10E-02 | 372 | 34 |
| GO:BP | regulation of MAPK cascade | GO:0043408 | 613 | 2.20E-02 | 372 | 20 |
| GO:BP | Fc receptor signaling pathway | GO:0038093 | 71 | 2.20E-02 | 372 | 7 |
| GO:BP | regulation of B cell differentiation | GO:0045577 | 28 | 2.20E-02 | 372 | 5 |
| TF | Factor: HNF-3alpha; motif: YTRWGTMAATATTTRCWYWN | TF:M11571 | 3470 | 2.20E-02 | 372 | 64 |
| TF | Factor: c-MYB; motif: NNNGNCAGTTN | TF:M00773 | 2962 | 2.20E-02 | 372 | 57 |
| TF | Factor: COUPTF; motif: NNNNNTGACCYTTGNMCNYNGMN | TF:M01036 | 3252 | 2.20E-02 | 372 | 61 |
| TF | Factor: JunD; motif: RTGACGTCA | TF:M04681 | 4994 | 2.20E-02 | 372 | 84 |
| TF | Factor: HOXB2:Sox-20; motif: ACAATRSNNNNNNNATYA | TF:M08719 | 1470 | 2.20E-02 | 372 | 35 |
| TF | Factor: FOXP3; motif: NNNVAAACANWD | TF:M07419 | 5230 | 2.20E-02 | 372 | 87 |
| TF | Factor: Sox-14; motif: NNNACAATGN | TF:M11618 | 927 | 2.20E-02 | 372 | 26 |
| GO:BP | regulation of adaptive immune response | GO:0002819 | 164 | 2.30E-02 | 372 | 10 |
| GO:BP | leukocyte mediated cytotoxicity | GO:0001909 | 99 | 2.30E-02 | 372 | 8 |
| TF | Factor: JUNB:FOSB; motif: NRTGASTCAT | TF:M08920 | 2610 | 2.30E-02 | 372 | 52 |
| TF | Factor: LUMAN; motif: CYCAGCYYCY | TF:M09729 | 9533 | 2.30E-02 | 372 | 139 |
| TF | Factor: PLZF; motif: ACTKTANNTN | TF:M04625 | 5075 | 2.30E-02 | 372 | 85 |
| TF | Factor: TBR2; motif: NAGGTGTGAN | TF:M12017 | 1044 | 2.30E-02 | 372 | 28 |
| TF | Factor: AP-2rep; motif: CAGTGGG; match class: 1 | TF:M00468_1 | 1409 | 2.30E-02 | 372 | 34 |
| TF | Factor: TEF-3; motif: GNTATTTTT; match class: 1 | TF:M07270_1 | 3552 | 2.30E-02 | 372 | 65 |
| TF | Factor: LHX2; motif: NNYTAATTRNNN | TF:M09976 | 1928 | 2.30E-02 | 372 | 42 |
| TF | Factor: HTF4; motif: NCACCTGN | TF:M11157 | 1601 | 2.30E-02 | 372 | 37 |
| GO:BP | production of molecular mediator involved in inflammatory response | GO:0002532 | 72 | 2.40E-02 | 372 | 7 |
| GO:BP | detection of bacterium | GO:0016045 | 14 | 2.40E-02 | 372 | 4 |
| GO:BP | negative regulation of leukocyte degranulation | GO:0043301 | 14 | 2.40E-02 | 372 | 4 |
| TF | Factor: Tal-1; motif: CAGATGG | TF:M03804 | 4459 | 2.40E-02 | 372 | 77 |
| TF | Factor: C/EBP; motif: NNATTGCNNAANNN; match class: 1 | TF:M00190_1 | 1046 | 2.40E-02 | 372 | 28 |
| TF | Factor: BCL-6; motif: NNCTTTCYAGGAA | TF:M09874 | 2405 | 2.40E-02 | 372 | 49 |
| TF | Factor: Fra-1; motif: NATGASTCAYM | TF:M11284 | 2615 | 2.40E-02 | 372 | 52 |
| TF | Factor: TTF-1; motif: NNNNCAAGNRNN | TF:M00794 | 4005 | 2.40E-02 | 372 | 71 |
| TF | Factor: C/EBPgamma:Elf-1; motif: TKRCGHAATWSCGGAAGT; match class: 1 | TF:M08676_1 | 2065 | 2.40E-02 | 372 | 44 |
| TF | Factor: CP2; motif: NTGNCTGGNN | TF:M03868 | 4006 | 2.40E-02 | 372 | 71 |
| GO:BP | positive regulation of complement activation | GO:0045917 | 5 | 2.50E-02 | 372 | 3 |
| GO:BP | detection of bacterial lipoprotein | GO:0042494 | 5 | 2.50E-02 | 372 | 3 |
| GO:BP | microglial cell activation involved in immune response | GO:0002282 | 5 | 2.50E-02 | 372 | 3 |
| GO:BP | positive regulation of neutrophil activation | GO:1902565 | 5 | 2.50E-02 | 372 | 3 |
| GO:BP | positive regulation of peptidyl-tyrosine phosphorylation | GO:0050731 | 166 | 2.50E-02 | 372 | 10 |
| GO:CC | actin cytoskeleton | GO:0015629 | 484 | 2.50E-02 | 372 | 16 |
| TF | Factor: HSF1; motif: GAANNTTCTNGN | TF:M07459 | 2902 | 2.50E-02 | 372 | 56 |
| TF | Factor: T3R-beta; motif: NTGACCTNACGTGACCTYA | TF:M11821 | 2134 | 2.50E-02 | 372 | 45 |
| TF | Factor: NR1B2; motif: NAAAGGTCAN | TF:M11798 | 2760 | 2.50E-02 | 372 | 54 |
| TF | Factor: JUND:FRA-1; motif: NRTGACTCAN | TF:M08929 | 1290 | 2.50E-02 | 372 | 32 |
| TF | Factor: c-Ets-2; motif: NACCGGAAGYRCTTCCGGTN; match class: 1 | TF:M11417_1 | 12734 | 2.50E-02 | 372 | 175 |
| TF | Factor: LHX3; motif: ATTAAW; match class: 1 | TF:M02097_1 | 5323 | 2.50E-02 | 372 | 88 |
| TF | Factor: IRF-4; motif: RGGAASWGR; match class: 1 | TF:M04818_1 | 2691 | 2.50E-02 | 372 | 53 |
| TF | Factor: MyoD; motif: CGNCAGSTGTTN | TF:M11183 | 3049 | 2.50E-02 | 372 | 58 |
| GO:BP | negative regulation of apoptotic process | GO:0043066 | 783 | 2.60E-02 | 372 | 23 |
| GO:BP | membrane organization | GO:0061024 | 728 | 2.60E-02 | 372 | 22 |
| GO:BP | regulation of exocytosis | GO:0017157 | 204 | 2.60E-02 | 372 | 11 |
| GO:BP | regulation of glial cell differentiation | GO:0045685 | 73 | 2.60E-02 | 372 | 7 |
| GO:CC | intracellular anatomical structure | GO:0005622 | 14269 | 2.60E-02 | 372 | 187 |
| TF | Factor: Pbx-1; motif: ATCAATCAW | TF:M00096 | 2137 | 2.60E-02 | 372 | 45 |
| TF | Factor: Oct-1; motif: NNGAATATKCANNNN | TF:M00136 | 3417 | 2.60E-02 | 372 | 63 |
| TF | Factor: AREB6; motif: NNYNYACCTGWVT | TF:M00412 | 3939 | 2.60E-02 | 372 | 70 |
| GO:BP | biological process involved in symbiotic interaction | GO:0044403 | 244 | 2.70E-02 | 372 | 12 |
| GO:BP | negative regulation of macromolecule metabolic process | GO:0010605 | 2662 | 2.70E-02 | 372 | 52 |
| TF | Factor: TEF-1; motif: ACATTCCWSNN | TF:M07340 | 3273 | 2.70E-02 | 372 | 61 |
| TF | Factor: GATA-3; motif: WGATAASN | TF:M08918 | 3568 | 2.70E-02 | 372 | 65 |
| TF | Factor: GATA3; motif: NGATAANN | TF:M03997 | 3568 | 2.70E-02 | 372 | 65 |
| TF | Factor: HNF4alpha; motif: VTGAACTTTGMMB | TF:M00638 | 2348 | 2.70E-02 | 372 | 48 |
| TF | Factor: ipf1; motif: TSNGYCATTANNNNC | TF:M01013 | 2348 | 2.70E-02 | 372 | 48 |
| TF | Factor: NRL; motif: NNNNTGCTGAC | TF:M04224 | 3869 | 2.70E-02 | 372 | 69 |
| TF | Factor: SREBP-2; motif: ATCACMCCAY | TF:M11084 | 2770 | 2.70E-02 | 372 | 54 |
| GO:BP | biological process involved in interaction with host | GO:0051701 | 168 | 2.80E-02 | 372 | 10 |
| GO:MF | CD4 receptor binding | GO:0042609 | 8 | 2.80E-02 | 372 | 3 |
| GO:MF | inhibitory MHC class I receptor activity | GO:0032396 | 8 | 2.80E-02 | 372 | 3 |
| TF | Factor: NR3C1; motif: NRGWACAYNRTGTWCYN; match class: 1 | TF:M04476_1 | 4480 | 2.80E-02 | 372 | 77 |
| TF | Factor: AR; motif: NGNACANNNTGTTCYNN | TF:M09589 | 4327 | 2.80E-02 | 372 | 75 |
| TF | Factor: HSF1; motif: NTTCTRGAANNTTCY | TF:M07100 | 2844 | 2.80E-02 | 372 | 55 |
| TF | Factor: CIZ; motif: TTTTNNNNNNNNNNNNNAAAAA | TF:M12070 | 2146 | 2.80E-02 | 372 | 45 |
| GO:BP | actin cytoskeleton organization | GO:0030036 | 680 | 2.90E-02 | 372 | 21 |
| REAC | Toll Like Receptor 4 (TLR4) Cascade | REAC:R-HSA-166016 | 127 | 2.90E-02 | 372 | 8 |
| TF | Factor: POU2F1:Elk-1; motif: ACCGGAWATGCAW | TF:M08398 | 7041 | 2.90E-02 | 372 | 109 |
| TF | Factor: TAL1::GATA1; motif: NTTATCWNNNNNNNNCAG | TF:M07230 | 2991 | 2.90E-02 | 372 | 57 |
| TF | Factor: ETF; motif: CCCCGCCCCYN | TF:M07039 | 14877 | 2.90E-02 | 372 | 198 |
| GO:BP | reactive oxygen species metabolic process | GO:0072593 | 207 | 3.00E-02 | 372 | 11 |
| TF | Factor: NFATc2; motif: TTTTCCATGGAAAA; match class: 1 | TF:M11985_1 | 3803 | 3.00E-02 | 372 | 68 |
| TF | Factor: c-Ets; motif: KRCAGGAARTRNKT; match class: 1 | TF:M00340_1 | 718 | 3.00E-02 | 372 | 22 |
| TF | Factor: CP2; motif: NNNNCCAGNCNN; match class: 1 | TF:M07602_1 | 4106 | 3.00E-02 | 372 | 72 |
| TF | Factor: BCL-11A; motif: NAAAGAGGAAGTGARAN; match class: 1 | TF:M09595_1 | 413 | 3.00E-02 | 372 | 16 |
| TF | Factor: CDP; motif: NNATYGATYN | TF:M11508 | 3068 | 3.00E-02 | 372 | 58 |
| GO:BP | positive regulation of nitrogen compound metabolic process | GO:0051173 | 2747 | 3.10E-02 | 372 | 53 |
| GO:BP | mature B cell differentiation | GO:0002335 | 30 | 3.10E-02 | 372 | 5 |
| GO:CC | apical part of cell | GO:0045177 | 391 | 3.10E-02 | 372 | 14 |
| TF | Factor: FXR:RXR-alpha; motif: CAAGGTSAWTAACC | TF:M00631 | 2924 | 3.10E-02 | 372 | 56 |
| TF | Factor: beta-catenin; motif: CTTTGATN | TF:M07599 | 6721 | 3.10E-02 | 372 | 105 |
| TF | Factor: Cdx-2; motif: NYMATAANN | TF:M10850 | 2154 | 3.10E-02 | 372 | 45 |
| TF | Factor: POU4F3; motif: CTAATYW | TF:M07061 | 5993 | 3.10E-02 | 372 | 96 |
| TF | Factor: TFF-1; motif: NNANCCACTTGAMNTT | TF:M01312 | 4653 | 3.20E-02 | 372 | 79 |
| TF | Factor: SRY; motif: AAACWAM; match class: 1 | TF:M00148_1 | 3665 | 3.30E-02 | 372 | 66 |
| TF | Factor: C/EBPbeta; motif: NATTGCRYAAYN | TF:M09597 | 2506 | 3.30E-02 | 372 | 50 |
| KEGG | Human T-cell leukemia virus 1 infection | KEGG:05166 | 208 | 3.40E-02 | 372 | 9 |
| TF | Factor: ESR1; motif: STGACCTN | TF:M12602 | 2862 | 3.40E-02 | 372 | 55 |
| TF | Factor: JunB; motif: NNNTGAGTCAY | TF:M09631 | 1628 | 3.40E-02 | 372 | 37 |
| TF | Factor: HOXA5; motif: ANGNTAATTANCNNAN | TF:M01452 | 3008 | 3.40E-02 | 372 | 57 |
| GO:BP | detection of stimulus | GO:0051606 | 337 | 3.50E-02 | 372 | 14 |
| GO:BP | negative regulation of hydrolase activity | GO:0051346 | 293 | 3.50E-02 | 372 | 13 |
| TF | Factor: Oct-1; motif: CWNAWTKWSATRYN | TF:M00162 | 2937 | 3.50E-02 | 372 | 56 |
| TF | Factor: GATA-6; motif: WGATAACGATCW | TF:M12208 | 3449 | 3.50E-02 | 372 | 63 |
| TF | Factor: AML2; motif: CCRCACCAYDN | TF:M01854 | 2372 | 3.50E-02 | 372 | 48 |
| TF | Factor: NKX2-2; motif: NNNCCACTCAANNN; match class: 1 | TF:M12456_1 | 2512 | 3.50E-02 | 372 | 50 |
| TF | Factor: ZNF7; motif: NCYYTGYCWNCWCTTR | TF:M12716 | 2166 | 3.60E-02 | 372 | 45 |
| GO:BP | cellular component disassembly | GO:0022411 | 432 | 3.70E-02 | 372 | 16 |
| GO:BP | Fc-gamma receptor signaling pathway | GO:0038094 | 31 | 3.70E-02 | 372 | 5 |
| TF | Factor: HOXD4; motif: NYMATTAN | TF:M10712 | 3380 | 3.70E-02 | 372 | 62 |
| TF | Factor: c-Ets-2; motif: NACCGGAAGYRCTTCCGGTN | TF:M11417 | 12989 | 3.70E-02 | 372 | 177 |
| GO:BP | regeneration | GO:0031099 | 174 | 3.80E-02 | 372 | 10 |
| TF | Factor: NeuroD; motif: NNSCWGCTGNSY | TF:M01288 | 4210 | 3.80E-02 | 372 | 73 |
| TF | Factor: PUR1; motif: GGGNCAGNN | TF:M01721 | 8254 | 3.80E-02 | 372 | 123 |
| TF | Factor: NF-kappaB; motif: NGGGGAMTTTCCNN | TF:M00194 | 2103 | 3.80E-02 | 372 | 44 |
| TF | Factor: AP-4:Fli-1; motif: RSCGGAWRCAGSTGN | TF:M08445 | 8339 | 3.80E-02 | 372 | 124 |
| TF | Factor: Sall1; motif: NGGTCCKRGKRA | TF:M05467 | 3757 | 3.80E-02 | 372 | 67 |
| TF | Factor: GR; motif: RGWACATWATGTWCY; match class: 1 | TF:M11847_1 | 3311 | 3.80E-02 | 372 | 61 |
| GO:BP | regulation of interleukin-10 production | GO:0032653 | 52 | 3.90E-02 | 372 | 6 |
| GO:BP | interleukin-10 production | GO:0032613 | 52 | 3.90E-02 | 372 | 6 |
| GO:BP | negative regulation of developmental process | GO:0051093 | 804 | 3.90E-02 | 372 | 23 |
| TF | Factor: Brachyury; motif: NTNNCANNNNRGTGTGAANN | TF:M09878 | 3240 | 3.90E-02 | 372 | 60 |
| TF | Factor: HPX42B; motif: NNYAATTANN | TF:M10679 | 1770 | 3.90E-02 | 372 | 39 |
| TF | Factor: Smad3; motif: NGNCAGACASNNN; match class: 1 | TF:M01888_1 | 571 | 3.90E-02 | 372 | 19 |
| TF | Factor: OCT-2; motif: ATGMATATGCWAAT | TF:M08836 | 2950 | 3.90E-02 | 372 | 56 |
| TF | Factor: ipf1; motif: NVSTAATTAC | TF:M01235 | 2878 | 3.90E-02 | 372 | 55 |
| GO:BP | positive regulation of secretion by cell | GO:1903532 | 254 | 4.00E-02 | 372 | 12 |
| TF | Factor: Brn-4; motif: NATTATGCAWGN | TF:M11921 | 1076 | 4.00E-02 | 372 | 28 |
| TF | Factor: SF-1; motif: NYYCAAGGYCA | TF:M10084 | 3390 | 4.00E-02 | 372 | 62 |
| TF | Factor: GSC2; motif: NNTAATCCNN | TF:M04325 | 3097 | 4.00E-02 | 372 | 58 |
| GO:MF | peptide antigen binding | GO:0042605 | 23 | 4.10E-02 | 372 | 4 |
| GO:MF | polysaccharide binding | GO:0030247 | 23 | 4.10E-02 | 372 | 4 |
| GO:BP | negative regulation of cell migration | GO:0030336 | 255 | 4.20E-02 | 372 | 12 |
| GO:CC | Fc-epsilon receptor I complex | GO:0032998 | 2 | 4.20E-02 | 372 | 2 |
| GO:CC | Fc receptor complex | GO:0032997 | 2 | 4.20E-02 | 372 | 2 |
| GO:CC | integrin alphaL-beta2 complex | GO:0034687 | 2 | 4.20E-02 | 372 | 2 |
| GO:CC | integrin alphaM-beta2 complex | GO:0034688 | 2 | 4.20E-02 | 372 | 2 |
| GO:CC | Toll-like receptor 1-Toll-like receptor 2 protein complex | GO:0035354 | 2 | 4.20E-02 | 372 | 2 |
| GO:CC | macrophage migration inhibitory factor receptor complex | GO:0035692 | 2 | 4.20E-02 | 372 | 2 |
| GO:MF | complement receptor activity | GO:0004875 | 9 | 4.20E-02 | 372 | 3 |
| GO:MF | T cell receptor binding | GO:0042608 | 9 | 4.20E-02 | 372 | 3 |
| TF | Factor: SPI1; motif: NNNAAAGAGGAAGTGANNNN; match class: 1 | TF:M12497_1 | 134 | 4.20E-02 | 372 | 9 |
| TF | Factor: FOXP1; motif: TNTGTTTMY | TF:M09933 | 4223 | 4.20E-02 | 372 | 73 |
| TF | Factor: NF-1B; motif: KCCAGANWN; match class: 1 | TF:M08825_1 | 903 | 4.20E-02 | 372 | 25 |
| TF | Factor: VDR; motif: GGGKNARNRRGGWSA | TF:M00444 | 9297 | 4.20E-02 | 372 | 135 |
| GO:BP | positive regulation of humoral immune response | GO:0002922 | 16 | 4.30E-02 | 372 | 4 |
| GO:BP | regulation of T cell differentiation | GO:0045580 | 141 | 4.30E-02 | 372 | 9 |
| KEGG | Fc epsilon RI signaling pathway | KEGG:04664 | 62 | 4.30E-02 | 372 | 5 |
| TF | Factor: HNF3; motif: NNNNNTRTTTRYTYWNKN | TF:M01012 | 1910 | 4.30E-02 | 372 | 41 |
| TF | Factor: Six-1; motif: CTCARRTTWCN | TF:M07466 | 2887 | 4.30E-02 | 372 | 55 |
| GO:BP | neutrophil mediated immunity | GO:0002446 | 32 | 4.40E-02 | 372 | 5 |
| GO:BP | positive regulation of cell death | GO:0010942 | 539 | 4.40E-02 | 372 | 18 |
| GO:MF | scavenger receptor activity | GO:0005044 | 44 | 4.40E-02 | 372 | 5 |
| TF | Factor: HOXA5; motif: ANGNTAATTANCNNAN; match class: 1 | TF:M01452_1 | 1143 | 4.40E-02 | 372 | 29 |
| TF | Factor: HNF-3alpha; motif: NNYTAWGTAAACAAAN; match class: 1 | TF:M11568_1 | 170 | 4.40E-02 | 372 | 10 |
| TF | Factor: CTCF; motif: ACCAGGKGGC | TF:M04727 | 5802 | 4.40E-02 | 372 | 93 |
| GO:BP | cellular response to external stimulus | GO:0071496 | 300 | 4.50E-02 | 372 | 13 |
| GO:BP | peptidyl-tyrosine phosphorylation | GO:0018108 | 345 | 4.50E-02 | 372 | 14 |
| KEGG | Chagas disease | KEGG:05142 | 95 | 4.50E-02 | 372 | 6 |
| TF | Factor: Tbx20; motif: SGAGGTGTGAGGSGR | TF:M07474 | 3181 | 4.50E-02 | 372 | 59 |
| TF | Factor: CDP; motif: ATCGATNNNNNNATCRAT | TF:M03951 | 3255 | 4.50E-02 | 372 | 60 |
| TF | Factor: msc; motif: NRACAGCTGTYN | TF:M11145 | 1024 | 4.50E-02 | 372 | 27 |
| TF | Factor: HOXB6; motif: NTAATKRC | TF:M10691 | 4386 | 4.50E-02 | 372 | 75 |
| TF | Factor: NFATc2; motif: TTTTCCA; match class: 1 | TF:M02265_1 | 1650 | 4.50E-02 | 372 | 37 |
| TF | Factor: MZF1; motif: NNAATCCCCANNN | TF:M12493 | 1330 | 4.50E-02 | 372 | 32 |
| GO:BP | integrin-mediated signaling pathway | GO:0007229 | 109 | 4.60E-02 | 372 | 8 |
| KEGG | Herpes simplex virus 1 infection | KEGG:05168 | 471 | 4.60E-02 | 372 | 14 |
| KEGG | Viral protein interaction with cytokine and cytokine receptor | KEGG:04061 | 63 | 4.60E-02 | 372 | 5 |
| TF | Factor: IRF-3; motif: GCNGTTTCCWGGAAACNGAAAC | TF:M11674 | 3929 | 4.60E-02 | 372 | 69 |
| TF | Factor: POU2F1; motif: ANKNRWATGSAAWYAW; match class: 1 | TF:M10033_1 | 794 | 4.60E-02 | 372 | 23 |
| TF | Factor: AR; motif: ARGAACANNNTGTNC | TF:M07204 | 4006 | 4.60E-02 | 372 | 70 |
| GO:BP | phosphate-containing compound metabolic process | GO:0006796 | 2574 | 4.70E-02 | 372 | 50 |
| GO:MF | virus receptor activity | GO:0001618 | 71 | 4.70E-02 | 372 | 6 |
| TF | Factor: CDX2; motif: NNGCAATAAANN | TF:M12513 | 2609 | 4.70E-02 | 372 | 51 |
| TF | Factor: POU2F3; motif: NNAYGCTNATS | TF:M11903 | 1395 | 4.70E-02 | 372 | 33 |
| TF | Factor: ZBRK1; motif: NGNNNGGTNAWAAAARRGCNG; match class: 1 | TF:M10468_1 | 3933 | 4.70E-02 | 372 | 69 |
| GO:BP | interleukin-12 production | GO:0032615 | 54 | 4.80E-02 | 372 | 6 |
| GO:BP | regulation of interleukin-12 production | GO:0032655 | 54 | 4.80E-02 | 372 | 6 |
| TF | Factor: Kaiso; motif: NTCCTGCNAN | TF:M01119 | 3633 | 4.80E-02 | 372 | 65 |
| TF | Factor: POU3F2; motif: NWTGCATAAWTTA | TF:M04078 | 3189 | 4.80E-02 | 372 | 59 |
| TF | Factor: EMX1; motif: NNTAATKANN | TF:M07988 | 2898 | 4.80E-02 | 372 | 55 |
| GO:BP | regulation of innate immune response | GO:0045088 | 218 | 4.90E-02 | 372 | 11 |
| GO:BP | positive regulation of lipoprotein particle clearance | GO:0010986 | 6 | 4.90E-02 | 372 | 3 |
| GO:BP | membrane raft distribution | GO:0031580 | 6 | 4.90E-02 | 372 | 3 |
| GO:BP | cellular response to bacterial lipoprotein | GO:0071220 | 6 | 4.90E-02 | 372 | 3 |
| GO:BP | response to bacterial lipopeptide | GO:0070339 | 6 | 4.90E-02 | 372 | 3 |
| GO:BP | cellular response to bacterial lipopeptide | GO:0071221 | 6 | 4.90E-02 | 372 | 3 |
| TF | Factor: Blimp-1; motif: NAGKGAAAGTGN | TF:M12243 | 1721 | 4.90E-02 | 372 | 38 |
| TF | Factor: Pit-1; motif: NMTTCATAAWTATWNMNA; match class: 1 | TF:M00802_1 | 797 | 4.90E-02 | 372 | 23 |
| TF | Factor: HSF2; motif: NGAANNWTCK | TF:M00147 | 4941 | 4.90E-02 | 372 | 82 |
| CORUM | C1q complex | CORUM:6418 | 2 | 5.00E-02 | 372 | 2 |
| CORUM | TLR1-TLR2 complex | CORUM:7048 | 2 | 5.00E-02 | 372 | 2 |
| CORUM | TLR1-TLR2-lipoprotein complex | CORUM:7049 | 2 | 5.00E-02 | 372 | 2 |
| GO:BP | peptidyl-tyrosine modification | GO:0018212 | 348 | 5.00E-02 | 372 | 14 |
| GO:MF | arachidonate 5-lipoxygenase activity | GO:0004051 | 2 | 5.00E-02 | 372 | 2 |
| GO:MF | chemokine (C-C motif) ligand 5 binding | GO:0071791 | 2 | 5.00E-02 | 372 | 2 |
| GO:MF | IgM binding | GO:0001791 | 2 | 5.00E-02 | 372 | 2 |

**Supplementary Table 6: KCL BrainBank enrichment for Cluster three.** GO:BP – Gene Ontology Biological Process, GO:CC – Gene Ontology Cellular Components, GO:MF – Gene Ontology Molecular Function, KEGG: Kyoto Encyclopaedia of Genes and Genomes, REAC: Reactome, TF: Transfac. MIRNA: miRTarBase CORUM: CORUM database of protein complexes.

| **Dataset** | **Sample ID** | **Cluster One** | **Cluster Two** | **Cluster Three** |
| --- | --- | --- | --- | --- |
| TargetALS | CGND.HRA.00217 | 0·0000 | 0·0000 | 1·0000 |
| TargetALS | CGND.HRA.00359 | 0·0000 | 0·0000 | 1·0000 |
| TargetALS | CGND.HRA.02228 | 0·0000 | 0·0000 | 1·0000 |
| TargetALS | CGND.HRA.02233 | 0·0000 | 0·0000 | 1·0000 |
| TargetALS | CGND.HRA.00223 | 0·0000 | 0·0000 | 1·0000 |
| TargetALS | CGND.HRA.00366 | 0·0000 | 0·0000 | 1·0000 |
| TargetALS | CGND.HRA.00587 | 0·0000 | 0·0000 | 1·0000 |
| TargetALS | CGND.HRA.00615 | 0·0000 | 0·0000 | 1·0000 |
| TargetALS | CGND.HRA.00089.2 | 0·0000 | 0·0000 | 1·0000 |
| TargetALS | CGND.HRA.01517 | 0·0000 | 0·0000 | 1·0000 |
| TargetALS | CGND.HRA.00604 | 0·0000 | 0·0002 | 0·9998 |
| TargetALS | CGND.HRA.01574 | 0·0002 | 0·0000 | 0·9998 |
| TargetALS | CGND.HRA.02239 | 0·0000 | 0·0002 | 0·9998 |
| TargetALS | CGND.HRA.01403 | 0·0001 | 0·0003 | 0·9996 |
| TargetALS | CGND.HRA.02227 | 0·0005 | 0·0001 | 0·9995 |
| TargetALS | CGND.HRA.00225 | 0·0006 | 0·0000 | 0·9994 |
| TargetALS | CGND.HRA.00083 | 0·0003 | 0·0010 | 0·9987 |
| TargetALS | CGND.HRA.01586.2 | 0·0002 | 0·0020 | 0·9978 |
| TargetALS | CGND.HRA.00334 | 0·0018 | 0·0012 | 0·9970 |
| TargetALS | CGND.HRA.00603 | 0·0021 | 0·0022 | 0·9957 |
| TargetALS | CGND.HRA.02427 | 0·0416 | 0·0015 | 0·9569 |
| TargetALS | CGND.HRA.01511 | 0·0011 | 0·0436 | 0·9553 |
| TargetALS | CGND.HRA.00215 | 0·0719 | 0·0007 | 0·9275 |
| TargetALS | CGND.HRA.01635 | 0·0811 | 0·0038 | 0·9151 |
| TargetALS | CGND.HRA.02252 | 0·1130 | 0·0007 | 0·8863 |
| TargetALS | CGND.HRA.00394 | 0·1197 | 0·0005 | 0·8797 |
| TargetALS | CGND.HRA.00733 | 0·1476 | 0·0007 | 0·8516 |
| TargetALS | CGND.HRA.01424 | 0·1467 | 0·0203 | 0·8330 |
| TargetALS | CGND.HRA.00664 | 0·1718 | 0·0005 | 0·8276 |
| TargetALS | CGND.HRA.00312 | 0·0120 | 0·1679 | 0·8201 |
| TargetALS | CGND.HRA.00393 | 0·1993 | 0·0001 | 0·8006 |
| TargetALS | CGND.HRA.00222 | 0·0027 | 0·2104 | 0·7869 |
| TargetALS | CGND.HRA.00561 | 0·2119 | 0·0067 | 0·7814 |
| TargetALS | CGND.HRA.00663 | 0·2177 | 0·0063 | 0·7760 |
| TargetALS | CGND.HRA.02428 | 0·2436 | 0·0001 | 0·7563 |
| TargetALS | CGND.HRA.00631 | 0·2443 | 0·0002 | 0·7555 |
| TargetALS | CGND.HRA.01568 | 0·2543 | 0·0052 | 0·7405 |
| TargetALS | CGND.HRA.01575 | 0·2639 | 0·0018 | 0·7343 |
| TargetALS | CGND.HRA.00599 | 0·0026 | 0·2822 | 0·7152 |
| TargetALS | CGND.HRA.01500 | 0·3084 | 0·0000 | 0·6916 |
| TargetALS | CGND.HRA.00586 | 0·2600 | 0·1167 | 0·6233 |
| TargetALS | CGND.HRA.01407 | 0·2472 | 0·1868 | 0·5661 |
| TargetALS | CGND.HRA.00624 | 0·3666 | 0·1009 | 0·5325 |
| TargetALS | CGND.HRA.00582 | 0·6016 | 0·0000 | 0·3984 |
| TargetALS | CGND.HRA.00313 | 0·0619 | 0·6137 | 0·3245 |
| TargetALS | CGND.HRA.00462 | 0·6421 | 0·0475 | 0·3104 |
| TargetALS | CGND.HRA.00748 | 0·6507 | 0·0950 | 0·2543 |
| TargetALS | CGND.HRA.02240 | 0·4191 | 0·3825 | 0·1984 |
| TargetALS | CGND.HRA.00430 | 0·8144 | 0·0005 | 0·1851 |
| TargetALS | CGND.HRA.00374 | 0·8247 | 0·0004 | 0·1749 |
| TargetALS | CGND.HRA.01320 | 0·7792 | 0·0472 | 0·1736 |
| TargetALS | CGND.HRA.00424 | 0·8274 | 0·0068 | 0·1659 |
| TargetALS | CGND.HRA.01398 | 0·8417 | 0·0018 | 0·1565 |
| TargetALS | CGND.HRA.00435 | 0·8915 | 0·0001 | 0·1084 |
| TargetALS | CGND.HRA.00488 | 0·8930 | 0·0000 | 0·1069 |
| TargetALS | CGND.HRA.00095 | 0·9025 | 0·0001 | 0·0974 |
| TargetALS | CGND.HRA.00461 | 0·9128 | 0·0044 | 0·0827 |
| TargetALS | CGND.HRA.01174 | 0·8919 | 0·0260 | 0·0821 |
| TargetALS | CGND.HRA.00226 | 0·9246 | 0·0086 | 0·0669 |
| TargetALS | CGND.HRA.01482 | 0·8987 | 0·0351 | 0·0662 |
| TargetALS | CGND.HRA.00250 | 0·0262 | 0·9138 | 0·0600 |
| TargetALS | CGND.HRA.00429 | 0·8988 | 0·0464 | 0·0548 |
| TargetALS | CGND.HRA.01518 | 0·9474 | 0·0001 | 0·0525 |
| TargetALS | CGND.HRA.00563 | 0·0014 | 0·9507 | 0·0480 |
| TargetALS | CGND.HRA.01415 | 0·9531 | 0·0000 | 0·0469 |
| TargetALS | CGND.HRA.00423 | 0·9591 | 0·0011 | 0·0399 |
| TargetALS | CGND.HRA.01394 | 0·9610 | 0·0002 | 0·0388 |
| TargetALS | CGND.HRA.02489 | 0·9662 | 0·0001 | 0·0336 |
| TargetALS | CGND.HRA.00285 | 0·4111 | 0·5641 | 0·0248 |
| TargetALS | CGND.HRA.01636 | 0·9683 | 0·0093 | 0·0224 |
| TargetALS | CGND.HRA.00065 | 0·5498 | 0·4288 | 0·0215 |
| TargetALS | CGND.HRA.01428 | 0·9009 | 0·0795 | 0·0196 |
| TargetALS | CGND.HRA.00358 | 0·9682 | 0·0141 | 0·0177 |
| TargetALS | CGND.HRA.00747 | 0·6360 | 0·3518 | 0·0122 |
| TargetALS | CGND.HRA.01175 | 0·9114 | 0·0801 | 0·0085 |
| TargetALS | CGND.HRA.02526 | 0·1310 | 0·8624 | 0·0066 |
| TargetALS | CGND.HRA.00342 | 0·1379 | 0·8581 | 0·0040 |
| TargetALS | CGND.HRA.01569 | 0·9959 | 0·0004 | 0·0036 |
| TargetALS | CGND.HRA.00220 | 0·9943 | 0·0024 | 0·0033 |
| TargetALS | CGND.HRA.00732 | 0·9952 | 0·0021 | 0·0026 |
| TargetALS | CGND.HRA.01493 | 0·9968 | 0·0006 | 0·0026 |
| TargetALS | CGND.HRA.00335 | 0·9191 | 0·0794 | 0·0015 |
| TargetALS | CGND.HRA.00367 | 0·4251 | 0·5735 | 0·0014 |
| TargetALS | CGND.HRA.00574 | 0·9234 | 0·0756 | 0·0010 |
| TargetALS | CGND.HRA.02905 | 0·9988 | 0·0002 | 0·0010 |
| TargetALS | CGND.HRA.00061 | 0·9860 | 0·0131 | 0·0008 |
| TargetALS | CGND.HRA.02904 | 0·9989 | 0·0005 | 0·0007 |
| TargetALS | CGND.HRA.00343 | 0·0080 | 0·9915 | 0·0005 |
| TargetALS | CGND.HRA.01321 | 0·9953 | 0·0042 | 0·0005 |
| TargetALS | CGND.HRA.01512 | 0·0669 | 0·9327 | 0·0004 |
| TargetALS | CGND.HRA.00351 | 0·9935 | 0·0061 | 0·0004 |
| TargetALS | CGND.HRA.00087 | 0·4071 | 0·5927 | 0·0003 |
| TargetALS | CGND.HRA.02170 | 0·0031 | 0·9966 | 0·0003 |
| TargetALS | CGND.HRA.02739 | 0·9997 | 0·0001 | 0·0003 |
| TargetALS | CGND.HRA.00600 | 0·5374 | 0·4624 | 0·0003 |
| TargetALS | CGND.HRA.02251 | 0·9327 | 0·0671 | 0·0002 |
| TargetALS | CGND.HRA.00350 | 0·9997 | 0·0000 | 0·0002 |
| TargetALS | CGND.HRA.01429 | 0·9998 | 0·0000 | 0·0002 |
| TargetALS | CGND.HRA.00436 | 0·9979 | 0·0020 | 0·0002 |
| TargetALS | CGND.HRA.00373 | 0·9998 | 0·0000 | 0·0002 |
| TargetALS | CGND.HRA.01557 | 0·9998 | 0·0000 | 0·0001 |
| TargetALS | CGND.HRA.00616 | 0·0934 | 0·9064 | 0·0001 |
| TargetALS | CGND.HRA.01481 | 0·0434 | 0·9565 | 0·0001 |
| TargetALS | CGND.HRA.00581 | 0·9998 | 0·0001 | 0·0001 |
| TargetALS | CGND.HRA.00638 | 0·9845 | 0·0154 | 0·0001 |
| TargetALS | CGND.HRA.01280 | 0·9998 | 0·0001 | 0·0001 |
| TargetALS | CGND.HRA.00147 | 0·9999 | 0·0000 | 0·0001 |
| TargetALS | CGND.HRA.00678 | 0·0362 | 0·9638 | 0·0001 |
| TargetALS | CGND.HRA.00219 | 0·9998 | 0·0001 | 0·0001 |
| TargetALS | CGND.HRA.01587 | 0·9694 | 0·0305 | 0·0001 |
| TargetALS | CGND.HRA.00556 | 0·8843 | 0·1157 | 0·0000 |
| TargetALS | CGND.HRA.01414 | 0·9999 | 0·0001 | 0·0000 |
| TargetALS | CGND.HRA.02221 | 0·9999 | 0·0001 | 0·0000 |
| TargetALS | CGND.HRA.00059 | 0·0050 | 0·9950 | 0·0000 |
| TargetALS | CGND.HRA.02234 | 0·9999 | 0·0000 | 0·0000 |
| TargetALS | CGND.HRA.00252 | 0·8953 | 0·1047 | 0·0000 |
| TargetALS | CGND.HRA.00575 | 0·9996 | 0·0004 | 0·0000 |
| TargetALS | CGND.HRA.02171 | 0·0015 | 0·9985 | 0·0000 |
| TargetALS | CGND.HRA.01629 | 0·9992 | 0·0008 | 0·0000 |
| TargetALS | CGND.HRA.01279 | 0·9998 | 0·0002 | 0·0000 |
| TargetALS | CGND.HRA.00134 | 0·9952 | 0·0048 | 0·0000 |
| TargetALS | CGND.HRA.00055 | 0·0000 | 1·0000 | 0·0000 |
| TargetALS | CGND.HRA.00677 | 0·0533 | 0·9467 | 0·0000 |
| TargetALS | CGND.HRA.00489 | 1·0000 | 0·0000 | 0·0000 |
| TargetALS | CGND.HRA.00562 | 0·9936 | 0·0064 | 0·0000 |
| TargetALS | CGND.HRA.00284 | 0·0321 | 0·9678 | 0·0000 |
| TargetALS | CGND.HRA.02222 | 0·9996 | 0·0004 | 0·0000 |
| TargetALS | CGND.HRA.02490 | 0·9999 | 0·0001 | 0·0000 |
| TargetALS | CGND.HRA.01399 | 0·9806 | 0·0194 | 0·0000 |
| TargetALS | CGND.HRA.02246 | 1·0000 | 0·0000 | 0·0000 |
| TargetALS | CGND.HRA.00564 | 0·0000 | 1·0000 | 0·0000 |
| TargetALS | CGND.HRA.02914 | 0·9964 | 0·0036 | 0·0000 |
| TargetALS | CGND.HRA.01532 | 1·0000 | 0·0000 | 0·0000 |
| TargetALS | CGND.HRA.01528 | 1·0000 | 0·0000 | 0·0000 |
| TargetALS | CGND.HRA.01531 | 1·0000 | 0·0000 | 0·0000 |
| TargetALS | CGND.HRA.00193 | 0·4984 | 0·5016 | 0·0000 |
| TargetALS | CGND.HRA.00093 | 0·7809 | 0·2191 | 0·0000 |
| TargetALS | CGND.HRA.00557 | 0·9959 | 0·0041 | 0·0000 |
| TargetALS | CGND.HRA.02520 | 0·9962 | 0·0038 | 0·0000 |
| TargetALS | CGND.HRA.02150 | 0·9999 | 0·0001 | 0·0000 |
| TargetALS | CGND.HRA.00321 | 1·0000 | 0·0000 | 0·0000 |
| TargetALS | CGND.HRA.00066 | 0·0000 | 1·0000 | 0·0000 |
| TargetALS | CGND.HRA.01505 | 0·0000 | 1·0000 | 0·0000 |
| TargetALS | CGND.HRA.01628 | 0·0003 | 0·9997 | 0·0000 |
| TargetALS | CGND.HRA.00249 | 0·0008 | 0·9992 | 0·0000 |
| TargetALS | CGND.HRA.00216 | 0·0313 | 0·9687 | 0·0000 |
| TargetALS | CGND.HRA.01384 | 0·0592 | 0·9408 | 0·0000 |
| TargetALS | CGND.HRA.01506 | 0·4441 | 0·5559 | 0·0000 |
| TargetALS | CGND.HRA.02644 | 0·8966 | 0·1034 | 0·0000 |
| TargetALS | CGND.HRA.00633 | 0·9712 | 0·0288 | 0·0000 |
| TargetALS | CGND.HRA.00068 | 0·9762 | 0·0238 | 0·0000 |
| TargetALS | CGND.HRA.00096 | 0·9858 | 0·0142 | 0·0000 |
| TargetALS | CGND.HRA.02915 | 0·9919 | 0·0081 | 0·0000 |
| TargetALS | CGND.HRA.01425 | 0·9973 | 0·0027 | 0·0000 |
| TargetALS | CGND.HRA.00637 | 0·9976 | 0·0024 | 0·0000 |
| TargetALS | CGND.HRA.01499 | 0·9986 | 0·0014 | 0·0000 |
| TargetALS | CGND.HRA.01494 | 0·9988 | 0·0012 | 0·0000 |
| TargetALS | CGND.HRA.01540 | 0·9992 | 0·0008 | 0·0000 |
| TargetALS | CGND.HRA.02151 | 0·9998 | 0·0002 | 0·0000 |
| TargetALS | CGND.HRA.00221 | 0·9999 | 0·0001 | 0·0000 |
| TargetALS | CGND.HRA.01539 | 1·0000 | 0·0000 | 0·0000 |
| TargetALS | CGND.HRA.01387 | 1·0000 | 0·0000 | 0·0000 |
| TargetALS | CGND.HRA.02740 | 1·0000 | 0·0000 | 0·0000 |
| TargetALS | CGND.HRA.01524 | 1·0000 | 0·0000 | 0·0000 |
| TargetALS | CGND.HRA.01527 | 1·0000 | 0·0000 | 0·0000 |
| TargetALS | CGND.HRA.00320 | 1·0000 | 0·0000 | 0·0000 |
| TargetALS | CGND.HRA.02245 | 1·0000 | 0·0000 | 0·0000 |
| TargetALS | CGND.HRA.01386 | 1·0000 | 0·0000 | 0·0000 |
| van Rheenen | GSM3077801 | 0·0037 | 0·0024 | 0·9939 |
| van Rheenen | GSM3078181 | 0·0107 | 0·0018 | 0·9876 |
| van Rheenen | GSM3078009 | 0·0150 | 0·0018 | 0·9831 |
| van Rheenen | GSM3078360 | 0·0188 | 0·0010 | 0·9803 |
| van Rheenen | GSM3078171 | 0·0511 | 0·0002 | 0·9487 |
| van Rheenen | GSM3077825 | 0·0360 | 0·0288 | 0·9352 |
| van Rheenen | GSM3077701 | 0·0591 | 0·0198 | 0·9211 |
| van Rheenen | GSM3077063 | 0·1036 | 0·0030 | 0·8934 |
| van Rheenen | GSM3078169 | 0·1080 | 0·0013 | 0·8907 |
| van Rheenen | GSM3077787 | 0·0056 | 0·1042 | 0·8902 |
| van Rheenen | GSM3078387 | 0·1374 | 0·0108 | 0·8518 |
| van Rheenen | GSM3078247 | 0·1339 | 0·0664 | 0·7997 |
| van Rheenen | GSM3076775 | 0·1991 | 0·0080 | 0·7928 |
| van Rheenen | GSM3078232 | 0·2814 | 0·0144 | 0·7042 |
| van Rheenen | GSM3077663 | 0·1590 | 0·1394 | 0·7016 |
| van Rheenen | GSM3076586 | 0·2931 | 0·0233 | 0·6836 |
| van Rheenen | GSM3077792 | 0·2797 | 0·0523 | 0·6680 |
| van Rheenen | GSM3078154 | 0·3290 | 0·0082 | 0·6628 |
| van Rheenen | GSM3080153 | 0·3518 | 0·0010 | 0·6472 |
| van Rheenen | GSM3080115 | 0·4577 | 0·0039 | 0·5384 |
| van Rheenen | GSM3078258 | 0·4350 | 0·0283 | 0·5368 |
| van Rheenen | GSM3079919 | 0·0362 | 0·4376 | 0·5263 |
| van Rheenen | GSM3080127 | 0·1154 | 0·3588 | 0·5259 |
| van Rheenen | GSM3080144 | 0·4786 | 0·0081 | 0·5134 |
| van Rheenen | GSM3078304 | 0·4159 | 0·0740 | 0·5101 |
| van Rheenen | GSM3079992 | 0·4114 | 0·0884 | 0·5002 |
| van Rheenen | GSM3077141 | 0·5054 | 0·0002 | 0·4944 |
| van Rheenen | GSM3078166 | 0·4804 | 0·0259 | 0·4938 |
| van Rheenen | GSM3080102 | 0·5332 | 0·0107 | 0·4561 |
| van Rheenen | GSM3077843 | 0·3086 | 0·2372 | 0·4542 |
| van Rheenen | GSM3080108 | 0·4893 | 0·0700 | 0·4408 |
| van Rheenen | GSM3078230 | 0·2677 | 0·2919 | 0·4404 |
| van Rheenen | GSM3080109 | 0·5049 | 0·0624 | 0·4327 |
| van Rheenen | GSM3076867 | 0·5569 | 0·0507 | 0·3924 |
| van Rheenen | GSM3078011 | 0·6374 | 0·0000 | 0·3626 |
| van Rheenen | GSM3080135 | 0·5016 | 0·1420 | 0·3565 |
| van Rheenen | GSM3078242 | 0·6667 | 0·0023 | 0·3310 |
| van Rheenen | GSM3079864 | 0·3975 | 0·2825 | 0·3200 |
| van Rheenen | GSM3078176 | 0·5652 | 0·1214 | 0·3134 |
| van Rheenen | GSM3078139 | 0·6918 | 0·0003 | 0·3080 |
| van Rheenen | GSM3077827 | 0·6977 | 0·0024 | 0·3000 |
| van Rheenen | GSM3080180 | 0·7062 | 0·0028 | 0·2910 |
| van Rheenen | GSM3078335 | 0·1420 | 0·5710 | 0·2870 |
| van Rheenen | GSM3077807 | 0·7227 | 0·0079 | 0·2694 |
| van Rheenen | GSM3077061 | 0·7338 | 0·0186 | 0·2477 |
| van Rheenen | GSM3078250 | 0·7408 | 0·0188 | 0·2404 |
| van Rheenen | GSM3076821 | 0·7602 | 0·0032 | 0·2366 |
| van Rheenen | GSM3076602 | 0·7629 | 0·0289 | 0·2082 |
| van Rheenen | GSM3079763 | 0·6445 | 0·1476 | 0·2079 |
| van Rheenen | GSM3080105 | 0·7899 | 0·0029 | 0·2073 |
| van Rheenen | GSM3080141 | 0·2733 | 0·5210 | 0·2057 |
| van Rheenen | GSM3077366 | 0·5752 | 0·2264 | 0·1984 |
| van Rheenen | GSM3080130 | 0·5239 | 0·2788 | 0·1974 |
| van Rheenen | GSM3079849 | 0·7195 | 0·0884 | 0·1922 |
| van Rheenen | GSM3076702 | 0·5332 | 0·2762 | 0·1906 |
| van Rheenen | GSM3077804 | 0·8160 | 0·0133 | 0·1707 |
| van Rheenen | GSM3080113 | 0·8291 | 0·0046 | 0·1663 |
| van Rheenen | GSM3077426 | 0·5327 | 0·3012 | 0·1661 |
| van Rheenen | GSM3079859 | 0·7555 | 0·0785 | 0·1660 |
| van Rheenen | GSM3079993 | 0·7902 | 0·0457 | 0·1642 |
| van Rheenen | GSM3079876 | 0·1340 | 0·7032 | 0·1628 |
| van Rheenen | GSM3077975 | 0·0310 | 0·8099 | 0·1592 |
| van Rheenen | GSM3076893 | 0·8184 | 0·0228 | 0·1587 |
| van Rheenen | GSM3076994 | 0·8415 | 0·0023 | 0·1563 |
| van Rheenen | GSM3078188 | 0·2632 | 0·5806 | 0·1562 |
| van Rheenen | GSM3077704 | 0·8419 | 0·0067 | 0·1514 |
| van Rheenen | GSM3078499 | 0·8579 | 0·0045 | 0·1376 |
| van Rheenen | GSM3079913 | 0·4348 | 0·4279 | 0·1373 |
| van Rheenen | GSM3077098 | 0·8612 | 0·0106 | 0·1282 |
| van Rheenen | GSM3076832 | 0·8278 | 0·0469 | 0·1253 |
| van Rheenen | GSM3077227 | 0·8487 | 0·0262 | 0·1251 |
| van Rheenen | GSM3076704 | 0·8709 | 0·0045 | 0·1245 |
| van Rheenen | GSM3078234 | 0·8703 | 0·0156 | 0·1141 |
| van Rheenen | GSM3079758 | 0·7136 | 0·1724 | 0·1140 |
| van Rheenen | GSM3076647 | 0·8495 | 0·0412 | 0·1092 |
| van Rheenen | GSM3080160 | 0·8175 | 0·0779 | 0·1047 |
| van Rheenen | GSM3079841 | 0·1158 | 0·7811 | 0·1031 |
| van Rheenen | GSM3078503 | 0·8965 | 0·0005 | 0·1029 |
| van Rheenen | GSM3079890 | 0·7220 | 0·1789 | 0·0991 |
| van Rheenen | GSM3078425 | 0·1305 | 0·7716 | 0·0979 |
| van Rheenen | GSM3079851 | 0·9000 | 0·0034 | 0·0966 |
| van Rheenen | GSM3078237 | 0·7455 | 0·1584 | 0·0961 |
| van Rheenen | GSM3077112 | 0·8704 | 0·0337 | 0·0959 |
| van Rheenen | GSM3078444 | 0·1233 | 0·7815 | 0·0952 |
| van Rheenen | GSM3079891 | 0·8039 | 0·1015 | 0·0946 |
| van Rheenen | GSM3080168 | 0·2405 | 0·6682 | 0·0914 |
| van Rheenen | GSM3076842 | 0·9093 | 0·0044 | 0·0862 |
| van Rheenen | GSM3076622 | 0·9121 | 0·0017 | 0·0862 |
| van Rheenen | GSM3078497 | 0·8650 | 0·0496 | 0·0855 |
| van Rheenen | GSM3077625 | 0·7189 | 0·1976 | 0·0835 |
| van Rheenen | GSM3077364 | 0·8432 | 0·0746 | 0·0822 |
| van Rheenen | GSM3079996 | 0·8422 | 0·0765 | 0·0813 |
| van Rheenen | GSM3078038 | 0·0025 | 0·9208 | 0·0767 |
| van Rheenen | GSM3077934 | 0·2723 | 0·6522 | 0·0755 |
| van Rheenen | GSM3077114 | 0·9118 | 0·0156 | 0·0726 |
| van Rheenen | GSM3077441 | 0·9111 | 0·0170 | 0·0719 |
| van Rheenen | GSM3076729 | 0·9306 | 0·0000 | 0·0694 |
| van Rheenen | GSM3079961 | 0·6985 | 0·2361 | 0·0654 |
| van Rheenen | GSM3080134 | 0·8637 | 0·0712 | 0·0651 |
| van Rheenen | GSM3076681 | 0·4997 | 0·4375 | 0·0629 |
| van Rheenen | GSM3079920 | 0·9151 | 0·0224 | 0·0625 |
| van Rheenen | GSM3078252 | 0·6968 | 0·2450 | 0·0582 |
| van Rheenen | GSM3076901 | 0·9417 | 0·0004 | 0·0579 |
| van Rheenen | GSM3077266 | 0·9111 | 0·0323 | 0·0566 |
| van Rheenen | GSM3079870 | 0·9272 | 0·0178 | 0·0550 |
| van Rheenen | GSM3078227 | 0·9474 | 0·0010 | 0·0516 |
| van Rheenen | GSM3080028 | 0·7241 | 0·2272 | 0·0487 |
| van Rheenen | GSM3076955 | 0·9415 | 0·0099 | 0·0486 |
| van Rheenen | GSM3080149 | 0·9115 | 0·0404 | 0·0481 |
| van Rheenen | GSM3076865 | 0·9390 | 0·0138 | 0·0472 |
| van Rheenen | GSM3079823 | 0·9274 | 0·0265 | 0·0461 |
| van Rheenen | GSM3079772 | 0·9194 | 0·0347 | 0·0458 |
| van Rheenen | GSM3077724 | 0·9526 | 0·0020 | 0·0454 |
| van Rheenen | GSM3078178 | 0·9510 | 0·0041 | 0·0450 |
| van Rheenen | GSM3080111 | 0·8830 | 0·0728 | 0·0443 |
| van Rheenen | GSM3079960 | 0·9224 | 0·0335 | 0·0441 |
| van Rheenen | GSM3080003 | 0·0805 | 0·8763 | 0·0432 |
| van Rheenen | GSM3078068 | 0·8844 | 0·0724 | 0·0432 |
| van Rheenen | GSM3078502 | 0·9554 | 0·0015 | 0·0431 |
| van Rheenen | GSM3079834 | 0·9415 | 0·0167 | 0·0418 |
| van Rheenen | GSM3076916 | 0·8389 | 0·1200 | 0·0411 |
| van Rheenen | GSM3076843 | 0·9582 | 0·0026 | 0·0392 |
| van Rheenen | GSM3077641 | 0·0219 | 0·9400 | 0·0382 |
| van Rheenen | GSM3077721 | 0·9399 | 0·0223 | 0·0378 |
| van Rheenen | GSM3076607 | 0·8571 | 0·1065 | 0·0364 |
| van Rheenen | GSM3080006 | 0·7604 | 0·2039 | 0·0357 |
| van Rheenen | GSM3078036 | 0·7817 | 0·1831 | 0·0352 |
| van Rheenen | GSM3077246 | 0·6869 | 0·2782 | 0·0349 |
| van Rheenen | GSM3076662 | 0·9660 | 0·0000 | 0·0340 |
| van Rheenen | GSM3076798 | 0·9655 | 0·0008 | 0·0337 |
| van Rheenen | GSM3080148 | 0·9636 | 0·0032 | 0·0332 |
| van Rheenen | GSM3079989 | 0·9468 | 0·0204 | 0·0328 |
| van Rheenen | GSM3077165 | 0·9674 | 0·0001 | 0·0325 |
| van Rheenen | GSM3080023 | 0·7293 | 0·2391 | 0·0316 |
| van Rheenen | GSM3078510 | 0·9536 | 0·0155 | 0·0309 |
| van Rheenen | GSM3077079 | 0·9684 | 0·0010 | 0·0306 |
| van Rheenen | GSM3076794 | 0·9694 | 0·0000 | 0·0306 |
| van Rheenen | GSM3077008 | 0·9706 | 0·0001 | 0·0293 |
| van Rheenen | GSM3079925 | 0·8076 | 0·1635 | 0·0289 |
| van Rheenen | GSM3076929 | 0·6708 | 0·3007 | 0·0286 |
| van Rheenen | GSM3080034 | 0·9547 | 0·0181 | 0·0272 |
| van Rheenen | GSM3079812 | 0·8256 | 0·1477 | 0·0267 |
| van Rheenen | GSM3079945 | 0·8680 | 0·1057 | 0·0262 |
| van Rheenen | GSM3079779 | 0·9657 | 0·0087 | 0·0256 |
| van Rheenen | GSM3078190 | 0·1535 | 0·8209 | 0·0256 |
| van Rheenen | GSM3080119 | 0·9248 | 0·0499 | 0·0253 |
| van Rheenen | GSM3078013 | 0·8779 | 0·0969 | 0·0252 |
| van Rheenen | GSM3076753 | 0·9385 | 0·0375 | 0·0240 |
| van Rheenen | GSM3077172 | 0·9503 | 0·0276 | 0·0222 |
| van Rheenen | GSM3076767 | 0·9206 | 0·0574 | 0·0220 |
[truncated: 34,996 more chars]
